# Effects of preconception nutrition interventions on pregnancy and birth outcomes in South Asia: a systematic review

**DOI:** 10.1016/j.lansea.2025.100580

**Published:** 2025-04-24

**Authors:** Naomi M. Saville, Sophiya Dulal, Faith Miller, Danielle Schoenaker, Ranadip Chowdhury, Avishek Hazra, Jane Hirst, Zivai Murira, Vani Sethi

**Affiliations:** a Institute for Global Health, University College London, London, UK; b School of Health Sciences, Western Sydney University, Sydney, Australia; c School of Human Development and Health, Faculty of Medicine, University of Southampton, Southampton, UK; d MRC Lifecourse Epidemiology Centre, University of Southampton, Southampton, UK; e NIHR Southampton Biomedical Research Centre, University of Southampton and University Hospital Southampton NHS Foundation Trust, Southampton, UK; f Society for Applied Studies, New Delhi, India; g Population Council Consulting, New Delhi, India; h The George Institute for Global Health, School of Public Health, Imperial College London, UK; i United Nations Children's Fund (UNICEF) Regional Office for South Asia (ROSA), Kathmandu, Nepal

**Keywords:** Preconception nutrition, South asia, Systematic review, Birth outcomes, Pregnancy outcomes, Micronutrient supplementation, Food supplementation, Complex intervention

## Abstract

Undernutrition amongst reproductive age women, low birth weight, small for gestational age and preterm birth present significant health burdens in South Asia which interventions in pregnancy alone have not resolved. Effectiveness of preconception nutrition interventions is not well-documented. This systematic review summarises evidence on the effect of preconception nutrition interventions on pregnancy and birth outcomes in South Asia. We found highly heterogeneous evidence across four micronutrient supplementation, two food supplementation, and three complex interventions trials. Preconception micronutrient supplementation alone did not affect birth size, but food supplementation was effective with and without multiple micronutrients, especially when initiated at least 90 days before conception. Combined health, nutrition, psychosocial care, and WaSH interventions addressing determinants at multiple levels were most effective. However intensive delivery by project employees poses problems for scale-up. More robust South Asian preconception intervention trials to identify scalable interventions that are effective in real-world delivery settings are needed.

**Funding:**

UNICEF Regional Office for South Asia contract number 43384734.

## Introduction

The burden of malnutrition in all forms among reproductive-aged women is high and widely varying across South Asia, as highlighted in our recent evidence review.^1^ Thirty-five percent of South Asian adolescent girls and women aged 15–49 years are <150 cm in height and 11% are <145 cm.^2^ Underweight, short stature, and micronutrient deficiencies contribute to the persistent public health challenges of low birth weight (LBW), small for gestational age (SGA) and preterm birth which affect 24.4%,^3^ 40.9% and 13.3% respectively.^4^ Some interventions during pregnancy, such as providing micronutrients, balanced energy/protein, nutrition education, and antimalarials, have shown limited effectiveness in reducing LBW and/or SGA births.^5^^,^^6^ Although antenatal care uptake has improved,^7^ half of pregnant women in South Asia do not access antenatal care until the second trimester of pregnancy,^8^ which is too late to improve foetal nutrition. Epigenetic modifications in the eggs and sperm, which are affected by the nutritional status of the parents before conception, have lifelong and intergenerational health impacts.^9^ Rudimentary organs are established within five weeks’ gestation,^10^ (often before pregnancy has been detected), and most organ development is complete before 10 weeks. Therefore, nutrition interventions to optimise pregnancy and birth outcomes should start before conception, taking a ‘1000 days + preconception’ approach.^11^^,^^12^

There is growing evidence regarding potential preconception interventions globally,^13^, ^14^, ^15^, ^16^, ^17^, ^18^, ^19^ but the effectiveness of preconception nutrition interventions in reducing adverse pregnancy and birth outcomes in South Asia is not documented. A review of preconception nutrition interventions focused upon South Asia alone is warranted because of the disproportionate burdens of low birth weight, SGA,^3^ preterm birth,^4^ neonatal mortality,^4^^,^^20^ adolescent pregnancy,^21^ anaemia,^22^ type-2 diabetes^23^ and its complex developmental origins associated with the South Asian ‘thin-fat’ baby phenotype,^24^ which may mean that interventions that work in other settings may not be appropriate to the South Asian context. Preconception interventions are often integrated into broader initiatives, such as “healthy transitions for adolescents” and “pre-pregnancy programmes” of maternal and reproductive health packages for couples.^25^ Previous systematic reviews of preconception health and care interventions have not sufficiently focused on nutritional interventions, and include few South Asian studies.^13^^,^^17^^,^^26^ In this paper, we aimed to synthesise evidence on the effect of preconception nutrition interventions conducted in South Asia on the nutritional status of pregnant women during pregnancy and birth outcomes (such as LBW, SGA, and preterm). We also aimed to explore mechanisms or pathways that explain how or why the interventions were (or were not) successful by examining intervention characteristics.

## Methods

### Search strategy and selection criteria

This systematic review was reported in accordance with the Preferred Reporting Items for Systematic Reviews and Meta-Analyses (PRISMA)^27^ (Supplementary File S1). We used the Population Intervention Comparison Outcome and Study type (PICOS) framework to define eligibility criteria (Table 1).

**Table 1:** PICOS framework to determine study eligibility.

Population	*Inclusion criteria* Women and girls: - Living in South Asia (Bangladesh, Bhutan, India, Nepal, Pakistan, Sri Lanka) - of reproductive age (10–49 years) who were either not known to be currently pregnant at the time of recruitment, or studies in which most participants were not pregnant - having outcome data from a subsequent pregnancy *Exclusion criteria* - who had not participated in an intervention study addressing preconception nutrition - without an outcome from a subsequent pregnancy - who are currently pregnant - who are not of reproductive age (<10 or >49 years)
Intervention	*Inclusion criteria* Preconception nutrition intervention in at least one study arm. *Exclusion criteria* No preconception nutrition intervention tested - Interventions delivered during pregnancy but not preconceptually - Interventions not addressing nutritional status
Comparison	*Inclusion criteria* Women and girls: - not receiving an intervention addressing preconception nutrition - receiving an intervention outside of the preconception period - receiving an intervention addressing factors other than nutrition *Exclusion criteria* - No comparison group
Outcome	*Inclusion criteria* At least one outcome reported from a subsequent pregnancy, including health and nutrition from the prenatal or neonatal period. The primary outcome is nutritional status among neonates including but not limited to: - Low birth weight (LBW) - Birth weight - Preterm birth - Small for Gestational Age (SGA) Outcomes may also include maternal nutritional status during pregnancy, including but not limited to: - Gestational weight gain - Gestational diabetes - Maternal anthropometry (weight, Mid Upper-Arm Circumference (MUAC), height) - Anaemia during pregnancy - Micronutrient deficiencies during pregnancy *Exclusion criteria* - Measures of infant or maternal nutrition outside the neonatal period >28 days - Other, non-nutritional measures of pregnancy or newborn health
Study type	*Inclusion criteria* - Randomised and non-randomised controlled trials of interventions to improve preconception nutrition in South Asia among reproductive aged women and girls - Published since 2000 in English - Data collected in South Asia - Secondary analyses of data from randomised or non-randomised intervention trials *Exclusion criteria* - Intervention trials with no control group - Studies published before 2000 - Data collected outside of South Asia - Reporting duplicate data - Published in a language other than English

This review protocol was registered on PROSPERO on 23 August 2023 (registration number: CRD42023398938).^28^ The protocol can be accessed at https://www.crd.york.ac.uk/prospero/display_record.php?ID=CRD42023398938.

There were two amendments to the protocol on 29 August 2023 we added inclusion of non-randomised controlled trials and on 20 November 2023 we changed the outcome definitions to focus on pregnancy/neonatal/birth outcomes (excluding child outcomes up to 19 years) as the review was too broad. We also added more specific terms in our searches (whilst screening titles) and permitted studies that collected data before 2000 if they were published after in or after 2000.

The specific research questions were: i) What are the effects of preconception nutrition interventions in South Asia upon the nutritional status of women (during pregnancy) and birth outcomes (such as LBW, SGA, and preterm) in South Asia? ii) What factors or impact pathways affect their success?

On 12th September 2023, one researcher (FM) searched Ovid (Medline, EMBASE, and Global Health), Web of Science, Global Index Medicus, and Cochrane Library databases to identify studies published since the year 2000 in the English language. Search terms included Medical Subject Headings (MeSH) terms or equivalent and free text under the themes of “Preconception period”, “Nutrition”, and “South Asia”. We undertook one search to answer a series of research questions presented here and elsewhere.^1^ We undertook forward and backward citation searching to capture resources citing or being cited by the included literature.^29^ Supplementary File S2 provides the complete search strategies across all databases. Supplementary File S3 provides details of article screening, selection, risk of bias assessments,^30^, ^31^, ^32^ data extraction and synthesis methods.

To map potential pathways to impact, we summarised which determinants of malnutrition from the United Nations Children's Fund (UNICEF) 2021 nutrition framework^33^ were being addressed by interventions (Supplementary File S4). Following the UNICEF framework, determinants being addressed by each intervention were categorized as enabling (governance, resources, norms), underlying (food, practices, services), or immediate (diets, care). We also mapped how each intervention fits into Partap et al., ’s 2022 conceptual framework of preconception health, nutrition, social, and Water Sanitation and Hygiene (WASH)-related intervention pathways at contextual, household, and individual levels (Supplementary File S5).^13^

## Results

### Preconception nutrition interventions

**Fig. 1:**
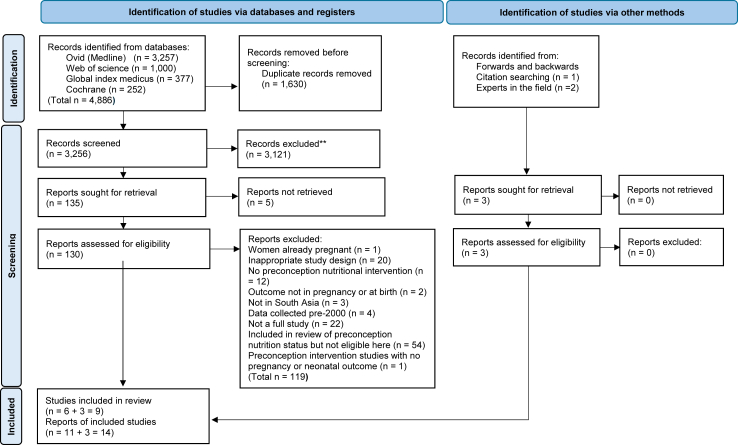
PRISMA diagram.

Our review included 14 studies published from January 2000 to December 2023 (Fig. 1, Table 2, Supplementary File S6). There were six individually randomised controlled trials (RCTs), one cluster-randomised controlled trial (cRCT),^37^ and two non-randomised studies.^36^^,^^44^ Nine intervention packages included four micronutrient supplementation interventions,^37^^,^^38^, ^45^, ^46^ two food supplements,^39^^,^^40^ and three complex interventions which combined food and/or micronutrient supplements with health, social, psychosocial and/or Water Sanitation and Hygiene (WASH) intervention components.^36^^,^^41^^,^^44^ Six were tested in India,^36^, ^38^, ^44^^,^^39^^,^^41^^,^^44^^,^^46^ one in Nepal,^37^ one in Bangladesh,^45^ and one across India, Pakistan, Guatemala, and the Democratic Republic of Congo (DRC).^40^ Four trials had published protocols.^35^, ^42^, ^43^, ^47^ Sample size varied from 226^46^ to 13,500.^41^ Hambidge 2019’s multi-country trial^40^ had three^48^, ^49^, ^50^ and Potdar 2014’s food supplementation trial^39^ had two^51^^,^^52^ additional sub-studies related to their interventions. We replaced one study presented in government report^53^ with a peer reviewed paper published in 2024.^36^

**Table 2:** Characteristics of South Asian preconception nutrition intervention study meeting the inclusion criteria.

First author/year	Country, City/Region, and Study Dates	Study design sample size	Participants characteristics	Timing of preconception intervention	Intervention/s	Comparator	Nutritional outcomes in pregnancy	Nutritional outcomes at birth and preterm
**1. Preconception micronutrient supplementation**								
ICMR 2000^30^	**India** Bangalore, Mumbai, Lucknow, New Delhi, Pune. 1988–1991	Double blind, randomised, placebo-controlled trial 2 arms **N** = 466	Women with previous childbirth with open neural tube defect^34^ who planned to have another child. Mean age was 25.69 ± 4.12 in Arm 1 and 26.07 ± 3.85 in Arm 2.	Daily supplement provided at least 1 month before conception and three months after conception.	**Arm 1:** Multivitamin supplement in preconception and pregnancy: ferrous sulphate 120 mg, calcium phosphate 240 mg, vitamins A 4000 IU, D 400 IU, B1 2.5 mg, B2 2.5 mg, B6 2 mg and C 40 mg, nicotinamide 15 mg, folic acid 4 mg and zinc 10 mg.	**Arm 2:** Placebo capsule containing ferrous sulphate 120 mg and calcium phosphate 240 mg.		**Secondary outcome:** - Low Birth Weight (LBW)
Katz 2000^27^ (protocol detail^35^)	**Nepal** Sarlahi, Plains. Jul 1994–Apr 1997	Double blind, cluster randomised, placebo controlled trial (cRCT). 3 arms **N** = 43,559 enrolled.	Married women of childbearing age (identified through baseline census).	Timing of preconception supplementation not provided.	Micronutrient supplement weekly doses in preconception and pregnancy. **Arm 1:** Vitamin A (7000 μg retinol equivalents as retinyl palmitate). **Arm 2:** 42 mg all-trans-b-carotene in gelatine capsules with 5 mg dL-a-tocopherol.	**Arm 3:** Weekly doses of peanut oil and 5 mg dL-a-tocopherol.	**Secondary outcome:** - Retinol (mmol/L) and deficiency <70 mmol/L (%) at four months gestation.	**Secondary outcome:** - Prevalence of preterm birth (gestation <37 week).
Khambalia 2009^31^	**Bangladesh** Kaliganj, Gazipur district. Mar 2007–Feb 2008	Individually randomized, double-blind, controlled trial (RCT). 2 arms N = 272 women	Nulliparous, non-pregnant women aged <40 years, married, living in household >6 months, living with husband, not using implant or not having surgery to prevent pregnancy, and had not used iron supplements in last three months.	Most women (n = 57) started supplementation before conception (mean 72.9 ± 57.8 days). n = 31 started mean 26.3 ± 12.3 days *after* conception. Median days from start of supplementation to conception: 25.5 (IQR −16.5 to 82.5) days.	**Arm 1:** Micronutrient supplement provided in preconception until pregnancy detected: Daily nutrient ‘sprinkles’ containing iron (60 mg as ferrous fumarate) and folic acid (400 mcg)	**Arm 2:** Micronutrient supplement provided in preconception until pregnancy detected: Daily nutrient ‘sprinkles’ containing 400 μg folic acid alone.	**Primary outcomes:** - **Haemoglobin** (Hb) (g/L) - **Plasma ferritin** (lg/L) - **Plasma transferrin recepto**r (mg/L) **Secondary outcomes:** - C-reactive protein (mg/L) - Plasma folate (nmol/L) - Anaemia (%)–Hb < 110 g/L - Iron deficiency (%)-plasma ferritin concentration <12 lg/L. - Iron deficiency and anaemia (%)- Hb < 120 g/L and - Plasma ferritin <12 lg/L.	
D’Souza 2021^32^ (protocol^36^)	**India** Pune Sep 2012–Feb 2020	Individually randomised placebo-controlled trial (RCT). 3 arms **N** = 226 female participants	Adolescents (∼16–18 y) girls and boys born of the original Pune Maternal Nutrition Study set up in 1993.	Duration of the supplement was 36 months in each arm, but it is not stated how long before pregnancy the intervention was started.	Micronutrient supplement daily in preconception and pregnancy. **Arm 1:** 2 μg B12 **Arm 2**: 2 μg B12 plus multiple micronutrients (MMN) and 20 g milk protein.	**Arm 3:** Placebo capsules daily in preconception and pregnancy.	**Secondary outcomes:** At 28 weeks’ gestation - Hb (gm/dL) - Vitamin B12 (pM) - Holo-TC (pM) - Folate (nM) - Vitamin B2 (pM). - Vitamin B6-pyridoxal-5-phospate (pM) - Vitamin B6-pyridoxal (pM) - Homocysteine (μmol/L)	**Secondary outcomes:** - Birth weight (gm) - Birth Length (cm) - Head circumference (cm) - Gestational age (weeks) - Cord micronutrients: Vitamin B12 (pM) Holo-TC (pM) Folate (nM) Vitamin B2 (pM) Vitamin B6-pyridoxal-5-phospate (pM) Vitamin B6-pyridoxal (pM) Homocysteine (μmol/L) Brain Derived Neurotrophic Factor (BDNF) (pg/ml).
**2. Preconception food supplementation**								
Hambidge 2019a^37^ (protocol^38^)	**India, Pakistan, DRC, Guatemala** Small town/rural sites in four countries: (Belgaum, Karnataka) Pakistan (Thatta, Sind Province) Democratic Republic of Congo (DRC, Equateur Province), and Guatemala (Chimaltenango Department) Dec 2013–Mar 2017.	Individually randomized, longitudinal, non-masked (non-blinded), multisite controlled efficacy trial (RCT). 3 arms **N** = 7387 women **n** = 1823 in India. **n** = 2015 in Pakistan **n** = 1808 in Guatemala **n** = 1741 in DRC.	Women/girls aged 16–35 years with parity 0–5, an expectation to have first or additional pregnancy within next 18 months, without intent to use contraception, agree to hospital delivery, has Hb > 8 g/dL, and has no history of pre-eclampsia or prolonged labour associated with cephalopelvic disproportion.	Required intervention exposure at least three months before pregnancy.	Fortified food supplement: 20 g lipid-based nutrient supplement (LNS) + non-fortified LNS Supplement 2 if Body mass index (BMI) < 20 kg/m^2^ (which provided 300 kcal and 11 g protein). **Arm 1:** Daily LNS commencing upon enrolment until delivery. Analysis of subjects receiving supplement from three months prior to conception **Arm 2:** Daily LNS commencing second and third trimesters of pregnancy and was stopped at delivery.	**Arm 3:** Routine care.		**Primary outcome:** - LAZ at birth <48 h **Secondary outcomes:** - Weight-for-age (WAZ), - Head Circumference-for-age (HCAZ), - BMI-for-age (BMIAZ) z score - Weight to length ratio-for-age z score (WLRAZ), - Proportions of infants with z scores < −1 and < −2 of GAA adjusted LAZ, WAZ, and HCAZ, WLRAZ - Low Birth Weight (LBW) (<2500 g), - SGA - Preterm birth (PTB)
Hambidge 2019b^39^	**India, Pakistan, Guatemala**	Sub study of Women First. **N** = 1465 maternal-newborn dyads. **n** = 515 in India. **n** = 457 in Pakistan. **n** = 493 in Guatemala.	As above but analysis Included maternal-newborn dyads who had gestational age determined from crown-rump length in the first trimester and newborn anthropometric measures (<48 h from delivery).	As above.	As above.	As above.		**Substudy of** **Primary outcomes:** - LAZ **Secondary outcomes:** - WAZ - WLRAZ
Dhaded 2020^40^	**India and Pakistan only** Study duration: Jan 2014–Mar 2017.	Sub study of Women First. 3 arms **N** = 972	Newborn of mothers who were poor, rural, of any nutritional status.	As above.	As above.	As above.		**Substudy of** **Primary outcome:** - LAZ **Secondary outcomes:** Continuous outcomes: - Newborn length - Weight - Head circumference - WAZ - HCAZ - WLRAZ Categorical outcomes: - SGA - Weight <10th percentile for gestational age), - LBW, - Preterm - LAZ < −2, - WAZ < −2, WLZ < −2 and–- WLRAZ < −2. - RR for Z-scores < −1
Young 2021^41^	**India, Pakistan, Guatemala** Study duration: Dec 2013–Dec 2016.	Sub study of Women First Trial. **N** = 872 **n** = 214 in India **n** = 323 in Pakistan **n** = 335 in Guatemala.	Women aged 16–35 years.	As above.	As above.	As above.	**Substudy outcomes:** - Urinary iodine concentration - Urinary creatinine concentrations	**Substudy outcomes:** - Gestational age adjusted z-scores: LAZ, WAZ, HCAZ and WLRAZ (INTERGROWTH-21st foetal growth charts)
Potdar 2014^33^	**India** Bandra, Khar, Santa Cruz, and Andheri slum areas of the city of Mumbai. Jan 2006–May 2012	Nonblinded, individually randomized controlled efficacy trial (RCT). 2 arms **N** = 6513 women enrolled. **n** = 2291 women followed up through pregnancy. **n** = 1094 birth outcomes.	Women aged <40 years who were married, not pregnant, not sterilized, planning to have more children, and intending to deliver in Mumbai. Median (IQR) age: 25^22^^,^^28^ year.	≥90 days before the last menstrual period.	**Food supplement (Arm 1) from 90+ days before conception and during pregnancy:** observed consumption (six days/week) of snack containing fresh and dried green leafy vegetables, milk, and dried fruit (containing 10–23% of the WHO/FAO Reference Nutrient Intake (RNI) for b-carotene, riboflavin, folate, vitamin B-12, calcium, and iron, and 0.69 MJ energy and 6.4 g protein).	**Control (Arm 2) from 90+ days before conception and during pregnancy:** snacks made from low-micronutrient vegetables such as potato, tapioca, and onion. Contained≤1% of RNI for b-Carotene, Riboflavin (mg), Folate (mg) and Vitamin C (mg). 7% of RNI for Vitamin B-12 (mg), 2% for Calcium (mg) and 5% for Iron (mg). 0.37 MJ energy and 2.4 g protein.		**Primary outcomes:** - Birth weight - LBW **Secondary outcomes:** - Gestational age - SGA - Newborn body measurements: chest circumference, abdomen circumference, MUAC, subscapular skinfold, and triceps skinfold.
Sahariah 2016^42^ (unpublished protocol is annexed)	As above	As above. **N** = 6513 women enrolled. **n** = 1008 women attended for an oral-glucose-tolerance test.	As above.	≥90 days before the last menstrual period. However, from Dec 2008 allowed women <3 months also.	As above.	As above.	**Substudy outcomes:** - Gestational diabetes mellitus, - Fasting and 120-min glucose concentrations, - Fasting insulin concentration.	
Lawande 2018^43^	As above	As above. **N** = 6513 women enrolled. **n** = 1677 foetal outcomes.	As above.	≥90 days before the last menstrual period.	As above.	As above.	**Substudy outcomes** Foetal growth measures: - Crown-rump length at 9–12 weeks, - Measures at 2nd and 3rd visit at 19–21 and 28–32 weeks’ gestation respectively (and on 1st visit if it was ≥13 weeks): Foetal head circumference Biparietal diameter Femur length, and Abdominal circumference.	
**3. Preconception complex interventions**								
Taneja 2022^44^ (protocol^45^)	**India** Low and middle-income neighbourhoods of Delhi. Jul 2017–Jun 2021.	Individually randomized controlled trial with factorial design. 4 arms **N** = 13,500 women enrolled in preconception period.	Women aged 18–30 years, married and living with their husband, with 0–1 child who intend to have a/another child	Median days from enrolment to conception: 126 days (IQR 33–275 days) in preconception arms, and 162 days (IQR 50–311 days in no preconception, (control) arm.	Complex intervention combining i) Health: screening/treatment ii) Nutrition: screening/treatment of malnutrition and anaemia: Iron Folic Acid (IFA) or MMN, locally prepared snacks, egg or milk iii) Psychosocial support. iv) WASH promotion **Arm 1:** The preconception, pregnancy, and early childhood interventions. **Arm 2:** The preconception interventions only. **Arm 3:** Pregnancy, and early childhood interventions.	**Arm 4:** Routine care	**Secondary outcomes** - Weight (low BMI<18.5) - Anaemia status (Hb) - Gestational weight gain	**Primary outcomes:** - **LBW** - **SGA** (birth weight centile <10th using INTERGROWTH-21st standards), - Mean **birth weight** and **length** and - **Preterm** (ultrasound confirmed gestational age at birth <37 completed weeks)
Kumar 2023^28^ (protocol^46^)	**India** Poorest areas in four districts Purnia in Bihar, Bastar in Chhattisgarh, and Angul and Koraput in Odisha. 2016–2022	Cross-sectional baseline and endline survey data analysis for evaluation of a non-randomised controlled trial. 2 arms N = 16,741 adolescent girls, 6844 pregnant women	Adolescent girls (10–19 years), pregnant women (15–49 years), and mothers of children under age two years (15–49 years).	No information provided.	**Arm 1:** Complex intervention using Community-led Participatory Learning Action (PLA) meetings with Self Help Groups (SHGs), village organisations, and community nutrition microplanning. Combined 14 nutrition-specific and nutrition- sensitive interventions in the domains of - Access to government rations and IFA - Uptake of health services - WASH - Dietary change - Nutrition sensitive Social and Behaviour Change. System strengthening (same as described in to Arm 2–Control).	**Arm 2:** - Weekly IFA supplementation via National Iron Plus Initiative - System strengthening: 1. Strengthening Village Health Sanitation and Nutrition Day (VHSNDs). 2. Strengthening adolescent health days. 3. An extended VHSND every six months for newlyweds and women. 4. Annual training/follow-up with food security, Integrated Child Development Services and WASH service providers. 5. Regular review meetings.	**Primary outcomes:** - MUAC <23 cm among pregnant women	
Doke 2024^29^ (protocol/government report^47^)	Nashik district, Maharashtra State, India Apr 2018–Jul 2021	Community-based implementation study 2 arms N = 7875 women enrolled **n** = 3601 pregnancy outcomes.	Women aged 15–49 years aspiring to have a baby. (Actual age of enrolled women 14.42–45.5 years; mean 23.2 ± 3.7).	No information provided.	**Arm 1:** Complex preconception care intervention involving: - Health screening/treatment - Anaemia prevention/treatment with IFA - Family planning services - Multi-channel Behaviour Change Communication at VHSNDs and home - Tailored counselling for BMI optimisation - Nutrition-sensitive counselling	**Arm 2:** Routine care.		**Secondary outcomes:** - LBW (birth weight <2500 g) - Preterm birth

Outcomes are allocated as primary and secondary as per authors classification and outcomes relating to additional studies are labelled as ‘substudy outcomes’.

BMI, Body mass index; HCAZ, Head Circumference for-age Z-score; IFA, iron-folic acid; LBW, Low Birth Weight; LAZ, Length-for-age Z-score; LNS, lipid-based nutrient supplement; RCT, Randomised Controlled Trial; MMN, Multiple Micronutrient; MUAC, Mid-upper Arm Circumference; PLA, Participatory Learning and Action; Preterm, Preterm Birth (Gestational age <37 weeks); RNI, Reference Nutrient Intake; SGA, Small for gestational age; VHSND, Village Health Sanitation and Nutrition Day; WASH, Water, Sanitation and Hygiene; WAZ, Weight-for-age Z-score; WLRAZ, Weight to length ratio-for-age Z-scores.

### Micronutrient supplementation

ICMR 2000 tested daily multiple micronutrients (MMN) amongst women with previous neural tube defect births.^38^ Katz 2000 tested weekly low-dose vitamin A or trans-beta-carotene,^37^^,^^54^ Khambalia 2009 tested iron and folic acid (IFA) micronutrient powders (sprinkles),^45^ and D’Souza 2021 tested vitamin B12 (2 μg) with or without MMN and milk powder.^42^^,^^46^

### Food supplementation

Hambidge 2019a tested daily Lipid-based Nutrient Supplements (LNS)^40^ and Potdar 2014 a locally manufactured snack containing green leafy vegetables, milk, and/or dried fruit.^39^

### Complex interventions

Kumar 2023,^44^ Doke 2024,^36^ and Taneja 2022^41^ tested a combination of individual, household and health system level components.^36^^,^^41^^,^^44^ These included health and nutritional status (body mass index (BMI)/anaemia) screening^36^^,^^41^^,^^44^ with tailored food supplementation (eggs or milk for underweight and meals for severely underweight women),^41^ anaemia treatment,^41^ provision of IFA or MMN,^36^^,^^41^ psychosocial support,^41^ and WASH.^41^^,^^44^ Social and behaviour change components included counselling,^36^^,^^41^^,^^44^ films and SMS/voice messaging on mobile phones,^36^ participatory learning and action (PLA) problem-solving approach to catalyse community action involving federations of women’s Self-Help Groups, adolescent groups, and farmer groups^44^ and encouraging the community to avail government rations and health services, especially via Village Health Sanitation and Nutrition Days.^36^^,^^44^ The PLA social and behaviour change intervention aimed to improve dietary habits and health behaviours, psychological well-being, family planning as well as social issues, such as girls’ secondary education delaying the age at marriage.^44^

### Outcomes

Outcomes assessed included birth size including birth weight,^39^, ^40^, ^41^, ^46^^,^^49^ length,^40^^,^^41^^,^^46^^,^^49^ head circumference,^40^^,^^41^^,^^46^^,^^49^ *z* scores for length-for-age (LAZ), weight-for-age (WAZ), and weight-to-length ratio-for-age (WLRAZ) and their respective cut-offs below < −2 SD,^40^^,^^49^ LBW,^36^^,^^38^^,^^39^, ^40^, ^41^^,^^49^ SGA,^39^, ^40^, ^41^^,^^49^ newborn circumferences and skinfold thicknesses,^39^ foetal growth,^52^ gestational age at birth,^39^^,^^46^ preterm birth,^36^^,^^37^^,^^39^, ^40^, ^41^^,^^49^ micronutrients in cord blood,^46^ and Brain Derived Neurotrophic Factor at birth.^46^ Nutritional outcomes in pregnancy included mid-upper arm circumference,^44^ weight,^41^ haemoglobin,^41^^,^^45^^,^^46^ anaemia,^41^^,^^45^ gestational diabetes, fasting glucose, and insulin,^51^ urinary iodine and creatinine,^50^ iron-related^45^ and other micronutrient blood assays,^46^ and retinol.^37^

### Comparison groups

Control groups provided alternative supplements of iron and calcium,^38^ folic acid alone,^45^ placebo capsules,^37^^,^^46^ routine care,^36^^,^^40^^,^^41^ a snack with a low micronutrient content (mostly potato, tapioca, and onion without any green leafy vegetables, milk, or dried fruit),^39^ and health system strengthening with weekly IFA supplementation.^44^

### Intervention modalities

The intensity and delivery mechanism of interventions varied (Supplementary File S7). Seven of the nine interventions targeted both pregnancy and preconception, and two had at least one arm focused solely on preconception.^41^^,^^45^ Preconception was defined as 1^38^ to 36^46^ months before pregnancy. Exposure to interventions varied, with some starting up to 27 months before conception.^46^ In two studies designed provide preconception micronutrient exposure, some participants joined after conception due to late detection of pregnancy.^37^^,^^45^ Most interventions targeted married women/girls,^35^^,^^37^^,^^39^^,^^41^^,^^45^^,^^46^ irrespective of parity,^37^^,^^39^^,^^41^^,^^44^ who intended to have a child.^36^^,^^39^, ^40^, ^41^^,^^45^ Khambalia 2009’s intervention focused on married non-pregnant nulliparous women,^45^ D’Souza 2021 on a 1993 birth cohort,^46^ and Kumar 2023 targeted newlyweds.^35^ Participants’ age varied widely, from adolescents 16–18 years,^46^ young women aged 16–35 years^40^ or 18–30 years,^41^ to a broader reproductive age range, including under 40 years,^39^^,^^45^ 10–49 years,^44^ and 15–49 years.^36^ Most studies were conducted in disadvantaged populations, predominantly in rural^36^^,^^37^^,^^40^^,^^43^, ^47^, ^35^ (including tribal)^36^ and some urban/peri-urban settings,^38^^,^^39^^,^^41^ in low and middle-income neighbourhoods.^41^

Project employees delivered all interventions except in two studies.^36^^,^^44^ In Kumar 2023, community-based workers (‘Poshan Sakhis’ and ‘Krishi Mitras’) facilitated PLA meetings, conducted screenings, and provided targeted home visits.^35^^,^^44^ Doke 2024 leveraged existing government health workers (Auxiliary Nurse Midwives and Accredited Social Health Activists).^36^ Taneja 2022 engaged local women to deliver nutrition/health support, with study teams managing measurements and referring as needed.^41^

Micronutrient and food supplementation studies enhanced compliance by observing consumption^37^^,^^39^^,^^41^ and/or counselling^40^^,^^46^ (Supplementary File S7). Compliance rates ranged from 57%^45^ to 89.6%.^49^ Compliance was 45% for Potdar 2014’s enhanced intervention snacks and 57% in control low-micronutrient snacks.^39^ Taneja 2022’s counselling reached 97% of participants and supplement consumption covered 75% of the follow-up period.^41^

### Alignment with frameworks on the causes of malnutrition and intervention pathways

**Fig. 2:**
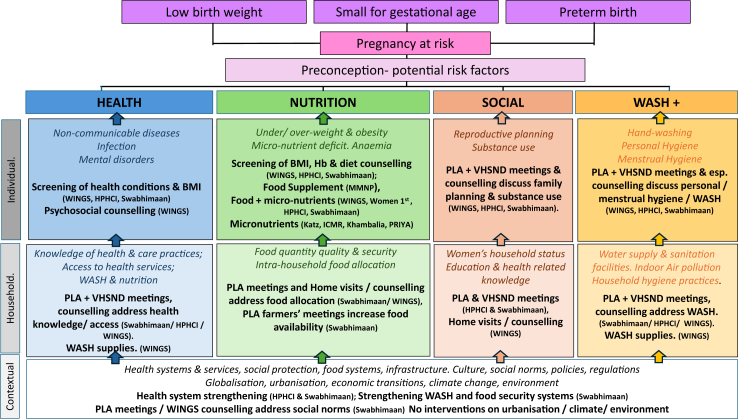
Where interventions in South Asia fit in the preconception conceptual framework (overall framework and text in italics taken from Partap 2022).

Mapping of determinants of preconception nutrition using the UNICEF 2021^33^ framework (Supplementary Files S4 and S7) and Partap’s conceptual framework^13^ (Fig. 2) showed that micronutrient^37^^,^^38^, ^45^, ^46^ and dietary supplement^39^^,^^40^^,^^48^, ^49^, ^50^, ^51^, ^52^ interventions work at an individual level to improve the immediate determinant diet. Complex interventions address health, nutrition, social and wash pathways both at individual and household levels.^41^^,^^44^ These interventions impact immediate determinants such as diet and care, underlying determinants like food, practices and services, and enabling determinants such as resources and norms. Kumar 2023’s PLA approach with Self-Help Groups federations influences community and contextual levels, addressing both underlying and enabling determinants.^44^ Further, building social and human capital through system strengthening initiatives influences services (underlying determinant) and governance, norms and resources (enabling determinants).^44^ Generally, most intervention efforts focused on improving individual level dietary intake in preconception, targeting the immediate determinants of nutrition as outlined in both Partap’s framework and the UNICEF framework. Only Kumar 2023 and Taneja 2022’s interventions attempted to address community, household and individual components simultaneously, and address a combination of health, nutrition, WASH and social drivers of poor preconception nutrition across the different levels of the UNICEF framework.^41^^,^^44^ The latter more complex multi-level interventions align closely with Partap’s pathways and might be expected to achieve stronger and more sustainable impacts by targeting multiple interconnected determinants.

### Effects of preconception nutrition interventions

Table 3 provides the effect sizes of nutritional outcomes for each study. Outcomes reported in two or more studies include birth weight, birth length, head circumference, LBW and SGA infants, birth/gestational age at birth, haemoglobin levels, and anaemia. Effects of interventions on outcomes such as micronutrient levels in pregnancy and cord blood, pregnancy-related measurements, gestational diabetes, and foetal growth were each examined in only one study.

**Table 3:** Preconception nutrition interventions in South Asia and their impacts on nutrition outcomes.







Overall effects of interventions on outcomes are indicated by shading as follows: Green shaded–mostly significant positive effects; orange–mostly null effects; and blue–effects varying by outcome.

AC Abdominal circumference; ARR absolute risk reduction; BPD Biparietal Diameter; BMI Body Mass Index; BMIAZ, body mass index-for-age *Z* score; BDNF Brain Derived Neurotrophic Factor; CI Confidence Interval; CRL Crown-Rump length; DRC Democratic Republic of the Congo; FA folic acid; FL Femur Length; GAA gestational-age-adjusted; GDM Gestational Diabetes Mellitus; GWG gestational weight gain; Hb Haemoglobin; HCAZ head circumference-for-age *Z* score; HC head circumference; holo-TC holotranscobalamin; I/Cr iodine to creatinine ratios; IFA Iron and folic acid; ITT intention to treat; IQR Interquartile range; IRR incidence rate ratio; LAZ length-for-age *Z* score; LBW Low Birth Weight; LGA Large for Gestational Age; LNS Lipid nutrient supplement; MD Mean Difference; MUAC mid-upper arm circumference; MMN multiple micronutrients; OR odds ratio; Preterm: Preterm birth; RR relative risk; Standard Deviation SD; SGA Small for Gestational Age; WAZ weight -for-age *Z* score; WHO World Health Organisation; WLZ length-for-age *Z* score; WLRAZ weight-length ratio-for-age *Z* score.

^a^Taneja 2022 intervention: A Complex intervention combining i) Health: screening/treatment, ii) Nutrition: screening/treatment of malnutrition and anaemia: iron folic acid or multiple micronutrients, locally prepared snacks, egg or milk, iii) Psychosocial support, iv) Water, Sanitation and Hygiene promotion.

^b^Kumar 2023 intervention: Complex intervention using Community-led Participatory Learning Action meetings with Self Help Groups, village organisations, and community nutrition microplanning. Combined 14 nutrition-specific and nutrition-sensitive interventions in the domains of access to government rations and iron folic acid, uptake of health services, Water, Sanitation and Hygiene, Dietary change and Nutrition-Sensitive Social and Behaviour Change.

^c^Doke 2024 intervention: Health screening/treatment; Anaemia prevention/treatment with IFA; Family planning services; Multi-channel Behaviour Change Communication at VHSNDs and home; Tailored counselling for BMI optimisation and Nutrition-sensitive counselling.

### Multiple micronutrient supplementation impact

The effects of preconception micronutrient supplementation on pregnancy-related outcomes were modest. Katz 2000’s vitamin A and beta-carotene supplementation during preconception and early pregnancy improved maternal serum retinol and reduced retinol deficiency at <4 months gestation.^37^ D’Souza’s preconception vitamin B12 and B12 + MMN significantly increased B12 (at 28 weeks’ gestation) and holo-TC (at 28 weeks’ and in cord blood) and B12 + MMN improved folate and B2 at 28 weeks.^46^ There was no effect on haemoglobin or anaemia at birth of Khambalia 2009’s preconception IFA powder (‘sprinkles’)^45^ or B12 or B12 +MMN supplements^46^ or of ICMR 2000’s MMN intervention on birthweight.^38^

### Food supplementation impact

In Hambidge 2019’s multi-country trial, combined preconception and pregnancy LNS improved some measures of newborn size at birth but effects varied between countries and outcomes.^40^ Dhaded 2020’s analysis of the effects of this intervention in India and Pakistan showed consistent positive impacts on newborn size of combined preconception and pregnancy LNS vs control, but not on preterm birth.^49^ Comparing preconception and pregnancy LNS with pregnancy only indicated reduced risk of SGA, WAZ < −1 and WLRAZ < −1 for preconception supplementation.^49^ Higher 12-week iodine status across the four countries was nominally associated with birth length-for-age (LAZ) and head circumference for age z-score (HCAZ), but not with other newborn anthropometry.^50^

Potdar 2014’s local snack enriched with green leafy vegetables, milk and dried fruit did not improve birth anthropometry in Intention to Treat (ITT) analyses,^39^ or foetal size or growth.^52^ However, a per protocol analysis amongst those who had ≥90 days of preconceptual supplementation showed a 48 g increase in birthweight in the enhanced snack arm.^39^ Second trimester foetal biparietal diameter and femur length were smaller among primiparous and larger among multiparous women in treatment arms vs control.^52^ The enhanced snacks reduced gestational diabetes mellitus (GDM) using ‘old’ WHO-1999 definitions, but no effects were found using WHO-2013 GDM criteria nor for fasting or 120-min glucose or insulin concentrations.^51^

### Complex preconception interventions impact

**Fig. 3:**
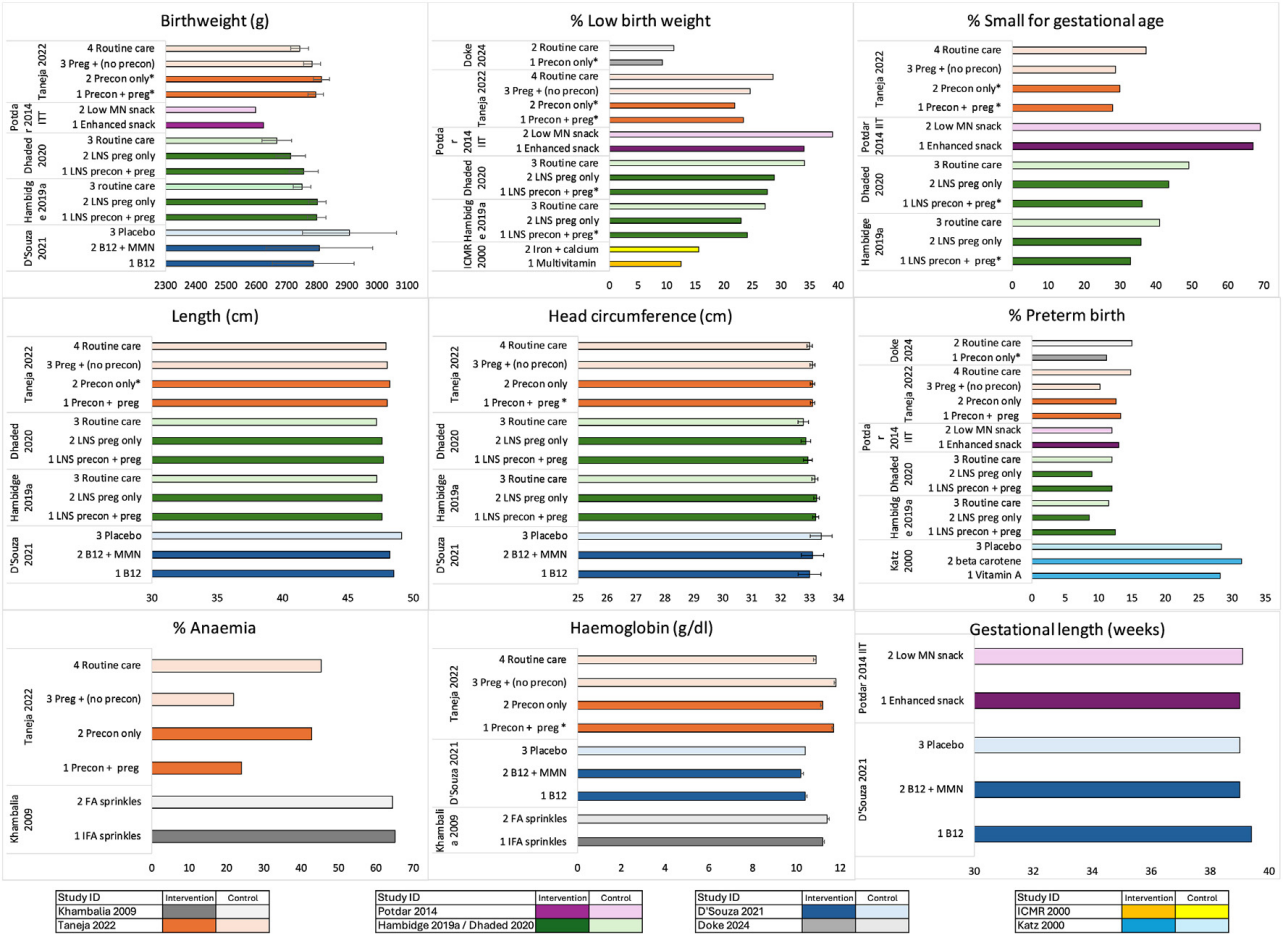
Nutritional outcomes by study arm across studies.

In Taneja 2022, preconception interventions (Arms 1 + 2) resulted in higher haemoglobin, higher gestational weight gain, and reduced underweight during late pregnancy, higher birthweight and length, and lower rates of LBW, SBA and stunting compared to no intervention (Arms 3 + 4), although head circumference showed no difference.^41^ Birthweight and length were higher in the preconception only arm than the pregnancy and preconception arm (Fig. 3).^41^ In Kumar 2023, there was no significant difference in difference between arms in mid-upper arm circumference <23 cm during pregnancy.^44^ Doke 2024 reported a higher adjusted prevalence ratio (PR) 1.3 (95% CI 1.1–1.6) for preterm birth in the comparison arm compared to the intervention.^36^

### Synthesis of effects

**Fig. 4:**
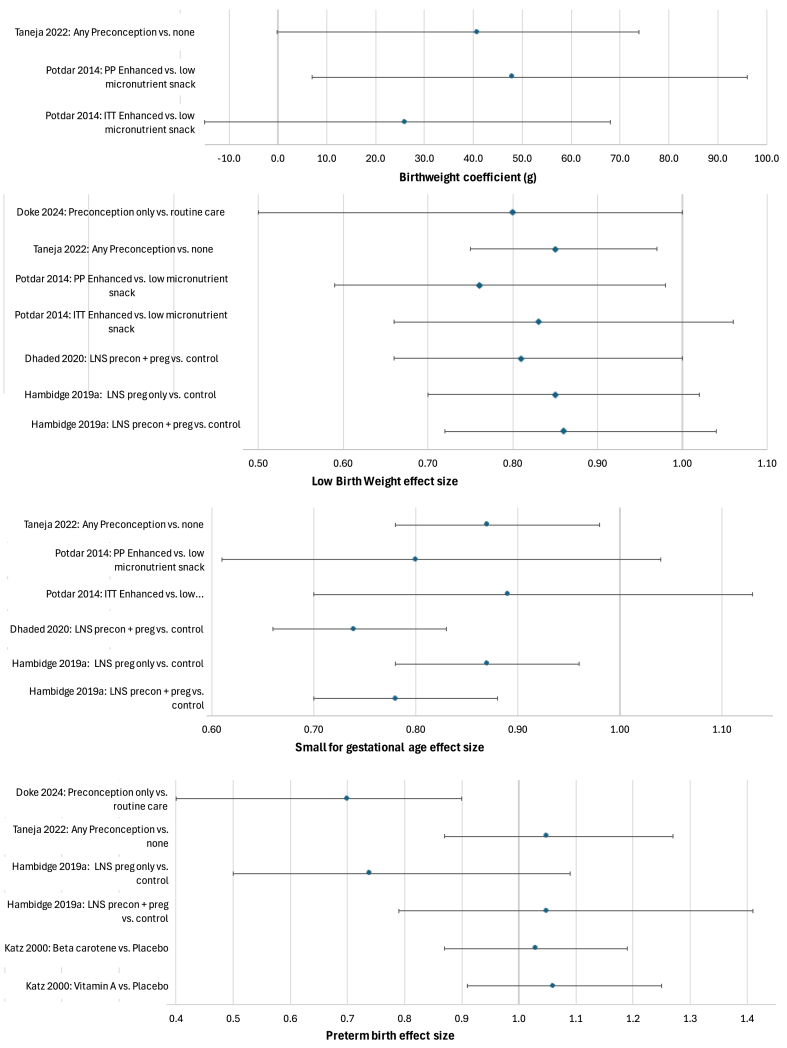
Coefficient plots illustrating effect sizes from various analyses across and within studies on preconception nutrition interventions.

Graphical syntheses of findings on outcomes which were reported in at least two studies are provided in Fig. 3, Fig. 4. Birthweight,^39^, ^40^, ^41^, ^46^^,^^49^ birth length and head circumference^40^^,^^41^^,^^49^ were higher in intervention arms than in control in most studies, apart from D’Souza 2021’s vitamin B12 supplementation intervention which had small sample size and no food supplementation.^46^ The coefficient plot in Fig. 4 summarises the estimates of the effect size for all the analyses using birthweight, SGA, LBW and preterm. Prevalence of LBW and SGA was higher in the control arm in all studies, but significant intervention effects were found more often on SGA than LBW. Potdar 2014’s per-protocol (≥90 preconception exposure) analysis showed that the enhanced snack arm had lower LBW (but not SGA). However, in the intention-to-treat analyses the effect was not significant.^39^ Dhaded 2020’s South Asian only analyses of combined pregnancy and preconception LNS vs control showed stronger effects on birthweight, LBW and SGA^49^ than Hambidge 2019’s main trial analyses which covered 4-countries.^40^ In Fig. 4, the effect of the LNS in pregnancy only vs control is shown for comparison; the effect upon SGA of preconception + pregnancy LNS vs control was greater than the effect of pregnancy only LNS, but this was not found with LBW.^40^

Most preconception interventions showed a lack of impact on preterm birth except for Doke 2024, which found lower preterm in the intervention arm.^36^ Generally, effects of preconception nutrition interventions upon anaemia or haemoglobin were null with the exception of Taneja 2022, whose intervention increased haemoglobin and decreased moderate-severe anaemia in pregnancy^41^ (Further description of intervention impacts is given in Supplementary file S8.)

### Intervention timing

We found substantial variation in the timing of interventions across studies, particularly in relation to the duration of preconception exposures and the combination of preconception and pregnancy interventions, which made it difficult to make direct comparisons between studies.

### Mechanisms of action

No process evaluations were reported across the 9 interventions in our review, providing scant evidence of changes along pathways to impact. Taneja 2022 was designed to improve multifactorial pathways across nutrition, health, social and WASH domains,^41^ informed by the World Health Organization framework on childhood stunting.^55^ The intervention in preconception and pregnancy increased gestational weight gain and haemoglobin in pregnancy, which later translated to improved birth size.^41^ The authors stressed that only a complex intervention concurrently addressing health, nutrition, psychosocial, and environmental issues can tackle a multifactorial problem like LBW or stunting.^41^ Preconception improvements in anaemia, nutritional status, and reproductive tract infections may have increased fertility, while counselling may have improved mental well-being and pregnancy planning.^41^

Hambidge 2019 found larger effects of the preconception fortified Lipid Nutrient Supplement on birth size in nulliparous compared with parous women and that an interaction between the treatment arm and anaemia suggested that anaemia reduction may be on the impact pathway.^48^ In this trial, mothers in the intervention arm had higher iodine to creatinine ratios at 12 weeks’ gestation (but not 3rd trimester). These were associated with higher birth length and head circumference, suggesting that supporting iodine metabolism through supplementation in preconception and early pregnancy is important to prevent iodine deficiency from limiting foetal growth.^50^ Supplementary Files S7 and S9 further summarise potential mechanisms of action discussed by the authors.

## Discussion

Preconception nutrition intervention trials in South Asia have used micronutrient supplementation (vitamin A/beta carotene, multivitamin, iron, and B12), food supplementation (LNS or a micronutrient-rich snack containing green leafy vegetables, milk and dried fruit), and complex interventions that address health, nutrition, social and WASH pathways to improved pregnancy and birth outcome. The evidence is heterogeneous with studies reporting varying effects on pregnancy related outcomes (such as micronutrients levels, gestational diabetes mellitus, gestational weight gain, and foetal growth) and birth outcomes (such as birth size, pre-term birth, and micronutrient level in cord blood). Preconception micronutrient supplements alone had limited success at improving micronutrient levels in pregnancy and cord blood, but did not significantly impact birth size or preterm birth. In contrast, preconception food supplementation, with or without multiple micronutrients and other complex intervention components, showed improvements in birth size, especially when initiated at least 90 days before conception. The lack of similar effect measures and heterogeneity in intervention and comparison groups makes it difficult to draw comparison between studies and prevented meta-analysis.

A package of health, nutrition, psychosocial care, and WASH interventions delivered during preconception and pregnancy periods, reduced the risk of LBW by 24%, with more than half of this reduction attributed to preconception interventions.^41^ This success, and the comparatively large impact of LNS supplements in reducing SGA and LBW in South Asia^49^ compared to in DRC and Guatemala,^40^ demonstrated that preconception interventions are at least as important as those delivered during pregnancy in improving birth outcomes in South Asia.^41^ However, several interventions^39^, ^40^, ^41^ were delivered through intensive use of project employees, which is challenging to scale up. Kumar 2023’s PLA approach increased the uptake of services,^44^ suggesting that integrating community mobilization approaches might further support social and behavioural changes. Two complex interventions which engaged with government systems^36^^,^^44^ showed promise and warrant further rigorous investigation.

Contrary to older reviews of protein-energy supplementation in pregnancy,^56^ the Potdar 2014 food supplementation intervention had a greater impact on the newborn size among mothers with higher BMI, primarily by increasing “soft tissue” rather than “bone” mass.^39^ This finding aligns with 11 of 12 pregnancy MMN supplementation trials, which observed a stronger effect on birth-weight in mothers with higher BMI.^57^ Nutrient metabolism, foetal supply line development, trans-placental transportation of nutrients, and foetal growth require energy and other substrates. These may be inadequate in underweight mothers, who may need extra macronutrients to effectively utilize the nutrients provided by supplements or to ensure nutrient partitioning to the foetus.^39^

Epigenetics studies suggest that preconception supplements may have anti-inflammatory and antioxidant properties that improve the maternal metabolic profile and change foetal DNA methylation, leading to better placental development, enhanced foetal organogenesis, increased birth size and improved long-term health.^40^^,^^43^^,^^58^ The EMPHASIS study found changes in DNA methylation in 7–9-year-old children, whose mothers had MMN supplementation before and during pregnancy.^59^ Preconception LNS activated placental protein or mRNA expression in Pakistani pregnant women and was associated with improved foetal growth compared with control.^60^

Despite some promising findings, gaps in evidence remain. Although the results of Taneja 2022^41^ and Dhaded 2020^49^ highlight the potential of preconception interventions, more RCTs with robust and thorough process evaluations are needed in South Asia. Studies need to identify the most effective intervention settings for delivering preconception care, whether in schools, community centres, or clinics to all adolescent girls, women and couples of reproductive age.^17^^,^^61^ Process evaluations must capture intervention delivery processes, the effect of context, and mechanisms of impact.^62^ Follow-up studies of South Asian cohorts^39^^,^^41^^,^^46^^,^^49^ are needed along the lines of PRECONCEPT. This Vietnamese trial tested the impact of preconception micronutrients in mothers and children,^34^ exploring long-term effects on adiposity,^63^^,^^64^ child stunting/wasting,^16^^,^^65^ anaemia, cognition^66^ and mental health.^14^ Although in Vietnam both MMN and IFA increased prenatal and postnatal ferritin levels, and infants had greater iron stores,^15^ Khambalia,^45^ ICMR,^38^ and others,^17^^,^^19^^,^^26^ found inconclusive evidence to support IFA supplementation during the preconception period. More research on the aetiology and prevention of anaemia and studies on the epigenetic effects of preconception nutrition interventions and their long-term impacts are warranted.

Limitations of the studies in this review included a lack of trial registration, only four interventions had peer-reviewed published protocols^35^, ^42^, ^43^, ^47^ and most studies did not provide a statistical analysis plan. Supplementary Files S10–S13 provide the risk of bias analyses (RoB) and tools. RoB for one cRCT^37^ and RoB2 for five reports from two parallel RCTs scored ‘low risk’^40^^,^^41^^,^^48^, ^49^, ^50^ and ‘some concerns’ for six RCT reports^38^, ^39^, ^45^, ^46^^,^^51^^,^^52^ due to deviation from intended interventions and missing outcome data.^39^^,^^51^^,^^52^ One RCT scored ‘some concerns’ because no pre-specified analysis plan was mentioned.^38^ There were minor deviations from intervention protocol^39^ and the COVID-19 pandemic disrupted studies.^36^^,^^46^ Two non-randomised trials scored ‘moderate’^44^ and ‘serious’^36^ risk of bias, respectively using ROBINS-I. Lack of randomisation presents challenges of systematic differences between arms.^36^^,^^44^ Methodological and reporting issues included missing details on birthweight measurement, lack of outcome assessment surveys, and lack of sufficient adjustment for baseline imbalances between arms.^36^ The strengths of our review include the use of standard systematic review methodology, multiple database searches, two investigators screened papers, the use of a detailed data extraction sheet and standardised tools for risk of bias assessment. A limitation is that data extraction and risk of bias assessment were not done in duplicate. The high heterogeneity in study designs, interventions, comparison groups and effect measures limits what interpretation can be made to inform intervention scale-up in programme settings. Meta-analysis could not be done due to heterogeneity of study design, interventions and outcomes. Overall grading of the evidence was not possible due to low number of studies reporting the same outcome or comparing similar study arms and the inability to conduct a meta-analysis due to different methods of estimating effect, which prevented the calculation of a pooled effect size.

Our study uniquely complements global reviews of preconception interventions by focusing on South Asia and systematically mapping interventions to established frameworks of pathways to impact. Previous reviews examined interventions such as delaying pregnancy,^17^ folic acid or IFA compared with MMN supplementation,^17^, ^18^, ^19^ or MMN compared with LNS supplementation.^19^ They found mixed evidence of impact on maternal anaemia and neural tube defects, and no clear impact on newborn outcomes. Partap 2022’s review of 58 nutrition, general health and reproductive planning interventions concluded that FA reduces neural tube defects, but evidence on what works to improve LBW, SGA and preterm was uncertain.^13^ These reviews included only two^18^ to seven^13^ studies from South Asia, and rarely considered complex interventions or explored intervention mechanisms. Despite data limitations, our review found that the effectiveness of preconception interventions differs in South Asia compared to other populations, as evidenced by the greater effects of preconception LNS supplementation in South Asia vs Africa and South America.^40^^,^^49^ This highlights the need for a regional approach to preconception care and further context specific research. Drivers of maternal and newborn malnutrition that are prevalent in South Asia include maternal undernutrition and micronutrient deficiency leading to low birth weight, poor maternal education, lack of sanitation,^67^^,^^68^ as well as poor diets, early marriage and maternal depression.^69^ Building on Partap’s conceptual framework of preconception risk factors,^13^ we examine which pathways were addressed by each South Asian preconception nutrition intervention, and where they lie within the UNICEF framework on the determinants of malnutrition. This unpicking of the potential mechanisms and pathways to impact provides unique insights on the importance of complex interventions that span multifactorial pathways. Our study resonates with Partap’s review findings^13^ in terms of the heterogeneity of intervention designs, the variability in the duration and design of preconception exposures and differing combinations of preconception and pregnancy interventions which makes collation of findings challenging. However, our study provides unique insights into specifically what works in South Asia, stressing the importance of at least 3 months preconceptual intervention exposure,^39^^,^^49^ the need for screening and targeting to tailor care to underweight, over-weight and anaemic women in the context of the triple burden of malnutrition,^36^^,^^41^^,^^44^ and the importance of integrating community mobilisation,^44^ psychosocial care and sanitation^41^ into intervention packages. The five food supplementation and complex intervention trials in this review collectively covered ∼35,000 preconception women, ∼11,500 pregnancies and ∼12,000 birth outcomes, but sample sizes in the two recent micronutrient supplementation trials were small (<300 each). Hence, the evidence is sufficient to support the WHO^12^ and UNICEF^70^ guidance on preconception education/counselling on healthy diets, IFA supplementation, screening and treatment of anaemia, monitoring of nutritional status and supplementing undernourished women/girls. However we found no evidence on the role of food fortification, promotion of exercise or screening and management of diabetes mellitus, which are also recommended.^70^ Our study calls for further South Asian studies, building on successes of food supplementation^49^ and complex interventions^41^ to undertake ‘real-world’ rigorous evaluations at scale within government systems. In addition, more conclusive evidence is needed on the impact of preconception micronutrient interventions on birth size, the role of food fortification and interventions to tackle diabetes.

## Conclusion

Our findings highlight that preconception interventions in South Asia combining micronutrient and food supplements with and without health screening, psychosocial counselling and WASH interventions, can reduce low birthweight and may improve other measures of newborn size at birth. However, this limited heterogeneous evidence calls for more complex intervention trials and implementation science research to evaluate sustainable and scalable preconception nutrition interventions in ‘real world’ programme settings engaging government workers and/or community volunteers in existing government intervention platforms wherever possible. Rigorous, well-structured, longitudinal studies following standardised study designs and data reporting to improve comparability and facilitate more precise interpretations are needed to better inform evidence-based preconception policies and interventions tailored to South Asian populations.

## Contributors

Conceptualisation: VS, NS and FM.

Supervision and resources: VS and ZM.

Project Administration: NS, FM and VS.

Methodology: NS, FM and VS. FM registered the protocol with PROSPERO.

Investigation: FM conducted the database and literature searches. NS and FM screened references at title/abstract and full text stages.

Data Curation: NS designed the data extraction table, extracted most of the data, and compiled the results. SD assisted with data extraction and summarised data in the manuscript tables.

Formal analysis: NS.

Visualisation: NS, SD and FM prepared and revised figures and graphs.

Validation: SD and NS undertook risk of bias assessment.

Writing—original draft: NS wrote the first draft and prepared the final draft for submission.

Writing—review & editing: All authors (NS, SD, FM, DS, RC, AH, JH, ZM and VS) read and commented on various drafts and approved the final manuscript.

## Availability of data, code and other materials

Data from the studies included in this review were not accessed other than to compile estimates from tables in published papers, so requests for data from the 14 studies included in this review should be directed to the authors of the original papers.

## Declaration of interests

VS and ZM are employed by UNICEF Regional Office for South Asia (ROSA) which funded this study. Other than this, the authors declare no conflicts of interest. The primary funders, UNICEF Regional Office of South Asia were involved in the design of the review, provided oversight in its conduct and VS and ZM are authors. Authors were not precluded from accessing data in the study, and they accept responsibility to submit for publication.

## Appendix A Supplementary data

Supplementary Files S1–S14

CRD42023398938_prospero_record_intervention_review
