# Supplementary material for: Effects of preconception nutrition interventions on pregnancy and birth outcomes in South Asia: a systematic review
Source: Lancet Reg Health Southeast Asia. 2025 Apr 24;36:100580. doi: 10.1016/j.lansea.2025.100580 (PMC12105516; doi:10.1016/j.lansea.2025.100580)
Supplement: Supplementary Files S1–S14 [file mmc1.docx]

**Supplementary material for review entitled Regional systematic review of preconception nutrition interventions in South Asia**

Table of contents

[1. Supplementary File S1. Preferred Reporting Items for Systematic Reviews and Meta-Analyses (PRISMA) checklist 2](#_Toc194327335)

[2. Supplementary File S2. Complete search strategies across all databases 5](#_Toc194327336)

[3. Supplementary File S3. Details of methods including data extraction form domains 8](#_Toc194327337)

[4. Supplementary File S4. UNICEF Conceptual framework 10](#_Toc194327338)

[5. Supplementary File S5. Partap 2023 Conceptual Framework 11](#_Toc194327339)

[6. Supplementary File S6. Data extraction spreadsheet 12](#_Toc194327340)

[7. Supplementary File S7. Intervention characteristics, compliance and mechanisms of preconception interventions 125](#_Toc194327341)

[8. Supplementary file S8. Narrative summary of Results of individual studies 135](#_Toc194327342)

[9. Supplementary File S9. Mechanisms of action or mediating factors discussed by authors 139](#_Toc194327343)

[10. Supplementary file S10. Visual presentation of risk of bias judgement of randomised controlled trials using the RoB2 tool 142](#_Toc194327344)

[11. Supplementary file S11. Visual presentation of risk of bias judgement of non-randomised trials using the ROBINS-I tool 143](#_Toc194327345)

[12. Supplementary file S12. The Risk Of Bias In Non-randomized Studies – of Interventions (ROBINS-I) assessment tool filled for Kumar A, Sethi V, Wagt A, et al. 144](#_Toc194327346)

[13. Supplementary file S13. The Risk Of Bias In Non-randomized Studies – of Interventions (ROBINS-I) assessment tool filled for Doke 2024. 155](#_Toc194327347)

[14. Supplementary File S14. References cited in supplementary material 168](#_Toc194327348)

#

# **Supplementary File S1. Preferred Reporting Items for Systematic Reviews and Meta-Analyses (PRISMA) checklist**


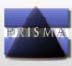
**PRISMA 2020 Checklist**

| **Section and Topic** | **Item #** | **Checklist item** | **Location where item is reported** |
| --- | --- | --- | --- |
| **TITLE Regional systematic review of interventions addressing preconception nutrition in South Asia** | | |  |
| Title | 1 | Identify the report as a systematic review. | Pages 1 and 4, abstract, |
| **ABSTRACT** | | |  |
| Abstract | 2 | See the PRISMA 2020 for Abstracts checklist. | NA |
| **INTRODUCTION** | | |  |
| Rationale | 3 | Describe the rationale for the review in the context of existing knowledge. | Page 3 |
| Objectives | 4 | Provide an explicit statement of the objective(s) or question(s) the review addresses. | Pages 3 and 4 |
| **METHODS** | | |  |
| Eligibility criteria | 5 | Specify the inclusion and exclusion criteria for the review and how studies were grouped for the syntheses. | Table 1 |
| Information sources | 6 | Specify all databases, registers, websites, organisations, reference lists and other sources searched or consulted to identify studies. Specify the date when each source was last searched or consulted. | Methods page 4, Supp file S2  Figure 1 |
| Search strategy | 7 | Present the full search strategies for all databases, registers and websites, including any filters and limits used. | Supp. File S2 |
| Selection process | 8 | Specify the methods used to decide whether a study met the inclusion criteria of the review, including how many reviewers screened each record and each report retrieved, whether they worked independently, and if applicable, details of automation tools used in the process. | Supp. File S3 |
| Data collection process | 9 | Specify the methods used to collect data from reports, including how many reviewers collected data from each report, whether they worked independently, any processes for obtaining or confirming data from study investigators, and if applicable, details of automation tools used in the process. | Methods page 4, Supp. Files S3 & S6 |
| Data items | 10a | List and define all outcomes for which data were sought. Specify whether all results that were compatible with each outcome domain in each study were sought (e.g. for all measures, time points, analyses), and if not, the methods used to decide which results to collect. | Pages 5-6,  Table 3 |
|  | 10b | List and define all other variables for which data were sought (e.g. participant and intervention characteristics, funding sources). Describe any assumptions made about any missing or unclear information. | Supp. File S3. Table 3. Methods page 4, |
| Study risk of bias assessment | 11 | Specify the methods used to assess risk of bias in the included studies, including details of the tool(s) used, how many reviewers assessed each study and whether they worked independently, and if applicable, details of automation tools used in the process. | Supp. File S3. Methods page 4 |
| Effect measures | 12 | Specify for each outcome the effect measure(s) (e.g. risk ratio, mean difference) used in the synthesis or presentation of results. | Table 3 |
| Synthesis methods | 13a | Describe the processes used to decide which studies were eligible for each synthesis (e.g. tabulating the study intervention characteristics and comparing against the planned groups for each synthesis (item #5)). | Supp. File S3. |
|  | 13b | Describe any methods required to prepare the data for presentation or synthesis, such as handling of missing summary statistics, or data conversions. | NA |
|  | 13c | Describe any methods used to tabulate or visually display results of individual studies and syntheses. | Supp. File S3. |
|  | 13d | Describe any methods used to synthesize results and provide a rationale for the choice(s). If meta-analysis was performed, describe the model(s), method(s) to identify the presence and extent of statistical heterogeneity, and software package(s) used. | Supp. File S3. |
|  | 13e | Describe any methods used to explore possible causes of heterogeneity among study results (e.g. subgroup analysis, meta-regression). | NA |
|  | 13f | Describe any sensitivity analyses conducted to assess robustness of the synthesized results. | NA |
| Reporting bias assessment | 14 | Describe any methods used to assess risk of bias due to missing results in a synthesis (arising from reporting biases). | NA |
| Certainty assessment | 15 | Describe any methods used to assess certainty (or confidence) in the body of evidence for an outcome. | NA |
| **RESULTS** | | |  |
| Study selection | 16a | Describe the results of the search and selection process, from the number of records identified in the search to the number of studies included in the review, ideally using a flow diagram. | Page 4-5  Fig 1 |
|  | 16b | Cite studies that might appear to meet the inclusion criteria, but which were excluded, and explain why they were excluded. | NA |
| Study characteristics | 17 | Cite each included study and present its characteristics. | Page 4-5  Table 2  Table 3  Supp File S7 |
| Risk of bias in studies | 18 | Present assessments of risk of bias for each included study. | Page 12  Supp Files S10 to S13 |
| Results of individual studies | 19 | For all outcomes, present, for each study: (a) summary statistics for each group (where appropriate) and (b) an effect estimate and its precision (e.g. confidence/credible interval), ideally using structured tables or plots. | Table 3  Supp Files S8 |
| Results of syntheses | 20a | For each synthesis, briefly summarise the characteristics and risk of bias among contributing studies. | Pages 9 and 12  Figures 3 and 4  Supp Files S10 to S13 |
|  | 20b | Present results of all statistical syntheses conducted. If meta-analysis was done, present for each the summary estimate and its precision (e.g. confidence/credible interval) and measures of statistical heterogeneity. If comparing groups, describe the direction of the effect. | Figures 3 and 4  p7-8 |
|  | 20c | Present results of all investigations of possible causes of heterogeneity among study results. | NA |
|  | 20d | Present results of all sensitivity analyses conducted to assess the robustness of the synthesized results. | NA |
| Reporting biases | 21 | Present assessments of risk of bias due to missing results (arising from reporting biases) for each synthesis assessed. | NA |
| Certainty of evidence | 22 | Present assessments of certainty (or confidence) in the body of evidence for each outcome assessed. | NA |
| **DISCUSSION** | | |  |
| Discussion | 23a | Provide a general interpretation of the results in the context of other evidence. | Page 10-14 |
|  | 23b | Discuss any limitations of the evidence included in the review. | Page 12 |
|  | 23c | Discuss any limitations of the review processes used. | Page 12 |
|  | 23d | Discuss implications of the results for practice, policy, and future research. | Page 13-14 |
| **OTHER INFORMATION** | | |  |
| Registration and protocol | 24a | Provide registration information for the review, including register name and registration number, or state that the review was not registered. | Page 4 |
|  | 24b | Indicate where the review protocol can be accessed, or state that a protocol was not prepared. | Page 4 |
|  | 24c | Describe and explain any amendments to information provided at registration or in the protocol. | NA |
| Support | 25 | Describe sources of financial or non-financial support for the review, and the role of the funders or sponsors in the review. | Page 15 |
| Competing interests | 26 | Declare any competing interests of review authors. | Page 16 |
| Availability of data, code and other materials | 27 | Report which of the following are publicly available and where they can be found: template data collection forms; data extracted from included studies; data used for all analyses; analytic code; any other materials used in the review | Supp. File S6 |

*From:*  Page MJ, McKenzie JE, Bossuyt PM, Boutron I, Hoffmann TC, Mulrow CD, et al. The PRISMA 2020 statement: an updated guideline for reporting systematic reviews. BMJ 2021;372:n71. doi: 10.1136/bmj.n71

#

# **Supplementary File S2. Complete search strategies across all databases**

**Ovid - MEDLINE, EMBASE, and Global Health**

[Search terms relating to preconception period, in addition to MeSH terms and/or database filters]:

preconcept* or pre*concept* or prepregnant* or pre*pregnan* or periconcept* or peri*concept* or "before conception" or "before pregnan*" or "newly* wed" or "newly married" or "pre*marriage" or "pre*marital" or (adolescen* AND (wom*n or girl* or female*)) or inter*preganan* or "between pregnan*" or "birth*interval" or "birth*spacing" or "pregnancy spacing" or "pregnancy interval" AND

[Search terms relating to nutrition status (exposure), in addition to MeSH terms and/or database filters]:

*nutrition* or nutrition* or diet* or under*weight or undernourish* or stunted or stunting or "short stature*" or obes* or over*weight or anthropometr* or "thin" or "thinness" or BMI or

"body*mass*index" or thin* or anemi* or anaemi* or weight or height or MUAC or

"mid*upper*arm*circumference" or haemoglobin or hemoglobin or hb or iron or IFA or ferritin or

"vitamin*a" or "vitamin*b12" or "vitamin d" or folate or "micro*nutrient" or zinc or micronutrien* AND

[Search terms relating to pregnancy and birth outcomes, in addition to MeSH terms and/or database filters]:

"gestational diabetes" or "gestational weight gain" or "gestational diabetes" or ((weight or stunted or stunting or "short stature*" or obes* or BMI or "body*mass*index" or thin* or weight or height or MUAC or "mid*upper*arm*circumference" or anemi* or anaemi* or haemoglobin or hemoglobin or hb or iron or ferritin or "vitamin*a" or "vitamin*b12" or "vitamin d" or folate or "micro*nutrient" or zinc or micronutrien*) AND pregnan*) or "low birth weight" or LBW or "fetal growth restric*" or FGR or "small for gestational age" or SGA or ((birth or 72 h* or 72h*) and ("weight*for*age" or "WAZ" or "height*for*age" or "HAZ" or "weight*for*height" or WHZ or weight or length)) or "intra*uterine growth" or IUGR or "pre*term birth" or PTB or "pre*term delivery" or still*birth or stillbirth or miscarriage

AND

[Search terms relating to South Asia, in addition to MeSH terms and/or database filters]:

"South Asia*" OR nepal* OR afghanistan* OR bhutan* OR india* OR maldiv* OR pakistan* OR "Sri Lanka*" OR Bangladesh*

AND

[Since 2000]

**Global Index Medicus (WHO)**

tw:(preconcept* or pre*concept* or prepregnant* or pre*pregnan* or periconcept* or

peri*concept* or "before conception" or "before pregnan*" or "newly* wed" or "newly married" or

"pre*marriage" or "pre*marital" or (adolescen* AND (wom*n or girl* or female*)) or inter*preganan* or "between pregnan*" or "birth*interval" or "birth*spacing" or "pregnancy spacing" or "pregnancy interval") AND tw:(*nutrition* or nutrition* or diet* or under*weight or undernourish* or stunted or stunting or "short stature*" or obes* or over*weight or anthropometr* or "thin" or "thinness" or BMI or "body*mass*index" or thin* or anemi* or anaemi* or weight or height or MUAC or "mid*upper*arm*circumference" or haemoglobin or hemoglobin or hb or iron or IFA or ferritin or "vitamin*a" or "vitamin*b12" or "vitamin d" or folate or "micro*nutrient" or zinc or micronutrien*) AND tw:("gestational diabetes" or "gestational weight gain" or "gestational diabetes" or ((weight or stunted or stunting or "short stature*" or obes* or BMI or "body*mass*index" or thin* or weight or height or MUAC or "mid*upper*arm*circumference" or anemi* or anaemi* or haemoglobin or hemoglobin or hb or iron or ferritin or "vitamin*a" or "vitamin*b12" or "vitamin d" or folate or "micro*nutrient" or zinc or micronutrien*) AND pregnan*) or "low birth weight" or LBW or "fetal growth restric*" or FGR or "small for gestational age" or SGA or ((birth or 72 h* or 72h*) and ("weight*for*age" or "WAZ" or "height*for*age" or "HAZ" or "weight*for*height" or WHZ or weight or length)) or "intra*uterine growth" or IUGR or "pre*term birth" or PTB or "pre*term delivery" or still*birth or stillbirth or miscarriage) AND tw:("South Asia*" OR nepal* OR afghanistan* OR bhutan* OR india* OR maldiv* OR pakistan* OR "Sri Lanka*" OR Bangladesh*)

AND

[Since 2000]

**Web of science**

All fields: [Search terms relating to preconception period, in addition to MeSH terms and/or database filters]:

preconcept* or pre*concept* or prepregnant* or pre*pregnan* or periconcept* or peri*concept* or "before conception" or "before pregnan*" or "newly* wed" or "newly married" or "pre*marriage" or "pre*marital" or (adolescen* AND (wom*n or girl* or female*)) or inter*preganan* or "between pregnan*" or "birth*interval" or "birth*spacing" or "pregnancy spacing" or "pregnancy interval" AND

All fields: [Search terms relating to nutrition status (exposure), in addition to MeSH terms and/or database filters]:

under*nutrition* or over*nutrition* or nutrition* or diet* or under*weight or undernourish* or stunted or stunting or "short stature*" or obes* or over*weight or anthropometr* or "thin" or "thinness" or BMI or "body*mass*index" or thin* or anemi* or anaemi* or weight or height or MUAC or "mid*upper*arm*circumference" or haemoglobin or hemoglobin or hb or iron or IFA or ferritin or "vitamin*a" or "vitamin*b12" or "vitamin d" or folate or "micro*nutrient" or zinc or micronutrien*

AND

Title, Abstract OR Key words (because too many search terms for all fields): [Search terms relating to pregnancy and birth outcomes, in addition to MeSH terms and/or database filters]:

"gestational diabetes" or "gestational weight gain" or "gestational diabetes" or ((weight or stunted or stunting or "short stature*" or obes* or BMI or "body*mass*index" or thin* or weight or height or MUAC or "mid*upper*arm*circumference" or anemi* or anaemi* or haemoglobin or hemoglobin or hb or iron or ferritin or "vitamin*a" or "vitamin*b12" or "vitamin d" or folate or "micro*nutrient" or zinc or micronutrien*) AND pregnan*) or "low birth weight" or LBW or "fetal growth restric*" or FGR or "small for gestational age" or SGA or ((birth or 72 h* or 72h*) and ("weight*for*age" or "WAZ" or "height*for*age" or "HAZ" or "weight*for*height" or WHZ or weight or length)) or "intra*uterine growth" or IUGR or "pre*term birth" or PTB or "pre*term delivery" or still*birth or stillbirth or miscarriage

AND

All fields:

"South Asia*" OR nepal* OR afghanistan* OR bhutan* OR india* OR maldiv* OR pakistan* OR "Sri Lanka*" OR Bangladesh*

AND

[Since 2000]

**Cochrane**

[Search terms relating to preconception period, in addition to MeSH terms and/or database filters]:

preconcept* or pre*concept* or prepregnant* or pre*pregnan* or periconcept* or peri*concept* or

"before conception" or "before pregnan*" or "newly* wed" or "newly married" or "pre*marriage" or

"pre NEXT marital" or (adolescen* AND (wom*n or girl* or female*)) or inter*preganan* or

"between pregnan*" or "birth NEXT interval" or "birth NEXT spacing" or "pregnancy spacing" or "pregnancy interval"

AND

[Search terms relating to nutrition status (exposure), in addition to MeSH terms and/or database filters]:

under*nutrition* or over*nutrition* or nutrition* or diet* or under*weight or undernourish* or stunted or stunting or "short stature*" or obes* or over*weight or anthropometr* or "thin" or "thinness" or BMI or "body NEXT mass NEXT index" or thin* or anemi* or anaemi* or weight or height or MUAC or "mid NEXT upper NEXT arm NEXT circumference" or haemoglobin or hemoglobin or hb or iron or IFA or ferritin or "vitamin NEXT a" or "vitamin NEXT b12" or "vitamin NEXT d" or folate or "micro NEXT nutrient" or zinc or micronutrien*

AND

[Search terms relating to pregnancy and birth outcomes, in addition to MeSH terms and/or database filters]:

"gestational diabetes" or "gestational weight gain" or "gestational diabetes" or ((weight or stunted or stunting or "short stature*" or obes* or BMI or "body NEXT mass NEXT index" or thin* or weight or height or MUAC or "mid NEXT upper NEXT arm NEXT circumference" or anemi* or anaemi* or haemoglobin or hemoglobin or hb or iron or ferritin or "vitamin NEXT a" or "vitamin NEXT b12" or "vitamin NEXT d" or folate or "micro NEXT nutrient" or zinc or micronutrien*) AND pregnan*) or "low birth weight" or LBW or "fetal NEXT growth NEXT restric*" or FGR or "small for gestational age" or SGA or ((birth or 72 h* or 72h*) and ("weight NEXT for NEXT age" or "WAZ" or "height NEXT for NEXT age" or "HAZ" or "weight NEXT for NEXT height" or WHZ or weight or length)) or "intra NEXT uterine growth" or IUGR or "pre NEXT term birth" or PTB or "pre NEXT term delivery" or still*birth or stillbirth or miscarriage

AND

"South NEXT Asia*" OR nepal* OR afghanistan* OR bhutan* OR india* OR maldiv* OR pakistan* OR "Sri NEXT Lanka*" OR Bangladesh*

AND

[Since 2000]

# **Supplementary File S3. Details of methods including data extraction form domains**

**Article screening and selection**

Two authors (FM and NS) independently screened all titles, abstracts, and full texts for relevant publications using Covidence software (Melbourne, Australia)(30) according to the criteria in Table 1. We resolved disagreements by discussion. For all included publications, we reviewed published protocols and searched for publications reporting sub-studies or secondary analyses. VS added two additional studies from her knowledge of preconception nutrition research in India.

**Risk of bias and quality assessment**

Two authors (NS and SD) assessed the risk of bias in each of the completed trials using the Cochrane risk of bias tool for parallel and cluster-randomised trials (RoB 2)(31, 32) or the Risk Of Bias In Non-randomised Studies of Interventions (ROBINS-I) assessment tool for non-randomised studies.(33) Disagreements were resolved through discussion.

**Data extraction and synthesis**

For each paper, one author (NS or SD) extracted available data on study characteristics and findings (details in Supplementary File S3). We grouped studies by type of preconception nutrition intervention (micronutrient supplementation, food supplementation, or complex intervention) and type of outcome (pregnancy, birth, anthropometric and blood assay), and tabulated key information for each study. We assessed heterogeneity in outcomes and analytical methods to scope meta-analysis of findings; however, no studies were found with comparable effect measures. When two or more studies reported on the same pregnancy or birth outcome, findings were plotted to compare effect estimates across studies.

**Domains of data included in the data extraction form**

i)        Study details, including author, year, setting, dates of data collection, study objectives, trial registration, protocol citation, and trial name or acronym

ii)       Details of study design, including sample size / power calculations, randomisation, blinding, how pregnancies were identified, follow-up schedule for measurements, adverse event and concomitant care reporting, compliance measurement and analyses approach

iii)     Characteristics of participants including, age, inclusion/exclusion criteria, and summary of baseline comparisons between arms

iv)     Details of intervention, including

·       timing (number of months or days of preconception exposure),

·       characteristics of intervention(s) delivered in preconception including exposures relating to preconception nutrition and during pregnancy in control and intervention arms,

·       delivery system (project team, health-, food- or social protection- systems),

·       engagement category (nutrition sensitive/specific and place in UNICEF framework ^23^), proposed mechanism of action provided by authors) and other (where relevant)

v)       Outcomes relating to nutritional status of the neonate at birth including but not limited to

·       LBW, Birth weight, SGA, Preterm

·       Other indicators of child nutritional status including but not limited to: Weight-for-age z score (WAZ), Underweight, Length-for-age z score (LAZ), Stunting, Weight-for-length z score (WLZ), BMI-for-age z score (BMIAZ), Wasting or Overweight.

vi)     Outcomes relating to nutritional status in mothers during preconception, pregnancy or at birth) including but not limited to:

·       Gestational weight gain, Gestational diabetes,

·       Maternal anthropometry (weight, MUAC, height),

·       Anaemia or other micronutrient deficiencies

vii)    Potential biological, behavioural or social mediating factors (as identified by authors of the primary studies)

viii)   Details on who interventions are available to, who accessed services, and how many accessed services

ix)     Author reflections on factors affecting the success/failure of preconception nutrition interventions, including factors relating to leadership, financing, governance, supplies, and capacity if provided

x)       Author recommendations on strategies to improve preconception nutrition in South Asia and on future research needed

# **Supplementary File S4. UNICEF Conceptual framework**

# **Supplementary File S5. Partap 2023 Conceptual Framework**

**
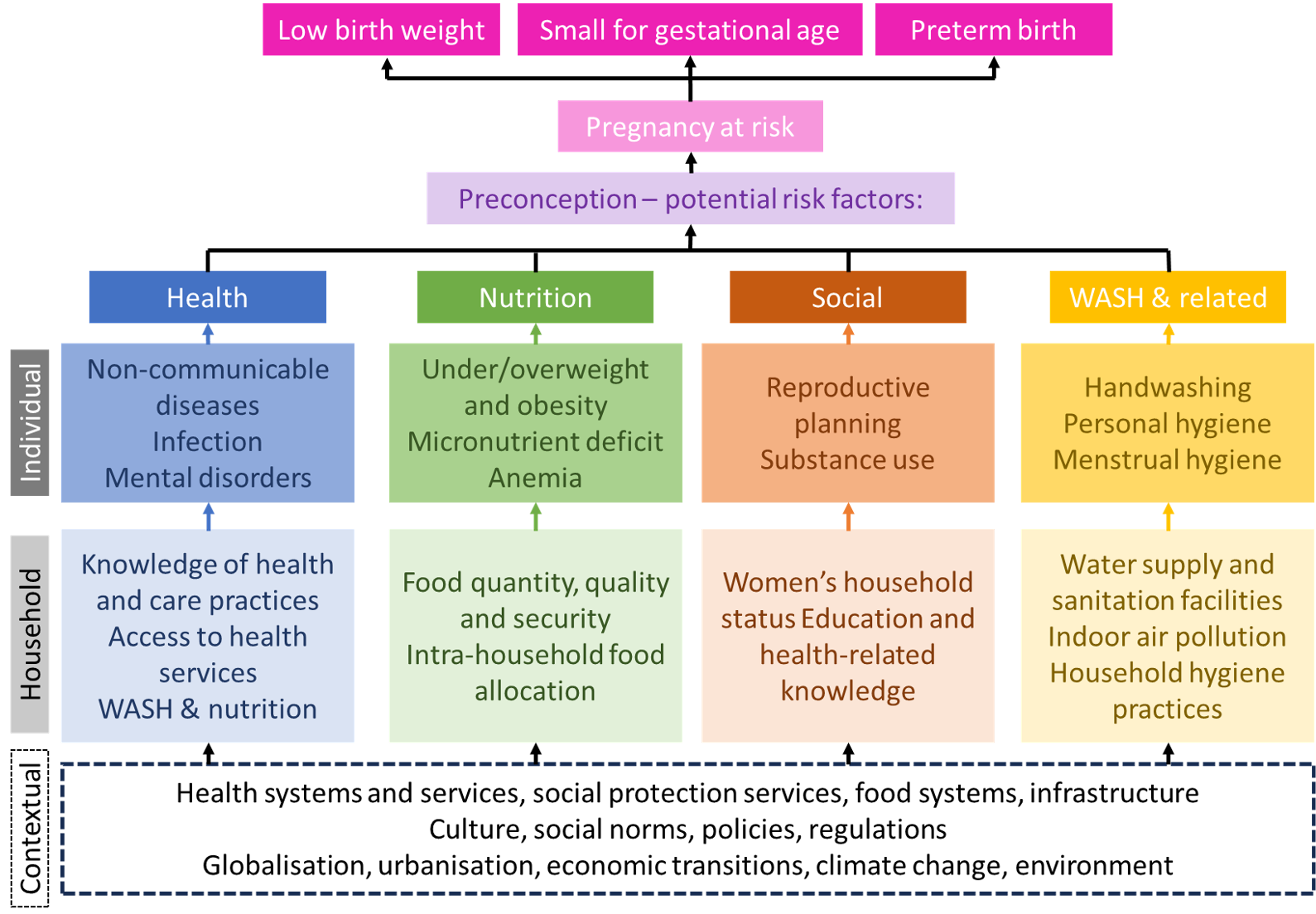
**

# **Supplementary File S6. Data extraction spreadsheet**

This table is in long format, the excel sheet is available from the corresponding author.

| **SN** | **1** | **2** | **3** |
| --- | --- | --- | --- |
| **Short name** | ICMR2000 | Katz2000 | West1999 |
| **Type of paper** | Trial findings | Trial findings | Trial findings |
| **Authors** |  | Joanne Katz, Keith P West Jr, Subarna K Khatry, Elizabeth K Pradhan, Steven C LeClerq, Parul Christian, Lee Shu-Fune Wu, Ramesh K Adhikari, Sharada R Shrestha, Alfred Sommer, and the NNIPS-2 Study Group | Keith P West Jr, Joanne Katz, Subarna K Khatry, Steven C LeClerq, Elizabeth K Pradhan, Sharada R Shrestha, Paul B Connor, Sanu M Dali, Parul Christian, Ram P Pokhrel, Alfred Sommer on behalf of the NNIPS-2 Study Group |
| **Year** | 2000 | 2000 | 1999 |
| **Title** | Multicentric study of efficacy of periconceptional folic acid containing vitamin supplementation in prevention of open neural tube defects from India | Maternal low-dose vitamin A or b-carotene supplementation has no effect on fetal loss and early infant mortality: a randomized cluster trial in Nepal | Double blind, cluster randomised trial of low dose supplementation with vitamin A or beta carotene on mortality related to pregnancy in Nepal |
| **Citation** | ICMR. (2000). "Multicentric study of efficacy of periconceptional folic acid containing vitamin supplementation in prevention of open neural tube defects from India. ." Indian J Med Res 112: 206–211. Jana, A., U. R. Saha, R. S. Reshmi and T. Muhammad (2023). "Relationship between low birth weight and infant mortality: evidence from National Family Health Survey 2019-21, India." Arch Public Health 81(1): 28. | Katz J, West Jr KP, Khatry SK, Pradhan EK, LeClerq SC, Christian P, Wu LS, Adhikari RK, Shrestha SR, Sommer A, NNIPS-2 Study Group. Maternal low-dose vitamin A or β-carotene supplementation has no effect on fetal loss and early infant mortality: a randomized cluster trial in Nepal. The American journal of clinical nutrition. 2000 Jun 1;71(6):1570-6. | West Jr KP, Katz J, Khatry SK, LeClerq SC, Pradhan EK, Shrestha SR, Connor PB, Dali SM, Christian P, Pokhrel RP, Sommer A. Double blind, cluster randomised trial of low dose supplementation with vitamin A or βcarotene on mortality related to pregnancy in Nepal. BMJ. 1999 Feb 27;318(7183):570-5. |
| **doi** |  | doi.org/10.1093/ajcn/71.6.1570 | 10.1136/bmj.318.7183.570 |
| **Clinical trials registration no** | NA | N/A | N/A |
| **Context / setting** | Bangalore, Mumbai, Lucknow, New Delhi, Pune, India  India: five centres viz, 1) Division of Human Genetics, Department of Anatomy, St John’s Medical College, Bangalore, 2) Department of Medical Genetics, Sanjay Gandhi Post Graduate Institute of Medical Sciences, Lucknow, 3) ICMR Genetics Research Centre, Mumbai; 4) Genetics Unit, Department of Pediatrics, All India Institute of Medical Sciences, New Delhi and 5) Department of Pediatrics, B.J. Medical College, Pune. | Sarlahi, Plains, Nepal | Sarlahi, Plains, Nepal |
| **Protocol citation** | None cited | No separate protocol. Protocol details referred to in West 1999 | None cited |
| **Arising from which main trial** | NA | No name given | No name given |
| **Study design** | Double blind, randomised, placebo-controlled trial with two arms | Double blind, cluster randomised, placebo controlled trial | Double blind, cluster randomised, placebo controlled trial |
| **Has Preconception intervention only arm (1/0)** |  | 0 | 0 |
| **Has Preconception + Pregnancy intervention arm (1/0)** | 1 | 1 | 1 |
| **Has Pregnancy intervention only arm (1/0)** |  | 0 | 0 |
| **Has Pregnancy, postpartum + childhood intervention arm (1/0)** |  | 0 | 0 |
| **Has Control arm with only usual standard of care or less intensive intervention in preconception & pregnancy (1/0)** |  | 1 | 1 |
| **MMN in Preconception arm** |  |  |  |
| **B12 in Preconception arm** |  |  |  |
| **LNS in Preconception arm** |  |  |  |
| **Viatmin A in preconception arm** |  | 1 | 1 |
| **Iron in preconception arm (both arms had Folic acid)** |  |  |  |
| **Nutritious snack in Preconception arm** |  |  |  |
| **Pregnancy outcomes** | 1 | 1 | 1 |
| **Birth outcomes** | 1 | 1 |  |
| **Infancy outcomes** |  | 1 |  |
| **Child outcomes** |  |  |  |
| **Narrative summary of main finding** |  | Small weekly doses of vitamin A or b-carotene given to women before conception, during pregnancy, and through 24 wk postpartum did not improve fetal or early infant survival in Nepal. | Small weekly doses of vitamin A or b-carotene given to women before conception, during pregnancy, and through 24 wk postpartum reduced pregnancy related mortality. This represented reductions of 40% (P < 0.04) and 49% (P < 0.01) among those who received vitamin A and beta carotene. |
| **Date of data collection** | 1988 to 1991 | Pregnancies reported from the last week of July 1994 onward and ended in an outcome before the first week of April 1997 were included in the analysis. | Eligible pregnancies for analysis were those ending from mid-July 1994 (by which time women had been routinely given supplements for >=5 months) and the end of June 1997, which permitted 12 weeks of postpartum dosing and follow up. |
| **Study objectives** |  | To assess the effect of maternal vitamin A or b-carotene supplementation on fetal loss and survival of infants <6 mo of age. | To assess the impact on mortality related to pregnancy of supplementing women of reproductive age each week with a recommended dietary allowance of vitamin A, either preformed or as Beta carotene |
| **Sample size / power** | The sample size was estimated for reduction by 80% in known risk of recurrence at α=0.05 and β=0.02. The minimum sample size required for each group was 197. Assuming that approximately 20% women may drop out or may not conceive during the trial period, it was planned to enroll a total of 500 women (250 in each group). | The trial required <10000 pregnancies in the 2 treatment groups combined and 5000 pregnancies in the placebo group to show a 20% reduction in fetal and infant mortality through 6 mo of age with 80% power and a type I error of 5%. | 14000 pregnancies (roughly 7000 in each supplemented group) and 7000 in placebo group, yielding an assignment ratio of 2 to 1, to show a >40% reduction in mortality related to pregnancy with >80% power (1 − beta) and 95% confidence (1 −alpha). Assumptions were based on mortality from pregnancy of > 600 deaths per 100 000 pregnancies in the study area. Smaller differences (>20%) in fetal and infant mortality up to 6 months of age would be discernible with the same sample size |
| **Sample characteristics** |  | 30 subdistrict areas (village development communities), each composed of 9 wards, were enrolled in the study. Within each subdistrict, each of the 9 wards were randomly assigned to receive 1 of the 3 treatments, resulting in 90 wards assigned to each treatment group. | 270 wards in 30 subdistricts (9 wards each) covering an area of around 500 sq km with a total population of around 176000 participated in the study. |
| **Randomisation** | Containers of vitamin or placebo capsules were given a random number and supply for one year was sent to the participating centres, where they were stored at a cool dry place. They key to random numbers was kept at the Central Tecnical Coordinating Unit at the ICMR Headquarters, New Delhi. | Within each subdistrict, each of the 9 wards were randomly assigned to receive 1 of the 3 treatments, resulting in 90 wards assigned to each treatment group. | All wards were assigned in Kathmandu by a random draw of numbered chits, blocked on subdistrict, for eligible women to receive one of three identical coded supplements. |
| **Blinding** |  | Participants and trial staff were blind to the allocation. Supplements were in opaque plastic bottles labeled with 1 of 3 masked, numeric codes. The bottles were relabeled with individual ward numbers that had been assigned to the specific codes. | Participants and trial staff were blinded to allocation. Doctors analysing verbal autopsies were blind to treatment allocation |
| **Age of participants (years)** | Mean age was 25.69 +/- 4.12 in vitamin group and 26.07 +/1 3.85 in placebo group. | Range not given but included women below 20 and above 30. Mostly 20-29y. | Range not given |
| **Inclusion criteria** | Women who had a previous pregnancy with Neural Tube Defects (NTD). | All married women of childbearing age were eligible for enrolment in the study (identified through a baseline census before dosing). | Women of childbearing age who were married and living with their husbands as of the first week of March 1994 were recruited to the trial after giving their verbal consent. Newly married women were recruited throughout the trial. |
| **Exclusion criteria** |  | Women who migrated into the study area were not enrolled | Women who were already married who had moved into study wards were not eligible to participate to minimise crossover. |
| **Preconception intervention characteristics** | Arm 1: Multivitamin supplement. Each capsule containing: ferrous sulphate 120 mg, calcium phosphate 240 mg, vitamin A 4000 IU, D 400 IU, BI 2.5 mg, B2 2.5 mg, B6 2 mg, nicotinamide 15 mg, vitamin C 40 mg, folic acid 4 mg and zinc 10 mg.  Women was provided with drug for three months at one time via participating centers. At next visit the women were advice to return the entire unused drug. The women were advised not to take any vitamin preparation during the trial period. | Wards were randomly assigned to have women receive weekly doses of 7000 𝜇g retinol equivalents as retinyl palmitate (vitamin A) or 42 mg all-trans-b-carotene in gelatin casules (with 5 mg dl-a-tocopherol which was also in placebo) | Opaque, gelatinous capsules containing peanut oil and 23 300 IU of preformed vitamin A (7000 𝜇g retinol equivalents) as retinyl palmitate or 42 mg of all trans beta carotene (7000 𝜇g retinol equivalents, assuming a conversion ratio to retinol of 6 to 1 after uptake). The dosage was intended to deliver an approximate recommended dietary allowance during pregnancy and lactation on a weekly basis. All capsules also contained about 5 mg dl-alpha-tocopherol as an antioxidant - including in placebo. |
| **Preconception control group intervention** | Arm 2: Placebo capsule containing ferrous sulphate 120 mg and calcium phosphate 240 mg was provided to women for three months at one time via participating centres. | Weekly doses of gelatin capsules of identical appearance containing peanut oil and 5 mg dl-a-tocopherol | Opaque, gelatinous capsules containing peanut oil and 5 mg dl-alpha-tocopherol as an antioxidant, without no vitamin A or beta carotene |
| **Intervention timing (how far before conception)** | Daily supplement provided at least 1 month before conception and 3 months after concpetion | Women generally had the opportunity to receive supplements for ≥20 wk before declaring themselves pregnant and provided enough time for 24 wk of postpartum follow-up before the trial was concluded.  Note that most women reported pregnancy at 4 months gestation so *may not have received the supplement before pregnancy*. | First pregnancies enolled > 5 months after start of intervention but timing of preconception supplementation not provided. |
| **Intervention delivery (health systems, food systems, social protection systems)** |  | Local women were hired and trained to visit the homes of all enrolled women to distribute the weekly supplements. The schedule of visits was such that there were always ≥ 4 d between each consecutive dose.  Not within the health system - trial had own delivery system. | From April 1994 to September 1997 432 local female workers carried out weekly home visits and dosed participating women with their assigned supplement. At least 4 days between doses were maintained to avoid any potential risk of toxicity from receiving supplements on two consecutive days.  Capsules were not left at homes. |
| **Pregnancy detection** |  | The 426 distributors of the supplements recorded pregnancy and vital status, menses in the past week, and receipt of the capsule. | Field workers who delivered the supplements recorded menstrual activity in the previous week, and pregnancy status as reported by women. |
| **Pregnancy intervention characteristics (if any)** |  | Continuation of the preconception supplement throughout pregnancy and into the postpartum period. | Weekly dose of opaque, gelatinous capsules containing peanut oil and 23 300 IU of preformed vitamin A (7000 𝜇g retinol equivalents) as retinyl palmitate or 42 mg of all trans beta carotene (7000 𝜇g retinol equivalents, assuming a conversion ratio to retinol of 6 to 1 after uptake) as given before pregnancy. 5 mg dl-a-tocopherol was in both intervention and placebo supplements. |
| **Pregnancy control group intervention (if any)** |  | Continuation of the preconception weekly doses of gelatin capsules peanut oil and 5 mg dl-a-tocopherol | Weekly dose of opaque, gelatinous capsules containing peanut oil with 5 mg dl-a-tocopherol. |
| **Interventions after birth (if any)** |  |  |  |
| **Control group intervention after birth (if any)** |  |  |  |
| **Compliance measurement** | Each women was called for a follow up every three months. At each visit compliance of drug intake was checked with the help of diary card maintained by the woman and number of capsules returned. If the total number of missed days in three months did not exceed 10 days and the total number of missed days at a stretch did not exceed three, the complance was taken satisfactory. Regular intake of drug for at least one month prior to the conception and up to three months after concepton was taken as full compliance. The woman was taken out of trial if she developed any side effect or did not conceive within one year of enrolment. | Receipt of the capsule was noted only if the distributor observed the woman swallowing the capsule.  Compliance with treatment was based on the proportion of eligible doses taken in each pregnancy from 1 mo before conception through 6 mo postpartum or until miscarriage, stillbirth, maternal death, or death of the infant. | authors checked compliance in each supplement group by examining the percentage of all eligible doses during the trial (or until death) taken by women and the differences in serum retinol and β carotene concentrations by code among pregnant women in the substudy sample. Half of the women who were ever pregnant and 44% of those who were never pregnant received >80% of their intended supplements. Over 75% of the pregnant women received at least half of their eligible doses—that is, more than half of a dietary allowance for those receiving vitamin A or beta carotene— compared with around 62% of those who were never pregnant. Compliance was about 3% lower in the beta carotene group in the mid-range of supplement intake. |
| **Adverse events** |  | Not mentioend but a Data Safety and Monitoring Committee met in November 1996 and in March 1997 and agreed to continue the study. This committee and the data analysts were unmasked to the treatment codes, but the codes were made available to study investigators only at the end of the trial. | Not mentioned |
| **Engagement, category (including nutrition sensitive/specific)** | Nutrition Specific | Nutrition specific | Nutrition specific |
| **Where in the overall UNICEF 2020 framework the intervention lies** | Intervention changing an the immediate determinant of Diet: Multivitamin supplement is directly altering diet. | Intervention changing an the immediate determinant of Diet: low dose Vitamin A supplement is directly altering altering diet | Intervention changing an the immediate determinant of Diet: low dose Vitamin A supplement is directly altering altering diet |
| **Where in the Partap 2021 framework the intervention lies** | Giving multivitamin capsules containing containing ferrous sulphate 120 mg, calcium phosphate 240 mg, vitamin A 4000 IU, D 400 IU, BI 2.5 mg, B2 2.5 mg, B6 2 mg, nicotinamide 15 mg, vitamin C 40 mg, folic acid 4 mg and zinc 10 mg is an individual-level nutrition intervention. | Vitamin A supplement is an individual-level nutrition intervention. | Vitamin A supplement is an individual-level nutrition intervention. |
| **Proposed mechanism of intervention action** |  | Not discussed |  |
| **Exposures relating to preconception nutrition** |  | Vitamin A or beta carotene supplements | None |
| **Preconception measures** |  | None | None |
| **Concommittant care** |  | Not mentioned | Not mentioned |
| **Follow-up schedule for measurements** |  | Pregnancies, miscarriages, stillbirths and livebirths were recorded weekly and were based on self-reports. Deaths of women were also noted at this time.  Pregnant women were interviewed at the time they declared themselves pregnant (at an average of 4 mo gestation) and 3 mo later (at an average of 7 mo gestation). These interviews included completion of a 7-d food frequency questionnaire, a pregnancy history, recording of socioeconomic status, measurement of midupper arm circumference, morbidity, and an activity history. In the event of a miscarriage or stillbirth, a trained interviewer visited the woman and recorded information concerning that event. Women who delivered a live infant were followed and an interview was scheduled at 3 and 6 mo postpartum to assess the health and survival of the infant and mother. | Field workers who delivered the supplements recorded the survival of the women, receipt of capsules, menstrual activity in the previous week, and pregnancy status as reported by women. They revisited the homes of women who were absent until they were able to give them the dose or until the last day of a dosing week.  Five months after supplementation and reporting were running smoothly, newly enrolled pregnant women entered followup that included a mid-pregnancy, home based, 7 day dietary, morbidity, and activity assessment and measurement of arm circumference by one of a trained team of about 30 interviewers. A second visit during the third trimester included socioeconomic evaluation. Seven months after the start of the study newly enrolled pregnant women from a subsample of three contiguous subdistricts (27 wards), were enrolled for additional measures that required blood collection and measurement of concentrations of retinol and beta carotene.  A verbal autopsy history of events and illnesses preceding death was obtained by interviewing family members of the dead woman, usually within one month after the death had been reported. |
| **Summary of baseline comparisons by study arm** | Mean age was 25.69 +/- 4.12 in vitamin group and 26.07 +/1 3.85 in placebo group. Mean parity was 1.96+/- 1.22 in vitamin group and 2.37+/-1.44 in placebo group. 91.18% women in vitamin group had one previous NTD child and 92.09% in placebo group. 10 families in the vitamin group (7.3%) an 13 in placebo group (9.15%) had the history of consanguinity, but the difference was not significant. | The pregnant women in the 3 treatment groups were not significantly different with respect to age, midupper arm circumference, dietary intake, parity, and socioeconomic status. | Comparability of randomised groups by socioeconomic and dietary characteristics of women during their first enrolled pregnancy was assessed by the χ2 test; differences in distributions of serum retinol and β carotene concentrations were tested by analysis of variance and comparing the two groups with the t test. At the time of their first study pregnancy, the three groups of women were comparable in age, arm circumference, and weekly dietary intakes. Small differences were evident with respect to cigarette smoking, alcohol consumption, and literacy. A smaller percent- age of the placebo group were of low Hindu caste or were not Hindus. Only 3% of pregnancies were delivered at a health post, clinic, or hospital. |
| **Pregnancy outcomes: gestational weight gain, gestational diabetes, maternal anthropometry (weight, MUAC, height), anaemia,** | live births, still births, spontaneous abortion, induced abortion, NTD, birth weight | Primary outcome: Fetal loss rate (any reported miscarriage, stillbirth, or materno-fetal death during pregnancy) /1000 pregnancies.  Retinol (mmol/L) & deficiency <70 mmol/L (%) at 4 months gestation. | Mid-pregnancy retinol and beta carotene concentrations |
| **Preconceptual outcomes: (BMI, Hb or other outcomes which could be attributed to preconception care)** |  |  |  |
| **Birth outcomes in child: birthweight, low birth weight (LBW), small for gestational age (SGA), preterm birth/ delivery (PTB), cord blood b12, DXA,** | Primary outcome: NTD,  Others: birth weight, | Prevalence of preterm birth (gestation <37 wk). However, iit is possible that gestational age was underestimated because it appeared that women often mistook vaginal bleeding during early pregnancy for menses. This underestimation might make it harder to observe a treatment effect on the rate of preterm birth if such an effect existed. | None |
| **Outcomes in mother at birth** |  | Maternal death up to 42 days (reported in West 1999) |  |
| **Post-natal outcomes (in Infancy)** |  | Serum all-trans-retinol concentrations in infants at 3 mo of age: all-trans-Retinol (mmol/L) & deficiency <70 mmol/L (%). Neonatal mortality rate. Mortality rate from 28 d to 24 wk per 1000 infants alive at 28 days. Cumulative 24-wk mortality rate per 1000 livebirths (second main outcome - not called primary).  Analysis stratified by compliance (0–25%, 25–50%, 50–75%, and 75–100%) showed that there was a larger (but not significant) difference in infant serum retinol between the vitamin A and placebo groups among women with high compliance. However, there was no difference in the survival of infants by level of compliance. Hence, compliance altered vitamin A status but not the health outcomes of the infants. | Pregnancy-related death up to 12 weeks. authors extended postpartum follow up from 6 to 12 weeks because maternal mortality related to malnutrition could extend beyond the conventional period of 6 weeks.  Maternal mortlaity ratio per 100000 livebirths from conception to 6 weeks postpartum. |
| **Outcomes in childhood after 12 m: z scores for weight (WAZ), length/height (HAZ), and BMI for age (BMIZ), weight-for-height'length (WHZ), underweight, stunting, wasting, overweight, cognitive** |  | None | None |
| **Long term outcomes** |  | None | None |
| **Analysis** | Information for each woman was collected on pre-designed proforma at all centres participating in the study. These forms were sent every month to the ICMR headquarters for data entry and regular onitoring of the progress of the trial.  Statistical comparisons between placebo and vitamin groups were made for various parameters under study. Student's t, Z and chi-square test used for testing the difference between means and proportions as applicable. One tailed P value <0.05 was considered significant for comparison of drug effect. Statistical software SPSS 9.0, Epi Info 6.0 and NCSS 6.0 were used for analysis. | Analysis done on intention-to-treat basis.  The proportion of pregnancies that resulted in fetal loss was compared across treatment groups by estimating relative risks and 95% CIs. Similarly, the 24-wk infant mortality rates (number of infants who died by 24 wk divided by the total number of live births for which vital status was known at 24 wk) were compared across treatment groups. In addition, neonatal mortality was calculated as the number of deaths of infants < 28 d of age/1000 live births. Deaths from 28 d to 24 wk were divided by the number of infants alive at 28 d for whom vital status was known at 24 wk.  Treatments were assigned to wards, not to individuals, with stratification by village development community (a subdivision of 9 wards each). The 95% CIs were adjusted for cluster design by using a generalized-estimating-equations logistic regression model with exchangeable correlation structure in which survival was modeled as a function of the treatment assignment, adjusted for the correlation within the units of ran- domization (the ward) and stratification (by village development community). If a woman became pregnant more than once during the study, each pregnancy was included in the analysis, as were both infants in twin pairs. An analysis with only the first pregnancy and first infant enrolled in the study produced the same relative risks with 5% wider 95% CIs. SAS (SAS Institute Inc, Cary, NC) was used for the analyses. | differences in distributions of serum retinol and β carotene concentrations were tested by analysis of variance and comparing the two groups with the t test. Ascertained pregnancies served as the denominator for rate estimation. Mortality was evaluated on an intention to treat basis—that is, by supplement assignment irrespective of compliance. Mortality related to pregnancy and specific causes for each group was calculated from deaths that occurred during pregnancy up to 12 weeks post partum and was expressed per 100 000 pregnancies. However, authors also examined impact on the maternal mortality ratio (for which authors excluded deaths due to reported injury and all deaths >6 weeks post partum) in relation to live births. Relative risks with 95% confidence intervals were calculated with the placebo group as the reference. Each confidence interval was adjusted to account for the fact that the ward rather than the person was the unit of randomisation. A quasi-likelihood Poisson regression model was used to estimate the degree of overdispersion in the ward specific death rates. This overdispersion, due to the design effect, of about 21% of the variance resulted in a 10% inflation in the length of a confidence interval which was applied to the natural logarithm of all estimates of relative risk. |
| **EFFECTS on Preconception outcomes: maternal anthropometry (weight, MUAC, height. BMI), Haemoglobin (Hb), anaemia, iodine status** | None |  |  |
| **EFFECTS on Pregnancy outcomes: gestational weigt gain, gestational diabetes, maternal anthropometry (weight, MUAC, height), anaemia, iodine status** | The occurrence of live birth was higher in vitamin group vs placebo: 125 (91.24%) in Vitamin group and 117 (82.39%) in placebo group, the difference was statistically significant.The occurence of still birth in two groups were similar : 3 (11.9%) in Vitamin group and 3 (2.11%) in placebo group  Spontaneous and induced abortion were higher in placebo group vs vitamin group. Spontaneous abortion: 6 (4.38%) in Vitamin group and 14 (9.86%) in placebo group Induced abortion: 3 (2.19%) in Vitamin group and 8 (5.63%) in placebo group | The prevalence of fetal loss (miscarriages, stillbirths, and materno-fetal deaths during pregnancy) was 92.0/1000 pregnancies in the placebo group, comparable with the rates in the vitamin A and b-carotene groups. The relative risks and 95% CIs indicated no effect of either supplement type on fetal loss.  In a subsample of 935 women, maternal serum retinol at <4 mo gestation was highest in the vitamin A group (1.30 ± 0.33 mmol/L), next highest in the b-carotene group (1.14 ± 0.39 mmol/L), and lowest in the placebo group (1.02 ± 0.35 mmol/L) (13). The means of all 3 groups were significantly different from each other (P < 0.0001). Only 2.9% of women in the vitamin A group (P < 0.0001) and 13.5% of those in the b-carotene group (P = 0.0001) had serum retinol concentrations <0.70 mmol/L; 19.3% of women in the placebo group had concentrations below this amount. | The mean serum retinol concentration was lowest in the placebo group (1.02 micromol/l), highest among vitamin A recipients (1.30 micromolmol/l), and between these two values in the beta carotene group (1.14 micromolmol/l).  The percentage of women by supplement group with serum retinol concentrations < 0.70 micromol/l followed the same pattern. The mean beta carotene concentration was significantly higher (0.20 micromolmol/l) and the percentage of women with concentrations < 0.09 micromol/l lower (26.5%) in the beta carotene than in the vitamin A and placebo groups (around 0.14 micromol/l and about 42% in both groups). The relative risk of mortality related to pregnancy was protective for both nutritional supplements during pregnancy but not significant. Mortality during pregnancy was 235, 142, and 111 maternal deaths per 100 000 pregnancies in the placebo, vitamin A, and beta carotene groups, yielding relative risks of 0.60 (0.26 to 1.38) (P = 0.2) and 0.47 (0.18 to 1.20) (P = 0.11) in the vitamin A and beta carotene groups, respectively. |
| **EFFECTS on Birth outcomes in child: birthweight, low birth weight (LBW), small for gestational age (SGA), preterm birth/ delivery (PTB)** | The recurrence of open NTD in the vitamin group was 2.92 per cent compared to 7.04 per cent in the placebo group, a reduction by about 60 per cent. The difference, however, was not statistically significant (P = 0.06).  In multivitamin group (Arm 1), 12.5% neonates had birth weight below 2.5 kg compared to 15.6% in the placebo group (Arm 2). The difference was not statistically significant. | There was no effect of supplementation on the prevalence of preterm birth (gestation <37 wk), which was 282, 314, and 284/1000 pregnancies in the placebo, vitamin A, and b-carotene groups, respectively. | NA |
| **EFFECTS on Outcomes in mother at birth** | None. | NA | Shown in postnatal as spans up to 12 weeks |
| **EFFECTS on Post-natal outcomes (in Infancy)** | None | There was no effect of supplementation on neonatal mortality (birth through 28 d), 28 d through 24 wk mortality, or cumulative 24 wk mortality. There was also no significant difference in the effect of treatment by sex or by whether the delivery occurred at the mother’s parental home or not. In a subsample of 704 of 1215 eligible infants (58%) for whom a heel-stick blood sample was obtained at 3 mo of age, 83% of the placebo group had concentrations <0.70 mmol/L, whereas 62% and 76% of those in the vitamin A and b-carotene groups, respectively, had concentrations below this amount. P < 0.0001 for all 3 groups and vit A vs placebo. p=0.07 & P = 0.03 for comparison of placebo and b-carotene continuous and binary outcome respectively. | Mortality related to pregnancy up to 12 weeks post partum was 704, 426, and 361 maternal deaths per 100 000 pregnancies in the placebo, vitamin A, and beta carotene groups, yielding relative risks of 0.60 (0.37 to 0.97) (P = 0.04) and 0.51 (0.30 to 0.86) (P = 0.01) in the vitamin A and beta carotene groups, respectively.  Mortality among women receiving beta carotene was not significantly different from that in the vitamin A group (relative risk 0.85 (0.48 to 1.49), P = 0.57). authors therefore combined the effects to obtain a relative risk of 0.56 (0.37 to 0.84), reflecting a 44% reduction in mortality related to pregnancy associated with vitamin A or beta carotene supplementation (P = 0.005).  The relative risk was protective for both nutritional supplements during pregnancy, from the end of pregnancy to 6 weeks post partum, and from 6 to 12 weeks post par- tum. Analysis of cause specific mortality, showed protective but non-significant effects of supplementation against risk of death from obstetric causes and infection. Point estimates of relative risk are stronger for beta carotene than for vitamin A. Supplementation was associated with protection from death attributed to injuries and other miscellaneous causes. The maternal mortality ratio was 645 (42 deaths/ 6670 live births), 407 (29/7074), and 361 (23/6643) per 100 000 live births in the placebo, vitamin A, and beta carotene groups, respectively (P = 0.08 for vitamin A and 0.04 for beta carotene v placebo). The ratio for women receiving either vitamin A or beta carotene was 385, yielding a relative risk of 0.60 (0.39 to 0.93), representing a 40% reduction in mortality by this measure (P = 0.02). |
| **EFFECTS on Outcomes in childhood after 12 m: z scores for weight (WAZ), length/height (HAZ), and BMI for age (BMIZ), weight-for-height'length (WHZ), underweight, stunting, wasting, overweight, cognitive** | None | NA | NA |
| **Potential Biological mediating factors (as identified by authors of the primary studies)** | None mentioned | Lack of observed association between maternal serum retinol and infant mortality unlikely to have been confounded by factors such as other micronutrient deficiencies that affect both infant mortality and maternal serum retinol. However, it other micronutrient defi- ciencies, protein-energy malnutrition, or both, could have limited the effect of maternal vitamin A or b-carotene supplementation on early infant mortality. Women were not provided with iron or other supplements other than through regular health care channels, except in the 10% subset of women enrolled in the more detailed clinic study, which included severely anemic women who were treated. | The effect seemed to be more strongly associated with beta carotene (relative risk 0.56, P = 0.18) than vitamin A (relative risk 0.88, P = 0.73). Although the putative role of antioxidant defences in preventing dis- ease and an in vivo antioxidant role for beta carotene remain controversial, beta carotene, acting as an antioxidant could have reduced some forms of obstetric risk in this malnourished population. Low serum beta carotene concentrations have been observed in pregnant women with pre-eclampsia and eclampsia, whose pathogenesis entails vascular endothelial injury that may be associated with oxidative stress. Placental abruption has also been associated with depressed serum antioxidant concentrations, including beta carotene. |
| **Potential Behavioural mediating factors (as identified by authors of the primary studies)** | None given | None given | None given |
| **Potential Social mediating factors (as identified by authors of the primary studies)** | None given | None given | None given |
| **Details on who interventions are available to, and who is accessing services and in what numbers** | None given | Not given. However, compliance with treatment was relatively good, with half of all women receiving ≥80% of all possible doses (ie, equivalent to 80% of the RDA). The mean (± SD) proportion of doses taken was 70 ± 30%, and <75% of all women received at least half of all possible doses (equivalent to 50% of the RDA). | None given |
| **Author reflections on factors affecting the success of preconception nutrition interventions, including factors relating to leadership, financing, governance, supplies, and capacity** |  | None | None. This paper is really about both preconcption and pregnancy supplementation so doesn’t dicsuss preconception. |
| **Author reflections on factors affecting the failure of preconception nutrition interventions, including factors relating to leadership, financing, governance, supplies, and capacity** |  | None | None. |
| **Author recommendations on strategies to improve preconception nutrition in South Asia** |  | None | Raising the intake of preformed vitamin A or provitamin A carotenoids towards the values recommended for pregnancy or lactation from before and throughout pregnancy, presumably by supplementation or by dietary means, can complement antenatal and essential obstetric services in lowering maternal mortality in rural south Asia |
| **Author recommendations on future research to improve preconception nutrition in South Asia** |  |  |  |
| **Barriers to Preconception nutritional intake** |  | Not mentioned | Not discussed |
| **Enablers to preconception nutritional intake** |  | Not mentioned | Not discussed |
| **Risk of Bias assessment ROB2 completed?** |  |  |  |
| **ABSTRACT** | A folic acid containing multivitamin preparation was evaluated for its efficacy in preventing recurrence of open neural tube defect (NTD) in a blind, placebo-controlled randomized trial. The trial was carried out at the five centres in India, viz., Bangalore, Mumbai, Lucknow, New Delhi and Pune. The preparation contained 4 mg of folic acid besides calcium, iron, zinc and vitamins A, B1, B2, B6, C, D and nicotinamide. The placebo contained calcium and iron only. A total of 466 women with previous history of giving birth to a child with open NTD were included in the trial (231 in the vitamin group and 235 in the placebo group). The supplementation was given for at least one month prior to conception and up to three months after conception. All women were offered antenatal diagnosis with screening of maternal serum alpha foetoprotein (AFP) and ultrasound. Pregnancy outcome with reference to recurrence of NTD was unknown in 137 women in the vitamin group and 142 in the placebo group. The recurrence of open NTD in the vitamin group was 2.92 per cent compared to 7.04 per cent in the placebo group, a reduction by about 60 per cent. The difference, however, was not statistically significant (P = 0.06). The study seems to support the role of periconceptional folic acid supplementation in prevention of recurrence of NTDs in the Indian population. The reason for high recurrence rate observed in the placebo group requires further investigation. | Background: The effect of vitamin A supplementation on the survival of infants aged <6 mo is unclear. Because most infant deaths occur in the first few month of life, maternal supplementation may improve infant survival. Objectives: The objective was to assess the effect of maternal vitamin A or b-carotene supplementation on fetal loss and survival of infants <6 mo of age. Design: Married women of reproductive age in 270 wards of Sarlahi district, Nepal, were eligible to participate. Wards were randomly assigned to have women receive weekly doses of 7000 mg retinol equivalents as retinyl palmitate (vitamin A), 42 mg all-trans-b-carotene, or placebo. Pregnancies were followed until miscarriage, stillbirth, maternal death, or live birth of one or more infants, who were followed through 24 wk of age. Results: A total of 43559 women were enrolled; 15832 con- tributed 17 373 pregnancies and 15 987 live born infants to the trial. The rate of fetal loss was 92.0/1000 pregnancies in the placebo group, comparable with rates in the vitamin A and b-carotene groups, which had relative risks of 1.06 (95% CI: 0.91, 1.25) and 1.03 (95% CI: 0.87, 1.19), respectively. The 24-wk mortal- ity rate was 70.8/1000 live births in the placebo group, compa- rable with rates in the vitamin A and b-carotene groups, which had relative risks of 1.05 (95% CI: 0.87, 1.25) and 1.03 (95% CI: 0.86, 1.22), respectively. Conclusions: Small weekly doses of vitamin A or b-carotene given to women before conception, during pregnancy, and through 24 wk postpartum did not improve fetal or early infant survival in Nepal. Am J Clin Nutr 2000;71:1570–6. | Objective To assess the impact on mortality related to pregnancy of supplementing women of reproductive age each week with a recommended dietary allowance of vitamin A, either preformed or as beta carotene. Design Double blind, cluster randomised, placebo controlled field trial. Setting Rural southeast central plains of Nepal (Sarlahi district). Subjects 44 646 married women, of whom 20 119 became pregnant 22 189 times. Intervention 270 wards randomised to 3 groups of 90 each for women to receive weekly a single oral supplement of placebo, vitamin A (7000 microgram retinol equivalents) or beta carotene (42 mg, or 7000 microgram retinol equivalents) for over 31⁄2 years.  Main outcome measures All cause mortality in women during pregnancy up to 12 weeks postpartum (pregnancy related mortality) and mortality during pregnancy to 6 weeks postpartum, excluding deaths apparently related to injury (maternal mortality). Results Mortality related to pregnancy in the placebo, vitamin A, and beta carotene groups was 704, 426, and 361 deaths per 100 000 pregnancies, yielding relative risks (95% confidence intervals) of 0.60 (0.37 to 0.97) and 0.51 (0.30 to 0.86). This represented reductions of 40% (P < 0.04) and 49% (P < 0.01) among those who received vitamin A and beta carotene. Combined, vitamin A or beta carotene lowered mortality by 44% (0.56 (0.37 to 0.84), P < 0.005) and reduced the maternal mortality ratio from 645 to 385 deaths per 100 000 live births, or by 40% (P < 0.02). Differences in cause of death could not be reliably distinguished between supplemented and placebo groups. Conclusion Supplementation of women with either vitamin A or beta carotene at recommended dietary amounts during childbearing years can lower mortality related to pregnancy in rural, undernourished populations of south Asia. |

| **SN** | **4** | **5** | **6** |
| --- | --- | --- | --- |
| **Short name** | Khambalia2009 | De'Souza2021 | Kumaran2017 |
| **Type of paper** | Trial findings | Trial findings | Trial protocol |
| **Authors** | Amina Z Khambalia,, Deborah L O'Connor, Colin Macarthur, Annie Dupuis, Stanley H Zlotkin | Naomi D’souza, Rishikesh V. Behere, Bindu Patni, Madhavi Deshpande, Dattatray Bhat, Aboli Bhalerao, Swapnali Sonawane, Rohan Shah, Rasika Ladkat, Pallavi Yajnik, Souvik K. Bandyopadhyay, Kalyanaraman Kumaran, Caroline Fall and Chittaranjan S. Yajnik | Kumaran, K., P. Yajnik, H. Lubree, C. Joglekar, D. Bhat, P. Katre, S. Joshi, R. Ladkat, C. Fall and C. Yajnik |
| **Year** | 2009 | 2021 | 2017 |
| **Title** | Periconceptional iron supplementation does not reduce anemia or improve iron status among pregnant women in rural Bangladesh | Pre-conceptional Maternal Vitamin B12 Supplementation Improves Offspring Neurodevelopment at 2 Years of Age: PRIYA Trial. | The Pune Rural Intervention in Young Adolescents (PRIYA) study: design and methods of a randomised controlled trial. |
| **Citation** | Khambalia AZ, O'Connor DL, Macarthur C, Dupuis A, Zlotkin SH. Periconceptional iron supplementation does not reduce anemia or improve iron status among pregnant women in rural Bangladesh. Am J Clin Nutr. 2009 Nov;90(5):1295-302. | D’souza N, Behere RV, Patni B, Deshpande M, Bhat D, Bhalerao A, Sonawane S, Shah R, Ladkat R, Yajnik P, Bandyopadhyay SK, Kumaran K, Fall C and Yajnik CS (2021) Pre-conceptional Maternal Vitamin B12 Supplementation Improves Offspring Neurodevelopment at 2 Years of Age: PRIYA Trial. Front. Pediatr. 9:755977. | Kumaran, K., P. Yajnik, H. Lubree, C. Joglekar, D. Bhat, P. Katre, S. Joshi, R. Ladkat, C. Fall and C. Yajnik (2017). "The Pune Rural Intervention in Young Adolescents (PRIYA) study: design and methods of a randomised controlled trial." BMC Nutrition 3(1): 41. https://doi.org/10.1186/s40795-017-0143-5 |
| **doi** | doi: 10.3945/ajcn.2009.28350. | doi: 10.3389/fped.2021.755977 | doi:10.1186/s40795-017-0143-5 |
| **Clinical trials registration no** | www.clinicaltrials.gov as NCT00953134.  https://clinicaltrials.gov/study/NCT00953134 | ISRCTN32921044 https://doi.org/10.1186/ISRCTN32921044 CTRI 2012/12/003212, registered on 02/12/2012. Retrospectively registered. | ISRCTN32921044 https://doi.org/10.1186/ISRCTN32921044 CTRI 2012/12/003212, registered on 02/12/2012. Retrospectively registered. |
| **Context / setting** | Bangladesh: Kaliganj, 1 of 5 administrative divisions in Gazipur district, central Bangladesh. | Pune, India | Pune, India |
| **Protocol citation** | This paper gives methods and baseline characteristics but is not a formal trial protocol as such:  Khambalia, A., D. L. O’Connor and S. Zlotkin (2009). "Periconceptional Iron and Folate Status Is Inadequate among Married, Nulliparous Women in Rural Bangladesh12." The Journal of Nutrition 139(6): 1179-1184. | Kumaran, K., P. Yajnik, H. Lubree, C. Joglekar, D. Bhat, P. Katre, S. Joshi, R. Ladkat, C. Fall and C. Yajnik (2017). "The Pune Rural Intervention in Young Adolescents (PRIYA) study: design and methods of a randomised controlled trial." BMC Nutrition 3(1): 41. https://doi.org/10.1186/s40795-017-0143-5 | this is the protocol |
| **Arising from which main trial** | No name given | Pune Rural Intervention in Young Adolescents (PRIYA) study | Pune Rural Intervention in Young Adolescents (PRIYA) study |
| **Study design** | randomized, double-blind, controlled trial | RCT: randomised placebo controlled trial with three arms | RCT: randomised placebo controlled trial with three arms |
| **Has Preconception intervention only arm (1/0)** | 1 | 0 | 0 |
| **Has Preconception + Pregnancy intervention arm (1/0)** |  | 1 | 1 |
| **Has Pregnancy intervention only arm (1/0)** |  |  |  |
| **Has Pregnancy, postpartum + childhood intervention arm (1/0)** |  | 0 | 0 |
| **Has Control arm with only usual standard of care or less intensive intervention in preconception & pregnancy (1/0)** | 1 | 1 | 1 |
| **MMN in Preconception arm** |  |  |  |
| **B12 in Preconception arm** |  | 1 | 1 |
| **LNS in Preconception arm** |  |  |  |
| **Viatmin A in preconception arm** |  |  |  |
| **Iron in preconception arm (both arms had Folic acid)** | 1 |  |  |
| **Nutritious snack in Preconception arm** |  |  |  |
| **Pregnancy outcomes** | 1 | 1 | 1 |
| **Birth outcomes** |  | 1 | 1 |
| **Infancy outcomes** |  | 0 | 0 |
| **Child outcomes** |  | 1 | 1 |
| **Narrative summary of main finding** | Daily peri-conceptional IFA supplementation compared with FA supplementation only did not reduce anemia or improve iron status among pregnant women..  2 unique design factors: the intervention (IFA or FA) commenced before **or very early in the first trimeste**r of pregnancy rather than later during pregnancy, and the supplement was provided as a powder added to food rather than a more traditional tablet or capsule.  Among pregnant women in the current study, periconceptional IFA supplemen tation compared with FA supplementation did not significantly decrease anemia at 15 wk of gestation. There was no clinically significant change in maternal hemoglobin among pregnant women despite iron supplementation. | Combination of B12 supplementation in Preconception & pregnancy improved BSID-II cognitive and ? | No findings as this is a protocoll. |
| **Date of data collection** | March 2007 to the end of February 2008 | Vitamin intervention began Sept 2012, Protein May 2013, Interim measurements Jun 2014, 1st delivery Jun 2014. Between May 2017 and February 2020, approached the parents of 85 children who had attained the age of 2 years, for participation in the neurodevelopmental study. Had to halt the assessments after February 2020 due to the COVID-19 pandemic. | Vitamin intervention began Sept 2012, Protein supplement in May 2013, Interim measurements began Jun 2014, 1st delivery Jun 2014. |
| **Study objectives** | Primary aim: to examine the effect of daily periconceptional IFA compared with folic acid (FA) in a powdered supplement on anemia, hemoglobin concentrations, and other iron indicators during pregnancy. The periconceptional period was defined as the period before the ascertainment of a pregnancy during the study period. Outcomes were also measured for women who did not become pregnant during the study period.  Rationale for IFA supplementation in nonpregnant women is to treat preexisting anemia, increase body iron stores, and reduce the risk of neural tube defects. | To determine whether pre-conceptional B12 supplementation of adolescents/young adults:  improves the B12 status of their new-borns  improves birth weight, neonatal body composition (increased lean mass and reduced adiposity), insulin sensitivity and cognitive function in the children  alters the methylome, transcriptome, and metabolome in the cord blood of babies born in the trial. | To determine whether pre-conceptional B12 supplementation of adolescents/young adults: - improves the B12 status of their new-borns - improves birth weight, neonatal body composition (increased lean mass and reduced adiposity), insulin sensitivity and cognitive function in the children - alters the methylome, transcriptome, and metabolome in the cord blood of babies born in the trial. |
| **Sample size / power** | Sample size calculations estimated that 30 pregnancies per group would be an adequate sample size to detect a clinically and statistically significant difference of 8 g/L in hemoglobin concentration between experimental and control groups (on the basis of 80% power and an SD of 11 g/L) (18). On the basis of a pilot study, authors assumed that 20% of women would become pregnant; thus, authors aimed to recruit 152 women per group.  The final sample size of >30 women per treatment group provided us with 80% power to detect a difference of 8 g/L (+/-11 g/L) in hemoglobin concentration at a 0.05 significance level. Our study showed that the actual difference in hemoglobin concentration between the treatment groups was much smaller (mean: 1.00 g/L; 95% CI: 24.1, 6.1) and had a larger degree of individual variability. A larger sample size would have been needed to detect a statistically significant difference. | Power calculated from Pune observational data; a 1 SD higher maternal B12 concentration was associated with a 0.5 SD higher cord blood B12 concentration. Our earlier pilot intervention study (in men, non-pregnant women and children) showed that a dose of 2 μg of B12 led to a rise in B12 concentrations of approximately 1 SD over one year, with most of that increase achieved within 4 months.  Based on **180–200 pregnancies in the female participants**, the trial will have 78–82% power to detect a change in newborn B12, with B12 supplementation compared with placebo, of 0.5 SD, at the 5% significance level. | Calculated the power from observational data from Pune relating maternal B12 concentrations to cord blood B12 concentrations; a 1 SD higher maternal B12 concentration was associated with a 0.5 SD higher cord blood B12 concentration. Our earlier pilot intervention study (in men, non-pregnant women and children) showed that a dose of 2 μg of B12 led to a rise in B12 concentrations of approximately 1 SD over one year, with most of that increase achieved within 4 months [34].  Based on 180–200 pregnancies in the female participants, the trial had 78–82% power to detect a change in newborn B12, with B12 supplementation compared with placebo, of 0.5 SD, at the 5% significance level. |
| **Sample characteristics** | Rural area has a high population density, relies primarily on agricultural labor. Predominantly Muslim population. | Married non-pregnant women were recruited from six villages around Pune in 1993 and those who became pregnant were followed up. 762 children were born and followed up serially. At ∼17 years of age, 690 participants from the PMNS cohort were screened for inclusion in the PRIYA trial. 557 PMNS cohort (266 females & 291 male) participants were randomized. | Screening procedures included height, weight and blood pressure measurements, a clinical medical examination, and a random blood sample. |
| **Randomisation** | Married, nulliparous women were randomly assigned to receive daily iron and folic acid (IFA; 60 mg ferrous fumarate and 400 lg folic acid) (n = 134) or folic acid (FA; 400 lg) (n = 138) in the form of a powdered supplement added to food and then followed up monthly for 9 mo or until they became pregnant. A total of 272 women were randomized to supplement groups, (acceptance rate of 89.8%). | Five hundred and fifty-seven (266 females) participants were randomized to receive either a placebo, B12 (2 μg/day) + multiple micronutrients (MMN) or B12 alone (2 μg/day). | 690 F1 generation screened of whom 557 (266 girls and 291 boys) were randomised individually into one of three groups to receive either: i) 2 μg B12, ii) 2 μg B12 plus multiple micronutrients (MMN) plus 20 g milk powder (equivalent to 5 g milk protein), or iii) placebo daily. Sequence generation: computer generated random sequence numbering system using STATA. |
| **Blinding** | Participants were blinded to which supplement they were on. Sachets were identical in appearance, except for a small embossed letter on the back of the sachet to identify the treat- ment group (“A” or “B”). | Participants and the study team were blinded to the vitamin/micronutrient supplementation | The participants and the study team are blinded to the vitamin/micronutrient supplementation. After randomisation, a name list participants in each allocation group was used by an independent staff member not involved in the day-to-day running of the study to package the bottles of capsules into boxes labelled only with the participants’ names (not with the allocation group). The labels clearly highlighted that the contents were for the consumption of the individual named person only.  The milk powder intervention is not blinded. It was not possible to supply a placebo powder to the other groups, in order to blind the milk powder intervention due to financial constraints. The field workers who distributed the supplements were unaware that it is only the MMN group that receives milk powder.  All outcome assessors were blinded to the allocation groups. |
| **Age of participants (years)** | <40 years. Minimum age of married woman not specified | ∼16-18 y | ∼16-18 y |
| **Inclusion criteria** | Nulliparous, <40 years, married, living in household >6 months, living with husband, not using impant or not having surgery to prevent pregnancy, had not used iron supplements in last 3 months. NOT PREGNANT To identify eligible women, field interviewers made house-to-house visits starting with households located nearest to the medical clinic where blood samples would be collected and moved outward from this location in a circular fashion. At recruitment, women who were otherwise deemed eligible but had not menstruated for >45 d were administered a urine pregnancy test. Only women with a negative urine pregnancy test were included in the study. | Adolescents (~16–18y) belonging to the original Pune Maternal Nutrition Study (PMNS) cohort girls and boys which was set up in 1993 | Adolescents (~16–18y) belonging to the original PMNS cohort |
| **Exclusion criteria** | Women were ineligible if they met at least one of the following criteria:  not married,  had previously given birth,  age >=40 y old,  not a permanent household member (living in household <6 mo),  not living in same household as their husband,  using an implant form of birth control,  previous surgery to prevent pregnancy,  had used iron supplements within the previous 3 mo,   known to be pregnant at enrollment, or  were identified as severely anemic at baseline (hemoglobin concentration of <70 g/L) (1).  Severely anemic women (n = 1) were excluded from the study and provided with iron supplements | i) Severe vitamin B12 deficiency (<100 pmol/L) or severe anaemia (haemoglobin < 7 g/dL) ii) Severe physical and mental disability likely to interfere with marriage and reproduction iii) Serious systemic illness (that would prohibit participation in any clinical trial e.g., malignancy, reproductive disorder leading to infertility, congenital or acquired cardiovascular disease with New York Heart Association Functional Classification III or IV) iv) Treatment with drugs that interfere with 1-C metabolism (e.g., those interfering with folate metabolism such as phenytoin, valproic acid, carbamazepine, trimethoprim, methotrexate and those interfering with B12 absorption such as metformin, proton pump inhibitors) v) Treatment with B12 supplements for more than 30 days at the time of randomisation | i) Severe vitamin B12 deficiency (<100 pmol/L) or severe anaemia (haemoglobin < 7 g/dL) (due to ethical considerations in a placebo controlled trial) ii) Severe physical and mental disability likely to interfere with marriage and reproduction iii) Serious systemic illness (that would prohibit participation in any clinical trial e.g., malignancy, reproductive disorder leading to infertility, congenital or acquired cardiovascular disease with New York Heart Association Functional Classification III or IV) iv) Treatment with drugs that interfere with 1-C metabolism (e.g., those interfering with folate metabolism such as phenytoin, valproic acid, carbamazepine, trimethoprim, methotrexate and those interfering with B12 absorption such as metformin, proton pump inhibitors) v) Treatment with B12 supplements for more than 30 days at the time of randomisation. |
| **Preconception intervention characteristics** | Women were randomly assigned to receive daily iron (60 mg as ferrous fumarate) and FA (400 micrograms) or FA alone (400 micrograms). Iron dose corresponds to recommendations where prevalence of anemia >40%.  FA dose is based on the amount of synthetic FA recommended for women who plan a pregnancy (or are capable of becoming pregnant) for prevention of a neural tube defect (20).  Nutrients were delivered in a powdered form in individual sachets. Sachets were identical in appearance, except for a small embossed letter on the back of the sachet to identify the treat- ment group (“A” or “B”). The use of these sachets, which contained powdered micronutrients, is a home-fortification strategy that allows micronutrients to be added to semisolid foods for consumption (Sprinkles; Ped-Med Ltd, Toronto, Canada). Each woman was given 35 sachets at enrollment and at each moni- toring visit (30 sachets for a 1-mo daily supply and 5 extra sa- chets). Participants were instructed to pour the entire contents of the package into any semiliquid food after the food had been cooked and at a temperature acceptable to eat and then to mix it into the food. Participants were told to use one full package per day at any mealtime. | 2 intervention arms:  i) 2 μg B12 daily (2* 1μg capsules) ,  ii) Daily 2 μg B12 (1* 1μg B12 cap) plus multiple micronutrients (MMN)  Vitamin A - 300 µg Vitamin D - 100 IU Vitamin E - 5 mg Vitamin C - 20 mg Vitamin B1 - 0.75 mg Vitamin B2 - 0.9 mg Vitamin B3 - 10 mg Vitamin B6 - 0.5 mg Vitamin B12 - 1.0 µg Zinc - 6 mg Copper - 1 mg Selenium - 20 µg Iodine - 75 µg  Plus 20 g milk powder (equivalent to 5 g milk protein). All participants are separately administered weekly 100 mg iron and 500 μg folic acid &  Treated with a six month course of B12 at the end of the study. | Study supplements started in preconception amongst the PMNS girls until their 1st delivery.  (i) 2 μg B12 daily (2* 1μg capsules) ,  ii) Daily 2 μg B12 (1* 1μg B12 cap) plus multiple micronutrients (MMN incl 1μg B12) + 20g milk powder incl. 5g milk protein |
| **Preconception control group intervention** | FA was used as the control because the protective effect of folate against the development of neural tube defects, specifically anencephaly and spina bifida, is well established (21, 22). Nutrients were delivered in a powdered form in individual sachets. Sachets were identical in appearance, except for a small embossed letter on the back of the sachet to identify the treatment group (“A” or “B”). The use of these sachets, which contained powdered micronutrients, is a home-fortification strategy that allows micronutrients to be added to semisolid foods for consumption (Sprinkles; Ped-Med Ltd, Toronto, Canada). Each woman was given 35 sachets at enrollment and at each monitoring visit (30 sachets for a 1-mo daily supply and 5 extra sachets). Participants were instructed to pour the entire contents of the package into any semiliquid food after the food had been cooked and at a temperature acceptable to eat and then to mix it into the food. Participants were told to use one full package per day at any mealtime. | 1 control arm:  iii) placebo capsules daily. All participants separately administered weekly 100 mg iron and 500 μg folic acid. All participants treated with a six month course of B12 at the end of the study. | Iron (100mg), Folic acid (500mcg) tablets to all 3 group provided as per the Government of India Guidelines.- 1 per week in adoescence, 100 tablets during pregnancy. |
| **Intervention timing (how far before conception)** | After enrolment 9 monthly follow-up visits were made. n = 31 women had an unidentified pregnancy between recruitment and 1st monthly follow-up visit. They received supplements for a mean 26.3 +/- 12.3 days after conception.  The majority of women (n = 57) started supple- mentation before conception (72.9 +/- 57.8 d before estimated conception).  No. of days between initiation of supplementation and conception did not differ by supplement group (P = 0.27). The median number of days from when supplementation was commenced and conception occurred, as indicated by last menstruation was **25.5 d** and ranged (first–third quartiles) from -16.5 to 82.5 days. | 3 years or until 1st delivery for girls or, for the boys, until their wives become pregnant | Duration: Orginally 3 years or until delivery of the first child, whichever is earlier but the protocol says it was extended the study to cover intervention for at least five years or until delivery so that adequate numbers of deliveries take place to enable a proper assessment of the primary outcome. This may have been over-ridden by COVID-19 stopping trial activities. |
| **Intervention delivery (health systems, food systems, social protection systems)** | Deliivery system by research staff (not within health system) Field interviewers visited participants once a month to replenish supplies of sachets, to count the number of empty sachet packages, to monitor side effects, and to encourage women to consume the contents of the sachets. | 3 years or until 1st delivery for girls or, for the boys, until their wives become pregnant | Intervention commenced September 2012. All participants were visited individually and counselled again before being given the supplements. Participants received supplements on a monthly cycle (with a buffer of 3 extra days’ supply). The protein supplements were started in May 2013. |
| **Pregnancy detection** | During monthly visits, participants were asked if they had menstruated in the past 45 d. After 2 missed menstruations, a urine-based human gonadotropin pregnancy test was administered. |  | Field workers monitor menstrual dates. When participants report missing a period, a urine pregnancy test or an ultrasound scan is done to confirm pregnancy. |
| **Pregnancy intervention characteristics (if any)** | None. Once a pregnancy was confirmed, women were removed from the study and advised to take IFA supplements (17). |  | All pregnant women (including the wives of male participants) are given and advised to take 1 tablet daily of 100 mg iron and 500 μg folic acid for 100 days to prevent anaemia. Study supplements continued among the PMNS girls until their 1st delivery.  (i) 2 μg B12 daily (2* 1μg capsules) ,  ii) Daily 2 μg B12 (1* 1μg B12 cap) plus multiple micronutrients (MMN incl 1μg B12) + 20g milk powder incl. 5g milk protein |
| **Pregnancy control group intervention (if any)** | NA |  | All pregnant women (including the wives of male participants) are given and advised to take 1 tablet daily of 100 mg iron and 500 μg folic acid for 100 days to prevent anaemia. |
| **Interventions after birth (if any)** |  |  |  |
| **Control group intervention after birth (if any)** |  |  |  |
| **Compliance measurement** | Field interviewers visited participants once a month to replenish supplies of sachets, to count the number of empty sachet packages, to monitor side effects, and to encourage women to consume the contents of the sachets. For each participant, adherence was calculated as percentage of the total eligible doses consumed during the study period.  Indicators: Total no. of sachets used Overall percentage adherence  On the assumption that plasma folate concentrations would reflect adherence, wauthors verified self-reported adherence by examining the association between adherence and plasma folate concentrations. The association was statistically significant among nonpregnant but not pregnant women. Pregnant women may have reported their adherence inaccurately or were at different stages of hemodilution at the time of blood sampling, which affected the relation between self-reported intake and plasma folate concentration. |  | Compliance is assessed by retrieving the containers of the tablets and counting the number remaining in the container every month |
| **Adverse events** | Not mentioned | Data Safety Monitoring Board (DSMB) monitored adverse events. | Adverse events and morbidity are monitored on a monthly basis using a standardised questionnaire. All relevant information about any illness is gathered, as well as details of any medication (self-administered or prescribed by a doctor). Serious adverse events (SAE; death, hospital admissions for more than 24 h, debilitating illness) are recorded and investigated by medical personnel overseen by the PI.  Regular reports are submitted to the Ethics Committee and the Data Safety Monitoring Board (DSMB). |
| **Engagement, category (including nutrition sensitive/specific)** | Nutrition specific | Nutrition specific | Nutrition specific intervention |
| **Where in the overall UNICEF 2020 framework the intervention lies** | Intervention changing an the immediate determinant of Diet: sprinkles containing IFA or FA are directly altering altering diet | Intervention changing an the immediate determinant of Diet: B12 supplement and milk is directly altering altering diet | Intervention changing an the immediate determinant of Diet: B12 supplement and milk is directly altering altering diet |
| **Where in the Partap 2021 framework the intervention lies** | Giving sprinkles containing IFA or FA is an individual-level nutrition intervention. | B12 supplement and milk is an individual-level nutrition intervention. | B12 supplement and milk is an individual-level nutrition intervention. |
| **Proposed mechanism of intervention action** |  |  |  |
| **Exposures relating to preconception nutrition** | IFA versus FA: daily iron (60 mg as ferrous fumarate) and FA (400 micrograms) or FA alone (400 micrograms) in the form of "sprinkles" in sachets to be added to food. | Daily 2 μg Vitamin B12 with or without additional micronutrients and 5g of milk protein/ day | Daily 2 μg Vitamin B12 with or without additional micronutrients and 5g of milk protein/ day |
| **Preconception measures** | At enrolment study participants were administered a standardized baseline interview on socioeconomic and reproductive health factors. Anthropometric measurements included Weight (kg), Height (cm), BMI (kg/m2).   Venous blood samples for measurement of : Hemoglobin (g/L) Plasma ferritin (lg/L) Plasma transferrin receptor (mg/L) C-reactive protein (mg/L) Plasma folate (nmol/L) Anemia (%) - defined as hemoglobin <120 g/L among nonpregnant women and hemoglobin <110 g/L among pregnant women Iron deficiency (%)- Defined as plasma ferritin concentration <12 lg/L. Iron deficiency and anemia (%)- Defined as hemoglobin concentration <120 g/L and plasma ferritin concentration <12 lg/L. | Blood samples at screening (17y and again 6 to 18 months after screening c.18y): Vitamin B12 (pM) Holo-TC Folate (nM) Homocysteine (μmol/L) note other preconception measures not reported here | **Blood samples** at 6 to 18 months after screening / intervention start (at 18 years):  Hemogram Vitamin B12 Folate Homocysteine DNA Insulin Adiponectin Leptin Vitamin B2 Vitamin B6 |
| **Concommittant care** | Not mentioned | see protocol | Women diagnosed with gestational diabetes are appropriately treated. Local physicians manage the women as per routine standards of care. Record all concomitant medication, including vitamin supplements. Husbands of the female participants are offered anthropometric and blood tests at the time of the 28 weeks measurements (hemogram, blood glucose). Wives of the male participants are offered the same tests during pregnancy as the female participants in the study. |
| **Follow-up schedule for measurements** | Study participants were administered a standardized baseline interview on socioeconomic and reproductive health factors.  Anthropometric measurements included weight and height.  Field interviewers visited participants once a month to replenish supplies of sachets, to count the number of empty sachet packages, to monitor side effects, and to encourage women to consume the contents of the sachets.   Venous blood samples were collected at baseline and after a pregnancy was ascertained or after 9 mo among nonpregnant women.  Hemoglobin was assessed using a hemoglobinometer (HemoCue, Angelholm, Sweden).  Venous blood was centrifuged at 1500 x g for 10 min, and plasma was separated from cells. Aliquots of plasma were stored at 220°C and shipped in a frozen state on dry ice to ICDDR,B in Bangladesh for plasma ferritin and transferrin receptor (TfR) analyses and to the Hospital for Sick Children (Toronto, Canada) for plasma folate analyses.  Aliquots of plasma for folate analyses included sodium ascorbate (1%, wt:vol) to prevent the oxidation of folate.  Plasma folate concentrations were measured by using a microbiologic assay that uses the test organism Lactobacillus rhamnosus (ATCC 7649; American Type Tissue Culture Collection, Manassas, VA) as described by Molloy and Scott (25).  Plasma ferritin was measured by using an enzyme-linked immunosorbent assay method with a commercial kit (Roche Diagnostics, Indianapolis, IN). Plasma TfR was measured by using an enzyme-linked immunosorbent assay method with a commercial kit (BioVendor, Modrice, Czech Republic) | Screening at 17 years.  Blood samples at 6 to 18 months after screening (c.18 years)  Blood samples at 28 weeks gestation. Measures of birth in the facility - cord blood and anthropometry.  Neurodevelopmental assessmnt between 24 and 42 months usng BSID-III  Participants were followed up regularly for health problems, and marriages were recorded. Married women were monitored to detect pregnancy which was confirmed by a urine pregnancy test.  At 24–28 weeks gestation, mothers visited the Diabetes Unit, KEM Hospital Research Center Pune, for a fasting oral glucose tolerance test and clinical and biochemical evaluations incl. anthropometric measurements, an obstetric consultation and estimate of fetal growth by ultra-sonography.  Socio demographic information and details of deliveries were recorded (gestational age and type of delivery) collected through interviews. |  |
| **Summary of baseline comparisons by study arm** | Age (y) Age at menarche (y) Age at marriage (y) Can read and write a letter (%)  Had a previous miscarriage (%)  Uses a contraceptive device (%)  Weight (kg) Height (cm) BMI (kg/m2)  No differences existed between groups, except in literacy. Women who were randomly assigned to the IFA group had a higher literacy rate (able to read and write a letter) than those in the FA group (P = 0.02). A separate comparison of baseline and follow-up measures for pregnant and nonpregnant women showed no significant differences by supplement group, except for a significantly higher proportion of literate pregnant women in the IFA group. Literacy was adjusted for in all multivariate analyses. There were no statistically significant differences in baseline characteristics or in biochemical indexes for iron and folate between women who completed the study and those lost to follow-up. | There were 42 boys and 32 girls; of these, 27 were in the placebo group, 26 in the B12 + MMN and 21 in the B12 alone group. There were no differences in gestational age at delivery, birth weight, length or head circumference amongst the offspring in the three supplementation groups. Similarly, there were no differences in parental education, standard of living index, maternal age, or IQ. Children who were not invited for the study because they were below 24 months of age differed from those studied; they had higher socio-economic status and parental education, higher maternal and cord B12 and holo-TC, and lower cord homocysteine compared to the study group. At baseline before supplementation (at 17 years), maternal B12 and holo-TC levels were similar across the three supplementation groups. | Examined whether randomization had equally distributed potential confounders such as parental education and standard of living index, maternal age, IQ, and anthropometry, length of supplementation and compliance across the three supplementation groups. |
| **Pregnancy outcomes: gestational weight gain, gestational diabetes, maternal anthropometry (weight, MUAC, height), anaemia,** | Primary outcomes included hemoglobin concentration among women who became pregnant and at 9 months after enrolment amonst those who did not fall pregnant.    Outcomes were measured in women after pregnancy was detected and amongst women who did not become pregnant during the study period.  Other outcomes reported Hemoglobin (g/L) Plasma ferritin (lg/L) Plasma transferrin receptor (mg/L) C-reactive protein (mg/L) Plasma folate (nmol/L) Anemia (%) - defined as hemoglobin <120 g/L among nonpregnant women and hemoglobin <110 g/L among pregnant women Iron deficiency (%)- Defined as plasma ferritin concentration <12 lg/L. Iron deficiency and anemia (%)- Defined as hemoglobin concentration <120 g/L and plasma ferritin concentration <12 lg/L. Total no. of sachets used Overall percentage adherence No. of times reported side effects Loose motion Constipation Nausea Dark stools Length of supplementation   Changes in hemoglobin, plasma ferritin, and plasma TfR concentrations from baseline. | Blood samples of mother at 28 weeks gestation:  Hemoglobin (gm/dl)  Vitamin B12 (pM)  Holo-TC (pM) Folate (nM) Vitamin B2 (pM) Viamin B6-pyridoxal-5-phospate (pM)  Vitamin B6-pyridoxal (pM)  Homocysteine (μmol/L) | Ultrasound scans are carried out at 28 weeks gestation, when blood pressure is also measured, and an OGTT performed (75 g anhydrous glucose) Blood samples at 28 weeks gestation:  Hemogram OGTT (oral glucose tolerance test) Insulin Vitamins B12, B2, B6 Folate Homocysteine Leptin Adiponectin DNA/RNA Breast swab and Stool: Microbiota |
| **Preconceptual outcomes: (BMI, Hb or other outcomes which could be attributed to preconception care)** |  |  |  |
| **Birth outcomes in child: birthweight, low birth weight (LBW), small for gestational age (SGA), preterm birth/ delivery (PTB), cord blood b12, DXA,** | None | Child characteristics Child age at assessment (months) Gender Birth anthropometry Gestation age (weeks) Birth weight (gm) Birth length (cm) Head circumference (cm) Cord micronutrients Vitamin B12 (pM) Holo-TC (pM) Folate (nM) Vitamin B2 (pM) Vitamin B6-pyridoxal-5-phospate (pM) Vitamin B6-pyridoxal (pM) Homocysteine (μmol/L) BDNF (pg/ml) | None |
| **Outcomes in mother at birth** | None | Not reported here | **Blood samples:**  Hemogram Glucose Breast & vaginal swabs and Stool: Microbiota |
| **Post-natal outcomes (in Infancy)** | None | Not reported here |  |
| **Outcomes in childhood after 12 m: z scores for weight (WAZ), length/height (HAZ), and BMI for age (BMIZ), weight-for-height'length (WHZ), underweight, stunting, wasting, overweight, cognitive** | None | Neurodevelopmental outcomes at 24–42 months of age: Bayleys Scale of Infant Development III (BSID-III) - domains of Cognitive, Motor and Language development. | **Baby:** Body size & composition: Anthopometry DXA up to 2 years **Stool:** Microbiota up to 2 y **Long term:** Body size & composition Cardiometabolic Cognition |
| **Long term outcomes** | None | Not reported here | long-term outcomes, include growth, body composition, glucose and insulin parameters, cardiovascular disease risk markers, and cognitive function at intervals during childhood, and in adult life. |
| **Analysis** | Intent-to-treat analysis. Analyses conducted separately for pregnant women (IFA compared with FA) and nonpregnant women (IFA compared with FA). The main outcomes were anemia and changes in hemoglobin, plasma ferritin, and plasma TfR concentrations from baseline.  Plasma ferritin concentrations were log-transformed. As a secondary analysis, change in plasma folate concentrations from baseline were examined in both treatment groups. Plasma folate concentrations were also examined by using linear regression analysis with the independent variable adherence to examine the accuracy of self-reported intake of supplements.  Univariate analyses of hematologic indexes were performed by using t tests for continuous variables and chi-square tests for categorical variables. Multivariate analyses were performed by using linear regression models for continuous response variables and logistic regression for the binary response variable for anemia. Multivariate analyses for pregnant women were adjusted for treatment group, literacy, adherence, gestational age, and timing of supplementation. Gestational age was the number of days since last menstruation reported by women who were identified as pregnant after 2 missed menstruations and a positive urine pregnancy test. Timing of supplementation is the number of days between commencement of supplementation and conception (days of last menses minus first day of supplementation). Timing of supplementation was examined as a continuous variable and as a categorical variable by categorizing pregnant women into those who initiated supplementation before and after conception, respectively. Multivariate analyses for nonpregnant women were adjusted for literacy and adherence. A 2-sided P value of 0.05 indicated statistical significance. | All data were represented as either mean and standard deviation (for normally distributed variables) or median and 25–75th percentile (for skewed variables). The skewed outcome variables (maternal and child biochemical measures, birth outcomes and neurodevelopmental measures) were log transformed.  Used Pearson’s correlation coefficient to test associations between the length of supplementation and biochemical measures at 28-week gestation and offspring cord blood.  Compared differences in outcome variables between B12 alone or B12+MMN groups and the placebo group using the t-test. Adjustments for additional covariates (e.g., maternal B12 levels at screening) were performed using ANCOVA. authors also examined longitudinal changes in the logarithmic values of vitamin B12 concentrations between time points and treatment groups using a two-way repeated measures ANOVA. Further, authors examined the additional effect of duration of supplementation and compliance in the same model. authors used a non-parametric test (Mann-Whitney U-test) to test the significance of difference in cord BDNF values between the supplementation groups because BDNF values could not be normalized by various transformations. | Analysis by ‘intention to treat’ (ITT), only including newborns whose mothers received micronutrient supplementation for at least 3 months before conception. authors will also undertake a per protocol analysis limiting the analysis to babies whose mothers had at least 50% compliance. authors will undertake separate analyses for female and male participants. Adjust for vitamin B12 supplements prescribed outside the trial when analysing the primary outcome. Explore the effect of possible confounders and effect modifiers as appropriate.  Adjust analyses for maternal age, socio-economic status (before and after marriage), paternal BMI and height, gestational age at birth, and gender of the offspring.  Examine possible interaction between allocation group and the following pre-pregnancy factors: maternal BMI, height, age, pre-intervention B12 concentration; pregnancy factors: circulating folate, glucose and lipid concentrations; and fetal factors: newborn sex, and carry out stratified analyses when appropriate. Use analysis of variance to test for differences between group means. Appropriate transformations will be used to normalise data if required. |
| **EFFECTS on Preconception outcomes: maternal anthropometry (weight, MUAC, height. BMI), Haemoglobin (Hb), anaemia, iodine status** |  |  |  |
| **EFFECTS on Pregnancy outcomes: gestational weigt gain, gestational diabetes, maternal anthropometry (weight, MUAC, height), anaemia, iodine status** | Multivariate analyses showed that daily peri- conceptional IFA supplementation compared with FA supplementation **did NOT** - reduce anemia (64.4% at follow-up in FA arm, 65.1% in IFA arm p=0.93),  - improve Hb status (114.0 (108.0 to 123.0)g/dl at follow-up in FA arm, 112.0 (105.0 to 124.0) in IFA arm p=0.76), or   -improve iron status among pregnant women (Plasma ferritin 48.5 (24.5 to 70.5) & Plasma transferrin receptor 2.4 1.9 to 3.0) at follow-up in FA arm versus 47.2 (31.4 to 60.8) and 2.2 (1.9 to 2.7) respectively in IFA arm p=0.92 & p=0.66 respectively).  No clinically significant change in maternal hemoglobin among pregnant women despite iron supplementation. Among pregnant women, overall percentage adherence and timing of supplementation in relation to conception were significantly associated with change in hemoglobin concentration from baseline. Each 1% increase in percentage adherence was associated with a 10-g/L improvement in change in hemoglobin from baseline (P = 0.03). Women who initiated supplementation before conception showed a 7.3 g/L greater change in maternal hemoglobin from baseline compared with those who initiated supplementation after conception (P = 0.01). Median (first–third quartile) change in plasma folate concentration from baseline when women in both treatment groups were pooled for statistical analysis was 11.7 nmol/L (2.5 – 26.5 nmol/L) in pregnant women. The prevalence of inadequate folate status (<10 nmol/L) decreased from 8.8% to 7.5% (P = 0.71) among pregnant women. Self-reported adherence was not significantly associated with change in plasma folate concentrations among pregnant women (P = 0.20), regardless of whether the pregnant women initiated supplementation before or after conception (P = 0.18). Each 1% increase in overall percentage adherence was associated with only a 0.14 nmol/L improvement in change in plasma folate from baseline (P = 0.20) among pregnant women. | NB. p values for comparisons with control arm (placebo) using t-test.  Vitamin B 12 at 28 weeks: Placebo: 134.0 (95.5, 163.0), B12+MMN: 164.0 (149.0, 218.5) p=0.007; B12: 204.0 (173.5, 261.0) p<0.001. Holo-TC differed in both intervention arms.  No other outcomes differed at 28 wks in the B12 only arm.  Other vitamins differed in the B12+MMN + milk at 28 weeks' gestation: folate, B2, but NOT B6 or B6 pyridoxal 5-phosphate, homocysteine, Hb.  There was a rise in vitamin B12 and holo-TC levels in the B12 supplemented groups compared to the placebo group, both pre-conceptionally (18 years of age) and at 28 weeks of gestation. There was no significant association between length of supplementation (from start of supplementation till date of 28 weeks gestation and date of delivery) and circulating concentration of vitamin B12 in either group. There was a significant association between length of supplementation and holo-TC concentrations in the cord blood of the B12 alone group (r = 0.462 p = 0.035). Repeated measures ANOVA showed a significant effect of time (F = 18.517, p < 0.001) and treatment (F = 9.363, p< 0.001) on log serial B12 concentrations. Addition of length of supplementation and compliance did not change the result. Post hoc comparisons using Bonferroni correction, showed that the log B12 concentrations in both B12 + MMN (95% CI = 0.14, 0.56, p < 0.001) and B12 alone (95% CI = 0.17, 0.63, p < 0.001) groups were significantly higher than the placebo group.  There was no difference in vitamin B12 concentrations between B12 + MMN and B12 alone groups. Cord blood levels of holo-TC were significantly higher in both the B12 supplemented groups compared to the placebo group, though vitamin B12 levels were similar. Baseline plasma homocysteine concentrations were high but similar in the three supplementation groups, and fell substantially in the vitamin B12 supplemented groups pre-conceptionally. During pregnancy, as expected, plasma homocysteine concentrations fell in all groups. They were similar in the three groups during pregnancy and in the cord blood. Circulating folate concentrations were similar at baseline in the three groups and increased during pregnancy (due to supplementation). Folate levels were significantly lower at 28 weeks gestation in the B12 + MMN group compared to those in the placebo group. Folate levels were similar in the cord blood across the groups. Circulating B2 levels were higher in the B12 + MMN group as compared to the placebo group during 28 weeks gestation. Hemoglobin concentrations were similar in the mother and the offspring across all the groups. |  |
| **EFFECTS on Birth outcomes in child: birthweight, low birth weight (LBW), small for gestational age (SGA), preterm birth/ delivery (PTB)** | None | Birthweight, length and head circumference did not differ by arm, neither did B12 but Holo-TC did. Results for Placebo, B12+MMN & B12 only respectively: Birth weight: 2,908.6 (412.5); 2,809.2 (458.6); 2,788.9 (315.9), p for B12+ MMN vs Placebo: p=0.411; p for B12 only vs Placebo: vs p= 0.277. Birth length: 49.1 (46.8, 49.8); 48.2 (47.4, 49.8); 48.5 (47.2, 49.3), p for B12+ MMN vs Placebo: p=0.990; p for B12 only vs Placebo: vs p= 0.936 Birth head circumference: 33.4 (1.0); 33.1 (1.0); 33.0 (0.9) , p for B12+ MMN vs Placebo: p=0.237; p for B12 only vs Placebo: vs p= 0.142. Cord Holo-TC: 40.7 (23.3, 81.9); 79.4 (39.2, 125.0); 96.1 (39.4, 125.0). p for B12+ MMN vs Placebo: p=0.021; p for B12 only vs Placebo: vs p= 0.048. Cord B12: 226.0 (138.0, 289.0); 275.5 (181.7, 313.7) p=0.240; 289.0 (167.0, 446.0) p<0.200. Cord blood Brain Derived Neurotrophic Factor (BDNF) was higher in intervention than the placebo group, the B12 alone group had the highest values, however the difference was non- significant. |  |
| **EFFECTS on Outcomes in mother at birth** | None | None |  |
| **EFFECTS on Post-natal outcomes (in Infancy)** | None | None |  |
| **EFFECTS on Outcomes in childhood after 12 m: z scores for weight (WAZ), length/height (HAZ), and BMI for age (BMIZ), weight-for-height'length (WHZ), underweight, stunting, wasting, overweight, cognitive** | None | The offspring of mothers in the B12 alone group performed the best in the cognitive and language domains, and significantly better than the placebo group. This difference persisted after adjusting for the baseline plasma vitamin B12 concentrations. Cognition and language composite scores were 5–7% higher in the B12 alone group than the placebo group. There were no significant differences between the B12 + MMN group and the placebo group on any of the neurodevelopmental domains. Results in Placebo, B12 + MMN group, B12 group. p values adjusted for maternal baseline B12 levels Cognitive: 90.0 (85.0, 95.0); 90.0 (85.0, 96.2); 95.0 (90.0, 100). p values B12+MMN vs placebo p= 0.781; p values B12 only vs placebo p= 0.044 Motor: 94.0 (91.0, 100.0); 95.5 (90.2, 100.0); 97.0 (91.0, 107.0) . p values B12+MMN vs placebo p= 0.522; p values B12 only vs placebo p= 0.384 Language: 92.2 (7.8); 93.7 (9.87); 98.6 (10.1). p values B12+MMN vs placebo p= 0.633; p values B12 only vs placebo p= 0.020. Cord blood BDNF values did not show significant associations with any of the BSID-III composite scores. |  |
| **Potential Biological mediating factors (as identified by authors of the primary studies)** | None mentioned | The role of pre-conceptional folic acid supplementation in preventing NTDs is well-established, especially in western (mainly non-vegetarian) populations. In vegetarian populations like India, vitamin B12 is likely to play a similar role, because both folate and B12 act as cofactors for the enzyme methionine synthase, in methylation reactions. Studies in India have highlighted an association of both maternal vitamin B12 and folate with different outcomes in the offspring including neurodevelopmental performance. Vitamins B12 and folate participate in the one-carbon metabolism pathway to stimulate synthesis of precursor nucleotides for DNA synthesis, and also generate the universal methyl donor S-Adenosyl methionine (SAM) which is involved in methylation of DNA (an important epigenetic mechanism), proteins and lipids and generating neurotransmitters. These mechanisms are reputedly involved in fetal growth and differentiation and a deficiency or imbalance of these vitamins may result in a permanent change in the structure and function of developing tissues which may manifest as disorders in later life (“fetal programming”). Neurodevelopment is a dynamic process that involves neurogenesis, neuronal migration, cortical growth and gyrification, starting in early pregnancy and lasting until infancy (first 1,000 days). The pre- and periconceptional   period is an important window within this broader window because of “epigenetic reprogramming” of the conceptus which happens within 48–72h of conception. |  |
| **Potential Behavioural mediating factors (as identified by authors of the primary studies)** | Compliance to sprinkles was limited 57% | None given |  |
| **Potential Social mediating factors (as identified by authors of the primary studies)** | Household danamics referred to in discussion. | None given |  |
| **Details on who interventions are available to, and who is accessing services and in what numbers** | No information given except that there were no differences in baseline chacateristics except literacy. | The interventions were avaiable to the adolescent offspring of the PMNS trial conducted between 1993 to 1996. 690 screened at 17 y. 557 (of whom 266 were girls) were randomised. Of these 166 pregnancies & 149 live births were detected. 85 children were>2 y by March 2020 then followup had to stop due to covid.  The trial was not about access to government services but the supplements were delivered to the participants. |  |
| **Author reflections on factors affecting the success of preconception nutrition interventions, including factors relating to leadership, financing, governance, supplies, and capacity** | None as intervention was not successful. | None given |  |
| **Author reflections on factors affecting the failure of preconception nutrition interventions, including factors relating to leadership, financing, governance, supplies, and capacity** | A possible explanation for why maternal hemoglobin and iron stores were not significantly affected by IFA supplementation in pregnant women is that iron status was already fairly good at baseline (only 6% of were iron deficient (ferritin <12 lg/L). Placenta and fetus may be able to compete effectively for iron among mothers with good iron status i.e. the iron is 'channelled' to the fetus.  Perhaps IFA supplementation had no effect among pregnant women because of low adherence (57.7% +/-26.9%) and a relatively short period of supplementation. Pregnant women were in the study for an average of 136.3 +/- 36.6 fewer days and therefore consumed the contents of 94.5 +/- 54.5 fewer sachets compared with nonpregnant women.  It is possible that women were less compliant because they perceived less benefit from supplement use before pregnancy. Also they were nulliparous and young (50% <adolescents) so may not have had expsore to antenatal messages. Reduced iron absorption from the powdered mixture may have occurred because of high amounts of phytate in rice or from mixing the powdered micronutrients with liquids, causing some of the particles to be lost in the process because of sticking to a glass or bowl.  Uncertain whether adding a powdered form of micronutrients to their food was difficult in household or social situations in which food is shared. | None given |  |
| **Author recommendations on strategies to improve preconception nutrition in South Asia** | None - all research recommendations | The majority of pregnancies are unplanned, and women approach the healthcare system after this window. Our supplementation was specifically started in adolescence to ensure adequate micronutrient stores in the mother from before conception, in time to support gametogenesis, conception, embryogenesis, organogenesis, and placentation. The success of pre-conceptional folic acid supplementation in preventing NTDs is well-known. Thus, authors propose that the 1,000-day window should be expanded to include the preconception period. This would shift the action from the clinic to the community and will fit well into a multitude of adolescent and reproductive age programs across the world. Our findings have strong implications for public health policy to improve the vitamin B12 status of young adolescents and reproductive age women in populations with a sizable prevalence of vitamin B12 deficiency. Utility of this approach in non-vegetarian populations, needs to be documented. authors foresee benefits of such a policy to many national nutrition programmes in India. |  |
| **Author recommendations on future research to improve preconception nutrition in South Asia** | The effect of periconceptional iron supplementation on functional outcomes in both the mother and infant is an important area of future research and would benefit from investigation of the bi- ological pathways by which maternal iron status affects fetal metabolism and growth. Further studies on adherence in this age group need to examine preferences for modality of supplements in relation to socioeconomic and cultural factors.  Before large-scale periconceptional IFA supplementation programs are imple- mented, there is a need for effectiveness studies and research that examines the effect of periconceptional IFA supplementation on maternal and infant outcomes. .  Future studies would benefit from determining red blood cell folate concentrations, which are long- term indicators of folate status and can be related to reduction in risk of neural tube defects. It has yet to be determined whether periconceptional IFA supplementation is a cost-effective strategy for preventing and controlling anemia during pregnancy and whether it can be effectively implemented at the operational level. |  |  |
| **Barriers to Preconception nutritional intake** |  | Not mentioned |  |
| **Enablers to preconception nutritional intake** |  | Not mentioned |  |
| **Risk of Bias assessment ROB2 completed?** |  | 1 |  |
| **ABSTRACT** | Background: There is a growing interest in periconceptional iron supplementation in developing countries by researchers and policy makers; however, there are no randomized controlled trials that examine the effectiveness of this strategy in decreasing anemia during pregnancy. Objective: The aim was to determine whether periconceptional iron supplementation reduces anemia during pregnancy. Design: A randomized, double-blind, controlled trial was conducted in rural Bangladesh. Married, nulliparous women were randomly assigned to receive daily iron and folic acid (IFA; 60 mg ferrous fumarate and 400 lg folic acid) (n = 134) or folic acid (FA; 400 lg) (n = 138) in the form of a powdered supplement added to food. Women were followed until pregnancy or the end of 9 mo. Primary outcomes included hemoglobin, plasma ferritin, and plasma trans- ferrin receptor concentrations. Results: Among 88 pregnant women, periconceptional IFA in com- parison with FA did not affect anemia or iron status at 15 wk gestation. However, each 1% increase in adherence was associated with a 10-g/L increase in change in hemoglobin from baseline (P = 0.03), and those who initiated supplementation at a mean (6SD) time of 72.9 6 57.8 d before conception showed a 7.3-g/L increase in change in hemoglobin from baseline compared with those who initiated supplementation at 26.3 6 12.3 d after conception (P = 0.01). Among 146 nonpregnant women, IFA decreased anemia (odds ratio: 0.19; 95% CI: 0.04, 0.95) and improved iron stores (P = 0.001) more than did FA. Conclusion: Good adherence and initiation of supplementation be- fore conception are needed to reduce anemia during early pregnancy. This trial was registered at www.clinicaltrials.gov as NCT00953134. Am J Clin Nutr 2009;90:1295–302 | Background: The first thousand days window does not include the pre-conceptional period. Maternal pre-conceptional health has a profound influence on early embryonic development (implantation, gastrulation, placentation etc). Nutrition provided by B-complex vitamins is important for fetal growth, especially neural development. We report effects of a maternal pre-conceptional vitamin B12 and multi micronutrient (MMN) supplementation on offspring neurodevelopmental performance. Methods: In the Pune Rural Intervention in Young Adolescents trial (PRIYA), adolescents (N = 557, 266 females) were provided with vitamin B12 (2 μg/day) with or without multiple micronutrients, or a placebo, from preconception until delivery. All groups received mandatory iron and folic acid. We used the Bayley’s Scale of Infant Development (BSID-III) at 24–42 months of age to investigate effects on offspring neurodevelopment. Results: Participants had similar baseline B12 levels. The levels improved in the B12 supplemented groups during pre-conception and pregnancy (28 weeks gestation), and were reflected in higher cord blood holotranscobalamin (holo-TC) levels compared to the placebo group. Neurodevelopmental outcomes in the B12 alone group (n = 21) were better than the placebo (n = 27) in cognition (p = 0.044) and language (p = 0.020) domains (adjusted for maternal baseline B12 levels). There was no difference in neurodevelopmental outcomes between the B12 + MMN (n = 26) and placebo group. Cord blood Brain Derived Neurotrophic Factor (BDNF) levels were highest in the B12 alone group, though not significant. Conclusion: Pre-conceptional vitamin B12 supplementation improved maternal B12 status and offspring neurodevelopment at 2 years of age. The usefulness of cord BDNF as a marker of brain development needs further investigation. Our results highlight the importance of intervening during pre-conception. |  |

| **SN** | **7** | **8** | **9** |
| --- | --- | --- | --- |
| **Short name** | Hambidge2014 | Hambidge2019a | Hambidge2019b |
| **Type of paper** | Trial protocol | Primary analysis of trial data - main trial paper | Secondary analysis of trial data |
| **Authors** | K. Michael Hambidge, Nancy F Krebs, Jamie E Westcott, Ana Garces, Shivaprasad S Goudar, Balachandra S Kodkany, Omrana Pasha, Antoinette Tshefu, Carl L Bose, Lester Figueroa, Robert L Goldenberg, Richard J Derman, Jacob E Friedman, Daniel N Frank, Elizabeth M McClure, Kristen Stolka, Abhik Das, Marion Koso-Thomas, Shelly Sundberg and For the Preconception Trial Group | K Michael Hambidge, Jamie E Westcott, Ana Garcés, Lester Figueroa, Shivaprasad S Goudar, Sangappa M Dhaded, Omrana Pasha, Sumera A Ali, Antoinette Tshefu, Adrien Lokangaka, Richard J Derman, Robert L Goldenberg, Carl L Bose, Melissa Bauserman, Marion Koso-Thomas, Vanessa R Thorsten, Amaanti Sridhar, Kristen Stolka, Abhik Das, Elizabeth M McClure, and Nancy F Krebs, on behalf of the Women First Preconception Trial Study Group | K Michael Hambidge,, Carla M. Bann, Elizabeth M. McClure, Jamie E. Westcott, Ana Garcés, Lester Figueroa , Shivaprasad S. Goudar , Sangappa M. Dhaded, Omrana Pasha, Sumera A. Ali, Richard J. Derman, Robert L. Goldenberg, Marion Koso-Thomas, Manjunath S. Somannavar , Veena Herekar, Umber Khan and Nancy F. Krebs |
| **Year** | 2014 | 2019 | 2019 |
| **Title** | Preconception maternal nutrition: a multi-site randomized controlled trial. | A multicountry randomized controlled trial of comprehensive maternal nutrition supplementation initiated before conception: the Women First trial | Maternal Characteristics Affect Fetal Growth Response in the Women First Preconception Nutrition Trial |
| **Citation** | Hambidge, K. M., N. F. Krebs, J. E. Westcott, A. Garces, S. S. Goudar, B. S. Kodkany, O. Pasha, A. Tshefu, C. L. Bose, L. Figueroa, R. L. Goldenberg, R. J. Derman, J. E. Friedman, D. N. Frank, E. M. McClure, K. Stolka, A. Das, M. Koso-Thomas, S. Sundberg and G. the Preconception Trial (2014). "Preconception maternal nutrition: a multi-site randomized controlled trial." BMC Pregnancy and Childbirth 14(1): 111. | Hambidge KM, Westcott JE, Garcés A, Figueroa L, Goudar SS, Dhaded SM, Pasha O, Ali SA, Tshefu A, Lokangaka A, Derman RJ, Goldenberg RL, Bose CL, Bauserman M, Koso-Thomas M, Thorsten VR, Sridhar A, Stolka K, Das A, McClure EM, Krebs NF; Women First Preconception Trial Study Group. A multicountry randomized controlled trial of comprehensive maternal nutrition supplementation initiated before conception: the Women First trial. Am J Clin Nutr. 2019 Feb 1;109(2):457-469. | Hambidge, K Michael, Carla M. Bann, Elizabeth M. McClure, Jamie E. Westcott, Ana Garcés, Lester Figueroa, Shivaprasad S. Goudar, Sangappa M. Dhaded, Omrana Pasha, Sumera A. Ali, and et al. 2019. "Maternal Characteristics Affect Fetal Growth Response in the Women First Preconception Nutrition Trial" Nutrients 11, no. 10: 2534. |
| **doi** | 10.1186/1471-2393-14-111 | doi: 10.1093/ajcn/nqy228 | doi:10.3390/nu11102534 |
| **Clinical trials registration no** | ClinicalTrials.gov NCT01883193. | https://clinicaltrials.gov/study/NCT01883193 | https://clinicaltrials.gov/study/NCT01883193 |
| **Context / setting** | Rural sites in four countries in India (Belgaum, Karnataka), Pakistan (Thatta, Sind Province), Democratic Republic of Congo (DRC, Equateur Province), and Guatemala (Chimaltenango Department), | Rural or semirural locations in the Democratic Republic of the Congo (DRC; Equateur), Guatemala (Chimaltenango), India (Belagavi, North Karnataka), and Pakistan (Thatta, Sindh Province). | Low-resource, small-town, rural communities in Chimaltenango, Guatemala, Thatta, Sindh Province, Pakistan, and Belagavi, Karnataka, India. (Not DRC (Democratic Repubic of Congo)) |
| **Protocol citation** | this is the protocol | Hambidge KM, Krebs NF, Westcott JE, Garces A, Goudar SS, Kodkany BS, Pasha O, Tshefu A, Bose CL, Figueroa L, Goldenberg RL. Preconception maternal nutrition: a multi-site randomized controlled trial. BMC pregnancy and childbirth. 2014 Dec;14:1-6. | Hambidge KM, Krebs NF, Westcott JE, Garces A, Goudar SS, Kodkany BS, Pasha O, Tshefu A, Bose CL, Figueroa L, Goldenberg RL. Preconception maternal nutrition: a multi-site randomized controlled trial. BMC pregnancy and childbirth. 2014 Dec;14:1-6. |
| **Arising from which main trial** | The Women First Randomized Controlled Trial | The Women First Randomized Controlled Trial | The Women First Randomized Controlled Trial |
| **Study design** | 3-arm individually randomized, longitudinal, nonmasked (non-blinded), multisite controlled efficacy trial | 3-arm individually randomized, nonmasked, multisite controlled efficacy trial | 3-arm individually randomized, nonmasked, multisite controlled efficacy trial |
| **Has Preconception intervention only arm (1/0)** | 0 | 0 | 0 |
| **Has Preconception + Pregnancy intervention arm (1/0)** | 1 | 1 | 1 |
| **Has Pregnancy intervention only arm (1/0)** | 1 | 1 | 1 |
| **Has Pregnancy, postpartum + childhood intervention arm (1/0)** | 0 | 9 | 0 |
| **Has Control arm with only usual standard of care or less intensive intervention in preconception & pregnancy (1/0)** | 1 | 1 | 1 |
| **MMN in Preconception arm** |  |  |  |
| **B12 in Preconception arm** |  |  |  |
| **LNS in Preconception arm** | 1 | 1 | 1 |
| **Viatmin A in preconception arm** |  |  |  |
| **Iron in preconception arm (both arms had Folic acid)** |  |  |  |
| **Nutritious snack in Preconception arm** |  |  |  |
| **Pregnancy outcomes** |  | 1 |  |
| **Birth outcomes** |  |  | 1 |
| **Infancy outcomes** |  |  |  |
| **Child outcomes** |  |  |  |
| **Narrative summary of main finding** | Protocol so no findings | the intervention initiated before conception or late in the first trimester resulted in greater mean birth size (LAZ, WAZ, WLRAZ) and improved rates of stunting, underweight, wasting (WLRAZ < −2), and SGA in comparison with the control arm. Across all sites (DRC, Guatemala, Pakistan and India of the Women 1st trial. LNS in preconception + pregnancy versus LNS started in pregnancy alone showed no difference in mean LAZ or other outcomes using either NGAA or GAA. Comparing preconeption & pregnancy with control: Mean LAZ (NGAA) for Arm 1 was greater than for Arm 3. For GAA outcomes, rates of stunting and small-for-gestational-age were lower in Arm 1 than in Arm 3. Effect sizes were similar for LNS in pregnancy alone on LAZ and WAZ. Rates of preterm birth did not differ among arms. Pairwise comparisons of treatment arms within an individual site used α=0.00625 cut-off. Mean length-for-age z score for Arm 1 differed from Arm 3 in Pak (P = 0.0057). | In 3 sites of the Women First Trial (Guatemala, Pakistan and India) the effect of LNS in preconception + pregnancy versus LNS started in pregnancy alone was modified by nulliparity and anaemia. Maternal nulliparity and anemia were associated with impaired fetal growth that was substantially improved by nutrition intervention, especially when commenced prior to conception. |
| **Date of data collection** | Timeline (in months, commencing December 1, 2012) 0-12 Study preparations 13-18 Enrollment of 1440 eligible participants and baseline studies 38 Conception of 50% participants in each arm complete 47 All pregnancies completed 53 Maternal/infant follow-up completed (May 30, 2017) 60 Laboratory analyses, data analyses, reporting complete (Nov 30, 2017) | Screening: December, 2013 and October, 2014. Dates of followup or start and end of interventions not provided. |  |
| **Study objectives** | Goal: to ascertain the benefits to the offspring of ensuring optimal human maternal nutrition before conception, during the peri-conceptional period and during the entire first trimester. Objective: to determine the benefits to the off- spring of women in poor, food-insecure environments of commencing a daily comprehensive maternal nutrition supplement (with additional balanced calorie/protein supplement for underweight participants) ≥ 3 mo prior to conception versus the benefits of commencing the same supplement at 12-14 wk gestation and also to compare offspring outcomes with those of a third trial arm who receive no supplement. | This study tested the effects on newborn size, especially length, of commencing nutrition supplements for women in low- resource populations ≥3 mo before conception (Arm 1), compared with the same supplement commenced late in the first trimester of pregnancy (Arm 2) or not at all (control Arm 3). |  |
| **Sample size / power** | Sample size determined to test the primary hypoth-esis based on the primary outcome of LAZ at birth with 80% power at each individual site, while maintaining an overall trial-wise Type I error rate of 0.05 across all planned primary hypothesis tests (two tests comparing Arm1 vs Arm2 and Arm1vs Arm3 at each of four sites). Thus an alpha level of 0.00625 was specified to account for the 8 planned primary comparisons. Assuming an alpha level of 0.00625, a 2-sided test, and a standard deviation of 1.0 for the primary outcome, LAZ, 192 evaluable women per arm at each site will allow detection of an effect size of 0.37 with 80% power. Accounting for 20% attrition during pregnancy requires that 240 women per arm enter Stage 2 (concpetion) at each site, and assuming that 50% of women randomized at Stage 1 will get pregnant and move to Stage 2 requires 480 women per arm enrolled at each site. Keeping the number entering Stage 2 at 240 per arm, if attrition during pregnancy is 10% in stead of 20%, 216 evaluable women per arm will allow detection of an effect size of 0.35. If authors consider only the Arm 1 vs Arm 2 comparison as primary, given 192 evaluable women per arm at each site for a total of 768 women per arm over all four sites, setting an alpha level of 0.025 for each primary hypothesis test will allow detection of an effect size of 0.18 with 90% power or an effect size of 0.20 with 95% power for each overall comparison. | Sample size determination was based on testing 2 co-primary hypotheses (comparison of Arm 1 with Arm 2 and Arm 1 with Arm 3), for the primary outcome of LAZ at birth. The sample size was based on also having 80% power within each site and maintaining a study-wide Type I error rate of 0.05 across all planned primary hypothesis tests (2 tests at each of 4 sites, for a total of 8 tests). Thus, an α-level of 0.00625 for single-site outcomes was specified to account for the 8 planned primary comparisons. Assuming an α-level of 0.00625, a 2-sided test, and an SD of 1.0 for the primary outcome, 192 evaluable women per arm in each site were needed to detect an effect size of 0.37 with 80% power. To account for 20% attrition during pregnancy required that 240 women per arm enter Phase 2 (pregnancy) within each site. The assumption that 50% of women randomly assigned at Phase 1 would get pregnant and move to Phase 2 required 480 women per arm to be enrolled in each site. Given 192 evaluable women per arm in each site for a total of 768 women per arm over all 4 sites, an α-level of 0.025 for each primary hypothesis test across all sites would allow detection of an effect size of 0.18 with 90% power for each pooled comparison of Arm 1 with Arm 2 and Arm 1 with Arm 3. |  |
| **Sample characteristics** |  |  |  |
| **Randomisation** | Allocation sequence generated separately for each site by RTI International. A per-muted block design with stratification by geographic cluster was used to generate the randomization sequence for assigning individual participants to a trial arm. The num ber of assignments to Arms 1, 2, and 3 were allocated with a ratio of 1:1:1 within blocks randomly varied in size between 3, 6, and 9. Stratification by cluster was used to ensure a similar number of participants randomized to each arm within cluster in order to provide geographic diversity across arms and for operational convenience given that the trial infrastructure in each country is organized around these clusters. | The DCC created the randomization scheme, centrally gener- ating the allocation sequence for each site. To ensure geographic balance, a permuted block design stratified by GN clusters was used for assigning individual participants to a trial arm. The allocation ratio was 1:1:1 within blocks which randomly varied between sizes of 3, 6, or 9 for each site. Once the responsible home visitor research assistant identified an eligible participant, they received the random assignment generated by the site data manager from the centralized computerized data management system maintained by the DCC. |  |
| **Blinding** | Non-masked/ Not blinded | Not masked. No mention of blinding at the analytical stage is mentioned |  |
| **Age of participants (years)** | 16-35 y | 16 to 35 y | 16 to 35 y |
| **Inclusion criteria** | 16-35 y of age; parity 0-5; expectation to have first or additional pregnancy within next 2 y and without intent to utilize contraception. | age 16–35 y; parity 0–5, with site- specific strategies to include nulliparous participants; no current or planned contraceptive use; and expectation to conceive during the following 18 mo. Consent of parous women was delayed until ≥2 mo postpartum. | Women of childbearing age (16–35 years) who were all expecting to conceive within the following eighteen months and had screening hemoglobin (Hb) >8 g/dL. Apart from the screening Hb, participating women were not selected with respect to their long- or shorter-term nutritional status as determined by stature and BMI. Included in the current analysis were the maternal–newborn dyads who had gestational age determined from crown-rump length measurements of gestational age in the first trimester and had newborn anthropometric measures completed within 48 h of delivery [26] Maternal characteristics evaluated were maternal stature, BMI, anemia status at baseline (defined as Hb <12 g/dL), parity, age, SES, and education. Newborn sex was also included in the regression analysis. Newborn outcome measures were length-for-age z-score (LAZ), weight-for-age z-score (WAZ), and weight to length ratio-for-age z-score (WLRAZ) based on INTERGROWTH-21st fetal growth standards [32,33]. |
| **Exclusion criteria** | Women with hemoglobin (Hb) ≤ 8 g/dL and nulliparous women who do not agree to hospital delivery (equipped for caesarian section) or/and do not have ready access to such a facility.  Parous women who have had a previous history of pre-eclampsia or a history of prolonged labor associated with cephalopelvic disproportion will be excluded. | Women with a known history of obstetric complications or those who were unwilling to deliver in hospital were excluded.  If an otherwise eligible woman had a hemoglobin concentration of ≤8 g/dL at screening, enrollment was delayed until successfully treated, if at all |  |
| **Preconception intervention characteristics** | A multi- micronutrient (MMN) fortified lipid-based supplement (Nutriset, Malauney, France) with MMN and polyunsaturated lipids (linoleic 4.9 g and α-linolenic 0.59 g), the composition includes dried skimmed milk, soybean and peanut extract, sugar, maltodextrin stabilizers, and emulsifiers. Modification of Nutributter by iLiNS based at UC Davis for research use with pregnant and lactating women (LNS P&L).  Initiated within 2 wk of enrollment which is: at least 4 mo before the estimated conception for nulliparous women; 4 to 8 mo postpartum depending on site-specific inter-pregnancy intervals  Supplementation is terminated at delivery.  Potential participants excluded if they become pregnant within 3 months of starting biweekly followup. Monthly weight checks for Arm 1 partici-pants identify those with BMI ≤ 20 and those participants will receive an additional balanced protein/energy (300 kcal/d; 12% calories from protein and no added micronutrients; Nutriset, Maluaney, France) supplement, and weight will be monitored monthly. Once initiated, the second supplement will be continued until the end of the trial or the completion of pregnancy, whichever is the soonest.  Participants cautioned not to take other micronutrient supplements or fortified supplemental foods while on the trial intervention. | 20 g lipid-based micronutrient supplement - LNS- (Nutriset) and provided micronutrients, polyunsaturated fats in a favorable balance, and modest quantities of protein and energy (2.6 g protein and 118 kcal) (25, 28). For Arm 1, the duration of the primary supplement was from the time of random assignment until delivery; participants were required to be on the primary supplement for ≥3 mo before conception.  In addition, in Arms 1 and 2, women were provided a second daily lipid-based protein-energy supplement (termed Supplement 2, Supplemental Table 1) if they had a BMI <20 at any time while receiving Supplement 1, or had weight gain in the second or third trimesters of pregnancy less than the Institute of Medicine’s guidelines. If consumed completely, this supplement provided 300 kcal and 11 g protein (∼15% of energy) without additional supplemental micronutrients (Nutriset). Supplement 2 was initiated before conception on the basis of BMI for participants in Arm 1 as well as for gestational weight gain during pregnancy (1.7–2.0 kg/mo). Unlike the primary supplement, high compliance was not required for the second supplement because of our intention to minimize reduction in habitual food intake and for participants to consume a quantity “to appetite.” Recipients were encouraged to consume ≥50% of the protein-energy supplement (Supplement 2) on a daily basis. Participants cautioned not to take other micronutrient supplements or fortified food products while taking the trial supplements. | In this trial, participants were randomized to one of three arms. Arm 1 commenced the nutrition intervention at least 3 months prior to conception; |
| **Preconception control group intervention** | No intervention preconceptionally in control or in arm 2 | Participants in Arm 3 were not provided any nutrition supplement by the study. | Arm 3 (control) received no trial supplement. |
| **Intervention timing (how far before conception)** | >=3 months i.e. at least 3 mo before pregnancy exposure to be included in the study | >=3 months | >=3 months |
| **Intervention delivery (health systems, food systems, social protection systems)** | Project staff deliver the LNS at home. NOT delivered through the health system. Fourteen daily supplements will be provided biweekly in 20 g sachets with instructions to take one sachet per day. A rodent and water resistant plastic container will be provided to each participant for storage of the sachets. | Project staff - home visitor research assistants - delivered supplements to home every 2 weeks. |  |
| **Pregnancy detection** | Biweekly home visits and assessment for all three arms (more frequently for first 2 wk or for longer period if issues with compliance) will commence within 2 wk of enrollment. Menstrual history will be obtained at each visit and a urine pregnancy test will be performed if last menstrual period (LMP) was > 4 wk prior or mother thinks she might be pregnant. Pregnancy testing will also be undertaken at every visit after the first 3 mo postpar- tum if menses have not yet resumed or are irregular. | Participants in all 3 arms were visited by the home visitor research assistants every 2 wk to record interim health history and to administer a urine pregnancy test. The pregnancy testing was combined with calendar records of menses to ascertain last menstrual period and to guide the timing of ultrasounds to be obtained between 10 and 12 weeks of estimated gestation. |  |
| **Pregnancy intervention characteristics (if any)** | A multi- micronutrient (MMN) fortified lipid-based supplement (Nutriset, Malauney, France) with MMN and polyunsaturated lipids (linoleic 4.9 g and α-linolenic 0.59 g), the composition includes dried skimmed milk, soybean and peanut extract, sugar, maltodextrin stabi- lizers, and emulsifiers. Modification of Nutributter by iLiNS based at UC Davis for research use with pregnant and lactating women (LNS P&L). Starts at 12 wk gestation after dietary assessments,, anthro pometry measurements and biospecimen collection, and ultrasound exams. Supplementation is terminated at delivery. Monthly weight checks will continue in pregnancy for Arm 1 and be initiated for Arm 2 at the time the nutrition supplement is started. Extra energy/protein supplement (see above) will be provided for women in Arms 1 and 2 if 2nd and 3rd trimester pregnancy weight gain fails to reach recommended guidelines (approximately 2 kg/mo for underweight and normal weight women and 1 kg/mo for overweight and obese women). Participants in Arm 2 will be advised to stop taking iron and folate supplements once they start the nutrition intervention at 12 wk gestation. | For Arm 2, the primary supplement covered the second and third trimesters of pregnancy and was stopped at delivery. In addition, in Arms 1 and 2, women were provided a second daily lipid-based protein-energy supplement (termed Supplement 2, Supplemental Table 1) if they had a BMI <20 at any time while receiving Supplement 1, or had weight gain in the second or third trimesters of pregnancy less than the Institute of Medicine’s guidelines (29). If consumed completely, this supplement pro- vided 300 kcal and 11 g protein (∼15% of energy) without additional supplemental micronutrients (Nutriset). For Arm 2, Supplement 2 was started after the initiation of Supplement 1 when either of the criteria became evident. Once initiated, Supplement 2 was provided until delivery. High compliance was not required for the second supplement because of our intention to minimize reduction in habitual food intake and for participants to consume a quantity “to appetite.” Recipients were encouraged to consume ≥50% of the protein-energy supplement (Supplement 2) on a daily basis.  Participants cautioned not to take other micronutrient supplements or fortified food products while taking the trial supplements. | Arm 2 received the same intervention commencing at the end of the first trimester; |
| **Pregnancy control group intervention (if any)** | Participants in all arms will be advised at 12-14 wk gestation to commence prenatal care, which, in the case of Arm 3, may include supplements provided by caregiver. |  | Arm 3 (control) received no trial supplement. |
| **Interventions after birth (if any)** |  |  |  |
| **Control group intervention after birth (if any)** |  |  |  |
| **Compliance measurement** | Compliance of supplement use will be monitored by self-reported history and sachet collection during biweekly visits. Women will also mark off daily supplement compli- ance on the calendar noted above. Compliance will also include random independent audits by other research personnel. | Compliance with use of supplements was documented by inspection of calendars the women completed daily and by collection of empty, partially eaten, and unused intervention sachets. Compliance was calculated for Supplement 1 as the total number of sachets fully eaten divided by the number of days between starting Supplement 1 and delivery. Supplement 2 compliance was calculated similarlyexcept numerator was the total number of Supplement 2 sachets fully or partially eaten. The mean ± SD length of exposure for Supplement 1 for Arm 1 during the preconception period was 37.3 ± 21.5 wk, with mean compliance of 88% (i.e., for every 100 d of exposure women consumed 88 sachets). During the first 12 wk of pregnancy, compliance for this group was similar at 87.3% ± 16.1%. From 12 wk to delivery, exposure for Arm 1 was 27.2 ± 1.9 wk and compliance was 84.2% ± 17.4%. Total length of exposure for Arm 1 from enrollment to delivery was 76.6 ± 21.6 wk with overall compliance of 87.2% ± 13.2%. For Arm 2, total length of exposure for Supplement 1 was 25.4 ± 3.2 wk, and compliance was 84.3% ± 17.4%. Supplement 2, the protein-energy supplement, was started in >90% of the women in Arm 1 in DRC, India, and Pakistan, and in 88–96% of the women in these sites for Arm 2 (after 12 weeks of gestation). Less than 10% of the women in Guatemala for either Arm 1 or Arm 2 started Supplement 2. Mean overall Supplement 2 compliance for both arms was 84%, with total duration of exposure 55.4 ± 29.4 and 22.0 ± 5.8 wk for Arms 1 and 2, respectively. |  |
| **Adverse events** | Adverse events monitored continuously by recording any of the following situations: - Any fatal or life threatening event occurs to the participating woman, fetus or infant, or - Any serious and unexpected adverse event occurs Specific adverse events: early termination of pregnancy, adverse pregnancy outcome, adverse neonatal event, hospitalization (mother or child) due to acute illness, allergic reactions, diarrhea, and vomiting. All such events, including other unexpected events will trigger completion of the adverse events form, and will be reported to the site PI, the Data Coordinating Center and the overall trial PI within 48 h for all deaths and within 7 d for other adverse events. No stopping rule. | Adverse events were monitored continuously as per protocol and reported to the overall study principal investigators and the DCC within 48 h for all deaths and within 7 d for other adverse events, including adverse pregnancy outcomes, adverse neonatal events, hospitalizations, and allergic reactions. A Data Monitoring Committee reviewed the study progress for safety, trial progress, data completion, supplement compliance, and protocol violations twice yearly |  |
| **Engagement, category (including nutrition sensitive/specific)** | Nutrition specific | Nutrition specific |  |
| **Where in the overall UNICEF 2020 framework the intervention lies** | Intervention changing an the immediate determinant of Diet: LNS supplement is directly altering altering diet | Intervention changing an the immediate determinant of Diet: LNS supplement is directly altering altering diet | Intervention changing an the immediate determinant of Diet: LNS supplement is directly altering altering diet |
| **Where in the Partap 2021 framework the intervention lies** | LNS supplement is an individual-level nutrition intervention. | LNS supplement is an individual-level nutrition intervention. | LNS supplement is an individual-level nutrition intervention. |
| **Proposed mechanism of intervention action** | Maternal environmentally-induced improvement in her epigenome provide an appealing hypothesized mechanism by which this preconception nutrition intervention is responsible, at least in part, for the hypothesized resulting phenotypic improvements in her offspring including pre- and post-natal growth. In particular it is hypothesized that DNA methyla- tion patterns in global and gene specific studies will vary with a maternal nutrition intervention commencing pre-conception vs. 12-14 wk gestation vs. no maternal nutrition supplement.  Chronic inflammation and oxidative stress may alter pregnancy outcomes, including the fetal and postnatal growth potential, nutrient utilization and nutritional status, and immunologic development and function of mother and of the offspring. The proposed LNS intervention, through anti-inflammatory and antioxidant features, may beneficially alter the maternal metabolic profile, especially for those who initiate the LNS preconception. | See protocol? |  |
| **Exposures relating to preconception nutrition** | A fortified lipid-based supplement (LNS) (Nutriset, Malauney, France) with MMN and polyunsaturated lipids (linoleic 4.9 g and α-linolenic 0.59 g), the composition includes dried skimmed milk, soybean and peanut extract, sugar, maltodextrin stabilizers, and emulsifiers started >3 months before conception. | A fortified lipid-based supplement (LNS) (Nutriset, Malauney, France) with MMN and polyunsaturated lipids (linoleic 4.9 g and α-linolenic 0.59 g), the composition includes dried skimmed milk, soybean and peanut extract, sugar, maltodextrin stabilizers, and emulsifiers started >3 months before conception. | A fortified lipid-based supplement (LNS) (Nutriset, Malauney, France) with MMN and polyunsaturated lipids (linoleic 4.9 g and α-linolenic 0.59 g), the composition includes dried skimmed milk, soybean and peanut extract, sugar, maltodextrin stabilizers, and emulsifiers started >3 months before conception. |
| **Preconception measures** | Mother's past medical and obstetric history; height and weight, body mass index (BMI); mid-upper arm circumference (MUAC); waist and hip circumference; and head circumference.  0.5 mL venous or fingerstick blood sample in all three trial arms for Hb and dried blood spot collections. A pregnancy test to ensure that the participant is not pregnant. Paternal height and weight at baseline whenever possible. Women in Arm 1 will be weighed monthly and BMI calculated by the Field Supervisor. |  |  |
| **Concommittant care** | Not mentioned | Not mentioned | Not mentioned |
| **Follow-up schedule for measurements** | Stage 1. Enrolment in prepregnancy Demographic information (including SES, water supply, sanitation, parental education and occupation); site-specific household food insecurity questionnaire and indoor air pollution assessment. Collectedi n the home by the local HVRA within 1 wk of enrollment. 2-weekly chekup visits by a HVRA throughout the study  Stage 2 from conception onwards Detection of pregnancy 12 and 34 weeks gestation - maternal blood, stool, urine, fetal growth measures, fetal issues but also 2-weekly checks of morbidity and MUAC in arm 3, & monthly weight for Arms 1 and 2  Delivery within 12 hours: newborn anthropmetry, vital status, cord blood and Maternal blood, stool sample and vaginal swab Assessments at 14-d, 1, 3, and 6 mo of age to collect neonatal and infant anthropometry 14 days and 3 months pospartum maternal and infant blood, maternal stool, urine, - breastmilk composition,  6 months infant followup morbidity,  HVRAs will also check on details of infant feeding at each visit, supporting exclusive breast feeding; | Women were identified through the Eunice Kennedy Shriver National Institute of Child Health and Human Development Global Network (GN) Maternal and Newborn Health Registry, household surveys, local health centers, word-of-mouth, and local advertising. Maternal height and weight measurements were obtained at enrollment, and maternal weight was obtained at ∼12 and 32 wks of gestation for all arms Maternal monthly weight also obtained once Supplement 1 was initiated (e.g., at enrollment for Arm 1 and after the 12 wk gestation measurement for Arm 2).  First trimester ultrasound crown-rump length (CRL) measure- ments were obtained for participants at 3 of the sites, allowing for newborn anthropometry to be adjusted for gestational age (GA). Newborn anthropometry was obtained within 48 h of delivery (neonatal stadiometer, Ellard Instrumentation, Ltd; seca 334 electronic scale and seca 201 measurement tape, seca North America). Infant recumbent length and weight measurements were obtained in triplicate and entered into the database; the median value was used for analysis. |  |
| **Summary of baseline comparisons by study arm** | Not reported in protocol | The only baseline difference between those with a primary outcome and the entire randomly assigned group was for parity: 20.4% and 27.5% were nulliparous, respectively. Overall baseline characteristics by arm among women who had the primary outcome obtained differed only in terms of maternal education, with a higher percentage of women in Arm 1 having no formal education (P = 0.0081). |  |
| **Pregnancy outcomes: gestational weight gain, gestational diabetes, maternal anthropometry (weight, MUAC, height), anaemia,** | biweekly visits monitoring maternal infectious disease morbidity and MUAC in all arms. monthly weight checks in arms 1 and 2. Pregnancy complications recorded as adverse eventS: miscarriages and still births, with estimation of gestational age of latter and weight when feasible 2. Longitudinal fetal growth from ultrasound measurements at 12 and 34-36 wk of crown-rump, femur and humeral length. Biparietal diameter and abdominal circumference also measured. 6. Epigenome from DNA of: - Mother from maternal blood samples at 12 and 34 wk gestation  - Fetus from fetal tissue (amnion). 8. Microbiome - maternal intestinal microbiome will be evaluated at 12 and 34-36 wk gestation using a partial stool sample & next-generation DNA sequencing assays. Biological samples (blood, urine, fecal) additional samples will be collected at 12 and 34-36 wk gestation in Arms 1 and 2.   50% of women in Arm 1 and Arm 2 have a dietary assessment at the biweekly visit when pregnancy is confirmed. A research nutritionist will obtain two multiple-pass 24-h dietary recalls (2-4 wk apart) from every other participant in Arms 1 and 2 prior to 12 wk gestation (i.e. 120/arm, 240/site). |  |  |
| **Preconceptual outcomes: (BMI, Hb or other outcomes which could be attributed to preconception care)** |  |  |  |
| **Birth outcomes in child: birthweight, low birth weight (LBW), small for gestational age (SGA), preterm birth/ delivery (PTB), cord blood b12, DXA,** | Primary outcome: **LAZ at birth** 1. Early neonatal length measurements at 12 h of age (neonatal stadiometer, Ellard Instrumenta- tion, Ltd, Monroe, WA). Also at Birth : - infant weight (electronic scales sensitive to 10 g),  - head circumference (plasticized measuring tape accurate to 1 mm), and  - MUAC*  * dataconverted to Z-scores automatically by the computer on data entry. 3. Mean birth weight and incidence of LBW recorded at delivery. 4. Perinatal mortality: Pregnancy losses >20 wk gestation (stillbirths), intrapartum losses, maternal ‘near deaths’ and neonatal deaths up to 1 mo of age as well as early infancy deaths from 1-6 mo of age. Verbal autopsy results for all neonatal and early infant death.  6. Epigenome of infant DNA from cord blood & maternal blood samples at delivery. 8. Microbiome - maternal intestinal microbiome will be evaluated at delivery. A partial stool sample with the addition of a vaginal swab.  Biological samples (blood, urine, fecal) will be collected from all maternal participants who consent at delivery. | The primary outcome, newborn length-for-age z score (LAZ), was based on length measurements obtained by the assessment teams before 48 h of age.  Secondary outcomes reported here by arm and site include  weight, head circumference (HC), and BMI, and the respective z scores: weight-for-age (WAZ),  HC-for-age (HCAZ), and  BMI-for-age (BMIAZ) z score Gestational-age-adjusted (GAA) outcomes were determined based on INTERGROWTH-21st fetal growth charts. Further outcomes included:  instead of BMIAZ, weight to length ratio-for-age z score (WLRAZ), proportions of infants with z scores < −1 and < −2 of GAA adjusted LAZ, WAZ, and HCAZ, WLRAZ low birth weight (LBW, <2500 g),  small-for- gestational age (SGA), and  preterm birth (PTB). |  |
| **Outcomes in mother at birth** | 6. Maternal blood samples for DNA (Epigenome) 2 wk and 3 mo postpartum.  Biological samples (blood, urine, fecal) will be collected from all maternal participants who consent at 14 d and 3 mo postpartum. |  |  |
| **Post-natal outcomes (in Infancy)** | 2. Infant LAZ at age 0.5, 1, 3 and 6 mo Infant vaccination history at 6 mo of age. 5. Incidence of severe neonatal and infant infectious disease (no. of acute visits/ admissions to health center/hospital for severe infectious disease). Diagnosis and treatment. Minor morbidity data.  6. Epigenome of mother and offspring DNA from:  - Infant finger stick blood will be collected at 2 wk and 3 mo. - Maternal blood samples at 2 wk and 3 mo postpartum. 7. Deep phenotyping of metabolic and nutritional status:  - Maternal systemic and/or gastrointestinal inflammatory markers: hsCRP, AGP, cytokines (IL-6, IL-8, IL-10, TNF-α, IFN-γ); urine and fecal neopterin, and fecal calprotectin, alpha-1-antitrypsin and myeloperoxidase. - Maternal oxidant status -oxidized-LDL and 4-HNE (4-hydroxynonenal, produced during oxidative stress and subsequent lipid peroxidation of polyunsaturated fatty acids). - Maternal endocrinologic milieu will be assessed by biomarkers of placental function: placental growth hormone; indices of iodine and energy utilization: thyroid hormone assays (TSH and Reverse T3), serum glucose, insulin (and calculated HOMA-IR: (fasting insulin [mLU/L]× fasting glucose (mmol/l]/22/5)); leptin and adiponectin [35]; and maternal stress (“allostatic load”): serum cortico- trophin releasing hormone (CRH). - Maternal nutritional status biomarkers: iron and zinc status (serum ferritin, AGP, soluble transferrin receptor, zinc); essential fatty acid profile; Vitamin B12 and methylmalonic acid; 25-hydroxy Vitamin D; RBC folate; retinol; pyridoxal phosphate,  8. Microbiome - Partial fecal samples will be collected at 14 d and 3 mo to characterize the infant microbiome and compare with mother's. Infant stool samples will be analyzed for biomarkers of gut inflammation including calprotectin, neopterin, alpha-1-antitrypsin and myeloperoxidase. 9. Composition of breast milk: potential growth factors, inflammatory mediators, hormones and adipokines, markers of maternal oxidant stress and antioxidant capacity of milk, all of which may directly or indirectly influence infant growth and development. |  |  |
| **Outcomes in childhood after 12 m: z scores for weight (WAZ), length/height (HAZ), and BMI for age (BMIZ), weight-for-height'length (WHZ), underweight, stunting, wasting, overweight, cognitive** | None |  |  |
| **Long term outcomes** | None in protocol |  |  |
| **Analysis** | Designed to formally test mean differences in the primary outcome of birth LAZ between women randomized to receive a daily nutrition supplement be- ginning 3 or more months prior to conception (Arm 1) and women randomized to receive the same daily nutrition supplement beginning at 12 wk gestation (Arm 2), and between Arm 1 and a control group of women (Arm 3) who will not receive the nutrition supplement. Analyses conducted separately by research site. Secondary analysis will compare outcomes across all four sites using the combined data. Following an intent- to-treat approach.. The primary analysis will compare mean LAZ between groups using a linear model with LAZ as the outcome and intervention group as the primary predictor to test the two primary hypoth- eses, namely that: (1) mean LAZ at birth differs between Arm 1 and Arm 2, and (2) mean LAZ at birth differs be tween Arm 1 and Arm 3. First, the two degree of freedom F-test will be used to test the hypothesis that the mean LAZ in at least one arm differs from the other two versus the null hypothesis that all three groups have equal mean LAZ. If that test is significant at the 0.0125 level then the two primary hypotheses will be tested at the 0.00625 level. Secondary analyses using multivariable re- gression models may adjust for the randomization cluster and any other critical covariates or confounders that may be imbalanced across the treatment groups. The same modeling approach will be used to evaluate the trial hypotheses over all sites combined, while controlling for research site. | For the primary and secondary outcomes, newborn LAZ, WAZ, HCAZ, and BMIZ were based on the WHO Child Growth Standards, which account for infant sex and age at measurement but are not adjusted for GA at delivery. Owing to the lack of GA determinations in the DRC, authors applied these standards to all births in all sites in the same manner across the 3 arms. Rates of LBW were also determined for the 4- site data set. In addition, for the 3 sites with GA determination (Guatemala, India, Pakistan), the INTERGROWTH-21st fetal growth standards were also applied to birth measurements and binary outcomes (31). These GAA analyses are a post hoc exploration, with P values provided for descriptive purposes. authors assessed the study outcomes using a modified intention- to-treat approach. The overall treatment effect and pairwise comparisons for the primary outcome and continuous secondary outcomes were obtained from linear models for the outcome of interest. Model-generated measures of effect size with 95% CIs and P values were adjusted for site and cluster-nested within site. For binary secondary outcomes, generalized linear models with generalized estimating equations were utilized to calculate RRs with 95% CIs and P values after adjusting for site while controlling for cluster correlations. The comparisons of Arm 1 with Arm 2 and Arm 1 with Arm 3 were prespecified in the protocol. The comparison of Arm 2 with Arm 3, also presented in this article, is a post hoc comparison. P values from chi-square tests for categorical variables and ANOVA analysis of means were calculated to assess differences between maternal baseline characteristics by treatment arm. In addition, for the primary outcome, authors investigated potential confounding by baseline maternal factors by first assessing differences in the maternal factors by treatment arm and then adjusting the aforementioned models for any factor which varied by treatment at an α-level of 0.10. These maternal factors included age, parity, education, BMI, height, and socioeconomic status. As a statistical check to ensure that evaluating only a subset of those randomly assigned (women who became pregnant and delivered a live birth, with birth length evaluated) was not unexpectedly skewing the results, authors constructed a composite binary secondary outcome that was evaluated alongside the primary outcome. Among the randomly assigned women who became pregnant, this outcome is defined as live birth free of growth failure. Specifically, the outcome compared women who delivered a live birth with LAZ ≥−1 to all other women who became pregnant (i.e., delivered a live birth with LAZ <−1 and women who did not deliver a live birth due to medical termination of pregnancy, miscarriage, stillbirth, and intrapartum death). Women without birth outcome data, including women (all 3 arms) who became pregnant too soon (i.e., enrolled in the study for <3 mo before conception), were excluded from this analysis. For this composite outcome, P values for the overall treatment effect and pairwise comparisons were obtained from a generalized estimating equation model adjusting for country and controlling for cluster correlations. |  |
| **EFFECTS on Preconception outcomes: maternal anthropometry (weight, MUAC, height. BMI), Haemoglobin (Hb), anaemia, iodine status** |  |  |  |
| **EFFECTS on Pregnancy outcomes: gestational weigt gain, gestational diabetes, maternal anthropometry (weight, MUAC, height), anaemia, iodine status** | protocol so no effects reported | From baseline (preconception) to 12 weeks of gestation, mean ± SD weight gain was greater for women in Arm 1 than for those in both Arms 2 and 3: 0.8±3.9kg, 0.0±3.8kg ,and 0.3±3.7kg, respectively (P < 0.0010).  BMI figures at 12 wk were 21.8 ± 3.8, 21.4 ± 3.8, and 21.6 ± 3.9, for Arms 1, 2, and 3, respectively (P = 0.082).  Change in weight from baseline to 32 wk was also greater for Arm 1 than for the other 2 arms: 6.9 ± 4.5 kg, 6.4 ± 4.1 kg, and 6.2 ± 4.4 kg, respectively (P < 0.0015). |  |
| **EFFECTS on Birth outcomes in child: birthweight, low birth weight (LBW), small for gestational age (SGA), preterm birth/ delivery (PTB)** | protocol so no effects reported | Starting the supplement before conception did not result in significantly greater newborn LAZ than starting the same intervention late in the first trimester. For neither all sites combined nor for any individual site was the LAZ for Arm 1 significantly greater than the LAZ for Arm 2. In Guatemala, the mean LAZ for Arm 1 was lower than that of Arm 2 (−0.27, P = 0.0044).  The mean LAZ, however, was higher for Arm 1 than for Arm 3 for combined sites (P < 0.01) and for DRC and Pakistan (P < 0.00625). A small positive effect size was observed for India (+0.17, P = 0.1244).  Post hoc comparison of Arm 2 with Arm 3 also revealed a significantly higher LAZ for combined sites and for Pakistan. The LAZ effect size for Arm 1 compared with Arm 3 was low (<0.2) for combined sites and in the moderate range (0.20–0.39) for DRC and Pakistan. The incidence of LBW for combined sites trended lower in both Arm 1 and Arm 2 compared with Arm 3, with an RR of 0.86 (95% CI: 0.75, 0.98, P = 0.0263) and 0.81 (95% CI: 0.70, 0.93, P = 0.0038), respectively. No differences between arms or sites were observed for either HCAZ or BMIAZ. The analysis of live births free of growth failure, demonstrated patterns consistent with that of the primary LAZ outcome: a significant treatment arm effect (P = 0.0021) and a difference between Arm 1 and Arm 3 (P = 0.0009) and Arm 2 and Arm 3 (P = 0.0166). Primary outcome sensitivity analysis adjusted for other maternal factors, including age, parity, and BMI, did not change the results. No important differences were observed according to interpregnancy interval, season of delivery,or mode of delivery among arms. For the GAA data, Arm 1 LAZ did not differ from LAZ for Arm 2. The Arm 1 compared with Arm 3 effect size was again positive for combined sites (+0.20), for India (+0.23), and for Pakistan (+0.35). None of the binary GAA -adjusted outcomes differed between Arm 1 and Arm 2. But, there was a reduction in RRs for stunting (LAZ <−2) for combined sites and for both Pakistan and India for Arm 1 compared with Arm 3, but not for Arm 2 compared with Arm 3.  There were reductions in RR for wasting (WLRAZ <−2) for Arm 1 compared with Arm 3 but not Arm 2 compared with Arm 3, for combined sites and for India. Substantial reductions in RR for SGA were evident for Arm 1 compared with Arm 3, for combined sites and for both Pakistan and India. A decrease in RR for SGA also occurred for Arm 2 compared with Arm 3, for combined sites and for India but not for Pakistan.   The deficits in mean HCAZ were small in comparison with other outcomes; no effects of the interventions on HCAZ were observed. For combined sites, the incidence of PTB by arm was 12.5%, 8.6%, and 11.5% (P = 0.0407) for Arm 1, Arm 2, and Arm 3, respectively, among all live newborns, and 11.7%, 7.4%, and 9.4% (P = 0.0047) among live newborns with birth length obtained, respectively. |  |
| **EFFECTS on Outcomes in mother at birth** | protocol so no effects reported |  |  |
| **EFFECTS on Post-natal outcomes (in Infancy)** | protocol so no effects reported |  |  |
| **EFFECTS on Outcomes in childhood after 12 m: z scores for weight (WAZ), length/height (HAZ), and BMI for age (BMIZ), weight-for-height'length (WHZ), underweight, stunting, wasting, overweight, cognitive** | protocol so no effects reported |  |  |
| **Potential Biological mediating factors (as identified by authors of the primary studies)** | protocol so no effects reported |  |  |
| **Potential Behavioural mediating factors (as identified by authors of the primary studies)** | protocol so no effects reported |  |  |
| **Potential Social mediating factors (as identified by authors of the primary studies)** | protocol so no effects reported |  |  |
| **Details on who interventions are available to, and who is accessing services and in what numbers** | protocol so no effects reported |  |  |
| **Author reflections on factors affecting the success of preconception nutrition interventions, including factors relating to leadership, financing, governance, supplies, and capacity** | protocol so no effects reported |  |  |
| **Author reflections on factors affecting the failure of preconception nutrition interventions, including factors relating to leadership, financing, governance, supplies, and capacity** |  |  |  |
| **Author recommendations on strategies to improve preconception nutrition in South Asia** |  |  |  |
| **Author recommendations on future research to improve preconception nutrition in South Asia** |  |  |  |
| **Barriers to Preconception nutritional intake** |  |  |  |
| **Enablers to preconception nutritional intake** |  |  |  |
| **Risk of Bias assessment ROB2 completed?** |  |  |  |
| **ABSTRACT** | Background: Research directed to optimizing maternal nutrition commencing prior to conception remains very limited, despite suggestive evidence of its importance in addition to ensuring an optimal nutrition environment in the periconceptional period and throughout the first trimester of pregnancy. Methods/Study design: This is an individually randomized controlled trial of the impact on birth length (primary outcome) of the time at which a maternal nutrition intervention is commenced: Arm 1: ≥ 3 mo preconception vs. Arm 2: 12-14 wk gestation vs. Arm 3: none. 192 (derived from 480) randomized mothers and living offspring in each arm in each of four research sites (Guatemala, India, Pakistan, Democratic Republic of the Congo). The intervention is a daily 20 g lipid-based (118 kcal) multi-micronutient (MMN) supplement. Women randomized to receive this intervention with body mass index (BMI) <20 or whose gestational weight gain is low will receive an additional 300 kcal/d as a balanced energy-protein supplement. Researchers will visit homes biweekly to deliver intervention and monitor compliance, pregnancy status and morbidity; ensure prenatal and delivery care; and promote breast feeding. The primary outcome is birth length. Secondary outcomes include: fetal length at 12 and 34 wk; incidence of low birth weight (LBW); neonatal/ infant anthropometry 0-6 mo of age; infectious disease morbidity; maternal, fetal, newborn, and infant epigenetics; maternal and infant nutritional status; maternal and infant microbiome; gut inflammatory biomarkers and bioactive and nutritive compounds in breast milk. The primary analysis will compare birth Length-for-Age Z-score (LAZ) among trial arms (independently for each site, estimated effect size: 0.35). Additional statistical analyses will examine the secondary outcomes and a pooled analysis of data from all sites. Discussion: Positive results of this trial will support a paradigm shift in attention to nutrition of all females of child-bearing age. Trial registration: ClinicalTrials.gov NCT01883193. Keywords: Preconception, Maternal, Nutrition, Birth length, Epigenetics, Microbiome | The objective of this secondary analysis was to identify maternal characteristics that modified the effect of maternal supplements on newborn size. Participants included 1465 maternal–newborn dyads in Guatemala, India, and Pakistan. Supplementation commenced before conception (Arm 1) or late 1st trimester (Arm 2); Arm 3 received usual care. Characteristics included body mass index (BMI), stature, anemia, age, education, socio-economic status (SES), parity, and newborn sex. Newborn outcomes were z-scores for length (LAZ), weight (WAZ), and weight to length ratio-for-age (WLRAZ). Mixed-effect regression models included treatment arm, effect modifier, and arm * effect modifier interaction as predictors, controlling for site, characteristics, and sex. Parity (para-0 vs. para ≥1), anemia (anemia/no anemia), and sex were significant effect modifiers. Effect size (95% CI) for Arm 1 vs. 3 was larger for para-0 vs. ≥1 for all outcomes (LAZ 0.56 (0.28, 0.84, p < 0.001); WAZ 0.45 (0.20, 0.07, p < 0.001); WLRAZ 0.52 (0.17, 0.88, p < 0.01) but only length for Arm 2 vs. 3. Corresponding effects for para ≥1 were >0.02. Arm 3 z-scores were all very low for para-0, but not para ≥1. Para-0 and anemia effect sizes for Arm 1 were > Arm 2 for WAZ and WLRAZ, but not LAZ. Arm 1 and 2 had higher WAZ for newborn boys vs. girls. Maternal nulliparity and anemia were associated with impaired fetal growth that was substantially improved by nutrition intervention, especially when commenced prior to conception. | Background: Reported benefits of maternal nutrition supplements commenced during pregnancy in low-resource populations have typically been quite limited. Objectives: This study tested the effects on newborn size, especially length, of commencing nutrition supplements for women in low- resource populations ≥3 mo before conception (Arm 1), compared with the same supplement commenced late in the first trimester of pregnancy (Arm 2) or not at all (control Arm 3). Methods: Women First was a 3-arm individualized randomized controlled trial (RCT). The intervention was a lipid-based micronu- trient supplement; a protein-energy supplement was also provided if maternal body mass index (kg/m2) was <20 or gestational weight gain was less than recommendations. Study sites were in rural locations of the Democratic Republic of the Congo (DRC), Guatemala, India, and Pakistan. The primary outcome was length-for-age z score (LAZ), with all anthropometry obtained <48 h post delivery. Because gestational ages were unavailable in DRC, outcomes were determined for all 4 sites from WHO newborn standards (non-gestational-age-adjusted, NGAA) as well as INTERGROWTH-21st fetal standards (3 sites, gestational age- adjusted, GAA). Results: A total of 7387 nonpregnant women were randomly assigned, yielding 2451 births with NGAA primary outcomes and 1465 with GAA outcomes. Mean LAZ and other outcomes did not differ between Arm 1 and Arm 2 using either NGAA or GAA. Mean LAZ (NGAA) for Arm 1 was greater than for Arm 3 (effect size: +0.19; 95% CI: 0.08, 0.30, P = 0.0008). For GAA outcomes, rates of stunting and small-for-gestational-age were lower in Arm 1 than in Arm 3 (RR: 0.69; 95% CI: 0.49, 0.98, P = 0.0361 and RR: 0.78; 95% CI: 0.70, 0.88, P < 0.001, respectively). Rates of preterm birth did not differ among arms. Conclusions: In low-resource populations, benefits on fetal growth– related birth outcomes were derived from nutrition supplements commenced before conception or late in the first trimester. This trial was registered at clinicaltrials.gov as NCT01883193. Am J Clin Nutr 2019;109:457–469. |

| **SN** | **10** | **11** |
| --- | --- | --- |
| **Short name** | Dhaded2020 | Young2021 |
| **Type of paper** | Secondary analysis of trial data limited to South Asia sites | Secondary analysis of trial data |
| **Authors** | Sangappa M. Dhaded, K. Michael Hambidge, Sumera Aziz Ali, Manjunath Somannava, Sarah Saleem, Omrana Pasha, Umber Khan, Veena Herekar, Sunil Vernekar, Yogesh Kumar S., Jamie E. Westcott, Vanessa R. Thorsten, Amaanti Sridhar, Abhik Das, Elizabeth McClure, Richard J. Derman, Robert L. Goldenberg, Marion Koso-Thomas, Shivaprasad S. Goudar, Nancy F. Krebs | Amy E. Young, Jennifer F. Kemp, Charis Uhlson, Jamie L. Westcott,  Sumera A. Ali, Sarah Saleem, Ana Garcès, Lester Figueroa, Manjunath S. Somannavar, Shivaprasad S. Goudar, K. Michael Hambidge Audrey E. Hendricks, Nancy F. Krebs, the Women First Preconception Maternal Nutrition Trial Group |
| **Year** | 2020 | 2021 |
| **Title** | Preconception nutrition intervention improved birth length and reduced stunting and wasting in newborns in South Asia: The Women First Randomized Controlled Trial | Improved first trimester maternal iodine status with preconception supplementation: The Women First Trial |
| **Citation** | Dhaded SM, Hambidge KM, Ali SA, Somannavar M, Saleem S, Pasha O, et al. (2020) Preconception nutrition intervention improved birth length and reduced stunting and wasting in newborns in South Asia: The Women First Randomized Controlled Trial. PLoS ONE 15(1): e0218960. | Young, A. E., Kemp, J. F., Uhlson, C., Westcott, J. L., Ali, S. A., Saleem, S., Garcès, A., Figueroa, L., Somannavar, M. S., Goudar, S. S., Hambidge, K. M., Hendricks, A. E., Krebs, N. F., & the Women First Preconception Maternal Nutrition Trial Group (2021). Improved first trimester maternal iodine status with preconception supplementation: The Women First Trial. Maternal & Child Nutrition, 17(4), e13204. |
| **doi** | doi.org/10.1371/journal. pone.0218960 | doi: 10.1111/mcn.13204 |
| **Clinical trials registration no** | https://clinicaltrials.gov/study/NCT01883193 | https://clinicaltrials.gov/study/NCT01883193 |
| **Context / setting** | Resource-poor rural communi- ties in two S. Asian countries: India and Pakistan | India, Pakistan, Guatemala . Due to limitations of cold chain capacity in the DRC (Democratic Repubic of Congo), data presented here represent only the first three of these participating sites. |
| **Protocol citation** | Hambidge KM, Krebs NF, Westcott JE, Garces A, Goudar SS, Kodkany BS, Pasha O, Tshefu A, Bose CL, Figueroa L, Goldenberg RL. Preconception maternal nutrition: a multi-site randomized controlled trial. BMC pregnancy and childbirth. 2014 Dec;14:1-6. | Hambidge KM, Krebs NF, Westcott JE, Garces A, Goudar SS, Kodkany BS, Pasha O, Tshefu A, Bose CL, Figueroa L, Goldenberg RL. Preconception maternal nutrition: a multi-site randomized controlled trial. BMC pregnancy and childbirth. 2014 Dec;14:1-6. |
| **Arising from which main trial** | The Women First Randomized Controlled Trial | The Women First Randomized Controlled Trial |
| **Study design** | 3-arm individually randomized, nonmasked, multisite controlled efficacy trial | 3-arm individually randomized, nonmasked, multisite controlled efficacy trial  Urinary I/Cr (μg/g) for women in low resource settings in rural Guatemala, Pakistan and India were measured during the first and third trimesters, and the potential association of iodine status with birth anthropometric outcomes was determined. |
| **Has Preconception intervention only arm (1/0)** | 0 | 0 |
| **Has Preconception + Pregnancy intervention arm (1/0)** | 1 | 1 |
| **Has Pregnancy intervention only arm (1/0)** | 1 | 1 |
| **Has Pregnancy, postpartum + childhood intervention arm (1/0)** | 0 | 0 |
| **Has Control arm with only usual standard of care or less intensive intervention in preconception & pregnancy (1/0)** | 1 | 1 |
| **MMN in Preconception arm** |  |  |
| **B12 in Preconception arm** |  |  |
| **LNS in Preconception arm** | 1 | 1 |
| **Viatmin A in preconception arm** |  |  |
| **Iron in preconception arm (both arms had Folic acid)** |  |  |
| **Nutritious snack in Preconception arm** |  |  |
| **Pregnancy outcomes** |  | 1 |
| **Birth outcomes** | 1 | 1 |
| **Infancy outcomes** |  |  |
| **Child outcomes** |  |  |
| **Narrative summary of main finding** | In the Pakistan and India sites of the Women 1st trial. Preconception & pregnancy LNS suplementation resulted in sigificantly higher length-for-age Z-score (LAZ); weight-for-age Z-score (WAZ), weight-to-length-ratio-for-age Z-score (WLRAZ), LAZ<-2, WAZ <-2, WLRAZ <-2, small- for-gestational-age (SGA), & low birth weight. Comparing preconception & pregnancy with control: LAZ, WAZ and SGA, were improved but not other outcomes. No significant differences in outcomes between pregnancy + preceoncpetion and Preconception alone was found. but effects between intervention and control were higher in the Preconception arm. | In 3 sites of the Women First Trial (Guatemala, Pakistan and India) the effect of LNS in preconception + pregnancy versus LNS started in pregnancy alone on iodine status was investigated amongst pregnant women. Iodine at 12 weeks gestation was higher in the preconception arm and deficiency lower compared to pregnancy only and control. The iodine status at 12 weeks gestation was also found to be nominally associated with birth length and HCAZ. Findings support the importance of 1st trimester iodine status and suggest need for preconception supplementation beyond salt iodization alone.  Maternal iodine status was better at the end of the first trimester, and prevalence of I/Cr < 150 μg/g was lower in women who had started nutrition supplementation at least 3months prior to conception compared with unsupplemented women. • Maternal iodine status at the end of the first trimester, but not in the third trimester, was positively associated with birth length and head circumference. • In two of three study sites, more than a third of the women who received supplementation prior to conception had urinary I/Cr ≥ 250 μg/g but with no evidence of adverse effects. |
| **Date of data collection** | Enrolled into the study between January and December 2014;  Data collection was completed in March 2017. | Data were collected between December 2013 and December 2016. |
| **Study objectives** | To determine the quantitative improvements in the deficits in birth anthropometry resulting from commencing maternal nutrition supplements at least three months prior to conception or in the first trimester of pregnancy in the combined S. Asian sites participating in the Women First Preconception Maternal Nutrition Trial. | The principal objective of this study, an a priori secondary analysis of the primary ‘Women First’ (WF) Preconception Maternal Nutrition trial (Hambidge et al., 2014), was to assess the impact of iodine supplementation started in the preconception period on maternal iodine status.  A second goal was to determine if iodine status of this population was associated with newborn anthropometric outcomes. Hypothesized that starting iodine supplementation pro- vided in the SQ-LNS during the preconception period would result in significantly higher urinary iodine to creatinine ratios (I/Cr) at 12 and 34 weeks gestation, representing improved maternal iodine status, which would be positively correlated with newborn anthropometry. |
| **Sample size / power** |  | Of 5646 women who were consented and randomized, 2442 women became pregnant and had the primary outcome for newborns, including 802, 835 and 805 for Arms 1, 2 and 3, respectively. From this main sample, longitudinal single spot urine and serum samples were obtained at 12 and 34 weeks gestation from approximately 100 women per arm per site. These numbers were considered to be adequate for the goals of the study and the maximum that could be collected, stored, transported and analysed within the resources of the individual sites and the WF trial. **No power calculation provided for this analysis.** |
| **Sample characteristics** |  | Baseline characteristics shown in table 1 and a supplementary figure. Look similar between arms for I/Cr ratios. Some differences between arms in age, education and parity, esp in control arm but these are not discussed. |
| **Randomisation** | The Data Coordinating Center (DCC) created the randomization scheme and centrally generated the allocation sequence for each site. The scheme included a permuted block design stratified by GN clusters for assigning individual participants to a trial arm. The allocation ratio was 1:1:1 within blocks which randomly varied between sizes of 3, 6, or 9 for each site. Once an eligible participant was identified, the randomization assignment was generated by the site data manager from the centralized computerized data management system maintained by the DCC. | Non-pregnant women in Guatemala, India and Pakistan (n 100 per arm per site) were randomized ≥ 3 months prior to conception to one of three intervention arms: a multimicronutrient-fortified lipid-based nutrient supplement containing 250-μg I per day started immediately after randomization (Arm 1), the same supplement started at 12 weeks gestation (Arm 2) and no intervention supplement (Arm 3). |
| **Blinding** | Unmasked | WF trial was an individually randomized, **nonmasked**, multisite controlled trial |
| **Age of participants (years)** | 16 to 35 y | 16 to 35 y |
| **Inclusion criteria** | Participants were newborns whose mothers met all criteria for inclusion and retention in the parent study and who had ultrasound measurements of crown- rump length (CRL) in the first trimester. They were located in resource-poor rural communi- ties in two S. Asian countries (India and Pakistan) and | For the analysis of iodine status in relation to birth measurements, only those newborns with gestational age determined by first trimester ultrasounds were used. This represented approximately two thirds of the mother–infant dyads, with equal distribution among the three arms. |
| **Exclusion criteria** |  | Exclusion not mentioned - refer to other WFT papers |
| **Preconception intervention characteristics** | Lipid-based micronutrient supplement (Nutriset, Melauney, France) which also provided a favorable balance of polyunsaturated fatty acids and small quan- tities of protein and energy. This product had minor modifications to a product previously designed for pregnant/lactating women. Participants were instructed to take one sachet daily until delivery. An additional protein-energy supplement was provided to women whose BMI was <20 kg/ m2 from the time of randomization for Arm 1 participants. This protein-energy supplement was also provided to any Arm 1 or Arm 2 participant who failed to meet guidelines for gestational weight gain in the 2nd and 3rd trimesters. Once the second supplement was commenced for either reason, it was provided until delivery. More than 90% of the women who had live births with newborn outcome measures received this additional protein energy supplement during the second and third trimesters. | The daily iodine supplement was provided in an SQ-LNS that provided 250-μg iodine daily. Arm 1 commenced the supplement immediately after randomization and continued for ≥ 3 months prior to conception until delivery, |
| **Preconception control group intervention** |  | Arm 3 received no study supplements. |
| **Intervention timing (how far before conception)** | >3 months preconception | >=3 months |
| **Intervention delivery (health systems, food systems, social protection systems)** | For Arms 1 and 2, visits by home visitor research assistants every two weeks were used to replenish the supply of trial supplements. | Home visitor employed by the project |
| **Pregnancy detection** | Participants in all three arms were visited by the home visitor research assistants every two weeks to record an interim health history and to administer a urine pregnancy test. The preg- nancy testing was combined with calendar records of menses to ascertain last menstrual period and to guide the timing of ultrasounds to be obtained between 10–12 weeks estimated gestation. | Not described - refer to other WFT papers |
| **Pregnancy intervention characteristics (if any)** | Lipid-based micronutrient supplement (Nutriset, Melauney, France) which also provided a favorable balance of polyunsaturated fatty acids and small quan- tities of protein and energy. This product had minor modifications to a product previously designed for pregnant/lactating women. Participants were instructed to take one sachet daily until delivery from after the 12 week investigations. An additional protein-energy supplement was provided to women whose BMI was <20 kg/ m2 from the beginning of the 2nd trimester for Arm 2 participants. This protein-energy supplement was also provided to any Arm 1 or Arm 2 participant who failed to meet guidelines for gestational weight gain in the 2nd and 3rd trimesters. Once the second supplement was commenced for either reason, it was provided until delivery. More than 90% of the women who had live births with newborn outcome measures received this additional protein energy supplement during the second and third trimesters. | The daily iodine supplement was provided in an SQ-LNS that provided 250-μg iodine daily. Arm 2 commenced the same intervention late in the first trimester and continued until delivery. |
| **Pregnancy control group intervention (if any)** |  |  |
| **Interventions after birth (if any)** |  |  |
| **Control group intervention after birth (if any)** |  |  |
| **Compliance measurement** | Compliance documented by inspection of calendars the women completed daily and by collection of empty, partially eaten, and unused intervention sachets. Compliance was calculated for Supplement 1 as the total number of sachets fully eaten divided by the number of days between starting Supplement 1 and delivery. Supplement 2 compliance was calculated similarly; however, the numerator is the total number of Supplement 2 sachets fully or partially eaten. Mean ± SD duration of exposure to Supplement for Arm 1 was 72.6 ± 16.8 weeks with overall compliance of 89.6 ± 9.9%. For Arm 2, duration of exposure to Supplement 1 was 26.3 ± 2.2 weeks with estimated compliance of 87.7 ± 15.7%. Supplement 2 was started in 67% of Arm 1 women prior to conception and in an additional 29% during gestation. In Arm 2, 92% of the women received Supplement 2 starting after 12 weeks gestation. The duration of Supplement 2 was 56.2 ± 25.5 weeks for Arm 1 and 23.7 ± 5.1 weeks for Arm 2. | Not described - refer to other WFT papers |
| **Adverse events** | Adverse events were monitored continuously as per protocol and reported to the overall study principal investigators and the DCC within 48 hours for all deaths and within seven days for other adverse events, including adverse pregnancy outcomes, adverse neonatal events, hospitalizations, and allergic reactions. The Data Monitoring Committee reviewed the study progress for safety, trial progress, data completion, supplement compliance, and protocol violations twice yearly. | Not described - refer to other WFT papers |
| **Engagement, category (including nutrition sensitive/specific)** | Nutrition specific | Nutrition specific |
| **Where in the overall UNICEF 2020 framework the intervention lies** | Intervention changing an the immediate determinant of Diet: LNS supplement is directly altering altering diet | Intervention changing an the immediate determinant of Diet: the iodine in the LNS supplement is directly altering altering diet |
| **Where in the Partap 2021 framework the intervention lies** | LNS supplement is an individual-level nutrition intervention. | LNS supplement is an individual-level nutrition intervention. |
| **Proposed mechanism of intervention action** | Mechanism not mentioned. Importance of preconception supplementation clearly demonstrated though, pointing to the need for enhanced efforts to improve the nutrition of all women of child-bearing age in resource-poor populations in these and similar environments in order to diminish impairment of fetal growth. | Hypothesized that starting iodine supplementation pro- vided in the SQ-LNS during the preconception period would result in significantly higher urinary iodine to creatinine ratios (I/Cr) at 12 and 34 weeks gestation, representing improved maternal iodine status, which would be positively correlated with newborn anthropometry. |
| **Exposures relating to preconception nutrition** | A fortified lipid-based supplement (LNS) (Nutriset, Malauney, France) with MMN and polyunsaturated lipids (linoleic 4.9 g and α-linolenic 0.59 g), the composition includes dried skimmed milk, soybean and peanut extract, sugar, maltodextrin stabilizers, and emulsifiers started >3 months before conception. | A fortified lipid-based supplement (LNS) (Nutriset, Malauney, France) with MMN and polyunsaturated lipids (linoleic 4.9 g and α-linolenic 0.59 g), **including 250-μg iodine daily** started >3 months before conception. |
| **Preconception measures** | Not mentioned | Age, parity, BMI, Education and SES (number of assets from a list) collected at enrolment into the study but not described. |
| **Concommittant care** | Not mentioned | Not mentioned |
| **Follow-up schedule for measurements** | Neonatal recumbent lengths, weights, and head circumferences were obtained within 48 hours of delivery by trained and periodically certified assessment teams. Measurements were obtained in triplicate, entered into the database, and the median value used for analysis. Crown rump length CRL measurements were obtained in the first trimester by ultrasonography. | All women were followed via biweekly visits from enrolment through 6 months post-partum.  Longitudinal single spot urine and serum samples were obtained at 12 and 34 weeks gestation from approximately 100 women per arm per site. Participants in Arms 1 and 2 provided samples at both time points whereas participants in Arm 3 only provided samples at 34 weeks. For Arm 2, the first urine sample was collected prior to initiation of the daily study supplement; this group thus served as control group at this time point. The India site was only able to collect samples from women in Arms 1 and 2. Spot urine samples (~30 ml) for iodine and creatinine were collected at 12 (Arms 1 and 2) and 34 weeks gestation (Arms 1, 2 and 3, excluding Arm 3 in India). Twenty-four-hour urine collections were not feasible. Specimens were collected in containers locally sourced by each site with ~5-ml aliquot transferred to a cryovial. Maternal blood samples for thyroid-stimulating hormone (TSH) were collected at the same time points and serum transferred to cryovials. Samples were stored in <80 C freezers located at each site until shipment on dry ice to the University of Colorado Pediatric Nutrition Laboratory for analyses. Upon receipt, all samples were stored at <-80 C until analysed. Details of procedures for newborn anthropometry (length, weight and head circumference), all obtained within 48 h of delivery, have been reported previously (Hambidge et al., 2019). |
| **Summary of baseline comparisons by study arm** | Nine hundred and sixty one pregnancies (972 newborns) for the combined S. Asian sites qualified for inclusion in this analysis. The number of pregnancies ranged from 295–337 per arm. Baseline maternal characteristics for S. Asian mothers with a primary outcome differed between arms only for maternal education, with a higher percentage of women in Arms 1 and 3 having no formal education (p = 0.04). | Baseline description of women who provided the urine samples for these analyses is in Table 1 and Figure S1 according to arm, each site and stage of gestation Table 1 shows differences in maternal age and parity between women sampled for this substudy between arms. Not clear how important these differences are as they are mostly small. Control arm 3 women were less educated, esp to secondary level. |
| **Pregnancy outcomes: gestational weight gain, gestational diabetes, maternal anthropometry (weight, MUAC, height), anaemia,** | Not reported | Urinary iodine concentration was measured by inductively coupled plasma mass spectrometry (Agilent Technologies 7700, Santa Clara, CA) following the CDC Urinary Iodine Method Protocol (CDC Environmental Health, 2007) with modifications.  Urinary creatinine concentrations were measured in the same batch order as iodine using a colorimetric assay (Creatinine Urinary Colorimetric Assay Kit 500701, Cayman Chemical Company, Ann Arbor, MI) per manufacturer's protocol.  TSH was measured in maternal serum from Guatemala and Pakistan using a one-step sandwich method (Beckman Coulter, Brea, CA) by the University of Colorado Hospital CTRC Laboratory (Aurora, CO). TSH in maternal serum samples from India were measured by the KLE Laboratory via chemiluminescence immunoassay (Siemens Advia XP). |
| **Preconceptual outcomes: (BMI, Hb or other outcomes which could be attributed to preconception care)** |  |  |
| **Birth outcomes in child: birthweight, low birth weight (LBW), small for gestational age (SGA), preterm birth/ delivery (PTB), cord blood b12, DXA,** | Continuous outcomes: newborn length, weight, head circumference (HC), length-for-age Z-score (LAZ), weight-for-age Z-score (WAZ), HC- for-age Z-score (HCAZ), and weight to length ratio-for-age Z-scores (WLRAZ).  Categorical outcomes include small for gestational age (SGA, weight <10th percentile for gestational age), low birth weight (LBW, <2500 g), preterm birth (PTB, gestational age <37 weeks), LAZ <-2, WAZ <-2, WLZ <-2 and WLRAZ <-2.  Also RR for Z-scores <-1 because of their predictive value for subsequent infant growth. | For newborns with first trimester ultrasound-determined gestational age, measurements were converted to gestational age adjusted length-, weight-, head circumference- and weight to length ratio-for-age z-scores (LAZ, WAZ, HCAZ and WLRAZ, respectively) using INTERGROWTH-21st foetal growth charts (Papageorghiou et al., 2014). |
| **Outcomes in mother at birth** |  | NA |
| **Post-natal outcomes (in Infancy)** | Not reported | NA |
| **Outcomes in childhood after 12 m: z scores for weight (WAZ), length/height (HAZ), and BMI for age (BMIZ), weight-for-height'length (WHZ), underweight, stunting, wasting, overweight, cognitive** |  | NA |
| **Long term outcomes** |  | NA |
| **Analysis** | The Intergrowth-21st standards were applied to describe the results of newborn anthropometry in terms of Z-scores and centiles (not shown except for weight-for-age centile <10th percentile). Study outcomes were assessed with a modified intention-to-treat approach. The overall treatment effect and pairwise comparisons (Arm 1 vs. Arm 2, Arm 1 vs. 3, as well as Arm 2 vs. 3) for the continuous anthropometry outcomes were obtained from linear models for the continuous outcomes (newborn length, weight, HC, LAZ, WAZ, HCAZ, and WLRAZ). Model-generated measures of ES with 95% confidence intervals (CI) and P values were adjusted for site and GN cluster nested within site. For binary anthropometry outcomes (SGA, LBW, PTB, LAZ <-1, LAZ <-2, WAZ <-1, WAZ <-2, WLZ <-1, WLZ <-2, WLRAZ <-1 and WLRAZ <-2), generalized linear models with generalized estimating equations were utilized to calculate relative risks with 95% CI and P values after adjusting for site while controlling for GN cluster correlations. P values are presented for descriptive purposes. | Chi-squared tests of independence were used to detect whether the number of women in each arm differed by batch for both iodine and creatinine assays. Pirate plots by batch were used to visualize iodine and creatinine. Women were assigned to categories of iodine status based on the following I/Cr: I/Cr < 50, 50–149, 150–249, ≥ 250–499 and ≥ 500 μg/g (Torlinska et al., 2018). Urinary iodine concentration (μg/L) was also determined and similarly categorized. For most analyses, an all-site regression model adjusting for site and regressions stratified by site were performed. Stratified regressions by site were not performed for dichotomous newborn anthropometry measures because the models were unstable due to low cell counts. All analyses adjusted for urinary iodine values ≤ 25 μg/g as these values were found to be less reliable. Separate analyses were performed for I/Cr at 12 and 34 weeks. Multiple linear regression was used to investigate the relationship between continuous I/Cr or TSH as the outcome and arm as the primary predictor using the glm function in base R. Given right skewness in the distributions of I/Cr and TSH, log10 transformation was used for I/Cr and TSH. Results were untransformed to interpret the results. Multinomial logistic regression was used to investigate the relationship between I/Cr category as the outcome and arm as the primary predictor using the multinom function from the nnet package (v7.3-12) (Venables & Ripley, 2002). [Multiple linear regression was used to investigate the relationship between continuous TSH as the outcome and I/Cr category as the primary predictor using the glm function in base R. The models were adjusted for arm and for I/Cr ≤ 25. 2.4.3 | Association between I/Cr and birth outcomes To investigate the relationship between continuous birth outcomes and maternal iodine status, a multiple linear regression model was used with continuous newborn anthropometry measures after adjusting for gestational age (i.e., LAZ, WAZ, HCAZ and WLRAZ) as the outcome and either I/Cr continuous or I/Cr categories as the primary predictor using the glm function in base R. A log-binomial regression was used to investigate the relationship between dichotomous birth outcome and I/Cr, with dichotomous gestational age adjusted newborn anthropometry measures (i.e., LAZ < -2, WAZ < -1.287 [consistent with cut-off for small-for-gestational age], HCAZ<-2 or WLRAZ<-2) as the outcome of the regression and either I/Cr continuous or I/Cr categories as the primary predictor using the glm function in base R. All models included arm as a covariateNominal statistical significance was assessed at p value of 0.05.  Statistical significance adjusting for multiple testing within 12 weeks and within 34 weeks models was assessed using the Bonferroni cor- rection at 0.05/18 = 0.0028 where the 18 primary models include association of eight birth outcomes (LAZ < 2, WAZ < 1.287, HCAZ < 2 and WLRAZ < 2) and arm by both continuous I/Cr and categorical I/Cr. Post hoc analyses were performed using the emmeans function from the emmeans R package v1.4.4. Tukey adjustment for multiple testing was used for post hoc comparison of categorical variables (i.e., Arms 1, 2 and 3 at 34 weeks and categori- cal I/Cr groups). For continuous newborn anthropometry measures with a nominal statistical significance (p < 0.05) with continuous I/Cr for the all-site model at either 12 or 34 weeks, authors proceeded with mediation analysis to determine whether I/Cr is a potential mediator of the relationship between arm and newborn anthropometry measures. Mediation analysis was performed using the mediate function from the mediation R package v4.5.0 (Tingley et al., 2014) setting robustSE to TRUE and using 1000 simulation replicates. As with the other primary analyses, outliers were excluded. |
| **EFFECTS on Preconception outcomes: maternal anthropometry (weight, MUAC, height. BMI), Haemoglobin (Hb), anaemia, iodine status** |  |  |
| **EFFECTS on Pregnancy outcomes: gestational weigt gain, gestational diabetes, maternal anthropometry (weight, MUAC, height), anaemia, iodine status** | Not reported | At 12 weeks gestation, adjusted mean I/Cr for combined sites was significantly higher for Arm 1 versus Arm 2. Similarly, a statistically significant higher adjusted mean I/Cr for Arm 1 versus Arm 2 was observed for both Guatemala and India but not for Pakistan. The distributions of I/Cr categories at 12 weeks differed by arm overall (p < 0.0001) and for Guatemala (p < 0.01) and India (p < 0.01) but not Pakistan (p = 0.54). The adjusted prevalence (95% CI) of I/Cr < 150 was lower for Arm 1 versus Arm 2 in India (26% [95% CI: 17%, 30%] vs. 47% [35%, 58%]) and Guatemala (30% [18%, 38%] vs. 44% [33%, 55%]). The adjusted prevalence of I/Cr < 150 in Pakistan was generally higher than India and Guatemala but similar by arm (44% [31%, 56%] vs. 39% [27%, 51%] in Arms 1 and 2, respectively). Adjusted prevalence of I/Cr ≥ 500 also differed by arm and by site; no women had I/Cr ≥ 500 in Pakistan once outliers were removed. A statistically significant difference in the adjusted mean I/Cr for both intervention arms compared with the control arm was observed for Guatemala but not for Pakistan; no data for Arm 3 in India were available for comparison with the intervention arms. No statistical differences were observed between Arm 1 versus Arm 2 for any of the three sites at 34 weeks.  TSH was significantly associated with I/Cr category at 12 weeks for India (p < 0.01) but not for Guatemala (p = 0.92) or Pakistan (p = 0.42). For India, adjusted TSH level increased as I/Cr increased. TSH did not differ significantly by arm at 12 weeks or by arm or I/Cr group at 34 weeks for any of the sites. |
| **EFFECTS on Birth outcomes in child: birthweight, low birth weight (LBW), small for gestational age (SGA), preterm birth/ delivery (PTB)** | Preconception & pregnancy LNS supplementation versus control resulted in sigificantly higher length-for-age Z-score (LAZ) +0.29 (0.11–0.46, p = 0.0011); weight-for-age Z-score (WAZ) +0.22 (0.07–0.37, p = 0.0043); weight-to-length-ratio-for-age Z-score (WLRAZ) +0.27 (0.06–0.48, p = 0.0133); LAZ<-2, 0.56 (0.38–0.82, p = 0.0032); WAZ <-2, 0.68 (0.53–0.88, p = 0.0028); WLRAZ <-2, 0.76 (0.64–0.89, p = 0.0011); small- for-gestational-age (SGA), 0.74 (0.66–0.83, p<0.0001); low birth weight 0.81 (0.66–1.00, p = 0.0461) Newborn with anthropometric outcomes ranged from 299–340 per arm. Twin pregnancies were equally distributed between the three arms (2.5% of total included in this analysis). Anthropometric measures for controls (Arm 3) were determined by sex. Mean ± SD lengths were 46.83 ± 2.48 and 47.53 ± 2.67 cm for females and males, respectively. Corre sponding figures for weight were 2605 ± 421 and 2731 ± 433 g and for head circumference were 32.44 ± 1.62 and 33.12 ±1.51 cm. Effects of the maternal supplements on newborn anthropometry did not vary by sex (p = 0.2133). Accordingly, sexes have been combined for all analyses. Ninety-five percent CI for the positive effect sizes of the nutrition intervention commencing >3 months prior to conception (Arm 1) in comparison with control Arm 3 did not include zero for LAZ, WAZ, and WLRAZ. In contrast, corresponding CI for effect sizes for continuous variables for the nutrition intervention commencing in the first trimester of pregnancy (Arm 2 vs. 3) included zero with the only exception of LAZ.  For binary variables, there was no reduction in relative risk for preterm delivery for either the preconception (Arm 1) or 1st trimester intervention (Arm 2) compared to the control arm (Arm 3). The intervention commencing in the preconception period (Arm 1) resulted in a reduction of 26% in the relative risk of SGA (p<0.0001) and a reduction in the incidence of LBW of 19% ((RR 0.81 (0.66–1.00, p = 0.0461)) in comparison with control arm (Arm 3). There were also substantial reductions (p<0.01) in the relative risks for stunting (LAZ <-2, 44%), underweight (WAZ <-2, 32%), and wasting (WLRAZ <-2, 24%).  For the inter- vention commencing in the first trimester (Arm 2) compared with the control arm (Arm 3), several outcomes were marginally different (P<0.05) but none of these outcomes differed at α = 0.01 level. Direct comparison of Arm 1 vs Arm 2 showed reduced risk for SGA, WAZ<-1 and WLRAZ<-1 for the preconception intervention. The only suggestion of the intervention commencing at either time in relation to conception having a benefit on head size was a RR for head circumference <3rd centile for Arm 1 vs. 3 of 0.73 (95% CI: 0.53–1.00). | For the all-site analysis at 12 weeks, adjusting for site and arm, there was a **small, nominally significant association between I/Cr and birth LAZ (p = 0.028)** and **between I/Cr and birth HCAZ (p < 0.01)** but not for other continuous or dichotomous newborn anthropometric measures. The adjusted effect size for LAZ was +0.07 per 100 unit increase in I/Cr and was primarily driven by Guatemalan data. The adjusted effect size for HCAZ was +0.09 per 100 unit increase in I/Cr and was driven by India (0.18 per 100 I/Cr, p < 0.01) and somewhat by Guatemala (0.10 per 100 I/Cr, p = 0.034) but was not observed in Pakistan.  No statistically significant (i.e., p < 0.05) associations were observed between the 34-week I/Cr and newborn anthropometric outcomes for the all sites analysis. Mediation analyses revealed that I/Cr at 12 weeks for combined sites was a nominally significant mediator for LAZ (p = 0.046) and HCAZ (p = 0.016). No individual sites showed strong or statistically significant evidence of mediation for LAZ. The HCAZ mediation result is driven primarily by India (p = 0.023) and partially by Guatemala (p = 0.06) . |
| **EFFECTS on Outcomes in mother at birth** |  | NA |
| **EFFECTS on Post-natal outcomes (in Infancy)** |  | NA |
| **EFFECTS on Outcomes in childhood after 12 m: z scores for weight (WAZ), length/height (HAZ), and BMI for age (BMIZ), weight-for-height'length (WHZ), underweight, stunting, wasting, overweight, cognitive** |  | NA |
| **Potential Biological mediating factors (as identified by authors of the primary studies)** |  | I/Cr levels in the highest category at 12weeks were associated with statistically greater birth length and head circumference, and mediation analysis supported possible causality. |
| **Potential Behavioural mediating factors (as identified by authors of the primary studies)** | Not mentioned | Not mentioned |
| **Potential Social mediating factors (as identified by authors of the primary studies)** | Not mentioned | Not mentioned |
| **Details on who interventions are available to, and who is accessing services and in what numbers** | Not mentioned | Not mentioned |
| **Author reflections on factors affecting the success of preconception nutrition interventions, including factors relating to leadership, financing, governance, supplies, and capacity** | Not mentioned | Not mentioned |
| **Author reflections on factors affecting the failure of preconception nutrition interventions, including factors relating to leadership, financing, governance, supplies, and capacity** | Not mentioned | Not mentioned |
| **Author recommendations on strategies to improve preconception nutrition in South Asia** | These results provide quantitative information on the benefits to fetal growth achievable by improvement in intake of energy, protein, and micronutrients commencing before the start of the second tri- mester of gestation without any other interventions directed to improvement of the poor envi- ronments in which the participants lived. Furthermore, the relatively large point estimates for the effects of the nutrition supplement commenced >3 months prior to conception support **enhanced efforts to improve the nutrition of all women of child-bearing age in resource-poor populations in these and similar environments in order to diminish impairment of fetal growth.** | Need for improvement of iodine status of women of reproductive age beyond salt fortification alone in settings where there is lack of awareness on iodine.  The absence of any significant associations of third trimester iodine status with birth anthropometry adds to the accumulating evidence that nutritional supplemen- tation initiated several months prior to conception has more beneficial effects compared with supplementation initiated after the first trimester. |
| **Author recommendations on future research to improve preconception nutrition in South Asia** |  | I/Cr levels in the highest category at 12weeks were associated with statistically greater birth length and head circumference, and mediation analysis supported possible causality. This finding warrants replication and raises an important perspective to be considered in assessment of optimal maternal iodine intake and status, especially in highly vulnerable populations.  With maternal iodine status and thyroid function playing a crucial role in embryogenesis (Zoeller & Rovet, 2004), it is important that maternal iodine status be sufficient very early in the first trimester of gestation. Future investigations are merited to determine the effects of maternal iodine and thyroid status, specifically in the period prior to conception, on neurodevelopment in the offspring of the participants in this study. |
| **Barriers to Preconception nutritional intake** | Not discussed | Not mentioned |
| **Enablers to preconception nutritional intake** | Not discussed | Not mentioned |
| **Risk of Bias assessment ROB2 completed?** |  |  |
| **ABSTRACT** | South Asia has >50% of the global burden of low birth weight (LBW). The objective was to determine the extent to which maternal nutrition interventions commenced before concep- tion or in the 1st trimester improved fetal growth in this region. This was a secondary analysis of combined newborn anthropometric data for the South Asian sites (India and Pakistan) in the Women First Preconception Maternal Nutrition Trial. Participants were 972 newborn of mothers who were poor, rural, unselected on basis of nutritional status, and had been randomized to receive a daily lipid-based micronutrient supplement commencing >3 months prior to conception (Arm 1), in the 1st trimester (Arm 2), or not at all (Arm 3). An additional protein-energy supplement was provided if BMI <20 kg/m2 or gestational weight gain was less than guidelines. Gestational age was established in the 1st trimester and newborn anthropometry obtained <48-hours post-delivery. Mean differences at birth between Arm 1 vs. 3 were length +5.3mm and weight +89g. Effect sizes (ES) and relative risks (RR) with 95% CI for Arm 1 vs. 3 were: length-for-age Z-score (LAZ) +0.29 (0.11–0.46, p = 0.0011); weight-for-age Z-score (WAZ) +0.22 (0.07–0.37, p = 0.0043); weight-to-length-ratio-for-age Z-score (WLRAZ) +0.27 (0.06–0.48, p = 0.0133); LAZ<-2, 0.56 (0.38–0.82, p = 0.0032); WAZ <-2, 0.68 (0.53–0.88, p = 0.0028); WLRAZ <-2, 0.76 (0.64–0.89, p = 0.0011); small- for-gestational-age (SGA), 0.74 (0.66–0.83, p<0.0001); low birth weight 0.81 (0.66–1.00, p = 0.0461). For Arm 2 vs. 3, LAZ, 0.21 (0.04–0.38); WAZ <-2, 0.70 (0.53–0.92); and SGA, 0.88 (0.79–0.97) were only marginally different. ES or RR did not differ for preterm birth for either Arm 1 vs. 3 or 2 vs. 3. In conclusion, point estimates for both continuous and binary anthropometric outcomes were consistently more favorable when maternal nutrition supple- ments were commenced >3 months prior to conception indicating benefits to fetal growth of improving women’s nutrition in this population. | Abstract Maternal iodine (I) status is critical in embryonic and foetal development. We examined the effect of preconception iodine supplementation on maternal iodine status and on birth outcomes. Non-pregnant women in Guatemala, India and Pakistan (n ~ 100 per arm per site) were randomized ≥ 3 months prior to conception to one of three intervention arms: a multimicronutrient-fortified lipid-based nutrient supplement containing 250-μg I per day started immediately after randomization (Arm 1), the same supplement started at ~12 weeks gestation (Arm 2) and no intervention supplement (Arm 3). Urinary I (μg/L) to creatinine (mg/dl) ratios (I/Cr) were determined at 12 weeks for Arm 1 versus Arm 2 (before supplement started) and 34 weeks for all arms. Generalized linear models were used to assess the relationship of I/Cr with arm and with newborn anthropometry. At 12 weeks gestation, adjusted mean I/Cr (μg/g) for all sites combined was significantly higher for Arm 1 versus Arm 2: (203 [95% CI: 189, 217] vs. 163 [95% CI: 152, 175], p < 0.0001). Overall adjusted prevalence of I/Cr < 150 μg/g was also lower in Arm 1 versus Arm 2: 32% (95% CI: 26%, 38%) versus 43% (95% CI: 37%, 49%) (p = 0.0052). At 34 weeks, adjusted mean I/Cr for Arm 1 (235, 95% CI: 220, 252) and Arm 2 (254, 95% CI: 238, 272) did not differ significantly but were significantly higher than Arm 3 (200, 95% CI: 184, 218) (p < 0.0001). Nominally significant positive associations were observed between I/Cr at 12 weeks and birth length and head circumference z-scores (p = 0.028 and p = 0.005, respectively). These findings support the importance of first trimester iodine status and suggest need for preconception supplementation beyond salt iodization alone. |

| **SN** | **12** | **13** | **14** |
| --- | --- | --- | --- |
| **Short name** | Potdar2014 | Sahariah2016 | Lawande2018 |
| **Type of paper** | Primary analysis of trial data - main trial paper | Secondary analysis of trial data | Secondary analysis of trial data |
| **Authors** | Ramesh D Potdar, Sirazul A Sahariah, Meera Gandhi, Sarah H Kehoe, Nick Brown, Harshad Sane, Monika Dayama, Swati Jha, Ashwin Lawande, Patsy J Coakley, Ella Marley-Zagar, Harsha Chopra, Devi Shivshankaran, Purvi Chheda-Gala, Priyadarshini Muley-Lotankar, G Subbulakshmi, Andrew K Wills, Vanessa A Cox, Vijaya Taskar, David JP Barker, Alan A Jackson, Barrie M Margetts, and Caroline HD Fall | Sirazul A Sahariah, Ramesh D Potdar, Meera Gandhi, Sarah H Kehoe, Nick Brown, Harshad Sane, Patsy J Coakley, Ella Marley-Zagar, Harsha Chopra, Devi Shivshankaran, Vanessa Cox, Alan A Jackson, Barrie M Margetts, and Caroline HD Fall | Ashwin Lawande, Chiara Di Gravio, Ramesh D. Potdar, Sirazul A. Sahariah, Meera Gandhi, Harsha Chopra, Harshad Sane, Sarah H. Kehoe, Ella Marley‐Zagar, Barrie M. Margetts, Alan A. Jackson, Caroline H. D. Fall |
| **Year** | 2014 | 2016 | 2018 |
| **Title** | Improving women’s diet quality preconceptionally and during gestation: effects on birth weight and prevalence of low birth weight—a randomized controlled efficacy trial in India (Mumbai Maternal Nutrition Project) | A daily snack containing green leafy vegetables, fruit and milk before and during pregnancy prevented gestational diabetes in a randomized controlled trial in Mumbai | Effect of a micronutrient‐rich snack taken preconceptionally and throughout pregnancy on ultrasound measures of fetal growth: The Mumbai Maternal Nutrition Project (MMNP). |
| **Citation** | Potdar RD, Sahariah SA, Gandhi M, Kehoe SH, Brown N, Sane H, Dayama M, Jha S, Lawande A, Coakley PJ, Marley-Zagar E. Improving women’s diet quality preconceptionally and during gestation: effects on birth weight and prevalence of low birth weight—a randomized controlled efficacy trial in India (Mumbai Maternal Nutrition Project). The American journal of clinical nutrition. 2014 Nov 1;100(5):1257-68. | Sahariah SA, Potdar RD, Gandhi M, Kehoe SH, Brown N, Sane H, Coakley PJ, Marley-Zagar E, Chopra H, Shivshankaran D, Cox VA. A daily snack containing leafy green vegetables, fruit, and milk before and during pregnancy prevents gestational diabetes in a randomized, controlled trial in Mumbai, India. The Journal of nutrition. 2016 Jul 1;146(7):1453S-60S. | Lawande A, Di Gravio C, Potdar RD, Sahariah SA, Gandhi M, Chopra H, Sane H, Kehoe SH, Marley‐Zagar E, Margetts BM, Jackson AA. Effect of a micronutrient‐rich snack taken preconceptionally and throughout pregnancy on ultrasound measures of fetal growth: The Mumbai Maternal Nutrition Project (MMNP). Maternal & child nutrition. 2018 Jan;14(1):e12441. |
| **doi** | doi: 10.3945/ajcn.114.084921 | DOI:10.3945/jn.115.223461 | doi: 10.1111/mcn.12441 |
| **Clinical trials registration no** | ISRCTN62811278 https://www.isrctn.com/ISRCTN62811278 | ISRCTN62811278 https://www.isrctn.com/ISRCTN62811278 https://doi.org/10.1186/ISRCTN62811278 | ISRCTN62811278 https://www.isrctn.com/ISRCTN62811278 https://doi.org/10.1186/ISRCTN62811278 |
| **Context / setting** | Mumbai, India: Bandra, Khar, Santa Cruz, and Andheri areas of the city of Mumbai, India, in slums covered by the health and social programs of the nongovernmental organization the Centre for the Study of Social Change (CSSC). | Mumbai, India | Mumbai, India |
| **Protocol citation** | The trial protocol can be obtained from the corresponding author. Protocol available in the supplementary online material attached to online version of Sahariah 2016. | Protocol available in the supplementary online material attached to this paper online version. | Protocol available in the supplementary online material attached to online version of Sahariah 2016. |
| **Arising from which main trial** | The Mumbai Maternal Nutrition Project (MMNP) SARAS trial. | The Mumbai Maternal Nutrition Project (MMNP) SARAS trial. | The Mumbai Maternal Nutrition Project (MMNP) SARAS trial. |
| **Study design** | Nonblinded, individually randomized controlled efficacy trial. | nonblinded, individually randomized con- trolled efficacy trial. | nonblinded, individually randomized con- trolled efficacy trial. |
| **Has Preconception intervention only arm (1/0)** |  |  |  |
| **Has Preconception + Pregnancy intervention arm (1/0)** | 1 | 1 | 1 |
| **Has Pregnancy intervention only arm (1/0)** |  |  |  |
| **Has Pregnancy, postpartum + childhood intervention arm (1/0)** |  |  |  |
| **Has Control arm with only usual standard of care or less intensive intervention in preconception & pregnancy (1/0)** | 1 | 1 | 1 |
| **MMN in Preconception arm** |  |  |  |
| **B12 in Preconception arm** |  |  |  |
| **LNS in Preconception arm** |  |  |  |
| **Viatmin A in preconception arm** |  |  |  |
| **Iron in preconception arm (both arms had Folic acid)** |  |  |  |
| **Nutritious snack in Preconception arm** | 1 | 1 | 1 |
| **Pregnancy outcomes** |  | 1 | 1 |
| **Birth outcomes** | 1 |  |  |
| **Infancy outcomes** |  |  |  |
| **Child outcomes** |  |  |  |
| **Narrative summary of main finding** | A daily snack providing additional green leafy vegetables, fruit, and milk before conception and throughout pregnancy had no overall effect on birth weight. Per-protocol and subgroup analyses indicated a possible increase in birth weight if the mother was supplemented >3 mo before conception and was not underweight. | Improving dietary micronutrient quality by increasing intake of leafy green vegetables, fruit, and/or milk may have an important protective effect against the development of gestational diabetes mellitus (GDM). ITT analysis showed prevalence of GDM was 7.3% compared with 12.4% in controls; OR: 0.56; 95% CI: 0.36, 0.86; P = 0.008. The reduction in GDM remained significant after adjusting for prepregnancy adiposity and fat or weight gain during pregnancy. Results were similar in per protocol analyses. There was no effect on GDM diagnosed based on WHO 2013 criteria | Non‐pregnant women living in Mumbai slums, India who received a daily micronutrient‐rich snack containing green leafy vegetables, fruit, and milk (treatment) vs a snack made from lower‐micronutrient vegetables (control) had similar fetal size or growth at all stages of pregnancy, whether they started the supplement >3 months preconcpetually or less.  2nd trimester interactions between parity & allocation group for biparietal diameter and femur length were found with both being smaller among fetuses of primiparous women and larger among those of multiparous women, in the treatment group compared with the controls. Key messages:  - a food‐based micronutrient‐rich supplement had no overall effect on standard ultrasound measures of fetal size or growth. - In a subgroup analysis the intervention may have increased growth in fetal biparietal diameter and femur length up to 20 weeks among multiparous mothers, and reduced it among primiparous mothers. |
| **Date of data collection** | January 2006 to May 2012 | January 2006 to May 2012 | Jan 2006 to May 2012 |
| **Study objectives** | To test whether improving women’s dietary micro-nutrient quality for a sustained period before conception and throughout pregnancy increases birth weight in a high-risk Indian population. The trial was **not** designed to answer another important question of whether preconceptional supplementation is more effective than starting supplementation, as is more usual, after pregnancy has been diagnosed. | To test whether increasing women's dietary intake of leafy green vegetables, fruit, and milk before conception and throughout pregnancy reduced their risk of gestational diebetes mellitus (GDM). | To test whether improving women’s dietary micro-nutrient quality for a sustained period before conception and throughout pregnancy improves fetal growth in a high-risk Indian population. Birth weight is not an optimal proxy for fetal growth, because two babies with the same birth weight and size may achieve this by different growth trajectories.  Aimed to use ultrasound measures of fetal size in the MMNP to  (a) determine whether supplementation influenced fetal size and growth and  (b) determine the timing during pregnancy of any effect.  Hypothesised that  - fetal measurements would be increased in the treatment group,  - differences would be present from early pregnancy,  - effects would increase with maternal pre‐pregnancy BMI. |
| **Sample size / power** | Before the start of the trial, authors estimated that, on the basis of an SD for birth weight of 600 g in this population, a sample size of 1500 subjects (750 subjects/group) would give >=85% power to detect an increase in birth weight in the treatment group of 100 g and an interaction between allocation group and maternal BMI (expected effect of 125 g in the lowest one-third, 100 g in the middle one-third, and 75g in the highest one-third) significant at the 5% level. | 6513 nonpregnant women participated in the trial, of whom 2291 became pregnant. Of these, 241 had either an abortion or termination of pregnancy before 28 wk gestation, and they lost contact with 22 women.  Of the remaining 2028, only 1008 (50%) attended for an OGTT. This resulted in a fairly small sample size (100 cases of GDM) and could have biased the results. | Not mentioned |
| **Sample characteristics** |  |  | 2291 women (35%) became pregnant during the trial and were followed up.  Age at conception: median 25 years (interquartile range: 22–28);  34% underweight (BMI < 18.5 kg/m2)  9% overweight (BMI 25-29.9 kg/m2)  2% were obese (BMI ≥ 30 kg/m2).  79% not in paid work (79%),  88% completed secondary education (), 70% Hindu  55% Marathi or Gujarati as 1st language  46% primiparous. |
| **Randomisation** | Random assignment was purposively generated remotely in Southampton, United Kingdom. Women were individually randomly assigned, stratified by age, and BMI (3 groups for each). Because recruitment took place over several years, and authors aimed to start supplementation quickly after recruitment to give as much time as possible on supplementation before pregnancy, random assignment was carried out in batches after every 1–3 recruitment camps. Initially, authors used an SPSS (version 14.0; SPSS Inc) randomization function, which produced approximately equal groups. Hoauthorsver, by April 2007, allocation groups differed in size by >100 subjects, and authors changed to a similar procedure that incorporated a block- randomization program developed in house by using STATA software (version 12.1; StataCorp LP), which subsequently allocated exactly equal numbers to each group. Age and BMI stratification was identical for both methods. | Women were individually randomly assigned and stratified by age and BMI (3 groups for each). | see Potdar 2014 |
| **Blinding** | Full blinding is not possible in a food-based trial. Treatment and control snacks were outwardly similar, but their contents looked different. To obscure allocation, authors created 2 treatment and 2 control groups, each with an independent set of recipes. Four different snacks were produced daily in an unpredictable pattern.  Staff who measured outcomes were blinded to the women’s allocation groups. The 2 treatment groups and 2 control groups were merged for analysis. Full blinding was impossible, and if women thought they were getting the healthier snack, this belief could have modified their behaviors in other ways fa- voring a better pregnancy outcome. However, this effect would not explain the BMI interaction. | Full blinding is impossible in a food-based trial. Treatment and control snacks were outwardly similar,but their contents looked different.  To obscure allocation, we created 2 treatment and 2 control groups, each with its own recipes, which were merged for the analysis. Four different snacks therefore were produced daily. Staff who measured outcomes were blind to the women's allocation group. | See Potdar 2014 |
| **Age of participants (years)** | <40 years. No minimum defined in eligibility. Median (IQR) age: 25 (22, 28) years | <40 y | <40 years. No minimum defined in eligibility. Median (IQR) age: 25 (22, 28) years |
| **Inclusion criteria** | Recruited nonpregnant women who were intending to have children and planned to test the effect of starting supplementation >3 mo before pregnancy. Women were eligible if aged <40 y, married, nonpregnant, not sterilized, planning to have more children, and intending to deliver in Mumbai. Initially, authors followed up pregnancies only if the women started supplementation >=90 d before their last menstrual period. Women who became pregnant sooner than this were excluded from additional supplementation and follow-up. However, from December 2008, authors followed up all pregnancies and censored data at the analysis stage. In per-protocol and main analyses, intrauterine deaths, stillbirths, twin or triplet pregnancies, and major congenital abnormalities were included only to compare their prevalence between groups and then excluded, and the analysis was limited to live singleton newborns measured within 10 d | Women were eligible if they were aged <40 y, married, not pregnant, not sterilized, planning to have more children, and intending to deliver in Mumbai. | Low‐income non‐pregnant women in Mumbai, who intended to have children, were randomly assigned to receive either a daily micronutrient‐rich snack containing green leafy vegetables, fruit, and milk or a lower‐micronutrient snack, in addition to their usual diet from before pregnancy until delivery |
| **Exclusion criteria** | Excluded 532 women (8% in both groups) who became pregnant too early (before December 2008: <90 d after starting supplementation; after December 2008: before starting supplementation). | not mentioned See Potdar 2014 | Excluded from analysis: twins (n = 26); fetuses with major congenital abnormalities (n = 12); ; missing LMP date (n = 69); pregnancies in which the LMP‐ derived gestation was >2 weeks different from gestation from <20 weeks ultrasound scan (n = 197); pregnant women with no delivery outcome (n = 22); newborns with missing information on sex (n = 41). Excluded all pregnancies resulting in abortions, terminations, stillbirths, and maternal deaths (n = 245) because of unknown fetal sex (illegal to reveal sex in pregnancy). Two preterm babies (<37 weeks of gestation) whose gestational‐age‐adjusted fetal measures, were >3 SDs higher than the population mean were excluded because LMP was likely incorrect. |
| **Preconception intervention characteristics** | Snacks were produced daily except Sundays and public holidays, packaged in color-coded bags to match identity cards, and transported to supplementation centers by autorickshaw. Women were asked not to alter their usual diets, and snacks were available from 1500 to 1800 to interfere least with main meals. During Ramadan, when Muslims eat only between sunset and sunrise, the time was extended to 2000. Women were given 1 snack/d, and consumption was observed and recorded (1 = full; 0.5 = greater than or equal to half; 0 = less than half).  Snacks resembled local street foods such as samosas and fritters, which could be filled with key ingredients, cooked, packaged, and easily transported.. Treatment snacks contained fresh and dried green leafy vegetables, milk, and dried fruit,. Green leafy vegetables included spinach, colocasia, amaranth, fenugreek, coriander, shepu, spring onion stalk, and curry leaves. Initially, we used dried green leafy vegetables to provide more micronutrients per unit volume of green leafy vegetables. These vegetables were commercially produced, air-dried at room temperature, and supplied as powders or flakes. However, as the trial progressed, we increased the proportion of fresh leaves purchased from local markets, which improved the palatability without major changes in the nutrient content.. Dried fruits included figs, dates, raisins, mango, apple, gooseberry (amla?), and guava. Milk was included as commercially bought full-fat milk powder.   Created 70 treatment from these foods of which 8–14 were in use at any time. Snacks were made fresh each day in a dedicated study kitchen at the CSSC. Both treatment and control snacks had similar added spices, bindings, and covering ingredients (wheat, rice, or chickpea flour and semolina) and (except for one recipe in each allocation group) were cooked by deep frying in sunflower oil. Treatment snacks contained 10–23% of the WHO/FAO recommended Reference Nutrient Intakes for b-carotene, riboflavin, folate, vitamin B-12, calcium, and iron. | The intervention was a daily snack resembling local street foods such as samosas and fritters, prepared fresh each day and fried in sunflower oil. Treatment snacks contained leafy green vegetables in fresh (~30 g) or dried (~7.5 g) form, full-fat milk powder (12–16 g), and dried fruits (4–60 g). Control snacks were made from low-micronutrient vegetables such as potato and onion. To avoid monotony, researchers created multiple recipes from these foods. On average, treatment snacks contained 10–23% of the WHO/ FAO recommended Reference Nutrient Intake for b-carotene, riboflavin, folate, vitamin B-12, calcium, and iron; they contained 0.69 MJ energy and 6.4 g protein, compared with 0.37 MJ and 2.4 g in control snacks. Snacks were produced daily except on holidays, packaged in color-coded bags, and transported to 61 supplementation centers. Women were asked to maintain their usual diet, and snacks were available from 1500 to 1800, to interfere least with meals. Women were offered 1 snack/d, and consumption was observed and recorded. | See Potdar 2014 |
| **Preconception control group intervention** | Control snacks were made from low-micronutrient vegetables such as potato, tapioca, and onion, which were purchased from local markets.  Created 40 control recipes from these foods of which 8–14 were in use at any time. Snacks were made fresh each day in a dedicated study kitchen at the CSSC.  Both treatment and control snacks had similar added spices, bindings, and covering ingredients (wheat, rice, or chickpea flour and semolina) and (except for one recipe in each allocation group) were cooked by deep frying in sunflower oil. Control snacks containd <= 1% of RNI for b-Carotene (RE), Riboflavin (mg), Folate(mg) & Vitamin C (mg). 7% of RNI for Vitamin B-12 (mg), 2% for Calcium (mg) & 5% for Iron(mg). | Control snacks were made from low-micronutrient vegetables such as potato, tapioca, and onion, which were purchased from local markets. | See Potdar 2014 |
| **Intervention timing (how far before conception)** | >=3 snacks/wk from 90 d before the last menstrual period date until delivery | started as > 3 months before preconception but later from Dec 2008 allowed all women to be followed up so some had <3 months | See Potdar 2014 |
| **Intervention delivery (health systems, food systems, social protection systems)** | Project's own system delivered intervention. | Via Project staff | See Potdar 2014 |
| **Pregnancy detection** | Centre staff also recorded women’s serial menstrual period dates at snack distribution centres.  Women who missed 2 periods had a urinary pregnancy test, and if this test was positive, the women were invited to a central clinic at the CSSC at 9–13 wk of gestation for an obstetric assessment, hemoglobin measurement, and ultrasonography to confirm and date the pregnancy (18). Ultrasound scans were conducted by a single operator throughout the trial, and the sex of the fetus was never divulged to the parents; 51% of pregnancies were scanned before 12 wk, and an additional 21% of pregnancies were scanned before 20 wk. | Center staff recorded the women's serial last menstrual period (LMP) dates. | See Potdar 2014 |
| **Pregnancy intervention characteristics (if any)** | The pregnancy intervention was the same as the preconeption intervention viz. Snacks were produced daily except Sundays and public holidays, packaged in color-coded bags to match identity cards, and transported to supplementation centers by autorickshaw. Women were given 1 snack/d, and consumption was observed and recorded (1 = full; 0.5 = greater than or equal to half; 0 = less than half). Women were asked not to alter their usual diets, and snacks were available from 1500 to 1800 to interfere least with main meals. During Ramadan, when Muslims eat only between sunset and sunrise, the time was extended to 2000.   After pregnancy women continued to receive antenatal care from their own obstetricians and chose their place of delivery. Obstetricians generally prescribed iron (100 mg) and folic acid (500 mg) from the confirmation of pregnancy. If these supplements were not prescribed, or women were unable to afford them, the study team supplied the supplements free of charge. Women shown to have anemia or gestational diabetes were referred to their obstetricians for additional management. Women who opted for a termination of pregnancy made this decision in discussion with their obstetrician, and the research team played no role. Women continued to receive study snacks until delivery. | Same snacks as in preconception. Routine ANC and IFA supplementation but IFA provided if the omen did not access them. | See Potdar 2014 |
| **Pregnancy control group intervention (if any)** | Care in pregnancy was same in control and intervention arms except for snacks:  After pregnancy women continued to receive antenatal care from their own obstetricians and chose their place of delivery. Obstetricians generally prescribed iron (100 mg) and folic acid (500 mg) from the confirmation of pregnancy. If these supplements were not prescribed, or women were unable to afford them, the study team supplied the supplements free of charge. Women shown to have anemia or gestational diabetes were referred to their obstetricians for additional management. | Routine ANC and IFA supplementation. | See Potdar 2014 |
| **Interventions after birth (if any)** | None | None | None |
| **Control group intervention after birth (if any)** | None | None | None |
| **Compliance measurement** | Women were given 1 snack/d, and consumption was observed and recorded (1 = full; 0.5 = greater than or equal to half; 0 = less than half). Centre staff also recorded women’s serial menstrual period dates. Compliance was defined as an average of >=3 snacks/wk from 90 d before the last menstrual period date until delivery. Examined the intervention effect on birth weight at different levels of maternal compliance: Fewer women in the treatment group than control group were compliant (45% compared with 57%, respectively). Compliance fell in the May through June holiday season each year and during major festivals. Compliance was unrelated to maternal BMI or socioeconomic status and was similar in the 3 mo before pregnancy (treatment: 44%; control: 53%) and during pregnancy (treatment: 38%; control: 49%).  Individual compliance fell with an increasing length of time in the study. There was no evidence of a larger intervention effect in compliant women, and indeed, compliance was associated with a reduction in the effect of the intervention on birth weight in the intention-to-treat analysis. This result remained true after adjustment for additional potential con- founders (household size, religion, tobacco use, supplementation center, timing of the pregnancy within the trial, and length of time in the study). | Compliance was defined as a mean of ≥3 snacks/wk from 90 d before the LMP date until delivery. | To adjust for compliance, average compliance was calculated from 3 months prior to the LMP (or from recruitment if supplementation <3 months) up to the visit of interest.  Throughout pregnancy, the percentage of compliant women was higher in the control group than in the treatment group. In the ITT analysis, 56% of women were compliant at visit 1 (treatment: 51%, control: 61%), and 58% were compliant at visits 2 (treatment: 50% control: 64%) and 3 (treatment: 50%, control: 66%). The percentage of compliant women decreased in the PP analysis (52%, 54%, and 55% at visits 1, 2, and 3, respectively). Less than 50% of the women in the treatment group (45%, 45%, and 47% at visits 1, 2, and 3, respectively) and approximately 60% of women in the control group were compliant (58%, 61%, and 62% in each of the three visits). Compliance had no significant effect on fetal size and growth at any stage of pregnancy. Adding compliance to the regression models described above did not change the significance or the direction of the associations. |
| **Adverse events** | Stopping rules, which were based on adverse incidents (abortions, maternal deaths, preterm births, LBW, congenital abnormalities, stillbirths and infant deaths) were predetermined. | An independent data monitoring committee reviewed data on compliance, completeness of follow-up, pregnancy outcomes, and adverse events every 6 mo for the first 3 y of the trial and then every year. | See Potdar 2014 |
| **Engagement, category (including nutrition sensitive/specific)** | Nutrition specific | Nutrition specific | Nutrition specific |
| **Where in the overall UNICEF 2020 framework the intervention lies** | Intervention changing an the immediate determinant of Diet: snacks containing green leafy veg, milk and dried fruit are directly altering altering diet. | Intervention changing an the immediate determinant of Diet: snacks containing green leafy veg, milk and dried fruit are directly altering altering diet. | Intervention changing an the immediate determinant of Diet: snacks containing green leafy veg, milk and dried fruit are directly altering altering diet. |
| **Where in the Partap 2021 framework the intervention lies** | Giving snacks containing green leafy veg, milk and dried fruit is an individual-level nutrition intervention. | Giving snacks containing green leafy veg, milk and dried fruit is an individual-level nutrition intervention. | Giving snacks containing green leafy veg, milk and dried fruit is an individual-level nutrition intervention. |
| **Proposed mechanism of intervention action** |  | Not discussed | Enhancing maternal nutritional status by supplementing the mother’s diet with green leafy vegetables, milk, and fruit for a >= 3 months before conception (& throughout pregnancy) may reduce LBW by affectign processes such as de‐ and re‐methylation of fetal DNA, the development of the placenta, and fetal organogenesis in early gestation. These are important determinants of size at birth and long‐term health. |
| **Exposures relating to preconception nutrition** | Snacks as samosas, fritters, and fruit bars containing fresh and dried green leafy vegetables, milk, and dried fruit provided in local centres in afternoon and consumption observed . On average, treatment snacks contained 10–23% of the WHO/ FAO recommended Reference Nutrient Intake for b-carotene, riboflavin, folate, vitamin B-12, calcium, and iron; they contained 0.69 MJ energy and 6.4 g protein compared with 0.37 MJ and 2.4 g in control snacks. Control snacks did not have green leafy vegetables, milk, or dried fruit. | Snacks as samosas, fritters, and fruit bars containing fresh and dried green leafy vegetables, milk, and dried fruit provided in local centres in afternoon and consumption observed . On average, treatment snacks contained 10–23% of the WHO/ FAO recommended Reference Nutrient Intake for b-carotene, riboflavin, folate, vitamin B-12, calcium, and iron; they contained 0.69 MJ energy and 6.4 g protein compared with 0.37 MJ and 2.4 g in control snacks. Control snacks did not have green leafy vegetables, milk, or dried fruit. | Snacks as samosas, fritters, and fruit bars containing fresh and dried green leafy vegetables, milk, and dried fruit provided in local centres in afternoon and consumption observed . On average, treatment snacks contained 10–23% of the WHO/ FAO recommended Reference Nutrient Intake for b-carotene, riboflavin, folate, vitamin B-12, calcium, and iron; they contained 0.69 MJ energy and 6.4 g protein compared with 0.37 MJ and 2.4 g in control snacks. Control snacks did not have green leafy vegetables, milk, or dried fruit. |
| **Preconception measures** |  | See Potdar 2014 | At recruitment, women were asked about their occupation, education, religion, parity, and use of tobacco (in both smoked and chewed form).  Socio‐economic status was assessed using the standard of living index, based on housing type, utilities, and household possessions.  7-day Diet was assessed at recruitment using quantified FFQ. Weight & height |
| **Concommittant care** | Usual ANC and delivery care available to all. | See Potdar 2014 | See Potdar 2014 |
| **Follow-up schedule for measurements** | Health workers known to families made home visits to explain the trial and deliver information leaflets. Community meetings held to obtain community consent and answer questions. Recruitment camps were scheduled at which women were screened for eli gibility, and consented..  At enrolment recorded women’s education; occupations; socioeconomic status (Standard of Living Index), based on housing type, utilities, and household possessions; tobacco use, diet using a food-frequency questionnaire; weight and height. Women who missed 2 periods had a urinary pregnancy test, and if this test was positive, the women were invited to a central clinic at the CSSC at 9–13 wk of gestation for an obstetric assessment, hemoglobin measurement, and ultrasonography to confirm and date the pregnancy (18). Ultrasound scans were conducted by a single operator throughout the trial. At 27–33 wk, we repeated the hemoglobin measurement and performed an oral- glucose-tolerance test (WHO protocol) (19). Samples were analyzed in a single laboratory. Health workers were issued mobile phones, and families were asked to notify them when women went into labor. Health workers visited women were not attending for supplementation 3 times/wk from 36 wk of gestation. Deliveries took place in 140 institutions, (small private nursing homes to large government hospitals). Aimed to measure newborns within 72 h of birth ) but included measurements up to 10 d..  Trained research nurses measured weight (to the nearest 10 g; Seca scales; seca), crown-heel length to the nearest 0.1 cm, Rollameter; CMS Instruments), circumferences [occipitofrontal head, midupper arm, chest (xiphisternum level), abdomen (below the umbilicus)], and triceps and subscapular skinfold thicknesses. Circumferences were measured thrice to the nearest 0.1 cm by using fiberglass tapes and averaged. Skinfolds were measured thrice to the nearest 0.2 mm by using Holtain calipers (Holtain Ltd) and averaged. Pediatricians as sessed newborns for congenital abnormalities.  Gestational age was calculated from LMP unless different by greater than +/-14 d from that estimated by a <20-wk ultrasound scan (9%) when the latter was used. | Women were screened for eligibility, and individual written informed consent was obtained. We recorded education, occupation, and socioeconomic status with the use of the Standard of Living Index. Tobacco use was recorded. Diet was assessed with an FFQ, with the reference period the preceding week. Weight, height, and triceps and subscapular skinfolds were measured with the use of standardized techniques.  Women were offered an **oral-glucose-tolerance test (OGTT)** at 28–32 wk gestation because, although not a primary outcome, GDM status was an important covariate for the interpretation of supplementation effects on birth weight. The OGTT data enabled testing of whether the intervention benefited maternal metabolism as assessed by glucose tolerance. | Women screened for eligibility and individual written consent at recruitment clinics. At recruitment, women were asked about their occupation, education, religion, parity, and use of tobacco (in both smoked and chewed form). Socio‐economic status was assessed using the stan- dard of living index, (housing type, utilities, and household possessions). 7-day Diet was assessed at recruitment and in the second trimester of pregnancy using a quantified food frequency questionnaire (FFQ). Weight and height were measured. urinary pregnancy test adminstreted to women who missed two periods. if positive, they were invited to a central clinic at Centre for the Study of Social Change at 9–12 weeks gestation for an obstetric assessment and ultrasonography to confirm the pregnancy and measure fetal size. Further ultrasound scans were scheduled for 19–21 and 28–32 weeks gestation. Based gestational age on LMP date rather than ultra- sound measurements. Fetal biometry was measured using a Siemens Sonoline ADARA ultrasound machine with a 4‐MHz probe. At visit 1 (9–12 weeks), crown‐rump length (CRL) was measured. However, if women attended late and the gesta- tional age at the first examination was ≥13 weeks, fetal head circum- ference (HC), biparietal diameter (BPD), femur length (FL), and abdominal circumference (AC) were recorded instead. HC, BPD, FL, and AC were assessed at the two subsequent visits (19–21 and 28–32 weeks). Scans were carried out by a single operator (AL) throughout the trial. Trained research nurses measured newborns within 10 days of birth. Measurements included weight (to the nearest 10 g, Seca scales) and occipito‐frontal head circumference and abdominal circumference immediately below the umbilicus, each measured thrice to the nearest 0.1 cm using fibreglass tapes and averaged. |
| **Summary of baseline comparisons by study arm** | Baseline characteristics were similar in the 2 allocation groups (Table 3). At recruitment, 32% of women were underweight (BMI <18.5), and 14% of women were overweight or obese (BMI >25). One-third of women were nulliparous. Only 6 women smoked, and 10% of women chewed tobacco. Eighty-seven percent of women had completed at least a secondary education. Only 21% of women were in paid employment, mainly unskilled or semiskilled. Food-frequency questionnaire data collected at en rollment showed that women’s diets were monotonous, with low intakes of micronutrient-rich foods. One-half of the women had not consumed milk or milk products (eg, yogurt) in the preceding week other than in tea, and one-quarter of women had not consumed any green leafy vegetables. The majority of women (85%) had eaten fruit ,1 time/d, and more than one-quarter of women had con- sumed no meat or fish in the preceding week. | Women who did not have an OGTT were younger and of lower socioeconomic status and parity than women who attended. However, there were only small differences in characteristics between allocation groups; in women who had an OGTT, those in the treatment group had lower baseline weight, BMI, and subscapular skinfold thickness than did controls; they also had thinner skinfolds at visit 1, but greater pregnancy weight gain. | Baseline characteristics were mostly similar between women with complete data and those with one or more scan missing; differences were observed with respect to parity and occupation.  Women with one previous delivery had higher odds of having a full set of scans when compared to primiparous women (OR: 1.47, 95% CI [1.22, 1.78], p < .001). Nonworking women had lower odds of having a full set of scans than women engaged in paid work outside the home (OR: 0.71, 95% CI [0.58, 0.87], p = .001). |
| **Pregnancy outcomes: gestational weight gain, gestational diabetes, maternal anthropometry (weight, MUAC, height), anaemia,** | None reported | Outcomes for this analysis were  gestational diabetes mellitus (GDM),  fasting and 120-min glucose concentrations, and  fasting insulin concentration.  used the 1999 WHO definition of GDM int he trial but, in 2013, the definition changed (29) to match the International Association of Diabetes and Pregnancy Study Groups Consensus Panel recommendations (30) as follows:  - fasting glucose concentration of 5.1–6.9 mmol/L (92–125 mg/dL) and/or a 120-min glucose concentration of 8.5–11.0 mmol/L (153–199 mg/dL) and a category of ‘‘diabetes in pregnancy’’ was introduced [fasting glucose concentration $7.0 (126 mg/dL) and 120-min glucose concentration $11.1 mmol/L (200 mg/dL)]. We report results for both 1999 (27) and 2013 (29) criteria. | Fetal growth measures:  - crown‐rump length (CRL) at 9-12 weeks . However, if women attended late and the gesta- tional age at the first examination , At 2nd and 3rd visits 19–21 and 28–32 weeks gestation respectively (and on 1st visit if it was ≥13 weeks):  - fetal head circumference (HC),  - biparietal diameter (BPD),  - femur length (FL), and  - abdominal circumference (AC). |
| **Preconceptual outcomes: (BMI, Hb or other outcomes which could be attributed to preconception care)** |  |  |  |
| **Birth outcomes in child: birthweight, low birth weight (LBW), small for gestational age (SGA), preterm birth/ delivery (PTB), cord blood b12, DXA,** | Primary birth outcomes were birth weight and rates of low birth weight. Secondary birth outcomes were:  gestational age,  small for gestational age,  other newborn body measurements,  operative delivery rates,  intrauterine deaths or stillbirths,  major congenital malformations, and  twin or triplet pregnancies. | None | Not reported |
| **Outcomes in mother at birth** | None reported |  | None |
| **Post-natal outcomes (in Infancy)** | None | None | None |
| **Outcomes in childhood after 12 m: z scores for weight (WAZ), length/height (HAZ), and BMI for age (BMIZ), weight-for-height'length (WHZ), underweight, stunting, wasting, overweight, cognitive** | None | None | None |
| **Long term outcomes** | None | None | None |
| **Analysis** | Authors compared baseline measurements between allocation groups and between women who remained in the study or dropped out.  Intention-to-treat analysis compared newborn measurements between allocation groups in all women who were randomly assigned and became pregnant after starting supplementation  Per-protocol analysis that was limited to women supplemented >=90 d before their last menstrual period date. In both analyses, intrauterine deaths, stillbirths, twin or triplet pregnancies, and major congenital abnormalities were included only to compare their prevalence between groups and then excluded, and the analysis was limited to live singleton newborns measured within 10 d. Hypothesized, a priori, an interaction between the allocation group and maternal prepregnant BMI; in regression models, we used an interaction term (allocation group 3 BMI as a continuous variable), and for the presentation of interaction effects in tables and figures, we used categories (thirds) of maternal BMI (in kg/m2; <18.6, 18.6–21.8, and >21.8). We also tested for interactions with maternal age and height (continuous variables), parity (binary variable; 0 = primiparous, 1 = at least one previous delivery), and newborn sex (binary variable).  Small-for-gestational-age and large-for-gestational-age births were defined according to Oken et al (21) and preterm as gestation <37 wk.  Comparisons were made by using t tests, Mann-Whitney U tests, and chi-square or Fisher’s exact tests for normally distributed continuous, non- parametric, and categorical variables, respectively.  Primary results are reported unadjusted; authors used multiple regression analysis to assess intervention effects with adjustment for gestational age, infant sex, age of newborn measurement, maternal BMI, height, parity, age, socioeconomic status, gestational diabetes, education, compliance, and baseline food intakes. Finally, they examined the intervention effect on birth weight at different levels of maternal compliance. Results were considered statistically significant at P< 0.05. | Compared baseline measurements between women who did and did not have an OGTT, and between allocation groups.  Compared outcomes between allocation groups in all women who were randomly assigned, became pregnant and had an OGTT (intention-to-treat analysis), and limited to women who started supplementation >90 d before their LMP date (per-protocol analysis). We tested for interactions between allocation group and maternal age, BMI, height, and parity. Small-for-gestational-age and large-for-gestational-age births were defined in accordance with Oken and also ‘‘within-cohort’’ as <10th and >90th percentile based on singleton live births without major congenital abnormalities. Preterm birth was defined as gestation <37 wk. A t test, Mann-Whitney U test, or chi-square or Fisher's exact test was used to compare groups for normally distributed continuous, nonparametric, and categorical varibles, respectively; further comparisons of glucose concentrations between groups were made with the use of Kernel density estimates. Main results are reported unadjusted; authors then used multiple regression to assess intervention effects on GDM while adjusting for maternal age, adiposity (subscapular skinfold thickness and/or weight at recruitment and subscapular skinfold gain and/or weight gain from recruitment to 28 wk gestation), height, parity, socioeconomic status, and compliance. | Change in protocol to follow-up all pregnancies after Dec 2008 led to the implementation of two analyses: intention‐to‐treat (ITT) including all pregnancies, and and per‐protocol (PP) limited to women supplemented for 3 months or more before their LMP date. Excluded: twins (n = 26); fetuses with major congenital abnormalities (n = 12); ; missing LMP date (n = 69); pregnancies in which the LMP‐ derived gestation was >2 weeks different from gestation from <20 weeks ultrasound scan (n = 197); pregnant women with no delivery outcome (n = 22); newborns with missing information on sex (n = 41). Excluded all pregnancies resulting in abortions, terminations, stillbirths, and maternal deaths (n = 245) because of unknown fetal sex (illegal to reveal sex in pregnancy). Two preterm babies (<37 weeks of gestation) whose gestational‐age‐adjusted fetal measures, were >3 SDs higher than the population mean were excluded because LMP was likely incorrect. The exclusion criteria reduced the initial sample to 1,677 pregnancies in the ITT analysis and 1,335 pregnancies in the PP analysis. Among those 90% (ITT analysis: 1,508; PP analysis: 1,197) had one or more ultrasound measures.  Examined baseline differences in measurements between women who had three scans and those who had two or fewer scans. Calculated partial correlations among gestation‐adjusted fetal size measures whilst controlling for sex and allocation group.  Compared fetal biometry with the INTERGROWTH‐21st standards (Papageorghiou et al., 2014) by computing the z score of HC, FL, and AC in the second (14–27 weeks) and the third trimester (>27 weeks). To test the effect of the intervention on fetal size, considered each visit separately and used all available data at each visit. Because ges- tational age at the time of each visit varied between women, and fetal size differed between the sexes, within cohort sex‐and‐gestation‐specific z scores were calculated using the Lambda‐ Mu‐Sigma (LMS) method. The effect of the intervention on fetal growth was analysed using mixed effects regression models to take into account the correlation between repeated observations in the same individual and the possibility of a non- linear association between fetal biometry and gestational age. Unadjusted comparisons of fetal measures between allocation groups were made using t tests and Mann–Whitney U tests for normally and non‐normally distributed variables, respectively.  Multiple regression models assessed the effect of the inter-vention on fetal size and growth. The presence of interactions between allocation group and maternal pre‐pregnancy BMI, height and age (continuous variables), parity (discrete variable), and sex (binary variable) was evaluated. Interactions between allocation group and second trimester intakes of green leafy vegetables, fruit, and milk were considered when analysing the effect of the intervention on fetal size at visit 3. The effect of adjusting for compliance was also examined; for this purpose, average compliance was calculated from 3 months prior to the LMP (or from recruitment if supplementation <3 months) up to the visit of interest. Tobacco use was not included in the final set of adjustors because only 206 (9%) of pregnant women consumed tobacco (mostly in chewed form), and there were no associations between maternal tobacco use and fetal measurements. Women’s occupation, education, and standard of living index score were first included as possible confounders; however, as there were no associations between those variables and fetal measures, they were excluded from the models. Results were considered statistically significant when p < .05. |
| **EFFECTS on Preconception outcomes: maternal anthropometry (weight, MUAC, height. BMI), Haemoglobin (Hb), anaemia, iodine status** |  |  |  |
| **EFFECTS on Pregnancy outcomes: gestational weigt gain, gestational diabetes, maternal anthropometry (weight, MUAC, height), anaemia, iodine status** | None reported | 6513 nonpregnant women participated in the trial, of whom 2291 became pregnant. Of these, 241 had either an abortion or termination of pregnancy before 28 wk gestation, and they lost contact with 22 women.  Of the remaining 2028, 1008 (50%) attended for an OGTT at a median (IQR) gestation of 29.7 (29.3, 30.4) wk.  The prevalence of GDM (WHO 1999 criteria) was 9.9%. Both in the intention-to-treat and per-protocol analyses, the **prevalence was lower in the treatment group (intention-to- treat: 7.3% compared with 12.4%**, P-difference = 0.008; OR: 0.56; 95% CI: 0.36, 0.86; and **per-protocol: 7.5% compared with 13.1%, P = 0.01; OR: 0.54; 95% CI: 0.33, 0.86)**. This effect was independent of baseline and 28 wk skinfold measurements (Table 3) or baseline and 28-wk weight, or all of these measures combined. There was **no difference between treatment and control groups when we used the WHO 2013 GDM criteria** (29) (intention-to-treat: 8.9% compared with 11.1%, P = 0.27; OR: 0.79; 95% CI: 0.52, 1.20; and per-protocol: 9.1% compared with 11.2%, P = 0.32; OR: 0.79; 95% CI: 0.50, 1.26) or diabetes-in-pregnancy criteria. Moreover, there were no significant differences between allocation groups in mean fasting or 120-min glucose concentrations, or fasting insulin concentration. A Kernel density analysis explained these findings, as well as the discrepancy between 1999 (27) and 2013 (29) criteria. **There were more control women than treatment women with 120-min glucose concentrations in the range 7.5–10 mmol/L** (P-heterogeneity = 0.06 in frequencies in 3 glucose groups, including <7.5, 7.5–10.0, and >10.0). Frequencies of normal or very high glucose concentrations were similar in both allocation groups. There were **no significant interactions between allocation group and maternal age, BMI, height, or parity** in relation to any outcome. Women who developed GDM were older and more adipose than women who did not (1999 criteria). They had a similar prepregnancy intake of leafy green vegetables, fruit, and milk. Overall, 25% of women were vegetarian (ate no meat or fish); **women who developed GDM ate nonvegetarian foods more frequently** than women who did not develop GDM. One-third of women were anemic in early pregnancy and 17% were vitamin B-12 deficient, whereas only 1% were folate deficient; there were no differences in the prevalence of anemia or B-12 or folate deficiency between women who did and did not develop GDM. **There were more preterm births in the GDM group** (P = 0.002) and **fewer small-for-gestational-age births, as well as more congenital anomalies and emergency Cesarean sections (all borderline significant ;P = 0.1**). Findings were similar for the 2013 criteria. | Intention‐to‐treat analysis Unadjusted analyses: **no effect of the intervention on**: **Crown rump length** CRL at visit 1 (treatment mean CRL: −0.02SD; control mean CRL: 0.02SD; difference between means: 0.04SD, 95% CI [−0.08SD, 0.16SD]; p = .50) **Head circuference HC, Biparietal Diameter BPD, Femur Length FL, and Abdominal Circumference AC** at any of the visits separately.  No significant interactions between allocation group and maternal pre‐pregnancy BMI, height, age, or fetal sex.  At visit 2, there were **significant interactions between parity and allocation group** for **BPD (p = .02) and FL** (p = .04), becoming more positive as parity increased.  Fetal BPD and FL were smaller among primiparous women and larger among women with more than one previous delivery in the treatment group than in the control group.  **Supplemental snacks had no significant effect on growth of any of the fetal biometry measures considered**. There were no interactions between allocation group and maternal characteristics or fetal sex. Per‐protocol analysis findings were similar to the ITT analysis.  **No significant difference between allocation groups for CRL** at visit 1 (treat-ment mean CRL: −0.03SD; control mean CRL: 0.02SD; difference between means: 0.05SD, 95% CI [−0.09SD, 0.18SD]; p = .51)  **or for HC, BPD, FL, and AC at subsequent visits.** Significant interaction between allocation group and parity for FL at visit 2 (p = .03) but not for BPD.  **The intervention did not have a significant effect on growth of HC, AC, and FL in women with >=3 months of preconception supplementation**.  The median (interquartile range) gestational age at each examination was 10 (9–12), 19 (19–20) and 29 (28–30) weeks, respectively.  Of n = 1,677 fetuses included in the analysis nos with crown rump length (CRL) measured at each visit: visit 1- 1,151 (treatment: 67%, control: 71%); visit 2- 1,332 fetuses (treatment: 74% control: 80%); visit 3- 1,233 (treatment: 73%, control: 75%). 1105 (treatment: 47%, control: 49%) women had three scans recorded, 471 (treatment: 22%, control: 19%) had two, and 223 (treatment: 10%, control: 10%) had only a single scan.  Partial correlation coefficients among gestation‐adjusted fetal and newborn measures were positive and statistically significant: fetuses who were larger in early gestation tended to be larger in the later stages of pregnancy and at birth. Compared to the INTERGROWTH‐21st standards, fetal Abdominal Circumference (AC) was significantly smaller in both the 2nd and 3rd trimesters (−1.21SD and −1.26SD, respectively), whereas Head Circumference (HC) (−0.03SD and 0.03SD, respectively) and Femur Length (FL) (0.31SD and 0.36SD, respectively) were more comparable. |
| **EFFECTS on Birth outcomes in child: birthweight, low birth weight (LBW), small for gestational age (SGA), preterm birth/ delivery (PTB)** | Achieved birth weights within 72 h in 77% of births, treatment: 76% of births; control: 77% of births. Median (IQR) age at measurement was 45 h (24–81 h) [treatment: 47 h (25–84 h); control: 45 h (23–79 h)]. In unadjusted analyses, the median (IQR) gestational age was 39.0 wk (37.9–40.0 wk) and 39.1 wk (38.0–40.0 wk) in treat- ment and control groups, respectively (P-difference = 0.50). No significant differences between allocation groups in - birth weight (treatment: 2624 g; control: 2598 g; P = 0.22; +26 g; 95% CI: -15, 68 g); ],  - percentages of LBW (treatment: 34%; control: 39%; OR: 0.83; 95% CI: 0.66, 1.05; P = 0.10),  - small-for-gestational age-births (treatment: 67%; control: 69%, OR: 0.89; 95% CI: 0.70, 1.13; P = 0.33),  - large- for-gestational-age births (treatment: 0.5%; control: 0.4%; P = 1.0), or  - preterm births (treatment: 13%; control: 12%; P = 0.60). Interactions found between the allocation group and maternal prepregnant BMI for birth weight (P-interaction , 0.001) and other newborn measurements such that the intervention effect was greater in mothers of higher BMI. Percentages of LBW infants in thirds of maternal BMI were as follows:  maternal BMI ≤18.6: treatment: 44%; control: 44% (OR: 0.99; 95% CI: 0.68, 1.45);  maternal BMI from 18.6 to 21.8: treatment: 32%; control: 39% (OR: 0.74; 95% CI: 0.49, 1.10);  maternal BMI >21.8: treatment: 25%; control: 32% (OR: 0.69; 95% CI: 0.44, 1.08) (P-interaction = 0.008). Results were similar for gestation-adjusted birth measurements (not shown) and in the regression analysis adjusted for other factors influencing birth size. There were more male than female newborns (54% compared with 46% in both allocation groups). Elective cesarean delivery, emergency cesarean delivery, and forceps/ventouse rates did not differ between intervention groups (treatment: 9.5%, 11.9% and 0.6%, respectively; control: 10.2%, 11.2%, and 1.0%, respectively). There were 25 singleton intrauterine deaths or stillbirths (Figure 1; treatment: 1.2% of pregnancies; control: 1.0% of pregnancies); 12 major congenital abnor-malities (major heart defects, neural tube defects, skeletal dysplasias, and Downs syndrome; treatment: 0.5%; control: 0.6%); and 26 twin and triplet pregnancies (treatment: 1.3%; control: 1.0%). Numbers for all outcomes were similar in both allocation groups. Per-protocol analysis Of 2291 pregnant women, 1826 women started supplemen tation >90 d before their last menstrual periods: In unadjusted analyses, the median (IQR) gestation was 39.1 wk (38.0–40.0 wk) in both allocation groups.  Birth weight was higher in the treatment group by 48 g (95% CI: 1, 96 g; P = 0.046).  Percentages of LBW and small-for-gestational- age births were lower [LBW: treatment: 34%; control: 41%; OR: 0.76; 95% CI: 0.59, 0.98 (P = 0.03);  small-for-gestational-age: treatment: 66%; control: 71%; OR: 0.80; 95% CI: 0.61, 1.04 (P = 0.09)].  % large-for-gestational-age infants (treatment: 0.6%; control: 0.5%; P = 1.0) and preterms (treatment: 12.7%; control: 12.3%; P = 0.87) were similar in both groups.  Found interactions between the allocation group and maternal prepregnant BMI for birth weight (P-interaction = 0.001) and other newborn measurement. In the highest one-third of maternal BMI, birth weight increased by 113 g (95% CI: 29, 197 g), birth length increased by 0.3 cm (95% CI: -0.2, 0.9 cm), chest circumference increased by 0.6 cm (95% CI: 0.2, 1.1 cm), midupper arm circumference increased by 0.2 cm (95% CI: 0.1, 0.4 cm), and triceps skinfold thickness increased by 0.2 mm (95% CI: -0.1, 0.5 mm).  % LBW in thirds of maternal BMI were as follows -lowest: treatment: 45%; control: 46% (OR: 0.95; 95% CI: 0.62, 1.44);  -middle: treatment: 31%; control: 42% (OR: 0.63; 95% CI: 0.40, 0.99); and  -highest: treatment: 24%; control: 34% (OR: 0.61; 95% CI: 0.36, 1.01) (P- interaction = 0.01).  Results were similar for gestation-adjusted birth measurements % in the regression analysis. Elective and emergency cesarean delivery and forceps/ ventouse rates were similar in both allocation groups (treatment: 9.6%, 11.3%, and 0.6%, respectively; control: 10.1%, 10.7%, and 0.9%, respectively).  21 singleton intrauterine deaths or stillbirths ( treatment: 1.4%; control: 0.9%), 10 major congenital abnormalities (treatment: 0.5%; control: 0.6%), and 23 twin and triplet pregnancies (treatment: 1.5%; control: 1.0%).  Numbers for all other outcomes were similar in both allocation groups. There were no consistent interactions between the allocation group and maternal age, parity, height, or newborn sex in relation to any of the outcomes in either the intention-to-treat or per- protocol analyses. | None | NA |
| **EFFECTS on Outcomes in mother at birth** | None reported | None | NA |
| **EFFECTS on Post-natal outcomes (in Infancy)** | None | None | NA |
| **EFFECTS on Outcomes in childhood after 12 m: z scores for weight (WAZ), length/height (HAZ), and BMI for age (BMIZ), weight-for-height'length (WHZ), underweight, stunting, wasting, overweight, cognitive** | None | None | NA |
| **Potential Biological mediating factors (as identified by authors of the primary studies)** | The food supplement appeared to have larger effects on the newborn size in mothers of higher BMI. This effect was the opposite of what authors hypothesize . Therefore, this result could have been a chance finding and should be interpreted with caution. The result was opposite to the effect seen in trials of protein-energy supplementation in pregnancy, which increases birth weight more in undernourished women. However, the result was consistent with data from multiple micronutrient trials in pregnancy; a meta-analysis of individual-level data from 12 trials showed an interaction between the allocation group and maternal BMI with a greater birth-weight effect in mothers of higher BMI in 11 of the trials.  The authors speculate that underweight mothers may have had inadequate macronutrients or other substrates to use nutrients supplied by our supplements or partition them to the fetus. A nutritional intervention will only have a benefit up to the point at which other nutrient deficiencies become limiting. The metabolism of nutrients, development of the fetal supply line, transportation of nutrients across the placenta, and fetal growth require energy and other substrates. Our interpretation of the BMI interaction is that extra macronutrients, in addition to micronutrients, may be needed for underweight women in low- and middle-income countries. The effect of the supplement on birth weight may have resulted from micronutrients in the snacks or other important compounds (eg, fatty acids) in foods. Observational studies in high-income populations have linked higher maternal intakes of cow milk with higher birth weight. To our knowledge, there are no equivalent data for green leafy vegetables or fruit. The effect may have resulted from the higher energy (+0.32 MJ) and/or protein (+4 g) in treatment than control snacks. However, trials that showed an effect of protein and energy supplementation on birth weight used more (2–10 times) energy and protein than were present in our treatment snacks. | Limited information on women's micronutrient status, limits the authors' ability to suggest mechanisms for the reduction in GDM. A separate study on a range of micronutrients before and after 3 mo of supplementation with the snack (vitamin C, b-carotene, retinol, ferritin, folate, vitamin B-12, and homocysteine) and found that, of these, only b-carotene concentrations increased.  There is a possibility that the interventtion did not prevent GDM in the treatment group, but that the control snacks, which were lower in protein, increased the risk of GDM. This seems unlikely, because the control snacks contained less energy than the intervention snacks, and women in the control group did not gain more fat than those in the intervention group.  In a predominantly vegetarian population with a very low baseline intake of leafy green vegetables, fruit, and milk, the daily snack made a substantial difference in the intake of these foods. Observational studies have shown that a higher ratio of polyunsaturated to saturated dietary fat, higher intake of carbohydrates relative to fat, and higher vitamin B-12, C, and D status are associated with a lower risk.  We do not know which constituents of the snacks produced the effect. The main differences between the snacks were the fillings (leafy green vegetables, fruit, and milk in the intervention snacks compared with low-micronutrient vegetables in the control snacks). Other nutrients in the snacks came from the covering/binding ingredients and the cooking oil, which were similar in both groups, although the former were greater in quantity in the intervention snacks, resulting in 0.32 MJ more energy on average and 4 g more protein per snack. Leafy green vegetables contain the antioxidants b-carotene, vitamin C, and polyphenols. However, these have not prevented T2DM in randomized trials. Leafy green vegetables are rich in magnesium, a higher intake of which has been associated with a lower risk of T2DM and which reduce fasting glucose in trials. The effect may be from Fatty Acids; leafy green vegetables are a rich source of long-chain v-3 PUFAs, which may improve insulin sensitivity by influencing the properties of cell membranes. Leafy green vegetables also contain nitrates, which increase thermogenesis, oxygen consumption, and b-oxidation in rat adipocytes. The association between higher dairy intake and lower risk of future T2DM has been attributed to calcium, vitamin D, or whey protein . The different results from the 1999 and 2013 diagnostic criteria were explained by the fact that fewer women in the treatment group had 120-min glucose values in a middle impaired glucose tolerance range than controls. The authors' interpretation of the findings is that the supplement had no effect on glucose concentrations in women with normal glucose tolerance or in those with established diabetes in pregnancy, but that there was an intermediate group of women who were vulnerable to diabetes and whose metabolic competence was improved by the supplement. | Potdar 2014 showed that the supplement increased birth weight and other “soft tissue” measurement (skinfolds and abdominal, mid‐upper arm and chest circumference) in the newborns of mothers supplemented for ≥3 months before pregnancy but had no effect on “bony measurements” (length and head circumference). Independently of length of supplementation, the effects on birth weight and soft tissue measurements were modified by maternal pre‐pregnancy BMI (there were greater effects of the supplementation among women of normal or high BMI). In contrast, this analysis in Lawande 2018 did NOT detect an effect on ultrasound measures of fetal size and growth, and there was no evidence of an interaction between allocation group and BMI.  These differences between fetal and newborn findings may be partially explained by the nature of the ultrasound measures. HC, BPD, and FL are measures of bone size. AC, the only soft tissue measure available, is characterised by high variability and can be distorted by the transducer. Fetal growth during late gestation (>33 weeks) might also explain the differences as this was not measured. Authors found an interaction between intervention group and parity in relation to fetal BPD and FL at visit 2 (19–21 weeks). Among primiparous women, fetal size was smaller in the intervention group than in controls, and the opposite was true among women with more than one previous delivery. Since there were no similar interactions at visit 1 (9–12 weeks) or visit 3 (28–32 weeks), this suggests that fetal growth was slower in the intervention group in early pregnancy among women of lower parity, but “caught up” between visits 2 and 3, or that it was faster in early pregnancy among women of higher parity and became slower between visits 2 and 3. The observed effect should be interpreted with caution as it might be a chance finding. Birth weight increased with parity as expected, with the greatest increase in means between first and second births (results not shown). |
| **Potential Behavioural mediating factors (as identified by authors of the primary studies)** |  | None given |  |
| **Potential Social mediating factors (as identified by authors of the primary studies)** |  | None given |  |
| **Details on who interventions are available to, and who is accessing services and in what numbers** | A total of 1447 women (treatment: 22%; control: 23%) stayed in the trial throughout but never became pregnant (Figure 1). An additional 2243 women (treatment: 35%; control: 34%) dropped out before becoming pregnant either because they moved away (usually because of slum redevelopment), declined additional follow-up, died, separated from their husbands, or were sterilized. Women who stayed in the study were better educated, of higher socioeconomic status, and had lower meat and fish intakes than did women who dropped out but did not differ between allocation groups. | See Potdar 2014 |  |
| **Author reflections on factors affecting the success of preconception nutrition interventions, including factors relating to leadership, financing, governance, supplies, and capacity** |  |  | In a food‐based randomised controlled trial among Indian women living in Mumbai slums, a daily micronutrient‐rich snack eaten preconceptionally and throughout pregnancy had no effect on ultra- sound measures of fetal size or growth. At visit 2 (19–21 weeks), BPD and FL were significantly influenced by an interaction between alloca-tion group and parity, with the supplement having a greater positive effect in fetuses of multiparous women. Fetal measures were positively correlated throughout gestation with the highest correlations observed. |
| **Author reflections on factors affecting the failure of preconception nutrition interventions, including factors relating to leadership, financing, governance, supplies, and capacity** | Full blinding was impossible, and if women thought they were getting the healthier snack, this belief could have modified their behaviors in other ways fa- voring a better pregnancy outcome. However, this effect would not explain the BMI interaction.  Several factors could have attenuated the intervention effect. Thirty-two percent of newborns were not measured, which reduced the sample size. Only 40–50% of women were fully compliant, and compliance was lower in the treatment group than control group. Pregnancy outcomes may have improved in both groups; both groups re- ceived antenatal monitoring and encouragement to take iron plus folate supplements. We had no baseline population-level birth- weight data in this community to assess this possibility. Control women may have increased their habitual intakes of green leafy vegetables, although such an increase was not supported by serial food-frequency questionnaire data (not shown).  We did not find larger effects on birth weight in women who ate more supplements; indeed, the intention-to-treat analysis showed a smaller effect on birth weight in fully compliant women. We are unable to explain this result. The finding could reflect confounding; the most-deprived women may have been hungrier and, therefore, more compliant and also had smaller infants. However, adjustment for multiple confounders did not alter the effect. There may have been contaminants (eg, pesticides) in the green leafy vegetables, which became toxic at higher intakes, but we washed the fresh leaves thoroughly. We speculate that, because women were most compliant in the first weeks and months of supplementation, women who were in the study for longer may have benefited from supplementation over a longer period even if their compliance was lower immediately before and during pregnancy (the period used to define compliance). | None |  |
| **Author recommendations on strategies to improve preconception nutrition in South Asia** | the findings from this trial clearly do not have any immediate implications for policies to improve maternal nutritional status and prevent LBW. The effect on birth weight was significant only in the per-protocol analysis and would need to be replicated to have confidence that it is real. The effect was modest but similar in magnitude to that achieved by using multiple micronutrient supplements in pregnancy, which suggests that food-based approaches may have a role. The effect was apparently present only in mothers who were not underweight. This finding suggests that women of different nutritional status may need different interventions, which could be extremely challenging in a programmatic setting. | The results of this randomized, controlled trial suggest that improving women's dietary micronutrient quality may have important protective effects against GDM. Because GDM was not the trial's primary outcome and because of 50% nonparticipation for the OGTT, the findings would need to be replicated. However, they are consistent with observational research showing a lower risk of GDM and T2DM in association with the foods contained in the trial supplements |  |
| **Author recommendations on future research to improve preconception nutrition in South Asia** | The effect on birth weight was significant only in the per-protocol analysis and would need to be replicated to have confidence that it is real.  The effect in mothers who were not underweight suggests that women of different nutritional status may need different interventions, which could be extremely challenging in a programmatic setting. However, it is a potentially important finding which, if replicated, needs to be understood biologically. The authors are following up the children born during the trial to assess whether this intervention, which covered the periconceptional period (and, therefore, epigenetic changes) and the first trimester (and, therefore, organogenesis), has any longer-term functional and health effects in the offspring. The results of these studies will determine whether this intervention is worth pursuing further. | findings add to the debate about diagnostic cutoffs, and perhaps make a case for maintaining the impaired glucose tolerance range within the criteria for GDM | To understand whether improving maternal nutrition has a significant effect on fetal growth, additional ultrasound measures of fetal soft tissues, such as mid‐thigh muscle thickness and abdominal subcutaneous tis-sue may be informative. |
| **Barriers to Preconception nutritional intake** | Barriers to intake not mentioned but baseline description says: Food-frequency questionnaire data collected at enrollment showed that women’s diets were monotonous, with low intakes of micronutrient-rich foods. 50% had not consumed milk or milk products (eg, yogurt) in the preceding week other than in tea, and 25% had not consumed any green leafy vegetables. 85% had eaten fruit <1 time/d, and >25% consumed no meat or fish in the preceding week. | Food frequency questionnaires used but results not reported. |  |
| **Enablers to preconception nutritional intake** | None mentioned | Food frequency questionnaires used but results not reported. |  |
| **Risk of Bias assessment ROB2 completed?** |  |  |  |
| **ABSTRACT** | Background: Low birth weight (LBW) is an important public health problem in undernourished populations. Objective: authors tested whether improving women’s dietary micro- nutrient quality before conception and throughout pregnancy in- creases birth weight in a high-risk Indian population. Design: The study was a nonblinded, individually randomized con- trolled trial. The intervention was a daily snack made from green leafy vegetables, fruit, and milk (treatment group) or low-micronutrient vegetables (potato and onion) (control group) from $90 d before pregnancy until delivery in addition to the usual diet. Treatment snacks contained 0.69 MJ of energy (controls: 0.37 MJ) and 10–23% of WHO Reference Nutrient Intakes of b-carotene, riboflavin, folate, vitamin B-12, calcium, and iron (controls: 0–7%). The primary out- come was birth weight. Results: Of 6513 women randomly assigned, 2291 women became pregnant, 1962 women delivered live singleton newborns, and 1360 newborns were measured. In an intention-to-treat analysis, there was no overall increase in birth weight in the treatment group (+26 g; 95% CI: 215, 68 g; P = 0.22). There was an interaction (P , 0.001) between the allocation group and maternal prepregnant body mass index (BMI; in kg/m2) [birth-weight effect: 223, +34, and +96 g in lowest (,18.6), middle (18.6–21.8), and highest (.21.8) thirds of BMI, respectively]. In 1094 newborns whose mothers started supplementation $90 d before pregnancy (per- protocol analysis), birth weight was higher in the treatment group (+48 g; 95% CI: 1, 96 g; P = 0.046). Again, the effect increased with maternal BMI (28, +79, and +113 g; P-interaction = 0.001). There were similar results for LBW (intention-to-treat OR: 0.83; 95% CI: 0.66, 1.05; P = 0.10; per-protocol OR = 0.76; 95% CI: 0.59, 0.98; P = 0.03) but no effect on gestational age in either analysis. Conclusions: A daily snack providing additional green leafy vege- tables, fruit, and milk before conception and throughout pregnancy had no overall effect on birth weight. Per-protocol and subgroup analyses indicated a possible increase in birth weight if the mother was supplemented $3 mo before conception and was not under- weight. This trial was registered at www.controlled-trials.com/isrctn/ as ISRCTN62811278. Am J Clin Nutr 2014;100:1257–68. | Background: Prospective observational studies suggest that maternal diets rich in leafy green vegetables and fruit may help prevent gestational diabetes mellitus (GDM). Objective: Our objective was to test whether increasing womenÕs dietary intake of leafy green vegetables, fruit, and milk before conception and throughout pregnancy reduced their risk of GDM. Methods: Project SARAS (‘‘excellent’’) (2006–2012) was a nonblinded, individually randomized, controlled trial in women living in slums in the city of Mumbai, India. The interventions included a daily snack made from leafy green vegetables, fruit, and milk for the treatment group or low-micronutrient vegetables (e.g., potato and onion) for the control group, in addition to the usual diet. Results for the primary outcome, birth weight, have been reported. Women were invited to take an oral-glucose-tolerance test (OGTT) at 28–32 wk gestation to screen for GDM (WHO 1999 criteria). The prevalence of GDM was compared between the intervention and control groups, and Kernel density analysis was used to compare distributions of 120-min plasma glucose concentrations between groups. Results: Of 6513 women randomly assigned, 2291 became pregnant; of these, 2028 reached a gestation of 28 wk, 1008 (50%) attended for an OGTT, and 100 (9.9%) had GDM. In an intention-to-treat analysis, the prevalence of GDM was reduced in the treatment group (7.3% compared with 12.4% in controls; OR: 0.56; 95% CI: 0.36, 0.86; P = 0.008). The reduction in GDM remained significant after adjusting for prepregnancy adiposity and fat or weight gain during pregnancy. Kernel density analysis showed that this was explained by the fact that fewer women in the treatment group had a 2-h glucose concentration in the range 7.5–10.0 mmol/L. Conclusions: In low-income settings, in which women have a low intake of micronutrient-rich foods, improving dietary micronutrient quality by increasing intake of leafy green vegetables, fruit, and/or milk may have an important protective effect against the development of GDM. This trial was registered at www.controlled-trials.com as ISRCTN62811278. J Nutr 2016;146(Suppl):1453S–60S. | Improving micronutrient intakes of under‐nourished mothers in low‐ and middle‐income countries increases birth weight, but there is little data on the nature and timing during gestation of any effects on fetal growth. Ultrasound measures of fetal size were used to determine whether and when a food‐based supplement affected fetal growth. Non‐pregnant women living in Mumbai slums, India (N = 6,513), were randomly assigned to receive either a daily micronutrient‐rich snack containing green leafy vegetables, fruit, and milk (treatment) or a snack made from lower‐micronutrient vegeta- bles (control) in addition to their usual diet from before pregnancy until delivery. From 2,291 preg- nancies, the analysis sample comprised 1,677 fetuses (1,335 fetuses of women supplemented for ≥3 months before conception). First‐trimester (median: 10 weeks, interquartile range: 9–12 weeks) fetal crown‐rump length was measured. Fetal head circumference, biparietal diameter, femur length, and abdominal circumference were measured during the second (19, 19–20 weeks) and third trimes- ters (29, 28–30 weeks). The intervention had no effect on fetal size or growth at any stage of pregnancy. In the second trimester, there were interactions between parity and allocation group for biparietal diameter (p = .02) and femur length (p = .04) with both being smaller among fetuses of primiparous women and larger among those of multiparous women, in the treatment group compared with the controls. Overall, a micronutrient‐rich supplement did not increase standard ultrasound measures of fetal size and growth at any stage of pregnancy. Additional ultrasound measures of fetal soft tissues (fat and muscle) may be informative. |

| **SN** | **15** | **16** |
| --- | --- | --- |
| **Short name** | Taneja2022 | Taneja2020 |
| **Type of paper** | Trial findings | Trial protocol |
| **Authors** | Sunita Taneja, Ranadip Chowdhury, Neeta Dhabhai, Ravi Prakash Upadhyay, Sarmila Mazumder, Sitanshi Sharma, Kiran Bhatia, Harish Chellani, Rupali Dewan, Pratima Mittal, MK Bhan, Rajiv Bahl, Nita Bhandari, on behalf of the WINGS Study Group | Taneja, S., R. Chowdhury, N. Dhabhai, S. Mazumder, R. P. Upadhyay, S. Sharma, R. Dewan, P. Mittal, H. Chellani, R. Bahl, M. K. Bhan, N. Bhandari, Women and G. Infants Integrated Growth Study |
| **Year** | 2022 | 2020 |
| **Title** | Impact of a package of health, nutrition, psychosocial support, and WaSH interventions delivered during preconception, pregnancy, and early childhood periods on birth outcomes and on linear growth at 24 months of age: factorial, individually randomised controlled trial | Impact of an integrated nutrition, health, water sanitation and hygiene, psychosocial care and support intervention package delivered during the pre- and peri-conception period and/or during pregnancy and early childhood on linear growth of infants in the first two years of life, birth outcomes and nutritional status of mothers: study protocol of a factorial, individually randomized controlled trial in India. |
| **Citation** | Taneja S, Chowdhury R, Dhabhai N, Upadhyay RP, Mazumder S, Sharma S, Bhatia K, Chellani H, Dewan R, Mittal P, Bhan MK. Impact of a package of health, nutrition, psychosocial support, and WaSH interventions delivered during preconception, pregnancy, and early childhood periods on birth outcomes and on linear growth at 24 months of age: factorial, individually randomised controlled trial. bmj. 2022 Oct 26;379. | Taneja, S., R. Chowdhury, N. Dhabhai, S. Mazumder, R. P. Upadhyay, S. Sharma, R. Dewan, P. Mittal, H. Chellani, R. Bahl, M. K. Bhan, N. Bhandari, Women and G. Infants Integrated Growth Study (2020). "Impact of an integrated nutrition, health, water sanitation and hygiene, psychosocial care and support intervention package delivered during the pre- and peri-conception period and/or during pregnancy and early childhood on linear growth of infants in the first two years of life, birth outcomes and nutritional status of mothers: study protocol of a factorial, individually randomized controlled trial in India." Trials 21(1): 127. |
| **doi** | doi: dx.doi.org/10.1136/ | https://doi.org/10.1186/s13063-020-4059-z |
| **Clinical trials registration no** | Clinical Trial Registry—India CTRI/2017/06/008908. | Clinical Trial Registry – India #CTRI/2017/06/008908; Registered 23 June 2017, http://ctri.nic.in/ Clinicaltrials/pmaindet2.php?trialid=19339&EncHid=&userName=society%20for%20applied%20studies |
| **Context / setting** | Low and middle income neighbourhoods of Delhi, India. | Low and middle income neighbourhoods of Delhi, India. |
| **Protocol citation** | Taneja S, Chowdhury R, Dhabhai N, Mazumder S, Upadhyay RP, Sharma S, Dewan R, Mittal P, Chellani H, Bahl R, Bhan MK, Bhandari N; Women and Infants Integrated Growth Study (WINGS) Group. Impact of an integrated nutrition, health, water sanitation and hygiene, psychosocial care and support intervention package delivered during the pre- and peri-conception period and/or during pregnancy and early childhood on linear growth of infants in the first two years of life, birth outcomes and nutritional status of mothers: study protocol of a factorial, individually randomized controlled trial in India. Trials. 2020 Jan 31;21(1):127. doi: 10.1186/s13063-020-4059-z. | this is the protocol |
| **Arising from which main trial** | The Women and Infants Integrated Interventions for Growth Study (WINGS) | The Women and Infants Integrated Interventions for Growth Study (WINGS) |
| **Study design** | Individually randomised factorial trial. Used a factorial design to examine the impact of the package when delivered only in the preconception period, and the combined effect of implementation of the package in preconception, pregnancy, and early childhood periods. The design also enabled us to examine the effect of maternal height reflecting intergenerational adversities and genetics on the efficacy of interventions to improve birth and child outcomes. | individually randomized controlled trial with factorial design |
| **Has Preconception intervention only arm (1/0)** | 1 | 1 |
| **Has Preconception + Pregnancy intervention arm (1/0)** | 1 | 1 |
| **Has Pregnancy intervention only arm (1/0)** |  |  |
| **Has Pregnancy, postpartum + childhood intervention arm (1/0)** | 1 | 1 |
| **Has Control arm with only usual standard of care or less intensive intervention in preconception & pregnancy (1/0)** | 1 | 1 |
| **MMN in Preconception arm** |  |  |
| **B12 in Preconception arm** |  |  |
| **LNS in Preconception arm** |  |  |
| **Viatmin A in preconception arm** |  |  |
| **Iron in preconception arm (both arms had Folic acid)** |  |  |
| **Nutritious snack in Preconception arm** |  |  |
| **Pregnancy outcomes** |  |  |
| **Birth outcomes** |  | 1 |
| **Infancy outcomes** |  | 1 |
| **Child outcomes** |  | 1 |
| **Narrative summary of main finding** | Preconception women randomised to preconception interventions or routine care. After pregnancy, pregnant women were randomised again to receive pregnancy and early childhood interventions or routine care. Preconception, pregnancy, and early childhood interventions (group A), preconception intervention (group B), pregnancy and early childhood interventions (group C), and control (group D).  Interventions: Health, nutrition, psychosocial care and support, and WaSH interventions were delivered during preconception, pregnancy, and early childhood periods. Nutrition intervention = at preconception: Provide IFA and MMN; provide egg or milk if body mass index <21; screen and treat malnutrition, anaemia. in pregnancy arm continue in pregnancy with different snacks depending on nutritional status. Add calcium and Vit D in postpartum and food supplement at 6-11 m for kids in early childhood arm. Main Outcome Measures: The primary outcomes were low birth weight, small for gestational age, preterm, and mean birth weight. At 24 months, the outcomes were mean length-for-age z scores and proportion stunted. Three prespecified comparisons were made: preconception intervention groups (A+B) versus no preconception intervention groups (C+D); pregnancy and early childhood intervention groups (A+C) versus routine care during pregnancy and early childhood (B+D) and preconception, pregnancy, and early childhood interventions groups (A) versus control group (D). intervention package delivered during preconception, pregnancy, and early childhood substantially reduced low birth weight and stunting at 24 months. Pregnancy and early childhood interventions alone had lower but important effects on birth outcomes and 24 month outcomes. Preconception interventions alone had an important effect on birth outcomes but not on 24 month outcomes.  Preconception interventions had important effects on birth size but not on linear growth at 24 months compared with the control group. Pregnancy and early childhood interventions had important effects on birth size and on linear growth at 24 months compared with the control group. The effect sizes of preconception and pregnancy and early childhood interventions together were larger for birth size and linear growth at 24 months than those of the control group compared with the effect sizes of interventions provided only in a single period.  In this trial, **a package of health, nutrition, psychosocial care, and WaSH interventions delivered during preconception and pregnancy periods reduced the risk of LBW by 24%, more than half of which was attributed to preconception interventions.** The intervention package delivered during preconception, pregnancy, and early childhood reduced the risk of stunting at two years of age by 51%; almost all the effect can be attributed to pregnancy and early childhood interventions. These intervention effects were not modified by maternal height. In addition to the effect on primary and secondary infant outcomes, the interventions improved several maternal outcomes—higher haemoglobin concentration and gestational weight gain, and reduced risk of reproductive tract infection, anaemia, and pregnancy induced hypertension. | No findings this is the protocol Summary of arms: Group A: Pre- and peri-conception, enhanced antenatal (pregnancy), postnatal and early childhood interventions  Group B: pre- and peri-conception intervention only (routine care in pregnancy postpartum/early childhood),  Group C: pregnancy and early childhood interventions (no pre-/peri-conception intervention)  Group D: control (no pre-/peri-conception intervention and routine care in pregnancy postpartum/early childhood). |
| **Date of data collection** | Women screened between 1 July 2017 and 30 December 2019. Data collection for primary outcomes ended on 30 June 2021.  Children who reached 24 months of age by 30 June 2021 (DSMC decision) were included in the analysis for 24 month outcomes |  |
| **Study objectives** | To determine the effect of integrated and concurrent delivery of health, nutrition, water, sanitation and hygiene (WaSH), and psychosocial support/care interventions during the preconception period alone, during pregnancy and early childhood, and throughout preconception, pregnancy, and early childhood on birth outcomes ( LBW) and linear growth at 24 months of age compared with routine care. | 1) to determine the effect of integrated and concurrent delivery of interventions to improve health, nutrition, WASH and psychosocial status during the pre- and peri-conception period alone (pre- and peri-conception intervention package); during pregnancy and early childhood (enhanced antenatal, postnatal and early childhood care) and; throughout the pre- and peri-conception period, pregnancy and early childhood, on preterm birth, LBW and SGA, and stunting at 24 months of age compared to routine care  1b) to assess whether the effect of interventions differs by maternal stature (< 150 cm or ≥ 150 cm). 2) to determine the effect of the same package on nutritional status, morbidity and neurodevelopment in children, and on women’s nutritional status and morbidity in the pre- and peri-conception, pregnancy and postpartum periods. |
| **Sample size / power** | Sample sizes were calculated for 90% power (80% for preterm birth) and 95% confidence interval for comparison between groups. authors assumed at least 0.15 standard deviation mean difference for birth weight or length and length-for-age z score at 24 months, 25% relative reduction for LBW, preterm birth and SGA, and stunting at 24 months for the impact of preconception (groups A+B v C+D) or pregnancy and early childhood interventions (groups A+C v B+D). authors used 0.20 standard deviation mean difference for birth weight or length, length-for-age z score at 24 months, 30% relative reduction for LBW, preterm birth, SGA, and stunting at 24 months for the combined effect of preconception and pregnancy and early childhood interventions compared with the control group (group A v D). authors aimed for 1100 live births (based on the outcome with the largest sample size) and 600 children at 24 months in each of the four groups. To achieve 4400 live births, authors enrolled 13 500 women in the preconception period. DSMB recommended stopping the study based on strong evidence for the beneficial effects for 24 month outcome so sample size not achieved at 24m. | Sample sizes were calculated for 90% power and 95% confidence level except for the preterm birth outcome for which the power is 80% for comparison between effect of pre- and peri-conception intervention package and enhanced antenatal, postnatal and early childhood care. Larger effect sizes than those shown for single interventions were assumed for the combined effect of pre- and peri-conception intervention package, enhanced antenatal, postnatal and early childhood care group [14–16]. A 1.5 times higher effect size for the impact of either pre- and peri-conception intervention package or enhanced antenatal, postnatal and early childhood care compared with control, and at least 2 times higher for combined effect of pre- and peri-conception intervention package and en- hanced antenatal, postnatal and early childhood care compared to control was assumed. Proposed to enrol 13,500 eligible women (6750 in pre- and peri-conception intervention package group and 6750 in control group) based on the following assumptions:  45% of reproductive-age women randomized get pregnant in the 18 months of the pre- and peri- conception intervention period, 30% loss (abortions, still births, maternal deaths, moving away, refusals) between pregnancy and live birth and 20% loss (loss-to-follow-up and child deaths) between birth and 24 m of age.  ~ 1100 live births per group enable detection of a 25% difference in preterm births, pro- portion LBW and SGA between pre- and peri- conception package alone and control group (A + B vs C + D) and enhanced antenatal, postnatal and early childhood care alone and control group (A + C vs B + D) and a mean difference of 0.15 SD in LAZ score at 24 months for the above comparisons. 600 children in each of the four groups (A, B, C, D) at 24 months will enable detection of 0.2 SD difference in mean LAZ score at 24 months. and 0.2 SD difference in birth weight and birth length between pre- and peri-conception intervention package and enhanced antenatal, postnatal and early childhood care and control group. ~2400 (at least 600 children in each of the 4 groups) at 24 months of age will also allow detection of interaction odds ratio (lOR) of ≥1.70 to 1.85 in the proportion of stunted children among short mothers (< 150 cm) and among tall mothers (≥ 150 cm) between the control (routine care) and the intervention group who received the intervention package throughout, with 80% power and 95% confidence level. To compare pre and periconception package with no preconception (antnatal, postpartum and child care) (A vs D) predict 90% power at 95% CI to detect 30% relative reduction in LBW, SGA, stunting at 24m; 0.2 SD diffrence in LAZ at 24m (0.8cm) or birth weight/ length (100g/0.45cm) (except 80% power for preterm births). To compare pre- and peri-conception intervention package alone (A + B vs C + D) or Enhanced antenatal, postnatal and early childhood care alone (A + C vs B + D) predict 90% power at 95% CI to detect 25% relative reduction in LBW, SGA, stunting at 24m; 0.15 SD diffrence in LAZ at 24m (0.65cm) or birth weight/ length (75g/0.35cm). |
| **Sample characteristics** | The baseline characteristics of women at both randomisations were similar except for women’s height, proportion underweight, families possessing a below poverty line card, and place of birth |  |
| **Randomisation** | Enrolled women were randomised to receive preconception interventions or routine care (first randomisation).  Women received ultrasonographic confirmation of pregnancy and were then randomised (second randomisation) to pregnancy and early childhood interventions or to routine care. The randomisation list was prepared by an independent statistician. Women were randomised using permuted blocks and stratified by maternal height (<150 cm and ≥150 cm). Group allocation (1:1) was through a web based system. The two step randomisation resulted in four groups:  - preconception, pregnancy and early childhood interventions (A),  - preconception interventions only (B),  - pregnancy and early childhood interventions only (C),  - no preconception interventions, and routine pregnancy and early childhood care (D). | The randomization list prepared by an independent statistician at World Health Organization (WHO) using random permuted blocks, stratified by maternal height [<150cm (< −2 SD) and ≥150cm (≥−2 SD)] of the WHO standards [58]. 1st randomization done at enrollment when married women aged 18 to 30 years fulfil the eligibility criteria and consent to participate in the study.  2nd randomization done when women become pregnant during the 18months follow up period, and are eligible (not moving away from the study area) at rescreening and consent to their own and their baby’s partici- pation in the study. The group allocation is done through a web-based system. There are no additional criteria for discontinuation or modification of allocated interventions. |
| **Blinding** | authors could not mask participants and teams because of the nature of the interventions. Outcomes were assessed by an independent team not involved in delivering interventions or aware of the group allocation before measurements. | Masking participants and study teams is not possible because of the nature of interventions in this trial. However, attempts are made to keep the independent out- come ascertainment team unaware of the group allocation, to the extent possible. The radiologists performing the scan are blinded to the group allocation of the pregnant women.  An interim analysis will be conducted in a blinded manner when 50% of the babies in the study are born, a second review will be done when 50% of the babies in the study reach 2 years of age. |
| **Age of participants (years)** | aged 18-30 years | 18 to 30 years |
| **Inclusion criteria** | 18-30 year old married women, with no child or one child who wish to have a child (identified through a survey) | Women aged 18–30 years, married and living with their husband, with no or one child and wish to have a child, and consent for participation in the study. |
| **Exclusion criteria** | Women living in temporary housing and those moving away and those not consenting were excluded.  Excluded cases of abortion, stillbirth, deaths of child or mother after birth. | Families who plan to move out of the study area or live in temporary housing (households without concrete roof, toilet, water connection and legal electricity) are excluded as they are likely to be relocated by the govern- ment in the near future. |
| **Preconception intervention characteristics** | Trial community workers or Sanginis (friends) visited homes weekly to reinforce interventions, replenish supplies, and help to manage health conditions. Women with moderate to severe anaemia, thyroid disorders, reproductive tract infections, or undernutrition were contacted more frequently. The interventions were in four domains: health, nutrition, psychosocial care and support, and WaSH delivered during **preconception**: Health: Screen medical conditions reproductive tract infections, tuberculosis, thyroid disorders, hypertension, prediabetes, diabetes, undernutrition, anaemia, and depressive symptoms and treated them according to standard protocols. Contraceptives were provided to women who had started living with their husbands in the past 12 months, had a child aged 12 months or younger, or had severe undernutrition, moderate to severe anaemia, hypothyroidism, reproductive tract infection, diabetes, or hypertension. Gave women an albendazole tablet twice a year.  Nutrition: Provide IFA (weeklyto women with no anaemia) and MMN (3 times a week to women who were given contraceptives);  Provide egg or milk (70 kcal, 6 g protein) daily for six days a week if body mass index <21; Screen and treat malnutrition using locally prepared snacks given to undernourished women (body mass index <16: 1000 kcal/day and 20-22 g protein/day; body mass index 16-18.40: 500 kcal/day and 12-14 g protein/ day)., Treated anaemia following standard protocols. Counselling on adequate diets Psychosocial support: Promote positive thinking and problem solving skills using an adaptation of the thinking health module of the World Health Organization during counselling. WaSH: Promote personal, menstrual and hand hygiene during counselling. | Health: Screen and treat medical conditions Nutrition: Screen and treat malnutrition and anemia; Provide iron-folic acid, multiple micro nutrients, locally-prepared snacks, egg or milk Psychosocial: Promote positive thinking and problem- solving skills WASH: Promote personal, menstrual and hand hygiene |
| **Preconception control group intervention** | Women in the control group were advised to seek care from government sources (free of cost) to access family planning services and weekly iron folic acid supplementation. | Only weekly IFA supplementation as part of National Program (National Iron Plus Initiative) |
| **Intervention timing (how far before conception)** | Up to 18 months in the control arm.  Median time between enrolment and pregnancy confirmation 126 days, interquartile range 33-275 days) in rpreconcpetion arm; cf. no preconception intervention group (162 days, 50-311 days). Median gestational age when pregnancy interventions were started was 10.6 weeks (interquartile range 9.9– 12.3 weeks) so that might have influenced timing of 2nd randomisation, so preconcepton arm women also got early pregnancy intervention. |  |
| **Intervention delivery (health systems, food systems, social protection systems)** | Intervention delivered by project staff who visited women in their homes to deliver supplements and give counselling. Health checkups delvered through the health system. | Intervention delivered by project staff who visited women in their homes to deliver supplements and give counselling. Health checkups delvered through the health system. |
| **Pregnancy detection** | Women randomised to the preconception intervention and the no preconception intervention groups were contacted monthly to enquire about missed periods. Women had a transabdominal ultrasound to confirm pregnancy when two consecutive missed periods were reported. Median gestational age when pregnancy interventions were started was 10.6 weeks (interquartile range 9.9– 12.3 weeks) | The enrolled woman is requested to inform the study team by calling designated phone numbers if she gets pregnant. SET workers make calls to all women every month (or home visits if the call is unsuccessful) to enquire about missed periods. If women report two missed periods or inform that they are pregnant (self-testing using a pregnancy kit), a trans-abdominal ultrasound is scheduled. |
| **Pregnancy intervention characteristics (if any)** | The median gestational age when pregnancy interventions were started was 10.6 weeks (interquartile range 9.9– 12.3 weeks). Trial community workers or Sanginis (friends) visited homes weekly to reinforce interventions, replenish supplies, and help to manage health conditions. Women with moderate to severe anaemia, thyroid disorders, reproductive tract infections, or undernutrition were contacted more frequently. The interventions were in four domains: health, nutrition, psychosocial care and support, and WaSH delivered during **pregnancy** Health: At least eight antenatal contacts. Screened for anaemia, gestational diabetes mellitus, thyroid disorders, gestational hypertension, gestational weight gain, asymptomatic bacteriuria, reproductive tract infections, and depressive symptoms during monthly antenatal care visits.  Hospital registration for childbirth was encouraged.   Screened and treated women with inadequate weight gain for infections (urinary tract infection, reproductive tract infection, dental infection, tuberculosis). Nutrition: Provide micronutrient supplements, iron folic acid, calcium and vitamin D daily, and albendazole once during pregnancy.  Weekly supplies of locally prepared snacks (210 kcal, 2 g protein in the second trimester, and 400 kcal, 21 g protein in the third trimester) were provided for daily consumption in women with body mass index <25. Milk was provided six days a week to all women and its consumption was observed. Weight of pregnant women was monitored every month and inadequate weight gain was identified (IoM guidelines). Women with inadequate weight gain were provided nutritional counselling and a hot cooked meal six days a week (500 kcal, 20 g protein) until delivery. Psychosocial support:Promote positive thinking and problem solving skills during counselling WaSH: Provide water filters, water storage bottles, soap, hand washing station, disinfectant provided to families. | Health: > 8 antenatal contacts, screen and treat medical conditions, calcium and vitamin D supplementation Nutrition: Provide iron-folic acid, multiple micro nutrients, locally-prepared snacks and milk, monitor weight; Provide iron-folic acid, multiple micro nutrients, locally-prepared snacks, egg or milk Psychosocial: Promote positive thinking and problem- solving skills WASH: Provide water filters, soap, hand washing station, disinfectant |
| **Pregnancy control group intervention (if any)** | Women in the control group were advised to register for antenatal care at a government or private facility, have at least four antenatal care check- ups, consume iron folic acid, calcium, vitamin D daily throughout pregnancy, access supplementary foods through the Integrated Child Development Services (ICDS) scheme and plan to deliver in health facilities. | Routine antenatal care |
| **Interventions after birth (if any)** | The interventions were in four domains: health, nutrition, psychosocial care and support, and WaSH delivered during early childhood periods. Trial community workers, Prernas (inspiration), enabled postnatal visits to facilities. **Early childhood:** Health: Empower family to identify danger signs and seek care early. Newborns were home visited within 24 hours of birth or hospital discharge, five times in the first month, monthly until 12 months, and three monthly thereafter up to 24 months of age. Additional visits were made for babies born preterm, LBW, and for mothers with breastfeeding problems. Nutrition: 0-6 months: lactation support for early and exclusive breastfeeding: Exclusive breastfeeding was promoted until six months. Mothers were trained to feed expressed breastmilk to babies born preterm. Lactation counsellors supported mothers with breastfeeding problems. Vitamin D (400 IU) was provided daily to all infants. Iron supplementation was given from two weeks for the very LBW and from six weeks to LBW infants until six months of age. 6-24 months: promote timely CF and continued breastfeeding. Complementary food supplements (milk-cereal mix) provided ; provide supplementary food (125 and 250 kcal, 2.5 and 5 g protein in months 6-11 and 12-23, respectively) *or was it (250 kcal and 5 g protein from 12 to 24 m?)*. Double supplement if inadequate weight gain. Weights were measured during home visits and children with inadequate weight gain (<15th centile from birth to 6 mo and <25th centile weight velocity/mo from 6-24 mo) were referred to lactation counsellors and paediatricians. Mothers were offered additional packets of food supplements until infants no longer showed inadequate weight gain. The increased amount was given as additional packets of milk-cereal mix or other locally available foods based on the mother’s preference.  Supplements provided 40-60% of daily energy & 80-100% daily micronutrient requirements. nutrient dense recipes made with locally available foods were provided to families and responsive feeding was promoted. An iron supplement was also given. Psychosocial support: Promote early child play and responsive care: Mothers were taught age specific child play, responsive care, and stimulation activities. Development milestones were assessed three monthly by Prernas. WaSH:Provide play mat and potty **Postnatal 6 months: mother:** Health: Arrange postnatal visit at six weeks Nutrition: Snacks, milk (600 kcal, 20 g protein), micronutrient supplements, iron folic acid, calcium, and vitamin D were given for six months to meet additional requirements during lactation. Psychosocial support: Pounselling on positive thinking and problem solving skills, screening and management of depressive symptoms WaSH: interventions were continued. Hand washing, use of diapers, appropriate disposal of faeces, and a clean play area for the child were promoted | Health: Empower family to identify danger signs and seek care early Facilitate postnatal visit at 6 wks for mother Nutrition: 0–6 mo: lactation support for early and exclusive breastfeeding 6–24 mo: promote timely complementary feeding and continued breastfeeding, provide quality food, monitor inadequate weight gain For mother: Iron-folic acid, multiple micronutrients, calcium and Vitamin D, locally- prepared snacks and milk supplementation Psychosocial: Promote early child play and responsive care Mother: Promote positive thinking and problem- solving skills WASH:  Provide play mat and potty Provide water filters, soap, hand washing station, disinfectant |
| **Control group intervention after birth (if any)** | Women in the control group were advised to go for a postnatal health check-up, and to consume iron folic acid, calcium, vitamin D, and supplementary foods daily through the ICDS scheme. Mothers were advised to breastfeed their babies exclusively for the first six months, and continue breastfeeding for at least two years. They were also encouraged to arrange home visits by the community health workers in the first 42 days of life, and to collect supplementary food from ICDS and iron folic acid from 6 to 24 months | Routine Postnatal care |
| **Compliance measurement** | Compliance to interventions was assessed by study workers through observation or by asking mothers during home visits  Compliance with interventions was high. Around 90% of women were screened and received treatment for different morbidities during the preconception and pregnancy periods. Women and children consumed nutritional supplements for around 75% of the follow- up period. Around 97% of women received counselling on positive thinking and problem solving skills, and 98% of children on early child play and responsive care. | The “Program Learning Team” comprises of two social scientists who conduct interviews and observations around the key study activities. They assess compliance to interventions through interviews and observations. Additionally, in-depth interviews are done with non-responders to anemia and those with inadequate weight gain to ascertain possible reasons. The findings are communicated to the relevant study teams and are used to strengthen intervention delivery through retraining of workers and improvement in processes, whatever is applicable. During pregnancy, weight gain, compliance to interventions and micronutrient status is assessed byt outcome measurement team. |
| **Adverse events** | The serious adverse events for this study were severe allergic reactions to supplements and death; these were reported to the data safety monitoring committee (DSMC) and the ethics committee. No adverse events related to the intervention were reported. | This is a low-risk trial and serious adverse events are not anticipated. However, all deaths in enrolled participants are being reported to the local ethics committee and the World Health Organization for further communication to the DSMC. Additionally, adverse events reported with any supplement are being documented and all severe adverse events will be reported to the ethics committees and the DSMC. |
| **Engagement, category (including nutrition sensitive/specific)** | Nutrition specific & nutrition sensitive | Nutrition specific & nutrition sensitive |
| **Where in the overall UNICEF 2020 framework the intervention lies** | Health: Screen & treat medical conditions is affecting underlying determinants of services the immediate determinant of care and the outcome "health". Nutrition: Providing IFA, MMN, egg or milk (70 kcal, 6 g protein) and treating anaemia is affecting underlying determinants of "food" the immediate determinant of "diet". Meanwhile counselling on adequate diets affects the underlying determinant "practices". Psychosocial support: positive thinking and problem solving skills address the enabling determinants "norms" and "social/human capital" WaSH: personal, menstrual and hand hygiene affects the underlying determinant "services" which drives the immediate determinant "care, driven by adequate services and practices". | Health: Screen & treat medical conditions is affecting underlying determinants of services the immediate determinant of care and the outcome "health". Nutrition: Providing IFA, MMN, egg or milk (70 kcal, 6 g protein) and treating anaemia is affecting underlying determinants of "food" the immediate determinant of "diet". Meanwhile counselling on adequate diets affects the underlying determinant "practices". Psychosocial support: positive thinking and problem solving skills address the enabling determinants "norms" and "social/human capital" WaSH: personal, menstrual and hand hygiene affects the underlying determinant "services" which drives the immediate determinant "care, driven by adequate services and practices". |
| **Where in the Partap 2021 framework the intervention lies** | This complex intervention works mostly at individual level but spans all 4 domains of health, nutrition, social and WASH related factors that affect preconception. | This complex intervention works mostly at individual level but spans all 4 domains of health, nutrition, social and WASH related factors that affect preconception. |
| **Proposed mechanism of intervention action** |  |  |
| **Exposures relating to preconception nutrition** | Trial workers delivered preconception services: Health: Screen and treat medical conditions undernutrition, anaemia, and depressive symptoms Deworming x2/year.  Nutrition: IFA weekly or MMN (x3/week) depending on health status.  Provide egg or milk (70 kcal, 6 g protein) daily for six days a week if body mass index <21; Screen malnutrition and give additional locally prepared snacks to to undernourished women (BMI <16: 1000 kcal/day and 20-22 g protein/day;  BMI 16-18.40: 500 kcal/day and 12-14 g protein/ day).  Treated anaemias. Counselled on adequate diets Psychosocial support: Promote positive thinking and problem solving skills. WaSH: Promote personal, menstrual and hand hygiene. |  |
| **Preconception measures** | The key secondary outcomes for women were infection, hypothyroid status, weight, anaemia status, and depressive symptoms at the end of preconception |  |
| **Concommittant care** | Not mentioned |  |
| **Follow-up schedule for measurements** | Women were followed up until pregnant or up to 18 months after enrolment. Women received ultrasonographic confirmation of pregnancy and were then randomised (second randomisation) to pregnancy and early childhood interventions or to routine care. Outcomes were measured at birth and 24 months of age. Outcomes were assessed by an independent team at the end of the preconception period, after 26-28 and 35-37 weeks of gestation, within the first week of birth, at one month, and three monthly thereafter until infant age 24 months. The infant’s weight, length, mid- upper arm circumference, and head circumference were measured by a pair of workers independently and repeated if the difference was outside the prespecified limit. The two readings were averaged and used for analysis. Ten per cent of measurements were repeated independently. | Screening and enrolment team (SET) identify eligible women through a door-to-door survey in the study areas of Dakshinpuri, Govindpuri, Madangir and Tigri, Khanpur, Sangam Vihar, Jaitpur and Meethapur and Madanpur Khadar areas of South Delhi. Information about the study is shared with families and written informed consent is taken.  Height (Seca-213 stadiometer) and weight (Salter 9509 weighing scale) measurements are taken and the participant is allocated to the intervention (pre- and peri-conception) or control (routine care) group]. The intervention delivery team is informed if the woman is randomized to the intervention group. Post-enrolment, information is documented on sociodemographic characteristics. Participants are visited by the independent outcome ascertainment team at the end of pre- and peri-conception period. The symptoms of RTI, depressive symptoms, compliance to interventions, micronutrient, anemia, thyroid and diabetes status are ascertained. During pregnancy, weight gain, compliance to interventions and micronutrient status is assessed. Weight, length, head- and mid-upper arm circumference, child care practices, prevalence of illness and care seeking and hospitalization are assessed during early childhood (Additional file 3).  Weights and lengths are taken by pair of workers using digital weighing scale (model 354; Seca, California, USA) and infantometer (model 417; Seca, California, USA) to the nearest 10 g and 0.1 cm, respectively. Head and mid-upper arm circumference is taken using a measuring tape (model 212; Seca, California, USA) [66,67,68]. |
| **Summary of baseline comparisons by study arm** | The baseline characteristics of women at both randomisations were similar except for women’s height, proportion underweight, families possessing a below poverty line card, and place of birth. In preconception arm versus control height <150cm in , 34.9% vs. 33.8%  underweight <18.5kg/m2 13.2 vs 16.6 <12 y scholing 48.9 vs 50.7 Biggest difference was in family below poverty line 2.3% vs 6.3%. Most differences between combined preconception and no preconception arms only small. | Comparability between the two groups Summary values (means, proportions) for sociodemo- graphic characteristics among the groups will be pre- sented in the baseline table. We will not perform any test of significance. Our large sample size is likely to yield a balance between the groups. How- ever, we will carefully examine the size of any base- line differences. Imbalanced characteristics that may influence the primary outcomes will be adjusted appropriately. |
| **Pregnancy outcomes: gestational weight gain, gestational diabetes, maternal anthropometry (weight, MUAC, height), anaemia,** | Outcomes were assessed by an independent team at the end of the preconception period, after 26-28 and 35-37 weeks of gestation. The key secondary outcomes for women were infection, hypothyroid status, weight, anaemia status, and depressive symptoms at the end of preconception and during pregnancy. | Key 2ndary outcomes for women are  micronutrients and anemia status,  depressive symptoms and  infection at the end of pre- and peri-conception Symptoms of RTI, depressive symptoms, compliance to interventions, micronutrient, anemia, thyroid and diabetes status are ascertained. During pregnancy, weight gain, compliance to interventions and micronutrient status is assessed: At end of pre-/peri conception only: Birth interval BMI (weight; height measured at enrollment) Thyroid status Diabetes status At end of pre-/peri conception and 35-36 weeks: Symptoms of RTI/STI Depressive symptoms Inflammatory markers (C-reactive protein, Alpha-acid glycoprotein) Micronutrient status (vitamin A, D, B12, zinc, iron, folate and selenium) Anemia status At 28 weeks gestation: Weight Gain |
| **Preconceptual outcomes: (BMI, Hb or other outcomes which could be attributed to preconception care)** |  |  |
| **Birth outcomes in child: birthweight, low birth weight (LBW), small for gestational age (SGA), preterm birth/ delivery (PTB), cord blood b12, DXA,** | The primary outcomes at birth were  proportion LBW (birth weight <2500 g),  preterm birth (ultrasound confirmed gestational age at birth <37 completed weeks),  SGA (birth weight centile <10th using INTERGROWTH-21st standards), and  mean birth weight and length. Outcomes were assessed by an independent team within the first week of birth, at one month, and three monthly thereafter until infant age 24 months. | The primary outcomes include proportion  preterm birth and preterm pre-labor rupture of membranes or spontaneous onset of labor (ultrasound-confirmed gestational age at birth < 37 completed weeks s of gestation0;  proportion SGA (birth weight centile <10th as per INTERGROWTH-21 standard) on day 7 of birth; (*Note they are not using weight on day 0 as could be unequally measured by arm*)  proportion LBW (birth weight < 2500 g) on day 7;  mean birth weight and length;   The list of secondary outcomes : - weight and length trajectories from from birth to 24 months at month 1,3,5,6,9,12,15,18,21,24,  - body composition (in a subsample) at 1 month of age and  - morbidity and hospitalization from birth to 24months. Early initiation of breastfeeding - birth or day 7 Exclusive breastfeeding - 1& 5 m  Mother's Postpartum morbidity at birth or day 7 Pregnancy outcomes, Still birth |
| **Outcomes in mother at birth** | The key secondary outcomes for women were infection, hypothyroid status, weight, anaemia status, and depressive symptoms at the end of preconception, during pregnancy, and postpartum. | Secondary outcomes for women during postnatal period are micronutrients and anemia status, depressive symptoms and infection.: Postpartum morbidity at birth or day 7 Pregnancy outcomes, Still birth |
| **Post-natal outcomes (in Infancy)** | Outcomes were assessed by an independent team within the first week of birth, at one month, and three monthly thereafter until infant age 24 months. LAZ, WHZ, WAZ Proportions stunted, wasted and underweight weight and length trajectories from birth to 24 months, and  hospital admission from birth to 24 months | show in adjacent coumn for over 12 m |
| **Outcomes in childhood after 12 m: z scores for weight (WAZ), length/height (HAZ), and BMI for age (BMIZ), weight-for-height'length (WHZ), underweight, stunting, wasting, overweight, cognitive** | At 24 months of age, primary outcomes were mean length-for-age z scores and proportion stunted. The key secondary outcomes for children were proportion stunted (length- for-age z score <–2 standard deviations of the WHO child growth standards), wasted (weight-for-height z score <−2 standard deviations of the WHO child growth standards), underweight (weight-for-age z score <−2 standard deviations of the WHO child growth standards) during 6-24 months,  weight and length trajectories from birth to 24 months,  anaemia status at 24 months, morbidity, and  hospital admission from birth to 24 months  DSMB recommended stopping the study based on strong evidence for the beneficial effects for 24 month outcome so sample size was not reached. | One of the primary outcomes: attained length (LAZ) at 24 months of age and proportion stunted (LAZ < -2 SD) Key child 2ndary outcomes: - proportion stunted at 6 & 12m,  - wasted and underweight at 6, 12 & 24m, all calculated based on length measured at 24months (±28days) by WHO standards. - weight and length trajectories from birth to 24 months at month 1,3,5,6,9,12,15,18,21,24,  - body composition (in a subsample) at 1 month of age and  - neurodevelopment at 6, 12, 18 and 24months (in a subsample): Caregiver reported development outcomes & Cognitive, language and motor scores, Mother-infant bonding (6,12,18 m) micronutrients (vitamin A, D, B12, zinc, iron, folate and selenium), Inflammatory markers and anemia status at 24 months,  morbidity and hospitalization from birth to 24months. Early initiation of breastfeeding - brith or day 7 Exclusive breastfeeding - 1& 5 m Continued breastfeeding - 12, 18 24 m Complementary feeding - 12, 18 24 m In **mother** during first year: BMI (weight; height measured at enrollment) - 6 and 12 months Depressive symptoms - 2 and 12 months Inflammatory markers (C-reactive protein, Alpha-acid glycoprotein) - 6 months Micronutrient status (vitamin A, D, B12, zinc, iron, folate and selenium) - 6 months Anemia status - 6 months |
| **Long term outcomes** | None |  |
| **Analysis** | A priori analysis approach for this factorial design, randomised controlled trial to evaluate the three study hypotheses by making three comparisons for all primary and secondary outcomes:  effect of preconception interventions (groups A+B v C+D);  effect of pregnancy and early childhood interventions (groups A+C v B+D); and the  combined effect of interventions from preconception until two years after birth (group A v D).  authors also displayed comparisons of individual groups and assessed interaction between preconception interventions and pregnancy and early childhood interventions for all primary outcomes.  authors used 98.3% confidence intervals of effect sizes for all primary and secondary outcomes to adjust for the three comparisons (significance level 0.05/3 or 0.017). The study had multiple primary outcomes related to LBW (proportion LBW, SGA, and preterm, and mean birth weight and length) and stunting (proportion stunted and mean length-for-age z score at 24 months of age). Our a priori decision was not to adjust for multiple comparisons because the primary outcomes were likely to be correlated, authors were not addressing a universal null hypothesis, and formal adjustments for multiplicity are unlikely to enhance interpretation. But onducted a post hoc sensitivity analysis adjusting P values for multiple primary outcomes using the Holm-Bonferroni method, adjusting for 21 comparisons; seven primary outcomes (five at birth and two at 24 months) and three,two group comparisons. means or proportions of baseline characteristics across the groups to check whether randomisation was successful. Intention-to-treat analysis was conducted. authors used generalised linear models of the Poisson family with a log link function and Gaussian family with an identity link function to calculate incidence rate ratio and mean difference for binary and continuous outcomes, respectively. authors also calculated absolute risk reduction with 98.3% confidence interval for primary and secondary outcomes at birth and 24 months. The final models were adjusted for place of birth, family possessing a below poverty line card, women’s height, and women’s body mass index which were potential confounders. authors also adjusted the analysis for clustering due to twins. The intervention effect on secondary outcomes was assessed using the same models as for primary outcomes. authors used Kernel weighted local polynomial smoothing technique to create length-for-age z score from birth to 24 months for all three comparisons. The relative measures of effect on key primary outcomes within each of the prespecified subgroup analyses were estimated and presented as forest plots. Data analysis was conducted with Stata version 16.0 | Primary comparisons The primary analysis will be factorial, for comparison of of effects on birth outcomes (mean birth weight and length, proportion stunted at birth, proportion of babies born preterm and spontaneous preterm births, SGA and LBW) and mean length for age z-score and proportion stunted at 24 months. Group A + C vs Group B + D: enhanced antenatal, postnatal and early childhood care (with pre- /peri- conception intervention package or enhanced antenatal, postnatal and early childhood care alone i.e. Group A + C) vs no enhanced antenatal, postnatal and early childhood care (pre-/ peri-conception intervention package alone or routine care i.e. Group B + D)  Group A + B vs Group C + D: - pre- /peri-conception intervention package (alone or with enhanced antenatal, postnatal and early childhood care package i.e. Group A + B) vs no pre- / peri- conception intervention package (enhanced antenatal, postnatal and early childhood care package alone or routine care i.e. Group C + D) Group A vs Group D: In addition to factorial analysis, examine the impact of the combined pre- / peri- conception intervention package and enhanced antenatal, postnatal and early childhood care (Group A) on birth outcomes and attained length at 24 months of age, compared to routine care (Group D). Examine interaction between the intervention package delivered during peri-preconception period and that delivered during antenatal and early childhood period on primary outcomes. Main effects Analysis by intention-to-treat. Mean (SD) birth weight, birth length, LAZ score at 24 months, proportion of preterm birth and spontaneous preterm birth, SGA, LBW, stunting at birth and 24 months presented for the different groups. For binary outcomes, generalized linear models (GLMs) of the binomial family with a log-link function will be used to calculate the effect size [relative risk and 95% confidence interval (CI)]. For continuous outcomes, GLMs of the Gaussian family with an identity-link function will be used to calculate the effect size (difference in means and 95% CIs). The effect of interventions on secondary outcomes will be assessed using the same models as for primary outcomes. Weight and length growth trajectories between birth and 24 months A multivariable linear mixed-effect regression model with an unstructured covariance matrix will be used to examine the effect of the interventions on weight and length trajectories from birth to 24 months.  Account for the interdependence of multiple observation periods in the same child, time with age in months at assessment as level-1 source of variation, with individual children at level 2.  All potential covariates will be included as fixed effect variables in this model.  Interaction between maternal stature and time (age in months) of assessment on different anthropometric outcomes i.e. LAZ, WAZ (weight-for-age z-score) and WLZ scores wexamined. If significant, the interaction term will be included in the model to obtain the independent effect of maternal stature at different ages. Pre-specified subgroup analysis - conduct subgroup analysis for women according to their height (< 150 cm and ≥ 150 cm), underweight, BMI status at enrolment and at the time of pregnancy confirmation, years of education and high risk pregnancy.  - also conduct subgroup analysis by wealth quintile of the household. The relative measures of effect within each of these subgroups will be estimated. The effect of interventions on secondary outcomes will be assessed using the same models as for primary outcomes. |
| **EFFECTS on Preconception outcomes: maternal anthropometry (weight, MUAC, height. BMI), Haemoglobin (Hb), anaemia, iodine status** |  |  |
| **EFFECTS on Pregnancy outcomes: gestational weigt gain, gestational diabetes, maternal anthropometry (weight, MUAC, height), anaemia, iodine status** | **Effects of any preconcpetion versus no preconception (control and post delvery) (A+B v C+D)**. After confirmation of pregnancy, the proportion of women with reproductive tract infections (RTI) was lower (incidence rate ratio 0.68, 98.3% confidence interval 0.59 to 0.80; absolute risk reduction −7.49%, 98.3% confidence interval −10.57% to −4.41%), and mean haemoglobin was 0.56 g/dL higher (98.3% confidence interval 0.48 to 0.64 g/dL) in the preconception intervention groups than in the groups that did not receive these interventions. The proportion of women with hypothyroidism (0.83, 0.68 to 1.03; −1.96%, −4.34% to 0.42%) and body mass index <18.5 (0.88, 0.73 to 1.05; −1.98%, −4.64% to 0.68%) was lower in groups that received preconception interventions than in groups that did not receive preconception interventions, but the upper limit of the confidence interval crossed null effect. **Effects of pregnancy intervention versus no pregnancy (preconception only & control) (A+C v B+D):** At 35-37 weeks of gestation, haemoglobin concentration (mean difference 0.68 g/dL, 98.3% confidence interval 0.56 to 0.80 g/dL) was higher, and the proportion with moderate anaemia (incidence rate ratio 0.36, 98.3% confidence interval 0.28 to 0.47; absolute risk reduction −13.45%, 98.3% confidence interval −16.42% to −10.48%), severe anaemia (0.03, 0 to 0.31; −1.69%, −2.46% to −0.92%), reproductive tract infection (0.66, 0.56 to 0.79; −8.33%, −11.88% to −4.78%), preeclampsia or eclampsia (0.55, 0.33 to 0.91; −1.68%, −3.12% to −0.24%) was lower (A+C v B+D). Gestational weight gain between enrolment and 35 weeks of gestation was 1.42 kg (98.3% confidence interval 1.15 to 1.70) higher in the pregnancy intervention groups than in the no pregnancy intervention groups (A+C v B+D). Effects of **preconcpetion only versus pregnancy and preconception** at 35-37 weeks of pregnancy (A v B): No effects on RTI −1.44 (−4.97 to 2.10), hypertension ARR 0.18 (−2.00 to 2.36). No effect on Hb ARR: 0.08 (−0.04 to 0.19) or on any, mild or moderate anaemia but signfiicanly lower risk of severe anaemia −0.77 (−1.50 to −0.04). No significant effect on gestational weight gain to 26-28wks, though ARR was 0.16 (−0.05 to 0.36).Lower risk of postpartum haemorrhage −1.44 (−2.49 to −0.38) but no signficant difference in pre-eclampsia−0.67 (−2.05 to 0.72) or antepartum harmorrhage 0.03 (−0.39 to 0.45). | None as this is protocol |
| **EFFECTS on Birth outcomes in child: birthweight, low birth weight (LBW), small for gestational age (SGA), preterm birth/ delivery (PTB)** | The proportion with low birth weight was lower in the preconception intervention groups (506/2235) than in the no preconception intervention groups (502/1889; incidence rate ratio 0.85, 98.3% confidence interval 0.75 to 0.97; absolute risk reduction −3.80%, 98.3% confidence interval −6.99% to −0.60%). The proportion with low birth weight was lower in the pregnancy intervention groups (502/2096) than in the no pregnancy intervention groups (506/2028) but the upper limit of the confidence interval crossed null effect (0.87, 0.76 to 1.01; −1.71%, −4.96% to 1.54%). There was a larger effect on proportion with low birth weight in the group that received interventions in the preconception and pregnancy periods (267/1141) compared with the control group (267/934; 0.76, 0.62 to 0.91; −5.59%, −10.32% to −0.85%). Effects of any preconcpetion versus no preconception (control and post delvery) (A+B v C+D).   proportion LBW was lower (incidence rate ratio 0.85, 98.3% confidence interval 0.75 to 0.97; absolute risk reduction −3.80%, 98.3% confidence interval −6.99% to −0.60%) and proportion stunted at birth was lower (incidence rate ratio 0.81, 98.3% confidence interval 0.69 to 0.96; absolute risk reduction −3.18%, 98.3% confidence interval −5.91% to −0.45%) proportion SGA was also lower (0.87, 0.78 to 0.98; −4.04%, −7.47% to −0.62%) .  Birth weight was higher (mean difference 40.84 g, 98.3% confidence interval 7.84 to 73.84 g) and birth length was also higher (0.17 cm, 0.01 to 0.32 cm) .   proportion of preterm births did not differ between the groups (incidence rate ratio 1.05, 98.3% confidence interval 0.87 to 1.27; absolute risk reduction 0.59%, 98.3% confidence interval −1.79% to 2.98%). Head circumference (mean difference 0.07, 98.3% confidence interval −0.03 to 0.17 cm) and still births (incidence rate ratio 0.97, 98.3% confidence interval 0.56 to 1.67; absolute risk reduction 0.01%, 98.3% confidence interval −0.89% to 0.87%) did not differ between the groups Effects of pregnancy intervention versus no pregnancy (preconception only & control) (A+C v B+D):  proportion SGA was lower incidence rate ratio 0.80, 98.3% confidence interval 0.71 to 0.90; absolute risk reduction −6.60%, 98.3% confidence interval −10.15% to −3.05%); proportion LBW (0.87, 0.76 to 1.01; −1.71, −4.96% to 1.54%) and  proportion preterm birth (0.85, 0.69 to 1.05; −1.47%, −3.86% to 0.93%) and mean birth weight was also lower (mean difference 35.97 g, 98.3% confidence interval −0.17 to 72.12 g), but the upper limit of the confidence interval crossed null effect.  proportion stunted at birth was lower (incidence rate ratio 0.84, 98.3% confidence interval 0.70 to 1.00; absolute risk reduction −2.10%, 98.3% confidence interval −4.88% to 0.67%) and head circumference was 0.12 cm higher (98.3% confidence interval 0.01 to 0.23 cm) Still births (incidence rate ratio 1.17, 98.3% confidence interval 0.62 to 2.18; absolute risk reduction −0.26%, 98.3% confidence interval −1.16% to 0.64%) did not differ between the groups Effects of preconception and pregnancy periods (A) vs control group (D):  proportion LBW was lower (incidence rate ratio 0.76, 98.3% confidence interval 0.62 to 0.91; absolute risk reduction −5.59%, 98.3% confidence interval −10.32% to −0.85%) and the proportion SGA was also lower (0.71, 0.61 to 0.83; −11.84%, −16.94% to −6.75%)  Birth weight was 77.67 g higher (98.3% confidence interval 26.38 to 128.96 g);  birth length (mean difference 0.21 cm, 98.3% confidence interval −0.03 to 0.45 cm) and proportion of preterm births (incidence rate ratio 0.91, 98.3% CI 0.69 to 1.19; absolute risk reduction −0.86%, 98.3% confidence interval −4.36% to 2.64%) did not differ in groups A and D. proportion stunted at birth was lower (incidence rate ratio 0.68, 98.3% confidence interval 0.53 to 0.87; absolute risk reduction −5.32%, 98.3% confidence interval −9.42 to −1.22) and head circumference was 0.18 cm higher (98.3% confidence interval 0.02 to 0.33 cm) . Still births (1.37, 0.59 to 3.19; −0.26%, −1.55% to 1.04%) did not differ between the groups. Subgroup analyses for primary outcomes for all prespecified comparisons did not show any significant effect modification (figs S1-S7, supplementary appendix). The intervention impact at birth and at 24 months was similar for short (<150 cm) and tall (≥150 cm) women. Table 7 shows interaction effects between pregnancy and preconception itnerventions and displays the effect sizes in each arm compared with control: extracting just the preconception only versus control: LBW: control 28.6, preconception only 21.9 IRR 0.78 (0.67 to 0.91); P=0.001. Measure of interaction (95% ci) 1.20 (0.97 to 1.48); P=0.10 Preterm birth: control 14.8, preconception only 21.9, IRR 0.88 (0.71 to 1.09); P=0.24. Measure of interaction (95% ci) 1.52 (1.11 to 2.08); P=0.01 Small for gestational age: control 37.7, preconception only 29.9, IRR 0.81 (0.72 to 0.91); P=0.001. Measure of interaction (95% ci) 1.17 (0.98 to 1.40); P=0.09 Birth weight: control 2744 (459)g, preconception only 2816 (449)g, MD 65 (26 to 103); P=0.001. Measure of interaction (95% ci) −47 (−101 to −7); P=0.09 Birth length: control 47.9 (2.2)cm, preconception only 48.19 (2.07)cm, MD 0.29 (0.11 to 0.47); P=0.002. Measure of interaction (95% ci) −0.23 (−0.48 to 0.02); P=0.08 | None as this is protocol |
| **EFFECTS on Outcomes in mother at birth** | None | None as this is protocol |
| **EFFECTS on Post-natal outcomes (in Infancy)** | Growth trajectories shown in the >12 month column One noteworthy finding was that the proportion of infants exclusively breastfed at 5 months of age was higher (incidence rate ratio 2.78, 98.3% confidence interval 2.54 to 3.05; absolute risk reduction 47.68%, 98.3% confidence interval 44.20% to 51.15%) in the pregnancy and early childhood intervention groups (A+C v B+D), and in the group that received interventions in both periods (group A v D; 2.57, 2.25 to 2.92; 45.15%, 40.11% to 50.19%) | None as this is protocol |
| **EFFECTS on Outcomes in childhood after 12 m: z scores for weight (WAZ), length/height (HAZ), and BMI for age (BMIZ), weight-for-height'length (WHZ), underweight, stunting, wasting, overweight, cognitive** | The proportion stunted at 24 months of age was substantially lower in the pregnancy and early childhood intervention groups (79/746) compared with the groups that did not receive these interventions (136/710; 0.51, 0.38 to 0.70; −8.32%, −12.31% to −4.32%), and in the group that received preconception, pregnancy, and early childhood interventions (47/453) compared with the control group (51/271; 0.49, 0.32 to 0.75; −7.98%, −14.24% to −1.71%). No effect on stunting at 24 months was observed in the preconception intervention groups (132/892) compared with the no preconception intervention groups (83/564). **Effects of any preconcpetion versus no preconception (control and post delvery) (A+B v C+D).**  Mean length-for-age z scores (LAZ) between birth and 24 months did not differ in the groups that received or did not receive preconception interventions (A+B v C+D). At 24 months of age, weight-for-length z score (WLZ) (mean difference 0.05, 98.3% confidence interval −0.09 to 0.18) and weight-for-age z score (WAZ) (0.08, −0.06 to 0.22), wasting (incidence rate ratio 1.04, 98.3% confidence interval 0.75 to 1.43; absolute risk reduction 1.01%, 98.3% confidence interval −3.15% to 5.16%), underweight (0.97, 0.76 to 1.25; 0.78%, −3.80% to 5.36%), and head circumference (mean difference 0.14, 98.3% confidence interval −0.05 to 0.32) *did not differ in the preconception intervention groups* **Effects of pregnancy intervention versus no pregnancy (preconception only & control) (A+C v B+D):** in the pregnancy and early childhood intervention groups- Mean LAZ between birth and 24 months of age were higher from 9 mo At 24 m mean WLZ was 0.33 standard deviation higher (98.3% confidence interval 0.20 to 0.47), the mean WAZ was 0.45 standard deviation higher (0.31 to 0.59), and the head circumference was 0.34 cm higher (0.15 to 0.53) Proportion wasted was lower (incidence rate ratio 0.68, 98.3% confidence interval 0.49 to 0.96; absolute risk reduction −5.12%, 98.3% confidence interval −9.18% to −1.06%) and the proportion underweight was lower (0.57, 0.44 to 0.75; −9.17%, −13.66% to −4.68%) **Effects of preconception and pregnancy periods (A) vs control group (D):**  in the group (A) compared with control group (D) -  Mean LAZ between birth and 24 months of age were higher from 6 months of age.  At 24 months: Mean WLZ was 0.40 standard deviation higher (98.3% confidence interval 0.20 to 0.60), the mean WAZ was 0.54 standard deviation higher (0.33 to 0.75), and the head circumference was 0.46 cm higher (0.19 to 0.74). Proportion wasted was lower (incidence rate ratio 0.71, 98.3% confidence interval 0.44 to 1.13; absolute risk reduction −3.71%, 98.3% confidence interval −9.57% to 2.15%) and proportion underweight was lower (0.56, 0.39 to 0.80; −8.76%, −15.67% to −1.85%). Assessed the interaction between preconception interventions and pregnancy and early childhood interventions for all primary outcomes. Did not find any evidence of interactions except for the effect on preterm birth.  Preconception interventions had important effects on birth size but not on linear growth at 24 months compared with the control group. Pregnancy and early childhood interventions had important effects on birth size and on linear growth at 24 months compared with the control group. The effect sizes of preconception and pregnancy and early childhood interventions together were larger for birth size and linear growth at 24 months than those of the control group compared with the effect sizes of interventions provided only in a single period. | None as this is protocol |
| **Potential Biological mediating factors (as identified by authors of the primary studies)** | mechanisms in the WHO framework on childhood stunting explain our findings related to LBW and stunting. Stewart CP, Iannotti L, Dewey KG, et al. Contextualising complementary feeding in a broader framework for stunting prevention. Matern Child Nutr 2013;9(Suppl 2):27-45. doI:10.1111/ mcn.12088. Preconception intervention group has a higher proportion of live births- improved anaemia and nutritional status and reduced the risk of reproductive tract infections might have improved fertility among women in this group. Counselling on positive thinking and problem solving skills might have led to a state of improved mental wellbeing, conducive to plan for a pregnancy. Pregnancy interventions increased gestational weight gain, reduced the risk of anaemia, micronutrient deficiency, reproductive tract infection, and pregnancy induced hypertension; these are major contributors to LBW. Preconception interventions were at least as important as pregnancy interventions for birth outcomes, but the effect was diluted two years after birth when preconception interventions only had a marginal additional benefit to pregnancy and early childhood interventions on the outcomes at 24 months. Intensive breastfeeding counselling, provision of high quality complementary food, and early child play stimulation after birth could play critical parts in improving child growth. |  |
| **Potential Behavioural mediating factors (as identified by authors of the primary studies)** | Preconcpetion positive thinking and problem solving skills mentioned but most mechanisms biological. Play / stimulation and breastfeeding after birth. |  |
| **Potential Social mediating factors (as identified by authors of the primary studies)** | NA Only psychosocial stimulation mentioned which is more behavioural. |  |
| **Details on who interventions are available to, and who is accessing services and in what numbers** | Apart from giving baseline characteristics who accessed what is not provided . 6722 received proconception intervention and 6778 did not receive it from first randomisation. 3594 and 3098 pregnancies detected in preconcpetion and no preconcpetion arms.  Of these 1326 & 1134 received pregnancy and early childhood care in preconcpetion & no precenceptio arms respectively. |  |
| **Author reflections on factors affecting the success of preconception nutrition interventions, including factors relating to leadership, financing, governance, supplies, and capacity** | Authors hypothesise that a complex, multifactorial problem like stunting could only be addressed by a complex intervention addressing key health, nutrition, psychosocial care, and environment issues at the same time. This theory is shown by the larger impact of our intervention compared with that seen in studies examining simple interventions. |  |
| **Author reflections on factors affecting the failure of preconception nutrition interventions, including factors relating to leadership, financing, governance, supplies, and capacity** | NA |  |
| **Author recommendations on strategies to improve preconception nutrition in South Asia** | Preconception interventions have major benefits for reducing the burden of infants with LBW and SGA. Strengthening the existing antenatal and early childhood programmes would reduce the risk of preterm births, SGA, and stunting at 24 months.  Integrating preconception interventions into current health systems coupled with early identification and management of pregnant women and infants who are at high risk should be a priority. These findings provide an opportunity for policymakers and managers to review and improve current programmes to improve women’s health and reduce adverse pregnancy outcomes and the burden of undernutrition in children younger than 24 months of age. Results are particularly generalisable to low and middle income populations in South Asia. |  |
| **Author recommendations on future research to improve preconception nutrition in South Asia** | Research on how to implement these interventions within routine systems to improve adverse growth outcomes like stunting seems necessary and should be a priority.  Implementation research studies are important in other low and middle income countries to assess the feasibility of delivering intervention packages effectively, identify context specific barriers, monitor quality and coverage of programmes, and improve community awareness and rollout. |  |
| **Barriers to Preconception nutritional intake** | Not covered. Authors suggest implementationr esearch is needed. |  |
| **Enablers to preconception nutritional intake** | Not covered. But counselling visits in which supplemental food was delivered at home enabled intake. |  |
| **Risk of Bias assessment ROB2 completed?** |  |  |
| **ABSTRACT** | Objective: To determine the effect of integrated and concurrent delivery of health, nutrition, water, sanitation and hygiene (WaSH), and psychosocial care interventions during the preconception period alone, during pregnancy and early childhood, and throughout preconception, pregnancy, and early childhood on birth outcomes and linear growth at 24 months of age compared with routine care. Design: Individually randomised factorial trial. Setting: Low and middle income neighbourhoods of Delhi, India. Participants: 13 500 women were randomised to receive preconception interventions (n=6722) or routine care (n=6778). 2652 and 2269 pregnant women were randomised again to receive pregnancy and early childhood interventions or routine care. The analysis of birth outcomes included 1290 live births for the preconception, pregnancy, and early childhood interventions (group A), 1276 for the preconception intervention (group B), 1093 for the pregnancy and early childhood interventions (group C), and 1093 for the control (group D). Children aged 24 months by 30 June 2021 were included in the 24 month outcome analysis (453 in group A, 439 in B, 293 in C, and 271 in D). Interventions: Health, nutrition, psychosocial care and support, and WaSH interventions were delivered during preconception, pregnancy, and early childhood periods. Main Outcome Measures: The primary outcomes were low birth weight, small for gestational age, preterm, and mean birth weight. At 24 months, the outcomes were mean length-for-age z scores and proportion stunted. Three prespecified comparisons were made: preconception intervention groups (A+B) versus no preconception intervention groups (C+D); pregnancy and early childhood intervention groups (A+C) versus routine care during pregnancy and early childhood (B+D) and preconception, pregnancy, and early childhood interventions groups (A) versus control group (D). Results: The proportion with low birth weight was lower in the preconception intervention groups (506/2235) than in the no preconception intervention groups (502/1889; incidence rate ratio 0.85, 98.3% confidence interval 0.75 to 0.97; absolute risk reduction −3.80%, 98.3% confidence interval −6.99% to −0.60%). The proportion with low birth weight was lower in the pregnancy intervention groups (502/2096) than in the no pregnancy intervention groups (506/2028) but the upper limit of the confidence interval crossed null effect (0.87, 0.76 to 1.01; −1.71%, −4.96% to 1.54%). There was a larger effect on proportion with low birth weight in the group that received interventions in the preconception and pregnancy periods (267/1141) compared with the control group (267/934; 0.76, 0.62 to 0.91; −5.59%, −10.32% to −0.85%). The proportion stunted at 24 months of age was substantially lower in the pregnancy and early childhood intervention groups (79/746) compared with the groups that did not receive these interventions (136/710; 0.51, 0.38 to 0.70; −8.32%, −12.31% to −4.32%), and in the group that received preconception, pregnancy, and early childhood interventions (47/453) compared with the control group (51/271; 0.49, 0.32 to 0.75; −7.98%, −14.24% to −1.71%). No effect on stunting at 24 months was observed in the preconception intervention groups (132/892) compared with the no preconception intervention groups (83/564).  Conclusions : An intervention package delivered during preconception, pregnancy, and early childhood substantially reduced low birth weight and stunting at 24 months. Pregnancy and early childhood interventions alone had lower but important effects on birth outcomes and 24 month outcomes. Preconception interventions alone had an important effect on birth outcomes but not on 24 month outcomes. Trial registration : Clinical Trial Registry—India CTRI/2017/06/008908. |  |

| **SN** | **17** | **18** |
| --- | --- | --- |
| **Short name** | Kumar2023 | Sethi2019 |
| **Type of paper** | Programme impact evaluation | Protocol |
| **Authors** | Abhishek Kumar, Vani Sethi, Arjan de Wagt, Rabi N. Parhi, Sourav Bhattacharjee, Sayeed Unisa, Reshmi R. S., Abhishek Saraswat, Nita Kejrewal, Monica Shrivastava, Lopamudra Tripathy, Zivai Murira, Sheila Vir on behalf of the Swabhimaan Study Group | Sethi, V., A. Bhanot, S. Bhattacharjee, R. Gope, D. Sarangi, V. Nath, N. Nair, U. Singh, A. Daniel, R. N. Parhi, S. Sinha, A. Loomba, S. S, A. Purty, N. Ali, B. Mohapatra, N. Agarwal, V. Bhatia, M. Ruikar, B. Sahu, S. R. R, S. Pedgaonkar, L. K. Dwivedi, F. Saiyed, M. Prajapati, P. Mishra, A. Prost, N. Kejrewal, A. De Wagt, H. Sachdev and S. Unisa |
| **Year** | 2023 | 2019 |
| **Title** | Evaluation of impact of engaging federations of women groups to improve women’s nutrition interventions- before, during and after pregnancy in social and economically backward geographies: Evidence from three eastern Indian States | Integrated multisectoral strategy to improve girls' and women's nutrition before conception, during pregnancy and after birth in India (Swabhimaan): protocol for a prospective, non-randomised controlled evaluation. |
| **Citation** | Kumar A, Sethi V, Wagt Ad, Parhi RN, Bhattacharjee S, Unisa S, et al. (2023) Evaluation of impact of engaging federations of women groups to improve women’s nutrition interventions before, during and after pregnancy in social and economically backward geographies: Evidence from three eastern Indian States. PLoS ONE 18(10): e0291866. | Sethi, V., A. Bhanot, S. Bhattacharjee, R. Gope, D. Sarangi, V. Nath, N. Nair, U. Singh, A. Daniel, R. N. Parhi, S. Sinha, A. Loomba, S. S, A. Purty, N. Ali, B. Mohapatra, N. Agarwal, V. Bhatia, M. Ruikar, B. Sahu, S. R. R, S. Pedgaonkar, L. K. Dwivedi, F. Saiyed, M. Prajapati, P. Mishra, A. Prost, N. Kejrewal, A. De Wagt, H. Sachdev and S. Unisa (2019). "Integrated multisectoral strategy to improve girls' and women's nutrition before conception, during pregnancy and after birth in India (Swabhimaan): protocol for a prospective, non-randomised controlled evaluation." BMJ Open 9(11): e031632. |
| **doi** | https://doi.org/10.1371/journal. pone.0291866 | 10.1136/ bmjopen-2019-031632 |
| **Clinical trials registration no** | 58261b2f46876 and CTRI/2016/11/007482 | 58261b2f46876 and CTRI/2016/11/007482 |
| **Context / setting** | Poorest areas in four districts Purnia in Bihar, Bastar in Chhattisgarh, and Angul and Koraput in Odisha in India:  Intervention arm included five sites covering 162 villages and control arm included five sites covering 151 villages.  The selected areas have a high burden of undernutrition, with significant populations of scheduled caste and tribal groups. Agriculture is the primary occupation and farmers rely heavily on rain-fed farming. These areas are designated as poverty blocks, with low literacy rates and limited awareness which contribute to excessive prevalence of undernutrition. | Poorest areas in four districts Purnia in Bihar, Bastar in Chhattisgarh, and Angul and Koraput in Odisha in India:  Intervention arm included five sites covering 162 villages and control arm included five sites covering 151 villages. |
| **Protocol citation** | Sethi, V., A. Bhanot, S. Bhattacharjee, R. Gope, D. Sarangi, V. Nath, N. Nair, U. Singh, A. Daniel, R. N. Parhi, S. Sinha, A. Loomba, S. S, A. Purty, N. Ali, B. Mohapatra, N. Agarwal, V. Bhatia, M. Ruikar, B. Sahu, S. R. R, S. Pedgaonkar, L. K. Dwivedi, F. Saiyed, M. Prajapati, P. Mishra, A. Prost, N. Kejrewal, A. De Wagt, H. Sachdev and S. Unisa (2019). "Integrated multisectoral strategy to improve girls' and women's nutrition before conception, during pregnancy and after birth in India (Swabhimaan): protocol for a prospective, non-randomised controlled evaluation." BMJ Open 9(11): e031632. |  |
| **Arising from which main trial** |  |  |
| **Study design** | Cross-sectional baseline and endline survey data analysis for evaluation of a non-randomised controlled trial. | Community-based intervention trial (non-randomised) |
| **Has Preconception intervention only arm (1/0)** |  |  |
| **Has Preconception + Pregnancy intervention arm (1/0)** | 1 (adolescent girls aged 10–19 years, pregnant women and mothers of children under 2) | 1 (adolescent girls aged 10–19 years, pregnant women and mothers of children under 2) |
| **Has Pregnancy intervention only arm (1/0)** |  |  |
| **Has Pregnancy, postpartum + childhood intervention arm (1/0)** | 1 | 1 |
| **Has Control arm with only usual standard of care or less intensive intervention in preconception & pregnancy (1/0)** | 1 (control sites receive only systems strength- ening interventions.) | 1 (control sites receive only systems strength- ening interventions.) |
| **MMN in Preconception arm** | 1 (calcium, iodised salt) |  |
| **B12 in Preconception arm** |  |  |
| **LNS in Preconception arm** |  |  |
| **Viatmin A in preconception arm** |  |  |
| **Iron in preconception arm (both arms had Folic acid)** | 1 | 1 |
| **Nutritious snack in Preconception arm** |  |  |
| **Pregnancy outcomes** |  |  |
| **Birth outcomes** |  |  |
| **Infancy outcomes** |  |  |
| **Child outcomes** |  |  |
| **Narrative summary of main finding** | In the intervention area at endline, 27–38% of women participated in the participatory learning and action meetings organized by women’s groups. Pregnant women participating in programme activities were two times more likely to receive an antenatal care visit in the first trimester of pregnancy (Odds ratio: 2.55 95% CI-1.68–3.88), while mothers of children under 2 were 60% more likely to receive 4 ANC visits (Odds ratio: 1.61, 95% CI- 1.30–2.02). Odds of consuming a diversified diet was higher among both pregnant women (Odds ratio: 2.05, 95% CI- 1.41–2.99) and mother of children under 2 years of age (Odds ratio: 1.38, 95% CI- 1.08–1.77) among those participating in programme activities in the intervention arm. Access to commodities for WASH including safe sanitation services (Odds ratio: 1.80, 95% CI- 1.38–2.36) and sanitary pads (Odds ratio: 1.64, 95% CI- 1.20–2.22) was higher among adolescent girls participating in programme activities. |  |
| **Date of data collection** | Baseline survey carried out during 2016–17 and endline survey carried out during (2021–22) |  |
| **Study objectives** | 1. To describe the implementation of the key interventions of the Swabhimaan programme across intervention and control areas. 2. To assess whether the programme has led to improvement in access to nutrition specific and nutrition sensitive interventions and nutritional outcomes. | This protocol describes the intervention and evaluation methods for the Swabhimaan programme, which aims to improve the nutrition status of adolescent girls and women in three Indian states: Bihar, Chhattisgarh and Odisha. |
| **Sample size / power** | A representative sample of 6250 adolescent girls (10–19 years), 2573 pregnant women (15– 49 years) and 8755 mothers of children under age two years (15–49 years) and their children (<2 years) were interviewed from five blocks of Bihar, Chhattisgarh, and Odisha at baseline. While for the endline survey, a representative sample of 4241 adolescent girls (10–19 years), 1698 pregnant women (15–49 years) and 4766 mothers of children under age two years (15–49 years) were interviewed from five blocks of Bihar, Chhattisgarh, and Odisha. The sample size accounted for a 10% non-response rate and 1.5 design effect.  10491 adolescent girls, 4271 pregnant women and 13521 mothers of children under age two years were included in the analysis. | Sample size accounted for 5% refusal rate and design effect of 1.5. Overall, we estimated that baseline and endline surveys should include a total of 6638 adolescent girls, 10160 mothers of children under 2 and 2992 pregnant women across the three states. |
| **Sample characteristics** |  |  |
| **Randomisation** | Across Bihar, Chhattisgarh and Odisha, five sites covering 162 villages (intervention arm) have **been purposively allocated** to community-led interventions delivered through VOs and CLFs since 2017, and five sites covering 151 villages (control arm) will initiate these activities 36 months later, in 2020. | Across Bihar, Chhattisgarh and Odisha, five sites covering 162 villages (intervention arm) have **been purposively allocated** to community-led interventions delivered through VOs and CLFs since 2017, and five sites covering 151 villages (control arm) will initiate these activities 36 months later, in 2020. |
| **Blinding** | It is not possible to blind participants to allocation, but data collection teams and analysts are blind to allocation. | It is not possible to blind participants to allocation, but data collection teams and analysts are blind to allocation. |
| **Age of participants (years)** | Adolescent girls (10–19 years), Pregnant women (15–49 years) and Mothers of children under age two years (15–49 years) |  |
| **Inclusion criteria** | adolescent girls, newlywed women and couples, pregnant women and mothers of children under 2 years of age. | adolescent girls, newlywed women and couples, pregnant women and mothers of children under 2 years of age. |
| **Exclusion criteria** |  |  |
| **Preconception intervention characteristics** | In Arm 1, community-led components: are delivered through community facilitators in nutrition, farming and (in Bihar only) for adolescents.   They facilitate Participatory Learning Action (PLA) meetings with Self Help Groups and village organisations and undertake nutrition microplanning with the community. - System strengthening (same as described in to Arm 2 - Control).  14 nutrition-specific and nutrition- sensitive interventions:  1.Access to generalised household ration through Public Distribution System, a food subsidy scheme. 2.Balanced energy protein supplementation through access to supplementary rations. 3. Access to knowledge and choices about how to increase maternal dietary diversity. 4. Access to knowledge and support for nutrition-sensitive agriculture at home (kitchen garden) and community-based food insecurity coping strategies. 5. IFA supplementation 6. Universal use of iodised salt. 7. Access to information and commodities like insecticide treated bed nets for malaria prevention. 8. Early registration in outreach services. 9. Quality reproductive health, antenatal and postnatal care. 10. Sanitation and hygiene (including menstrual hygiene) education. 11. Access to safe drinking water and sanitation commodities. 12. Promotion of secondary education and education for delaying the age at marriage to legal age. 13. Access to information and family planning commodities for delaying age at first pregnancy and prevention of repeated pregnancies. 14. Women’s collective voice and empowerment for decision-making to prevent child marriage, violence against women, child spacing and other gender-related issues. The system strengthening activities include five components: 1. Strengthening VHSNDs to improve access to antenatal care, family planning and micronutrient supplemen- tation through quarterly trainings of health service providers, monthly review of nutrition indicators, and the identification of women at risk of undernutrition (MUAC <23cm) for special supplementary food and counselling. 2. Strengthening adolescent health days to improve ac- cess to adolescent health and nutrition services via quarterly trainings of health service providers. 3. An extended VHSND once every 6months for newly weds and women, including individual counselling and information about entitlement camps. 4. Annual training and follow-up meetings with service providers from food security, Integrated Child Devel- opment Services (ICDS), water and sanitation depart- ments to help them improve the delivery of entitle- ments and services. 5. Ensuring regular review meetings with representation across government departments involved in service delivery. | 14 nutrition-specific and nutrition- sensitive interventions:  1.Access to generalised household ration through Public Distribution System, a food subsidy scheme. 2.Balanced energy protein supplementation through access to supplementary rations. 3. Access to knowledge and choices about how to increase maternal dietary diversity. 4. Access to knowledge and support for nutrition-sensitive agriculture at home (kitchen garden) and community-based food insecurity coping strategies. 5. IFA supplementation 6. Universal use of iodised salt. 7. Access to information and commodities like insecticide treated bed nets for malaria prevention. 8. Early registration in outreach services. 9. Quality reproductive health, antenatal and postnatal care. 10. Sanitation and hygiene (including menstrual hygiene) education. 11. Access to safe drinking water and sanitation commodities. 12. Promotion of secondary education and education for delaying the age at marriage to legal age. 13. ccess to information and family planning commodities for delaying age at first pregnancy and prevention of repeated pregnancies. 14. Women’s collective voice and empowerment for decision-making to prevent child marriage, violence against women, child spacing and other gender-related issues. The system strengthening activities include five components: 1. Strengthening VHSNDs to improve access to antenatal care, family planning and micronutrient supplemen- tation through quarterly trainings of health service providers, monthly review of nutrition indicators, and the identification of women at risk of undernutrition (MUAC <23cm) for special supplementary food and counselling. 2. Strengthening adolescent health days to improve ac- cess to adolescent health and nutrition services via quarterly trainings of health service providers. 3. An extended VHSND once every 6months for newly weds and women, including individual counselling and information about entitlement camps. 4. Annual training and follow-up meetings with service providers from food security, Integrated Child Devel- opment Services (ICDS), water and sanitation depart- ments to help them improve the delivery of entitle- ments and services. 5. Ensuring regular review meetings with representation across government departments involved in service delivery. |
| **Preconception control group intervention** | Control sites receive only systems strength- ening interventions. The system strengthening activities include five components: 1. Strengthening VHSNDs to improve access to antenatal care, family planning and micronutrient supplemen- tation through quarterly trainings of health service providers, monthly review of nutrition indicators, and the identification of women at risk of undernutrition (MUAC <23cm) for special supplementary food and counselling. 2. Strengthening adolescent health days to improve ac- cess to adolescent health and nutrition services via quarterly trainings of health service providers. 3. An extended VHSND once every 6months for newly weds and women, including individual counselling and information about entitlement camps. 4. Annual training and follow-up meetings with service providers from food security, Integrated Child Devel- opment Services (ICDS), water and sanitation depart- ments to help them improve the delivery of entitle- ments and services. 5. Ensuring regular review meetings with representation across government departments involved in service delivery. | Control sites receive only systems strength- ening interventions. |
| **Intervention timing (how far before conception)** | information not available |  |
| **Intervention delivery (health systems, food systems, social protection systems)** | Interventions are delivered through a combination of community-led and systems-led efforts. Community-led interventions are delivered through trained community cadre who are members of VOs, namely Poshan Sakhis (lit. ‘Nutrition sister/friend’) or Community Resource Persons (CRPs) and Krishi Mitras (lit. farmer friends) or Village Resource Persons. In Bihar, a separate cadre of Kishori Sakhis (lit. ‘Adolescent sister/friend’) for reaching out and serving adolescent girls has been created. | Interventions are delivered through a combination of community-led and systems-led efforts. Community-led interventions are delivered through trained community cadre who are members of VOs, namely Poshan Sakhis (lit. ‘Nutrition sister/friend’) or Community Resource Persons (CRPs) and Krishi Mitras (lit. farmer friends) or Village Resource Persons. In Bihar, a separate cadre of Kishori Sakhis (lit. ‘Adolescent sister/friend’) for reaching out and serving adolescent girls has been created. |
| **Pregnancy detection** | information not available |  |
| **Pregnancy intervention characteristics (if any)** | Same PLA intervention as described for preconception arm1 plus 17 nutrition-specific and nutrition- sensitive interventions:  1.Access to generalised household ration through Public Distribution System, a food subsidy scheme. 2.Balanced energy protein supplementation through access to supplementary rations. 3. Access to knowledge and choices about how to increase maternal dietary diversity. 4. Access to knowledge and support for nutrition-sensitive agriculture at home (kitchen garden) and community-based food insecurity coping strategies. 5. IFA supplementation 6. Universal use of iodised salt. 7. Calcium supplementation and deworming 8. Access to information and commodities like insecticide treated bed nets for malaria prevention. 9. Access to information on preventing tobacco and alcohol use in pregnancy 10. Early registration in outreach services. 11. Recording and monitoring of nutritional status and special community-based at-nutritional risk package. 12. Quality reproductive health, antenatal and postnatal care. 13. Access to knowledge and entitlements for promotion of institutional deliveries and maternity benefits. 14. Sanitation and hygiene (including menstrual hygiene) education. 15. Access to safe drinking water and sanitation commodities. 16. ccess to information and family planning commodities for delaying age at first pregnancy and prevention of repeated pregnancies. 17. Women’s collective voice and empowerment for decision-making to prevent child marriage, violence against women, child spacing and other gender-related issues. The system strengthening activities include five components: 1. Strengthening VHSNDs to improve access to antenatal care, family planning and micronutrient supplemen- tation through quarterly trainings of health service providers, monthly review of nutrition indicators, and the identification of women at risk of undernutrition (MUAC <23cm) for special supplementary food and counselling. 2. Strengthening adolescent health days to improve ac- cess to adolescent health and nutrition services via quarterly trainings of health service providers. 3. An extended VHSND once every 6months for newly weds and women, including individual counselling and information about entitlement camps. 4. Annual training and follow-up meetings with service providers from food security, Integrated Child Devel- opment Services (ICDS), water and sanitation depart- ments to help them improve the delivery of entitle- ments and services. 5. Ensuring regular review meetings with representation across government departments involved in service delivery. | 17 nutrition-specific and nutrition- sensitive interventions:  1.Access to generalised household ration through Public Distribution System, a food subsidy scheme. 2.Balanced energy protein supplementation through access to supplementary rations. 3. Access to knowledge and choices about how to increase maternal dietary diversity. 4. Access to knowledge and support for nutrition-sensitive agriculture at home (kitchen garden) and community-based food insecurity coping strategies. 5. IFA supplementation 6. Universal use of iodised salt. 7. Calcium supplementation and deworming 8. Access to information and commodities like insecticide treated bed nets for malaria prevention. 9. Access to information on preventing tobacco and alcohol use in pregnancy 10. Early registration in outreach services. 11. Recording and monitoring of nutritional status and special community-based at-nutritional risk package. 12. Quality reproductive health, antenatal and postnatal care. 13. Access to knowledge and entitlements for promotion of institutional deliveries and maternity benefits. 14. Sanitation and hygiene (including menstrual hygiene) education. 15. Access to safe drinking water and sanitation commodities. 16. ccess to information and family planning commodities for delaying age at first pregnancy and prevention of repeated pregnancies. 17. Women’s collective voice and empowerment for decision-making to prevent child marriage, violence against women, child spacing and other gender-related issues. The system strengthening activities include five components: 1. Strengthening VHSNDs to improve access to antenatal care, family planning and micronutrient supplemen- tation through quarterly trainings of health service providers, monthly review of nutrition indicators, and the identification of women at risk of undernutrition (MUAC <23cm) for special supplementary food and counselling. 2. Strengthening adolescent health days to improve ac- cess to adolescent health and nutrition services via quarterly trainings of health service providers. 3. An extended VHSND once every 6months for newly weds and women, including individual counselling and information about entitlement camps. 4. Annual training and follow-up meetings with service providers from food security, Integrated Child Devel- opment Services (ICDS), water and sanitation depart- ments to help them improve the delivery of entitle- ments and services. 5. Ensuring regular review meetings with representation across government departments involved in service delivery. |
| **Pregnancy control group intervention (if any)** | Control sites receive only systems strength ening interventions as described for preconception. | Control sites receive only systems strength- ening interventions. |
| **Interventions after birth (if any)** | 15 nutrition-specific and nutrition- sensitive interventions:  1.Access to generalised household ration through Public Distribution System, a food subsidy scheme. 2.Balanced energy protein supplementation through access to supplementary rations. 3. Access to knowledge and choices about how to increase maternal dietary diversity. 4. Access to knowledge and support for nutrition-sensitive agriculture at home (kitchen garden) and community-based food insecurity coping strategies. 5. IFA supplementation 6. Universal use of iodised salt. 7. Calcium supplementation and deworming 8. Access to information and commodities like insecticide treated bed nets for malaria prevention. 9. Access to information on preventing tobacco and alcohol use in pregnancy 10. Quality reproductive health, antenatal and postnatal care. 11. Access to knowledge and entitlements for promotion of institutional deliveries and maternity benefits. 12. Sanitation and hygiene (including menstrual hygiene) education. 13. Access to safe drinking water and sanitation commodities. 14. ccess to information and family planning commodities for delaying age at first pregnancy and prevention of repeated pregnancies. 15. Women’s collective voice and empowerment for decision-making to prevent child marriage, violence against women, child spacing and other gender-related issues. The system strengthening activities include five components: 1. Strengthening VHSNDs to improve access to antenatal care, family planning and micronutrient supplemen- tation through quarterly trainings of health service providers, monthly review of nutrition indicators, and the identification of women at risk of undernutrition (MUAC <23cm) for special supplementary food and counselling. 2. Strengthening adolescent health days to improve ac- cess to adolescent health and nutrition services via quarterly trainings of health service providers. 3. An extended VHSND once every 6months for newly weds and women, including individual counselling and information about entitlement camps. 4. Annual training and follow-up meetings with service providers from food security, Integrated Child Devel- opment Services (ICDS), water and sanitation depart- ments to help them improve the delivery of entitle- ments and services. 5. Ensuring regular review meetings with representation across government departments involved in service delivery. | 15 nutrition-specific and nutrition- sensitive interventions:  1.Access to generalised household ration through Public Distribution System, a food subsidy scheme. 2.Balanced energy protein supplementation through access to supplementary rations. 3. Access to knowledge and choices about how to increase maternal dietary diversity. 4. Access to knowledge and support for nutrition-sensitive agriculture at home (kitchen garden) and community-based food insecurity coping strategies. 5. IFA supplementation 6. Universal use of iodised salt. 7. Calcium supplementation and deworming 8. Access to information and commodities like insecticide treated bed nets for malaria prevention. 9. Access to information on preventing tobacco and alcohol use in pregnancy 10. Quality reproductive health, antenatal and postnatal care. 11. Access to knowledge and entitlements for promotion of institutional deliveries and maternity benefits. 12. Sanitation and hygiene (including menstrual hygiene) education. 13. Access to safe drinking water and sanitation commodities. 14. ccess to information and family planning commodities for delaying age at first pregnancy and prevention of repeated pregnancies. 15. Women’s collective voice and empowerment for decision-making to prevent child marriage, violence against women, child spacing and other gender-related issues. The system strengthening activities include five components: 1. Strengthening VHSNDs to improve access to antenatal care, family planning and micronutrient supplemen- tation through quarterly trainings of health service providers, monthly review of nutrition indicators, and the identification of women at risk of undernutrition (MUAC <23cm) for special supplementary food and counselling. 2. Strengthening adolescent health days to improve ac- cess to adolescent health and nutrition services via quarterly trainings of health service providers. 3. An extended VHSND once every 6months for newly weds and women, including individual counselling and information about entitlement camps. 4. Annual training and follow-up meetings with service providers from food security, Integrated Child Devel- opment Services (ICDS), water and sanitation depart- ments to help them improve the delivery of entitle- ments and services. 5. Ensuring regular review meetings with representation across government departments involved in service delivery. |
| **Control group intervention after birth (if any)** | Control sites receive only systems strengthening interventions as described in preconcpetion. | Control sites receive only systems strength- ening interventions. |
| **Compliance measurement** | Participation in AHD and VHSND was observed to be higher in the intervention area as compared to the control area. A higher percentage of mothers of children under 2 years (33.4%) attended AHD or VHSNDs and PLA meetings. While 28% of pregnant women and 19% adolescent girls attended both in the intervention area, participation of adolescent girls in AHD was low in the control area (14.7%). |  |
| **Adverse events** | No adverse events related to the intervention were reported. |  |
| **Engagement, category (including nutrition sensitive/specific)** | Nutrition specific & nutrition sensitive |  |
| **Where in the overall UNICEF 2020 framework the intervention lies** | 1. IFA supplementation, 2. Universal use of iodised salt, 3. Calcium supplementation and deworming and 4. Balanced energy protein supplementation through access to supplementary rations  affect immediate determinant "Diet";  5. Access to generalised household ration through Public Distribution System, a food subsidy scheme, 6. Access to knowledge and choices about how to increase maternal dietary diversity, and 7. Access to knowledge and support for nutrition-sensitive agriculture at home (kitchen garden) and community-based food insecurity coping strategies affect underlying determinant "Food"; 8. Sanitation and hygiene (including menstrual hygiene) education affect underlying determinant "Practices"; 9. Access to afe drinkign water and sanitation commodities, 10. Quality reproductive health, antenatal and postnatal care, 11. Access to knowledge and entitlements for the promotion of institutional deliveries and maternity benefits, 12. Promotion of secondary education and education for delaying the age at marriage to the legal age, 13. Early registration for outreach services, 14. Access to information and family planning commodities for delaying age at first pregnancy and prevention of repeated pregnancies, 15. Recording and monitoring of nutritional status and special community-based at-nutritional risk package, 16. Access to information on preventing tobacco and alcohol use in pregnancy, and 17. Access to information and commodities like insecticide-treated bed nets for malaria prevention affect underlyign determinant "Services"; 18. Women’s collective voice and empowerment for decision-making to prevent child marriage, violence against women, child spacing and other gender-related issues affect enabling determinant "Norms". |  |
| **Where in the Partap 2021 framework the intervention lies** | Individual screening, treatment and home visiting and counselling is working at individual level witin health and nutrition systms. Group BCC approaches at VHSNDs are affecting hosuehold and community-level factors as well as individual factors in health, nutrition and social domains. |  |
| **Proposed mechanism of intervention action** |  |  |
| **Exposures relating to preconception nutrition** |  |  |
| **Preconception measures** |  | Per cent adolescent girls with body mass index <18.5 kg/m2. |
| **Concommittant care** | None |  |
| **Follow-up schedule for measurements** | None |  |
| **Summary of baseline comparisons by study arm** | Reported from endline survey. The distribution does not vary across control and intervention area. In the inter- vention area, 51.3% of adolescent girls were aged 15–19 years while 66.1% of pregnant women and 69.7% of mothers of children under two years were 20–29 years of age. In control area, 51% of adolescent girls were aged 15–19 years while 69.6% of pregnant women and 68.9% of mothers of children under two years were 20–29 years of age. One in three adolescent girls were not attending school in control and intervention area. More than 30% of pregnant women and mothers of children under 2 had no education across both control and intervention areas. More than 70% of adolescent girls and women were Hindu and one-fourth were from the Schedule Caste category. | Not reported in protocol. |
| **Pregnancy outcomes: gestational weight gain, gestational diabetes, maternal anthropometry (weight, MUAC, height), anaemia,** | Primary: Mid-Upper Arm Circumference (MUAC) with cut-offs of <23 cm among pregnant women Secondary: 1. Per cent of pregnant women in the second and third trimester con- suming at least 25 IFA tablets in the month preceding the survey. 2. Mean dietary diversity score. 3. Per cent receiving minimum dietary diversity (5 out of 10 food groups). 4. Per cent living in a household with iodised salt. 5. Per cent living in food secure households. 6. Per cent living in households with a kitchen garden. 7. Per cent living in households with a toilet or covered pit latrine. 8. Per cent receiving Integrated Child Development Services (ICDS) entitlement for supplementary food in month preceding the survey. 9. Per cent who had one antenatal check-up in the first trimester. 10. Per cent weighed at least once in first trimester. 11. Per cent who received one dose of albendazole in second trimester. 12. Per cent who took two calcium tablets in second trimester. 13. Per cent below the age of 18. 14. Per cent who attended at least three Maitri Bethak meetings in 6 months. 15. Per cent who attended at least three Maitri Bethak meetings in 6 months. 16. Per cent who attended at least three Village Health Sanitation and Nutrition Days (VHSNDs) in 6 months. 17. Per cent who attended at least three VHSNDs in 6 months. 18. Per cent using a modern family planning method (in previous delivery), before the current pregnancy. 19. Per cent who are members of women’s farmer producer groups and have adopted at least one mix micronutrient-rich cropping methods, against previous practice. 20. Per cent who are members of women’s farmer producer groups and have adopted at least one pesticide-free agrimethods, against previous practice. | Mid-Upper Arm Circumference (MUAC) with cut-offs of <23 cm among pregnant women |
| **Preconceptual outcomes: (BMI, Hb or other outcomes which could be attributed to preconception care)** | Primary: BMI was used to assess the nutrition status of adolescent girls  Secondary:  1. Mean dietary diversity score. 2. Per cent receiving minimum dietary diversity score (5 of 10 food groups). 3. Per cent consuming four or more IFA tablets in the month preced- ing the survey. 4. Per cent living in a household with iodised salt. 5. Per cent living in food secure households. 6. Per cent living in households with a kitchen garden. 7. Per cent living in households with a toilet or covered pit latrine. 8. Per cent using safe pads or sanitary pads. 9. Per cent accessing adolescent health services (Kishori Divas) in 6 months preceding the survey. 10. PercentwhoattendedatleastthreeKishorimeetingsin6months. 11. Per cent who attended at least three Kishori meetings in in 6 months. | BMI |
| **Birth outcomes in child: birthweight, low birth weight (LBW), small for gestational age (SGA), preterm birth/ delivery (PTB), cord blood b12, DXA,** | None | None |
| **Outcomes in mother at birth** | Primary: BMI was used to assess the nutrition status of mother of children under 2 years. Secondary: 1. Per cent receiving minimum dietary diversity (5 out of 10 food groups). 3. Per cent living in a household with iodised salt. 4. Per cent living in food secure households. 5. Per cent living in households with a kitchen garden. 6. Per cent living in households with a toilet or covered pit latrine. 7. Per cent receiving their minimum Public Distribution System enti- tlement in month preceding survey. 8. Per cent receiving ICDS entitlement for supplementary food in month preceding survey. 9. Per cent who received at least four antenatal care overall in last pregnancy. 10. Per cent consuming 100 or more IFA tablets during last pregnancy. 11. Per cent weighed at least four times in last pregnancy. 12. Per cent using a modern family planning method. 13. Per cent who accessed at least one of three social protection schemes (JSY, Adarsh Dampati Yojana). 14. Per cent who delivered in a health facility in last pregnancy. 15. Per cent who attended at least three Maitri Bethak meetings and three VHSNDs in last year. 16. Per cent who attended at least three Maitri Bethak meetings and three VHSNDs in last year. 17. Per cent who are members of women’s farmer producer groups and have adopted at least 1one mix micronutrient-rich cropping methods, against previous practice. 18. Per cent who are members of women’s farmer producer groups and have adopted at least 1one pesticide-free agrimethods, against previous practice. | Per centage of mothers of children under 2 with BMI <18.5 kg/m2. |
| **Post-natal outcomes (in Infancy)** | None | None |
| **Outcomes in childhood after 12 m: z scores for weight (WAZ), length/height (HAZ), and BMI for age (BMIZ), weight-for-height'length (WHZ), underweight, stunting, wasting, overweight, cognitive** | None | None in protocol |
| **Long term outcomes** | None |  |
| **Analysis** | The differential effects were tested by using regression models that estimated changes over time between the 2 areas: intervention and Control area. To establish the association between improvement in service uptake and participation, a logistic regression model was estimated Logistic regression models were estimated for nutrition outcome and services with a better outcome being designated value 1 and 0 otherwise. Full adjusted models were estimated with frequency of participation as the independent variable. | Assess the comparability of intervention and control areas at baseline by examining area-level and individual-level characteristics, including: the number of SHGs and VOs in each area, the sociodemographic. The final impact analysis will be by intention to treat, comparing primary and secondary outcomes in five inter- vention areas and five control areas. This analysis will be carried out at an individual level, adjusting for clustering at the level of the village and VO, using linear and logistic random effects models.  Use the difference-in-difference method to compare primary and secondary outcomes between intervention and control areas at endline, adjusting for their baseline values and for other characteristics that differed significantly between the two areas at baseline. |
| **EFFECTS on Preconception outcomes: maternal anthropometry (weight, MUAC, height. BMI), Haemoglobin (Hb), anaemia, iodine status** | % Thin (BMI <18.5) : decreased from 18 to 13 (int arm) vs 15 to 12 (contl arm) % Experiencing double burden of short and thin decreased from 8 to 4 vs 6 to 4 Secondary outcomes:  % Minimum dietary diversity (6 out of 10 food groups): increased from 18 to 39 vs 18 to 37 % living in a household with iodised salt: increased from 80 to 98 vs 76 to 98 % living in households with a kitchen garden: increased from 43% to 52% vs 36% to 47% % living in households which do not practice open defecation: increased from 18 to 59 vs 18 to 60. % using safe pads or sanitary pads: increased from 38 to 73 vs 38 to 67. p<0.05 % consuming four or more IFA tablets in the month preceding the survey: increased from 9 to 18 vs 12 to 18 % consumed deworming tablets increased from 48 to 75 vs 48 to 70 % Adolescent girls who visit Anganwadi Centre (AWC) for any service: increased from 22 to 42 vs 29 to 32 P<0.01 |  |
| **EFFECTS on Pregnancy outcomes: gestational weigt gain, gestational diabetes, maternal anthropometry (weight, MUAC, height), anaemia, iodine status** | % Mid-Upper Arm Circumference (MUAC) with cut-offs of <23 cm: decreased from 41 to 37 vs 45 to 35 Secondary: % receiving minimum dietary diversity (6 out of 10 food groups): increased from 21 to 44 vs 21 to 39 % living in a household with iodised salt: increased from 79 to 99 vs 74 vs 96 % living in food secure households: increased from 25 to 35 vs 22 to 35 % living in households with a kitchen garden: increased from 36 to 50 vs 31 ro 39.  % Received ICDS entitlement for supplementary food in month preceding survey: increased from 28 to 65 vs 33 to 55. P<0.01 % living in households which do not practice open defeacation: increased from 16 to 57 vs 13 to 59. % consuming 25 or more IFA tablets in second or third trimester: increased from 24 to 64 vs 25 to 63 % two calcium tablets in second trimester: increased from 17 to 62 vs 23 to 54. P<0.01 % using a modern family planning method, before the current pregnancy: increased from 7 to 17 vs 7 to 13 % Decisions about their own health care: increased from 73 to 74 vs decreased from 73 to 70 % Decisions about making major purchases for the household: increased from 71 to 73 vs decreased from 73 to 70 % Taking decisions about visits to family members or relatives: increased from 66 to 72 vs 66 to 68 % first antenatal check-up in the first trimester: increased from 41 to 68 vs 48 to 58. P<0.01 % weighed at least once in first trimester: 73 vs 86 to 79 vs 76. P<0.01 % height was recorded: increased from 10 to 32 vs 11 to 21. P<0.01 |  |
| **EFFECTS on Birth outcomes in child: birthweight, low birth weight (LBW), small for gestational age (SGA), preterm birth/ delivery (PTB)** | None |  |
| **EFFECTS on Outcomes in mother at birth** | % Mothers who are thin (<18.5) decreased from 49 to 38 vs 50 to 39 Secondary: % receiving minimum dietary diversity (5 out of 10 food groups): increased from 19 to 44 % living in a household with iodised salt. increased from 76 to 98 % living in food secure households: increased form 22 to 29 % living in households with a kitchen garden: increased from 33 to 52 vs 30 to 38. P<0.01 % receiving their minimum Public Distribution System entitlement in month preceding survey. increased from 70 to 94 % receiving ICDS entitlement for supplementary food in month preceding survey. increased from 45 to 79 vs 50 to 76. P<0.01 %t living in households which do not practice open defeacation: increased from 20 to 59 % consuming 100 or more IFA tablets during last pregnancy. increased form 22 to 39. P<0.01 % consumed 100 or more calcium tablets last pregnancy increased from 0.1 to 29 % using a modern family planning method. increased from 13 to 31. P<0.01 % Decisions about their own health care: increased from 67 to 72 vs from 70 to 75 % Decisions about making major purchases for the household: increased from 69 to 72 vs 72 to 73 % Taking decisions about visits to family members or relatives: increased from 65 to 71 vs 70 to 74 % first antenatal check-up in the first trimester: increased from 30 to 65 vs 26 to 59 % received at least four antenatal care overall in last pregnancy. increased from 18 to 43 vs 21 to 39. P<0.01 % weighed at least four times in last pregnancyr: 22 vs 36 to 25 vs 31. P<0.01 % height was recorded: increased from 15 to 39 vs 13 to 27. P<0.01 % who accessed JSY: increased from 52 to 54 vs decreased from 53 to 48. P<0.01 % who delivered in a health facility in last pregnancy. increased from 73 to 84 vs 69 to 76 |  |
| **EFFECTS on Post-natal outcomes (in Infancy)** | None |  |
| **EFFECTS on Outcomes in childhood after 12 m: z scores for weight (WAZ), length/height (HAZ), and BMI for age (BMIZ), weight-for-height'length (WHZ), underweight, stunting, wasting, overweight, cognitive** | None |  |
| **Potential Biological mediating factors (as identified by authors of the primary studies)** |  |  |
| **Potential Behavioural mediating factors (as identified by authors of the primary studies)** |  |  |
| **Potential Social mediating factors (as identified by authors of the primary studies)** |  |  |
| **Details on who interventions are available to, and who is accessing services and in what numbers** |  |  |
| **Author reflections on factors affecting the success of preconception nutrition interventions, including factors relating to leadership, financing, governance, supplies, and capacity** | Participation status was associated with higher odds of consumption of micronutrients across all the groups and higher chances of utilization of health services among pregnant woman and mother of children under 2 years. The nutition outcomes were slightly better for adolescent girls which could be attributable to the use of cadre of resource persons specifically trained for working with adolescent girls in one of the states. However, the impact on nutritional status was noted to be minimal.  The role of community resource persons in identifying at risk household with adolescent girls, pregnant women and mothers of under twos through a simple measure of MUAC measure fol- lowed by microplanning and targeting efforts towards mobilising and improving their partici- pation in group meetings present evidence that engaging a dedicated cadre selected from within women collectives increases demands as well response by the relevant sectors to improve the coverage of health and nutrition services. |  |
| **Author reflections on factors affecting the failure of preconception nutrition interventions, including factors relating to leadership, financing, governance, supplies, and capacity** |  |  |
| **Author recommendations on strategies to improve preconception nutrition in South Asia** | Community resource persons (Poshan Sakhis and Kishori Sakhis) can play a positive role in mobilizing communities and identifying at-risk households. But the outcome could be context specific which implies that extensive consultations with communities and formative research should be conducted to develop consensus about the hypothesized pathways of impact. |  |
| **Author recommendations on future research to improve preconception nutrition in South Asia** | It is strongly recommended that the impact evaluation of similar interventions in future should be carried out over extended period to produce more useful information about the sustainability of the desired effects. It should be ensured that beneficiaries receive the intended duration and intensity of exposure to the pro- gramme activities before assessment. This will ensure a more accurate and reliable assessment of the program’s effectiveness and impact |  |
| **Barriers to Preconception nutritional intake** |  |  |
| **Enablers to preconception nutritional intake** |  |  |
| **Risk of Bias assessment ROB2 completed?** |  |  |
| **ABSTRACT** | Background: Undernutrition–before, during and after pregnancy endangers the health and well-being of the mother and contributes to sub-optimal fetal development and growth. A non-randomized controlled evaluation was undertaken to assess the impact of engaging federations of wom- en’s group on coverage of nutrition interventions and on nutrition status of women in the des- ignated poverty pockets of three Indian states—Bihar, Chhattisgarh, and Odisha.  Method: The impact evaluation is based on two rounds of cross-sectional data from 5 resource poor blocks across 3 States, assigning 162 villages to the intervention arm and 151 villages to the control arm. The cross-sectional baseline (2016–17) and endline survey (2021–22) covered a total of 10491 adolescent girls (10–19 years), 4271 pregnant women (15–49 years) and 13521 mothers of children under age two years (15–49 years). Exposure was defined based on participation in the participatory learning and action meetings, and fixed monthly health camps (Adolescent Health Days (AHDs) and Village Health Sanitation and Nutrition Days (VHSNDs)). Logistic regression models were applied to establish the association between exposure to programme activities and improvement in coverage of nutrition interventions and outcomes. Results: In the intervention area at endline, 27–38% of women participated in the participatory learn- ing and action meetings organized by women’s groups. Pregnant women participating in programme activities were two times more likely to receive an antenatal care visit in the first trimester of pregnancy (Odds ratio: 2.55 95% CI-1.68–3.88), while mothers of children under 2 were 60% more likely to receive 4 ANC visits (Odds ratio: 1.61, 95% CI- 1.30–2.02). Odds of consuming a diversified diet was higher among both pregnant women (Odds ratio: 2.05, 95% CI- 1.41–2.99) and mother of children under 2 years of age (Odds ratio: 1.38, 95% CI- 1.08–1.77) among those participating in programme activities in the intervention arm. Access to commodities for WASH including safe sanitation services (Odds ratio: 1.80, 95% CI- 1.38–2.36) and sanitary pads (Odds ratio: 1.64, 95% CI- 1.20–2.22) was higher among adolescent girls participating in programme activities. Conclusion: Women’s groups led participatory learning and action approaches coupled with strengthen- ing of the supply side delivery mechanisms resulted in higher coverage of health and nutri- tion services. However, we found that frequency of participation was low and there was limited impact on the nutritional outcomes. Therefore, higher frequency of participation in programme activities is recommended to modify behaviour and achieve quick gains in nutri- tional outcomes. | Introduction Swabhimaan is a community-based programme to improve adolescent girls’ and women’s nutrition in the rural areas of three Indian states— Bihar, Chhattisgarh and Odisha with high prevalence of undernutrition. Methods and analysis Swabhimaan has a nested prospective, non-randomised controlled evaluation. Since 2017, five intervention sites receive community- led interventions through national government’s livelihood mission supported women’s self-help group federations and five control sites will initiate these activities 36 months later, in 2020. Community-led activities aim to improve coverage of 18 interventions including adequacy of food consumed, prevention of micronutrient deficiencies, access to basic health services and special care of nutritionally ‘at risk’ girls and women, improving hygiene and access to water and sanitation services and access to family planning services. The evaluation includes baseline (2016–2017), midline (2018–2019) and endline (2020–2021) surveys covering 6638 adolescent girls, 2992 pregnant women and 8755 mothers of children under 2. The final impact analysis will be by intention to treat, comparing primary and secondary outcomes in five intervention areas and five control areas. The primary outcomes are: (1) a 15% reduction in the proportion of adolescent girls with a body mass index (BMI) <18.5 kg/m2; (2) a 15% reduction in the proportion of mothers of children under two with a BMI <18.5 kg/m2 and (3) and a 0.4 cm improvement in mean mid-upper arm circumference among pregnant women. |

| **SN** | **19** | **20** |
| --- | --- | --- |
| **Short name** | HealthyParents2021 | Doke 2024 |
| **Type of paper** | Government Report |  |
| **Authors** | Government of Maharastra, UNICEF, Bharati Vidyapeeth (Deemed to be University) Medical College Pune. | Prakash Prabhakarrao Doke a, Amruta Paresh Chutke a, Sonali Hemant Palkar a, Jayashree Sachin Gothankar a, Prasad Dnyandeo Pore a, Archana Vasantrao Patil b, Aniruddha Vinayakrao Deshpande b, Khanindra Kumar Bhuyanc, Madhusudan Vaman Karnataki d, , Aparna Nishikant Shrotri d, , Ravindra Gopal Chaudhari e, Mohan Sitaram Bacchav e, , Motilal Bajirao Patil e, Rupeshkumar Balasaheb Deshmukh a |
| **Year** | 2021 | 2024 |
| **Title** | Healthy Parents Healthy Child Inititiative: Report of intervention that promoted health of women desiring pregnancy and reduced Low Birth Weight and New Born Deaths in Nashik, Maharashtra, India | Implementation of preconception care for preventing adverse pregnancy outcomes in rural and tribal areas of Nashik District, India |
| **Citation** |  |  |
| **doi** | NA | <https://doi.org/10.1016/j.pmedr.2024.102796> |
| **Clinical trials registration no** | Clinical Trial Registry—India CTRI/2018/06/014658 dated 28 Jun 2018 | CTRI/2018/06/014658 dated 28 Jun 2018 |
| **Context / setting** | Intervention: Peint (Tribal) & Sinnar (non-tribal) blocks  Control: Trimbakeswar (Tribal) & Niphad (non-tribal) blocks of Nashik district in Maharastra, India | Four blocks of Nashik district, Maharashtra State, India. We selected one tribal block (block having a high proportion of the Scheduled Tribes population) for intervention and an adjacent tribal block for comparison. Similarly, we chose one non tribal block for intervention and one adjacent non-tribal block for comparison. There were 13 Primary Health Centers (PHCs) in the intervention and 15 in the comparison blocks. Detailed information about the study setting has already been reported. |
| **Protocol citation** | No separate protocol. | Not protocol but the study cites formative study for study setting and participants related design: Doke, P.P., et al., 2022. Prevalence of preconception risk factors for adverse pregnancy outcome among women from tribal and non-tribal blocks in Nashik district, India: a cross-sectional study. Reprod. Health 19 (1), 1–11. https://doi.org/10.1186/s12978- 022-01473-z. |
| **Arising from which main trial** | Healthy Parents Healthy Child Inititiative |  |
| **Study design** | Community-based intervention trial (non-randomised) | The community-based implementation study included two randomly selected blocks in the intervention and two adjacent blocks in the comparison arm. |
| **Has Preconception intervention only arm (1/0)** | 1 | 1 |
| **Has Preconception + Pregnancy intervention arm (1/0)** |  |  |
| **Has Pregnancy intervention only arm (1/0)** |  |  |
| **Has Pregnancy, postpartum + childhood intervention arm (1/0)** |  |  |
| **Has Control arm with only usual standard of care or less intensive intervention in preconception & pregnancy (1/0)** | 1 |  |
| **MMN in Preconception arm** |  |  |
| **B12 in Preconception arm** |  |  |
| **LNS in Preconception arm** |  |  |
| **Viatmin A in preconception arm** |  |  |
| **Iron in preconception arm (both arms had Folic acid)** | 1 |  |
| **Nutritious snack in Preconception arm** |  |  |
| **Pregnancy outcomes** |  |  |
| **Birth outcomes** | 1 |  |
| **Infancy outcomes** |  |  |
| **Child outcomes** |  |  |
| **Narrative summary of main finding** |  | The study enrolled 7,875 women, and 3,601 had outcomes. The proportion of preterm births in the intervention and comparison block was 11.18 % and 14.99 %, respectively (p =0.001), and the proportion of low-birth-weight babies was 9.23 % and 11.25 %, respectively (p =0.01). The adjusted prevalence ratio showed that the risk of preterm births in the absence of intervention was 1.3 (CI: 1.1–1.6). Preterm birth was a mediator between preconception care and low birth weight |
| **Date of data collection** | August 2018 to October 2020.  27 months Women enrolled July/Aug 2018  Intervention ran from August 2018 to October 2020. | The total study period was from April 2018 (kick-off meeting) to July 2021 (report submission). The women in the intervention and comparison arms were enrolled from July to August 2018, and the intervention was provided from August 2018 to October 2020 (27 months). |
| **Study objectives** | Goal: to assess the efect of interventions to reduce procnception risk factors among women desirig pregnancy and prevent adverse pregnancy outcomes in women and their newborn babies in selected blocks of Nashik district, Maharastra. Primary objective: to measure the change in pregnancy outcomes (LBW, premature delivery, abortion, stillbirth, early neonatal death). Secondary objectives: - to measure the change risk factors (Low BMI & anaemia) among women planning pregnancy - to measure the association of pregnancy outcomes with risk factors (addiction, consanguineous marriages, age at marriage, low BMI, anaemia) | The specific objectiveswere as follows; To compare the effect of preconception care on premature delivery, low birth weight, congenital physical defects, and neonatal mortality in the intervention and comparison arms. To compare the prevalence of pregnancy loss in the form of abortion and stillbirth in two arms. |
| **Sample size / power** | Used district-level household survey outcomes in total and rural (stillbirths 1.2%, 1.3%, induced abortion 2%, 2.4%, miscarriage 2.5%, 3.7%, LBW 14%, 13.9%. Hypothesised that micarriage and stillbirth may not be amenable for correction, so undertook power calculation for LBW. Estimated 1374 outcomes in each arm needed to detect 25% reduction in LBW with 80% power and 95% confidence. NOTE this was not adjusted for clustering by block. no intracluster corelation coefficient or baseline, endline LBW rate mentioned. This does not look like a correct power calculation. | Authors considered the prevalence of LBW in the rural Nashik district as 14 % (International Institute for Population sciences Ministry of Health and Family Welfare Government of India, 2020a) to estimate the sample size. Hypothesizing a 25 % reduction in the prevalence of low birth weight with 80 % power and 95 % confidence, the estimated sample size was 1,374 pregnancy outcomes in each arm. The probability of achieving the desired sample size was confirmed through precedent- year data from the health management information system. |
| **Sample characteristics** |  | Out of the total women, 37.8 % belonged to tribal blocks. The age of women ranged from 14.42 to 45.50 years (mean 23.19 ±3.71), and the majority (88.6 %) were Hindus. Non-nuclear families were more common (74.3 %) than nuclear ones. The height and weight of 7,167 women (91.0 %) were measured, and the mean baseline BMI was 19.73 (±3.51) kg/m2. Almost one-third of women were undernourished (BMI <18.5) at baseline. |
| **Randomisation** | Purposively selected one tribal and one non-tribal block per arm (2 intervention, 2 control blocks). NO RANDOM ALLOCATION OF CLUSTERS. | We randomly selected one tribal (Peint) and one non-tribal (Sinnar) block for proposed intervention, and one adjacent tribal (Trimbakeshwar) plus one adjacent non-tribal (Niphad) block was purposively assigned for comparison |
| **Blinding** | Not masked. No mention of blinding at the analytical stage is mentioned |  |
| **Age of participants (years)** | 15-45 years (actual age of enrolled women 14.42 to 45.5 years; mean 23.2 +/- 3.7) | women aged 15 to 49 years |
| **Inclusion criteria** | Reproductive age women 15-45 years aspiring to have a healthy intelligent newborn. Resident of the area, Women in reproductive age group 15-45 years and willing to conceive. | All women aged 15 to 49 years in the selected blocks desiring pregnancy within one year and residents of the area were eligible and enrolled by Accredited Social Health Activists (ASHAs). |
| **Exclusion criteria** | Women in reproductive age group and pregnant at the time of enrolment, using contraceptive methods and not willing to conceive, unable to understand Marathi or Hindi or English language, unable to respond due to psychotic illness. | We excluded the women unable to understand Marathi, Hindi, or English and unable to respond due to psychiatric illness |
| **Preconception intervention characteristics** | Investigation and treatment of diagnosed condition, Behaviour Change Communication (BCC) in the form of flip chart, audio-visual recording and audio mobile messages delivered mostly at VHSNDs and partially via home visits. Focus areas for Promoting Preconception Care (PCC) interventions: 1. Achieving normal BMI before conception 2. Preventing / treating anaemia with IFA & deworming 3. Periconceptual folic acid to reduce neural tube defects 4. Quitting tobacco & alcohol toreduce LBW 5. Preventing pregnancy in adolescent girls to avoid teenage pregnancy hazards and to have optimal inter-pregnancy interval following miscarriage or childbirth 6. Detecting / treating RTI/STIs before conception 7. Detecting /managing chronic diseases before conception Interention components: clinical examinaton; lab investigations; treatment, counselling and referral as needed; 6-monthly deworming with albendazole; prophyllactic IFA to all (if Hb <12g/dL- gave 100mg iron + 0.5mg folic acid daily; if Hb >=12g/dL - gave same dose weekly); folic acid supplementationfor prevention of neural tube (what dose to whom when); family planning. All aspects supported by BCC via various channels: manual for ANCs, flip chart, tailored diet plan counselling by BMI, film, group activities at VHSNDs, mobile phone messages (12 voice, 12 SMS). To enrol in the trial ASHAs collected data from eligible women on height, weight at home, Anganwadi Centre or nearest subcentre (SC) or PHC (heath facility). Camps were organised at village/ SC level in intervention blocks at 1st followup visit for blood samples testing: Hb, ABO grouping, Rh typing, blood sugar level estmation, oral glucose tolerance test (OGTT) in women with high random blood sugar levels, Thyroid stiumulating hormone (TSH), HIV, and VDRL (syphilis) tests. ASHAs visited women in househ-to-house visits for BCC and to mobilise attendance of VHSNDs for monthly followup. At VHSNDs ANMs check weight monthly, Hb every 3 months and provide IFA or FA as per guidelines.  ANM supported by ASHAs and monitored by medical officer (MO)) and LHV, delivered BCC at VHSNDs using flipbook and health care manual. Showed documentary film. Group Activities. ALso had mobile phone messages - 12 recorded voice messages and 12 text messages. 2 per week over 6 weeks to registered mobile nos of women. Tailored counselling on the basis of specific diet charts for women with low or high BMI to bring BMI in normal range. | Preconception care included the following components:  1. Clinical examination, including height, weight, and blood pressure, 2. Laboratory investigations included checking blood group, random blood sugar, Thyroid Stimulating Hormone (TSH), Human Immunodeficiency Virus (HIV), Venereal Disease Research Laboratory (VDRL) at enrollment, hemoglobin every three months, and urine pregnancy test after the missing period, 3. Treatment, counseling, and referral as needed for a diagnosed condition, 4. Six monthly deworming using a single dose of 400 mg albendazole, 5. Anemia treatment and prevention; for anemic women, two tablets daily, each containing 100 mg elemental iron and 500 μg folic acid (IFA); and for non-anemic women, weekly same iron and folic acid tablets supplementation, 6. Daily folic acid supplementation during the pre-pregnancy period to anemic women as a constituent of IFA and to non-anemic women (receiving weekly iron supplementation) daily plain folic acid tablet (5 mg or 500 μg depending upon availability), 7. Family planning services; temporary methods for women in the adolescent age group, those having severe anemia, short inter- pregnancy interval, and/or low Body Mass Index (BMI), 8. Behavioral Change and Communication (BCC) included information about quitting alcohol and tobacco, safe abortion centers, BMI normalization in addition to all the above points. The authors trained healthcare workers from the intervention blocks to provide preconception care, imparting BCC and monthly follow-ups of the enrolled women during Village Health Sanitation Nutrition Days (VHSNDs). Initially, all the enrolled women desiring pregnancy were examined by the Medical Officers of the PHCs. The laboratory investigations mentioned above were done at baseline. The women identified as having an illness like anemia, hypothyroidism, high blood sugar, or systemic illness were treated accordingly or referred to a higher center for further management. Women with low BMI were provided BCC with special emphasis on improving nutrition, and those with high BMI were guided to lower it. The ASHA closely followed up with anemic women for regular medication consumption and improvement in hemoglobin levels. ASHAs and Auxiliary Nurse Midwives (ANMs) executed BCC to the women at existing government health facilities. The BCC used three different modes of communication and covered all the points regarding preconception care awareness, education, and counseling. The first two modes of BCC were imparted on VHSND to women groups. The first mode included BCC using a flipbook and was an interactive session. The second mode was a specially produced documentary film of about 28 min shown on a laptop. In the third mode, the women received 12 audio and text messages of about one-minute each (three messages per week) on their mobile phone. Within six months of enrollment, each and every woman from all villages received the BCC intervention. |
| **Preconception control group intervention** | None described |  |
| **Intervention timing (how far before conception)** | Not described in detail? IFA treatment was on average for 5.4 +/- 4.21 months and FA treatment for 6.77+/- 6.89 months. It is not explained if all these are preconceptual women but one assumes so. | Not described. |
| **Intervention delivery (health systems, food systems, social protection systems)** | Health system |  |
| **Pregnancy detection** | Not described but presume that this was checked at VHSNDs by ANMs and/or home visits by ASHAs. |  |
| **Pregnancy intervention characteristics (if any)** | Routine ANC in both intervention and control arms. |  |
| **Pregnancy control group intervention (if any)** | None. Routine ANC in both arms. |  |
| **Interventions after birth (if any)** | None |  |
| **Control group intervention after birth (if any)** | None | None |
| **Compliance measurement** | Not described as such but coverage given in results as follows: Mean no of follow-up visits was 9.47 +/- 6.89 overall. Amongst women who became pregnanct it was 7.18 +/- 5.33 and non-pregnant women 12.17 +/- 7.52. characteristics of women who received no followup visits given. These women will be missing BMI and Hb which were measured as part of the intervention. IFA treatment was on average for 5.4 +/- 4.21 months and FA treatment for 6.77+/- 6.89 months. It is not explained if all these are preconceptual women but one assumes so. |  |
| **Adverse events** | Not described. Trial governance through advisory committee meetings but mention of DMC or adverse events is missing. |  |
| **Engagement, category (including nutrition sensitive/specific)** | Nutrition specific |  |
| **Where in the overall UNICEF 2020 framework the intervention lies** | Investigation and treatment of diagnosed condition affects underlying determinant "services" and immediate determinant "care" to influence health.  Behaviour Change Communication (BCC) in the form of flip chart, audio-visual recording and audio mobile messages and BMI montioring and tailored diet plans based on BMI affect underlying determinant "practices" and immediate determinant "diet".  Provision of IFA and FA affects immediate determinant "diet".  Counselling and group approaches to encourage quitting tobacco & alcohol, preventing pregnancy in adolescent girls and increasing inter-pregnancy interval following miscarriage or childbirth affect enabling determinant "norms".  Detecting / treating RTI/STIs & chronic diseases before conception affect underlying determinant "services" and immediate determinant "care". | Investigation and treatment of diagnosed condition affects underlying determinant "services" and immediate determinant "care" to influence health.  Behaviour Change Communication (BCC) in the form of flip chart, audio-visual recording and audio mobile messages and BMI montioring and tailored diet plans based on BMI affect underlying determinant "practices" and immediate determinant "diet".  Provision of IFA and FA affects immediate determinant "diet".  Counselling and group approaches to encourage quitting tobacco & alcohol, preventing pregnancy in adolescent girls and increasing inter-pregnancy interval following miscarriage or childbirth affect enabling determinant "norms".  Detecting / treating RTI/STIs & chronic diseases before conception affect underlying determinant "services" and immediate determinant "care". |
| **Where in the Partap 2021 framework the intervention lies** | The BCC intervention working through multiple channels (home visits, audio messages, SMS messages) mostly on the individual level by influencing health and nutrition preconception pathways to at risk pregnancies. Access to IFA or FA supplements is affecting individual nutritional intake. Group BCC approaches at VHSNDs are affecting hosuehold and community-level factors as well as individual factors in health, nutrition and social domains. | The BCC intervention working through multiple channels (home visits, audio messages, SMS messages) mostly on the individual level by influencing health and nutrition preconception pathways to at risk pregnancies. Access to IFA or FA supplements is affecting individual nutritional intake. Group BCC approaches at VHSNDs are affecting hosuehold and community-level factors as well as individual factors in health, nutrition and social domains. |
| **Proposed mechanism of intervention action** |  |  |
| **Exposures relating to preconception nutrition** |  |  |
| **Preconception measures** | Secondary risk factor outcomes in women planning pregnancy: - Low BMI & anaemia Risk factors in women (planning pregnancy?): addiction,  consanguineous marriages (marriage between two family members who are 2nd cousins or closer),  age at marriage (to measure in order to test association with birth outcomes) |  |
| **Concommittant care** | Not mentioned |  |
| **Follow-up schedule for measurements** | Baseline household cross-sectional survey May to July 2018 with recall of the preceding year. Plan to repeat this in 2020 but this could not be done because of the covid pandemic. Both baseline and trial enrolment data were collected by ASHAs, supervised by ANM, BF and Lady Health Visitor (LHV).  To enrol in the trial ASHAs collected data from eligible women on height, weight at home, Anganwadi Centre or nearest subcentre (SC) or PHC (heath facility). Camps were organised at village/ SC level in intervention blocks at 1st followup visit for blood samples testing: Hb, ABO grouping, Rh typing, blood sugar level estmation, oral glucose tolerance test (OGTT) in women with high random blood sugar levels, Thyroid stiumulating hormone (TSH), HIV, and VDRL (syphilis) tests. Followup info on enrolled women was collected by ANMs in registers. Block facilitator entered into excel sheets monthly. After pregnancy a woman's data was entered into ANC register and routine care offered. Woman contacted by phone after EDD to collect birth outcome. the outcomes are based upon mother's report on the phone?  ANMs recorded data on a tablet in an application in Marathi which provided summary and upcoming visits. Used for supervision and for generating preliminary analysis. In control areas a similar process was used for collecting outcomes after birth but there was no application available - only registers and excel sheets. There is no mention of how birth weight was measured or standardised between facilities or how birthweight of babies born to women who delivered at home was obtained. Enrolment & baseline studies have checks on data reporting/ quality, but this not explained for ANM registers. it says monitoring sheets were screened but not sure what these were? |  |
| **Summary of baseline comparisons by study arm** | According to the cross-sectional baseline survey intervention and comparison arms differed in education (small differences in many groups) and occupation (intervention had more working women 68.9% vs 53.5). Also intervention had slightly slower % in nuclear family (18.9 vs 20.8%). Baseline survey differenes in abortion (intervention 2.9% vs control 5.3%) and in stillbirths (1.3 vs 1.9%) but not in other birth outcomes according to chisquare test. Baseline risk factor differences in consanguity (numbers incorrect as shown as 17.4% in both arms but significantly different), heavy work in last 6 months (intervention 16.2% vs control 21.3%) a, tobacco use (4.9% vs 6.2%), pesticide exposure (1.6% vs control 2.9%), but no differences between arms in alcohol, domestic voiolence or existing illness.  Enrolment questionnaires showed significant differences in occupation (56.9% farmer vs 40.6%), 28.7% unemployed vs 38.7%, caste (41.8% vs control 25.5% tribal, 13.9% vs 18.7% scheduled caste), and joint/nuclear family. ALso showed differences in risk factors between arms in consanguity (17.2 vs 20.4%), low calorie intake (1.1 vs 1.7%), low protein intake (0.9% vs 1.7%), BMI<18.5kg/m2 (37.9 vs 32.6%, BMI>25 (4.7 vs 6.3%), but no signficiant difference in age <19y, age >=35y, illiterate, multiparity >=4, tobacco, alcohol, previous adverse birt outcome Baseline cross-sectional survey and enrolment differences reflect **systematic differences between intervention and comparison arms which is a major concern**. Authors reflect that differences are only because of sample size but major differences in caste and occupation, and smaller differences in protein intake, low and high BMI mean that only analyses adjusted for these factors should be taken as the primary results. Other differences were small but factors like pesticide exposure, tobacco use and work, could affect brith weight and other birth outcomes and are of concern | Out of the total women, 37.8 % belonged to tribal blocks. The age of women ranged from 14.42 to 45.50 years (mean 23.19 ±3.71), and the majority (88.6 %) were Hindus. Non-nuclear families were more common (74.3 %) than nuclear ones. The height and weight of 7,167 women (91.0 %) were measured, and the mean baseline BMI was 19.73 (±3.51) kg/m2. Almost one-third of women were undernourished (BMI<18.5) at baseline. In the intervention area, about 182 enrolled women (5.09 %) did not attend any follow-up and availed the preconception care services. Overall, the mean number of follow-up visits was 9.47 ±6.89. The women who did not become pregnant during the study period attended more follow-up visits than those who became pregnant.  The data showed statistically significant differences for risk factors like caste, education, occupation, consanguineous marriages, and protein intake among women with pregnancy outcomes between the ntervention and comparison arm (p < 0.05). More women with pregnancy outcomes belonged to a socially disadvantaged group (scheduled tribe) in the intervention area, whereas women with higher education, formal employment, and consanguineous marriages who reported pregnancy outcomes were more in the comparison arm. Overall, alcohol addiction was 0.62 % only; hence, it was not considered for analysis. |
| **Pregnancy outcomes: gestational weight gain, gestational diabetes, maternal anthropometry (weight, MUAC, height), anaemia,** | In intervention arm but not in control: weight each month Hb every 3 months No pregnancy data for control group? |  |
| **Preconceptual outcomes: (BMI, Hb or other outcomes which could be attributed to preconception care)** | BMI changes over time measured in enrolled women only (not in comparison arm) as weight & Hb monitoring was part of the intervention package. |  |
| **Birth outcomes in child: birthweight, low birth weight (LBW), small for gestational age (SGA), preterm birth/ delivery (PTB), cord blood b12, DXA,** | Low birth weight - based on hospital records? - how was this recorded? Secondary outcomes: Premature births, Early neonatal death, Still births, Abortions, (Congenital anomalies?) | The primary outcomes were live birth and fetal loss as abortion (spontaneous or induced before 20 weeks of gestation) or stillbirth (termination of pregnancy after 20 weeks up to 37 weeks). The secondary outcomes among live births were LBW (birth weight < 2500 gm.), premature delivery, congenital physical anomalies, and neonatal mortality. |
| **Outcomes in mother at birth** | None |  |
| **Post-natal outcomes (in Infancy)** | None |  |
| **Outcomes in childhood after 12 m: z scores for weight (WAZ), length/height (HAZ), and BMI for age (BMIZ), weight-for-height'length (WHZ), underweight, stunting, wasting, overweight, cognitive** | None |  |
| **Long term outcomes** | None |  |
| **Analysis** | Data collected on forms and entered were coded, anonymised and collated. Baseline and Enrolment data presented as absolute numbers, proportion and chi-squared tests applied to find an association between variables but it does not say what comparisons were to be made (but tables show intervention and comparison arm characteristics). On baseline data authors used univariate and multivariate analysis to find adjusted prevalence ratio for adverse pregnancy outcomes (but which variables and how these were chosen is not mentioned). Significance was taken at p<0.05. Change in BMI and in Hb was calculated as difference between baseline and pre-pregnancy or last available measure and tested for linear trend.  Compared pregnancy outcome in intervention blocks vs comparison blocks.  Also compared pregnancy outcome at the end of the study (over what period???) with baseline data preceding 1 year before the start of the study.   [Trial registration mentioned: Compare pregnancy outcome in tribal and non-tribal blocks] | We calculated the difference between prevalence of pregnancy outcomes in both arms using the Z test. A major contribution to LBW is from preterm babies. Hence, mediation analysis was done using the Sobel test (Fairchild and MacKinnon, 2009). Initially, bivariate analysis was done to find the crude Prevalence Ratio (PR) for potential risk factors for adverse pregnancy outcomes. The risk factors identified as statistically significant were considered for calculating the adjusted prevalence ratio (APR). The APR at a 95 % Confidence Interval (CI) was calculated using a generalized linear regression model with a log link function and family of binomial distribution. The authors used Statistical Package for the Social Sciences (SPSS; version 25.0) and STATA (15.1 version) for analysis. |
| **EFFECTS on Preconception outcomes: maternal anthropometry (weight, MUAC, height. BMI), Haemoglobin (Hb), anaemia, iodine status** | Thes figures are given for "enrolled women" but it is not explained if they are preconceptual or if any of them became pregnant. If pregnant women were included this would cause a lot of problems with interpretation. BMI Increased from 19.45 +/- 3.03 to 20.79 +/-2.36 % underweight BMI <18.5: 40.7 to 15.35%. P<0.001 for trend using chi squared (not adjusted for other predictors) % normal BMI 18.5-24.9: increased 54.16 to 80.6% % overweight BMI >=25: decreased data not given HB change from baseline to quarter 7 visit: 10.56+/- 1.25 g/dL to 10.1 +/- 1.07g/dL (reduced again to 10.28 +/-1.35 at quarter 8. Anaemia <11or <12?g/dL 71.96 to 57.37% to quarter 6 but increased to 62.76 and 78.94 in quarters 7 and 8 during covid. Is this correct cut-off? was this is preconceptual women not including pregnant women? % of non-anaemic women increased significantly as per chi squared p<0.001 - associated with IFA treatment in the intervention. |  |
| **EFFECTS on Pregnancy outcomes: gestational weigt gain, gestational diabetes, maternal anthropometry (weight, MUAC, height), anaemia, iodine status** | Not clear if the measuresof BMI and Hb reported included outcomes in pregnancy but assume not. |  |
| **EFFECTS on Birth outcomes in child: birthweight, low birth weight (LBW), small for gestational age (SGA), preterm birth/ delivery (PTB)** | of 7875 women enrolled 1721 intervention and 1872 comparison group became pregnant and had outcomes. Comparisons made between Intervention and comparison % unadjusted using chisuqared not adjusting for cofounders despite baseline difference between arms. Indicator, intervention %, comparison %, p value of chi-sq Low Birth Weight: 9.23. 11.25 p=0.057 Congenital anomaly: 1.62 0.84 p=0.036 favours intervention Early neonatal death: 0.19 0.50 p=0.123 Preterm: 11.18 14.99 p<0.001 favours intervention All miscarriages / abortions: 5.52 3.69 p=0.009* Abortion: 22.11 57.97  Miscarriage: 77.89 42.03 p=0.001 Stillbirth: 1.74. 0.64 p=0.002* favours comparison Interpretation of these findings is difficult as they are not adjusted for comfounding differences between arms within arm changes from baseline to study end: Indicator, baseline %, study end %, % change p value of chi-sq Intervention arm only vs control arm only Low Birth Weight: 12.92 to 9.23 -28.56% p=0.0001; 13.44 to 11.25% -16.29% p=0.02 fell in both int and comparison Congenital anomaly: 25.2 to 1.62 -35.71% p=0.038 ; 3.02 to 0.84 -72.19% p<0.001 fell more in comparison group than intervention Early neonatal death: 0.19 0.50 -60.41% p=0.115; 0.70 to 0.50 -28.57% p=0.372 no significant change Preterm: 4.55 to 11.18 +145.71% p<0.001; 3.69 to 14.99 +306.21 p<0.001 fell more in comparison than intervention Changes occurred in both intervention and control. Authors could have done a difference in differences analysis. suggests that endpoint comparison of intervention and control is not helpful without adjustment for confounders and baseline levels. | A total of 3,593 pregnant women reported 3,601 pregnancy outcomes during the study period. The number of pregnancy outcomes was higher than that of pregnant women due to twin pregnancies or women conceiving twice during the study period. The pregnancy outcome number was 1,726 in the study area and 1,875 in the comparison area. Among the enrolled women, only 0.38 % was home deliveries during the follow-up period. The overall adverse live birth outcomes (preterm, low birth weight, neonatal death) were lower in the intervention area. However, fetal loss (abortion of any type and stillbirth) and congenital physical anomalies were higher in the intervention area. Similar results were observed among women who conceived after receiving PCC including 3 modes of BCC (Table 2). Mediation analysis using the Sobel test denotes that preterm birth is a mediator between preconception care and low birth weight outcome (Fig. 3). Bivariate analysis (Table 3) showed a significant prevalence ratio of risk factors associated with adverse pregnancy outcomes. (The numbers in different cells are available as a supplementary file.) Apart from intervention, age, family type, occupation, cast, and BMI showed some association with specific adverse outcomes. Adolescents from socially disadvantaged groups and low BMI had a significant prevalence ratio for low birth weight. The absence of preconception intervention had a significant prevalence ratio for preterm birth. Women in socially disadvantaged groups had a significant prevalence ratio for physical congenital anomalies. The prevalence ratio for abortion was significantly higher among women living in nuclear families, and stillbirth was higher among women from the socially disadvantaged group. A similar state was noted among women who became pregnant after receipt of the BCC intervention package. An adjusted prevalence ratio was calculated to nullify the effect of confounding on the effect of risk factors identified by bivariate analysis. The adjusted prevalence ratio for LBW was significantly higher among women from the socially disadvantaged group, low BMI, and obese. At the same time, preterm birth was significantly higher among women who did not receive preconception intervention. |
| **EFFECTS on Outcomes in mother at birth** |  |  |
| **EFFECTS on Post-natal outcomes (in Infancy)** | None |  |
| **EFFECTS on Outcomes in childhood after 12 m: z scores for weight (WAZ), length/height (HAZ), and BMI for age (BMIZ), weight-for-height'length (WHZ), underweight, stunting, wasting, overweight, cognitive** | None |  |
| **Potential Biological mediating factors (as identified by authors of the primary studies)** | None mentioned | Mediation analysis using the Sobel test denotes that preterm birth is a mediator between preconception care and low birth weight outcome |
| **Potential Behavioural mediating factors (as identified by authors of the primary studies)** | None given |  |
| **Potential Social mediating factors (as identified by authors of the primary studies)** | None given |  |
| **Details on who interventions are available to, and who is accessing services and in what numbers** | None given |  |
| **Author reflections on factors affecting the success of preconception nutrition interventions, including factors relating to leadership, financing, governance, supplies, and capacity** | Not mentioned |  |
| **Author reflections on factors affecting the failure of preconception nutrition interventions, including factors relating to leadership, financing, governance, supplies, and capacity** | The authors see this as a huge success so there is no mention of failure. |  |
| **Author recommendations on strategies to improve preconception nutrition in South Asia** | 1. Scale-up this intervention to the entire state of Maharatsra and beyond. Replicate this intervention integrating preconception care (PCC) into health systems. Add PCC to medical / nursing & secondary school curricula.  Expand from first 1000 days to first 1200 days to include 200 days before conception. 2. Integrate monitoring of weight and BMI of all children, adolescents and women of reproductive age in existing programmes and private sector. (Rashtriya Kishore Swasthya Karyakram, Rashtriya Bal Swasthya Karyakram, School Health and wellness ambassador's initiative). Also provide food supplements to those with low BMI. 3. Expand consumption of multiple micronutrients by increasing consumption of green leafy veg and promote kitchen garden. 4. Promote preconcpetion health among men - ensure every pregnancy is wanted and panned. involve men in ensuring healthy pregnancy by promoting women's reproductive health, prevent gender bias and discrimination, prepare men for fatherhood. (Rashtriya Kishore Swasthya Karyakram, School Health and wellness ambassador's initiative). 5. Strengthen primary health care and design social protection scheme for women in need of preconception improvement (e.g. subsidized food grains for newly weds). | Preconception care addresses modifiable risk factors, particularly improving nutrition and optimizing women’s health. It decreases the risk of preterm birth and low birth weight. The interventions can be carried out with the existing health system with minimal additional resources. Hence, the government sector may introduce preconception care in addition to routine antenatal care |
| **Author recommendations on future research to improve preconception nutrition in South Asia** | Study the impact of reduction of fertility rates on maternal and under 5 mortality. Study malnutrition and early child development of woen receiving preconception care. Undertake cost-effectiveness studies on costs and savings |  |
| **Barriers to Preconception nutritional intake** | Used FGDs with women and health care professionals (HCPs) . Women mentioned hot and cold foods and 'forbidden foods' . Lack of knowledge of adverse impacts of alcohol and tobacco. Women had awareness of importance of conceiving after age 20 and that 'suboptimal' maternal weight can affect newborn health. Community barriers to preconception care identified by HCPs: lack of knowledge, curtailed decision making for women, gender bias, influence of elderly women in the hosuehold, myths and taboos. At institutional level lack of human resources or specialised services, heavy workload, disscrepancies in payments and lack of guidelines. None of these points were elaborated upon so interpretation is limited. |  |
| **Enablers to preconception nutritional intake** | Enablers to preconception nutritional intake not rspecifically mentioned. Factors promoting preconcpetion care identfied by health workers were community counseling for demand generation, women's empowerment, social mobilisation and HCP capacity building. Participant women said they increased from 2 to 3 meals er day. Moitorig BMI at VHSNDs and giving BCC. |  |
| **Risk of Bias assessment ROB2 completed?** |  |  |
| **ABSTRACT** | Summary taken fom the trial registry as there is no short abstract in the report: The study is a **non-randomized intervention** to measure efficacy of preconception interventions in reducing the adverse pregnancy outcomes in four blocks of Nashik district, Maharashtra. Tribal block Peint and non-tribal block Sinnar are intervention blocks while tribal block Trimbak and non-tribal block Niphad are control blocks. In all 7875 women of 15- 45 years age residing in these areas and aspiring to conceive were recruited. The recruitment started from 2nd July 2018 and was completed by the end of July 2018. A package of following seven components inclusive of clinical services and treatment of diagnosed conditions, deworming, folic acid supplementation and specific health education for diet, tobacco and alcohol cessation is being offered to enrolled women in intervention block. This intervention package started from the month of August 2018. These women in intervention blocks are being followed every month. Women in comparison blocks are given regular health care services and followed every six months. The primary outcome measures will be low birth weight and secondary outcomes will be premature births, early neonatal death, still births, abortions. The data on pregnancy outcome is being collected in all blocks. Also, the pregnancy outcomes will be compared among tribal and non-tribal blocks. The same intervention will be given in the control blocks at the end of the study. | Introduction: The World Health Organization has suggested preconception care to improve pregnancy outcomes. Hence, the study aimed to compare the effect of preconception care on pregnancy outcomes, particularly prematurity and low birth weight. Methods: We carried out interventions in one tribal and one non-tribal block. For comparison, one adjacent tribal block and one non-tribal block were included in Nashik district, India. The total study period was from April 2018 to July 2021. All reproductive age group women desiring pregnancy within one year in selected four blocks participated in the study. The services included clinical examination, laboratory investigations, treatment, six- monthly deworming, anemia management, folic acid supplementation, family planning services, and behavioral change communication using different media. The existing healthcare workers provided services to the women until they became pregnant or until the end of the follow-up period (27 months). We monitored pregnancy outcomes, including abortion, stillbirth, and live birth; among live births, low birth weight, preterm birth, congenital physical anomaly, and neonatal death. Results: The study enrolled 7,875 women, and 3,601 had outcomes. The proportion of preterm births in the intervention and comparison block was 11.18 % and 14.99 %, respectively (p =0.001), and the proportion of low-birth-weight babies was 9.23 % and 11.25 %, respectively (p =0.01). The adjusted prevalence ratio showed that the risk of preterm births in the absence of intervention was 1.3 (CI: 1.1–1.6). Preterm birth was a mediator between preconception care and low birth weight. Conclusion: Reduction in proportion of low birth weight and preterm babies can be achieved through preconception care using minimal additional resources. |

# 9. **Supplementary File S7. Intervention characteristics, compliance and mechanisms of preconception interventions**

| **Study ID & Main intervention characteristics** | **Delivered by whom and through what system** | **Intensity of intervention** | **Behaviour Change Communication (BCC)** | **Intervention changing which determinant in UNICEF 2021 framework & place in Partap 2022’s framework^#^** | **Compliance with the intervention** | **Mechanisms / mediating factors discussed by authors** |
| --- | --- | --- | --- | --- | --- | --- |
| ICMR 2000(ICMR, 2000)  Micronutrient capsule:  Multivitamin  Control: Placebo  2 arms | Own delivery system. | Women provided with a three-months supply of supplement every three-month follow up visit at the centre.  Participant consumed the multivitamin on daily basis.  Consumption not observed. | Not mentioned. | **UNICEF 2021 framework:**  Multivitamin supplement directly alters the immediate determinant **“Diet”**.  **Partap 2021 framework:**  individual-level nutrition intervention. | Not reported | Not discussed. |
| Katz 2000 (Katz et al., 2000)  Micronutrient capsule:  Vitamin A / Beta carotene  Control: placebo  3 arms | Own delivery system: local women visited participant homes to distribute the supplement, allowing ³4 days between each consecutive dose. | Supplement delivered weekly.  Consumption observed. | Home visits to observe consumption. | **UNICEF 2021 framework:**  Low dose Vitamin A supplement directly alter the immediate determinant **“Diet”**.  **Partap 2021 framework:**  individual-level nutrition intervention. | Half of all women receiving ≥80% of all possible doses, <75% of all women ≥50% (mean ± SD proportion of doses: 70 ± 30%) | Other micronutrient deficiencies and/or protein-energy malnutrition |
| Khambalia 2009 (Khambalia et al., 2009)  Micronutrient sprinkles:  Testing addition of iron to folic acid (iron folic acid [IFA])  Control: Folic acid (FA)  2 arms | Own delivery system.  Interviewers visited participants monthly to replenish Sprinkles sachets, count empty packages, monitor side effects, and encourage consumption. | 30 sachets + 5 extra sachets of supplements delivered monthly.  Participants consumed one full package per day at any mealtime.  Consumption not observed. | All participants were visited at their home and instructed to consume the powdered micronutrient supplement with semi-liquid food. | **UNICEF 2021 framework:**  IFA or FA sprinkles directly alter the immediate determinant **“Diet”**.  **Partap 2021 framework:**  individual-level nutrition intervention. | Mean ± SD compliance: 57.7% +/-26.9%  1% increase in IFA adherence associated with 10 g/L increase in Hb change from baseline (*p* = 0.03) | - Good baseline iron status  - Iron may be 'channelled' to the placenta and foetus  - Low adherence and short period of supplementation.  - Reduced iron absorption due to high amounts of phytate in rice.  - Mixing the powdered micronutrients with liquids may have caused particles to be lost by sticking to the bowl.  - May have been difficult to  adding sprinkles to food in situations where food is shared. |
| D’Souza 2021 (D'Souza et al., 2021)  Pune Rural Intervention in Young Adolescents (PRIYA) trial  Micronutrient capsule:  Vitamin B12 2mg with / without Multiple Micronutrient (MMN) and milk powder.  Control: placebo  3 arms | Own delivery system.  Project field workers visited participants individually to counsel and provide supplements. | Supplements delivered monthly with a buffer of three extra days’ supply.  Consumption not observed. | All participants were visited individually and counselled before being given the supplements. | **UNICEF 2021 framework:**  B12/MMN supplements and milk directly - affects immediate determinant **“Diet”**.  **Partap 2021 framework:**  individual-level nutrition intervention. | Not reported. | Not discussed. |
| Hambidge 2019a (Hambidge, Bann, et al., 2019; Hambidge, Westcott, et al., 2019) (Dhaded et al., 2020; Young et al., 2021)  The Women First Preconception Trial  Food supplement (lipid-based nutrient supplements [LNS] sachets):  LNS+MMN and targeted LNS for body mass index (BMI)<20kg/m^2^  Control: routine care  3 arms  Intervention arms start LNS in preconception (Arm 1) & 2^nd^ trimester of pregnancy (Arm 2). | Own delivery system.  Research assistant visited all participants’ homes to replenish the supply of trial supplements. | LNS delivered every fortnight.  Participant consumed one sachet per day.  Consumption not observed. | LNS was delivered at home of each participant, who were instructed to consume supplement daily until delivery. A rodent and water-resistant plastic container provided to each participant for storage. | **UNICEF 2021 framework:**  LNS supplement - affects immediate determinant **“Diet”**.    **Partap 2021 framework:**  individual-level nutrition intervention. | Compliance:  Arm 1: overall compliance: 87.2% ± 13.2%; 88% during preconception, 87.3% ± 16.1% during the first trimester, and 84.2% ± 17.4% during the second/third trimester  Arm 2: overall compliance 84.3% ± 17.4% for supplement 1 and 88–96% for supplement 2 | Intervention commencing pre-conception vs 12 to 14 week gestation may cause different DNA methylation patterns.  Improvements in the mother’s environmentally induced epigenome may result in phenotypic improvements in pre- and post-natal growth of her offspring  Chronic inflammation and oxidative stress may alter pregnancy outcomes, fetal and postnatal growth potential, nutrient utilization, nutritional status, and immune development and function in mother and child  LNS may beneficially alter the maternal metabolic profile, through anti-inflammatory and antioxidant features |
| Potdar 2014 (Ramesh D Potdar et al., 2014) (Lawande et al., 2018; Sahariah et al., 2016)  MMNP SARAS Trial  Food supplement:  Locally prepared snack containing green leafy vegetables, milk and dried fruit  Control: low micronutrient-density snack  2 arms | Own delivery system: Study areas were based around 61 supplementation centres.  Centre staff recorded women’s menstrual period dates, gave pregnancy tests after 2 missed periods, and referred pregnant women at 9–13 weeks’ gestation for an obstetric assessment, haemoglobin measurement, and ultrasonography to confirm/date the pregnancy. | Participants required to attend supplementation centres daily (except Sundays and public holidays) to receive snacks.  Women were given one snack per day.  Consumption observed at centres. | Women were given one snack per day, and consumption was observed and recorded. | **UNICEF 2021 framework:**  Snacks containing green leafy veg, milk and dried fruit - affect immediate determinant **“Diet”**.  **Partap 2021 framework:**  individual-level nutrition intervention. | Not reported. | Not discussed. |
| **Dhaded 2020** (Dhaded et al., 2020)  Women First |  |  |  |  | Duration of exposure ( mean ± SD):  Arm 1: 72.6 ± 16.8 weeks for supplement 1, 56.2 ± 25.5 weeks for supplement 2  Arm 2: 26.3 ± 2.2 weeks for supplement 1, and 56.2 ± 25.5 for supplement 2  Overall compliance (mean ± SD):  Arm 1: 89.6 ± 9.9%  Arm 2: 87.7 ± 15.7% | Mechanism not mentioned.  Importance of preconception supplementation clearly demonstrated, particularly in resource-poor settings |
| **Young 2021** (Young et al., 2021)  Women First |  |  |  |  | Not reported. | Since maternal iodine status and thyroid function play a crucial role in embryogenesis it is important that maternal iodine status be sufficient very early in gestation. Hence, starting iodine supplementation (in the SQ-LNS) during the pre-conception period may result in improved maternal iodine status (i.e. higher urinary I/Cr at 12- and 34-weeks’ gestation), which would be positively correlated with newborn anthropometry. |
| **Potdar 2014** (Ramesh D Potdar et al., 2014)  MMNP SARAS Trial |  |  |  |  | Fewer women were compliant in the treatment group (daily snack made from green leafy vegetables (GLV), fruit, and milk, Arm 1) than control group (low-micronutrient potato and onion, Arm 2): 45% compared with 57%, respectively. Compliance was similar in the three months before pregnancy (Arm 1: 44%; Arm 2: 53%) and during pregnancy (Arm 1: 38%; Arm 2: 49%). | Levels of micronutrients, fatty acids, milk, energy content, or protein content in treatment snacks.  Supplementing ≥3 months before pregnancy increased birth weight and other “soft tissue” measurements (skinfolds and abdominal, mid‐upper arm and chest circumference) but not “bony measurements” (length and head circumference).  Supplement effect greater in women with higher BMI. Underweight mothers may have inadequate micronutrient or other substrates which prevented nutrient use or partitioning.  Possible factors reducing effect of the intervention:  - pregnancy outcomes might have improved in Arm 2 as well as intervention due to IFA and counselling.  - control women could have increased their GLV intakes.  - pesticides in GLV could have been toxic.  The smaller effect in ITT analysis in more compliant women may have been because these women were hungrier and more malnourished so had smaller infants, but adjustment for confounding does not support this. Alternatively, the compliance measurement period (before/during pregnancy) may not have reflected long term intake of the snacks. |
| **Sahariah 2016** (Sahariah et al., 2016)  MMNP SARAS Trial |  |  |  |  | Not reported. | Understanding of mechanisms limited by lack of data on the micronutrient status of enrolled women.  Snack components that may have contributed to the effect on GDM were:   - Magnesium, fatty acids (long-chain v-3 PUFAs), or nitrates in GLV.   Calcium, vitamin D, or whey protein from milk. |
| **Lawande 2018** (Lawande et al., 2018)  MMNP SARAS Trial |  |  |  |  | <45-47% of the women in the Arm 1 and 58-61% in Arm 2 at each visit | Supplementation may affect processes such as de- and re-methylation of fetal DNA, the development of the placenta and fetal organogenesis in early gestation. These are important determinants of size at birth and long-term health.  Lack of impact of MMNP supplement on fetal size and growth might be because: i) HC, BPD, and FL are measures of bone size (which was not improved); ii) abdominal circumference (a soft tissue measure) is highly variable and can be distorted by the transducer; iii) differences in fetal growth may have occurred during late gestation and so were unmeasured. |
| Taneja 2022 trial paper (Taneja et al., 2022)  The Women and Infants Integrated Interventions for Growth Study (WINGS)  Complex intervention:  i) Health: screening / treatment  ii) Nutrition: screening / treatment & supplementation with locally prepared snacks, egg or milk iii) Psychosocial support.  iv) WASH promotion  Control: routine care  4 arms (factorial design).  Interventions arms vary in timing of the intervention.  Arm 1: Started in preconception & continued to early childhood.  Arm 2: only in preconception.  Arm 3: Started in pregnancy & continued to early childhood.  Arm 4: routine care | Own delivery system: Trial community workers visited women at home at least weekly.  Health check-ups delivered through the health system | Weekly interactions cover health, nutrition, social and water, sanitation and hygiene (WASH) systems.  More frequent interactions for women with moderate to severe anaemia, thyroid disorders, reproductive tract infections, or undernutrition.  **Preconception** period:  MMN supplement three times a week or IFA weekly  Daily snack or egg/ milk (six days a week) for those with BMI <16 or <21, respectively  **Pregnancy**: Weekly supplies of locally prepared snacks were provided for daily consumption (women with BMI <25). Milk provided six days a week to all women and its consumption was observed. | Preconception period:  Counselling on diets, positive thinking, problem-solving, and menstrual and hand hygiene  Trial community workers or ‘Sanginis’ (friends) visited homes to reinforce interventions, replenish supplement supplies, and manage health conditions.  Pregnancy: Women had weight monitored, nutrition counselling, and hot cooked meal provided. Positive thinking and problem-solving skills were promoted. | **UNICEF 2021 framework:**  Screening/treating medical conditions & anaemia- effect of **“services”** and “**care**” underlying / immediate determinant**.**  Providing IFA, MMN, egg or milk (70 kcal, 6 g protein) affect **“food”** and **“diet”** underlying /immediate determinants.  Dietary counselling affects **“practices”** underlying determinant  Positive thinking and problem-solving skills address “**norms”** and **“resources”** (social/human capital) enabling determinants.  WASH supplies affect **“services”**. underlying determinant. Counselling on personal, menstrual and hand hygiene affects **“care”** immediate determinant.  **Partap 2021 framework:**  Spans all four domains of health, nutrition, social and WASH related factors that affect preconception. Works mostly at individual and partially household levels. | Preconception morbidity screening and treatment: 90%  Counselling on positive thinking and problem-solving skills: 97% | The authors hypothesised that a complex, multifactorial problem like stunting could only be addressed by a complex intervention addressing key health, nutrition, psychosocial care, and environment issues at the same time. They believe the larger effects of their intervention are because the intervention covers more aspects of the WHO framework on child stunting, than simple interventions.  Improved fertility in the preconception arm could be due to:  - improvements in anaemia and nutritional status, and reduced risk of reproductive tract infections.  - better mental health (conducive to pregnancy planning) |
| Kumar 2023 (Kumar et al., 2023)  Swabhimaan  Complex intervention:  Mobilisation of federations of Self-Help Groups (SHGs), farmer groups, and adolescent groups using Participatory Learning Action (PLA) and home visits by Poshan Sakhis (Kishori Sakhis and Krishi Mitras) which supports a package of government services available in intervention and control.  Control: service strengthening including weekly IFA.  2 arms | Delivered via government systems.  Community-led components (PLA and home visits) delivered via platforms of SHGs by ‘Poshan Sakhis’ (nutrition friends). ‘Krishi Mitras’ facilitated farmers groups and ‘Kishori Sakhis’ (adolescent friends) targeted adolescents (in Bihar only). Health system intervention components were mostly delivered at VHSNDs. | Multiple intervention components across multiple community and health-system platforms. Control areas also included health system components. | Information sharing through counselling via home visits and VSHNDs  Community mobilisation through PLA meetings with women, adolescents and farmers.  Grant management activities were led by cluster-level federations.  Poshan Sakhis and Kishori Sakhis co-facilitated the development of the integrated nutrition micro-plan with block coordinators.  ‘Krishi Mitras’ facilitated meetings with women farmers on nutrition-sensitive agriculture practices, PLA, and setting up home-based nutrition gardens/backyard poultry. | **UNICEF 2021 framework:**  IFA & calcium supplementation, iodised salt, and BEP supplementation affect **“Diet”** - immediate determinant**.**  Generalised household rations, nutrition-sensitive agriculture and community-based food insecurity coping strategies affect **“Food**” - underlying determinant.  Education on maternal dietary diversity, sanitation and hygiene, reproductive health, institutional deliveries and maternity benefits, risks of tobacco and alcohol use & importance of insecticide-treated bed nets affect **“Practices**”- underlying determinant.  Reproductive, antenatal and postnatal care, institutional deliveries and maternity benefits, family planning, deworming, monitoring of nutritional status and community-based at-nutritional risk package, and early outreach services affect “**care**” immediate determinant. Strengthening of these systems affects “**services**” - underlying determinant.  Safe drinking water and sanitation commodities, insecticide-treated bed nets for malaria prevention and promotion of secondary education affect **“Services”** - underlying determinant  Raising women’s collective voice, decision-making to prevent child marriage, violence against women, improve birth spacing, and other gender-related issues affect **“Norms”** - enabling determinant**.**  **Partap 2021 framework:**  Screening, treatment, home visiting and counselling - individual level health and nutrition impact pathways.  Group BCC – household, community-level & individual factors in health, nutrition and social domains. Hygiene practices - WASH domain. | Trial meetings or community day attendance: 33.4% of mothers of children <2 years, 28% of pregnant women, and 19% adolescent girls attended both in the intervention areas  Community day attendance : 14.7% of adolescent girls in the control areas | Participation was associated with increased consumption of micronutrient supplements in all groups. Employing a cadre specifically trained to working with adolescent girls may have helped improve their nutrition.  Improved coverage and uptake of health and nutrition services may have been attributable to MUAC screening, microplanning and participation in group meetings. |
| Healthy Parents Healthy Child Initiative(Gov. of Maharashtra et al., 2021)  Complex intervention:   - BCC at VHSNDs & home visits. - Counselling to optimise BMI & nutrition sensitive - IFA. - Health screening / treatment.   Control: routine care  2 arms | Delivered via the health system.  Auxiliary nurse Midwives (ANMs) and Accredited Social Health Activist (ASHA) provided health services at VHSNDs and via home visits, respectively.  Medical Officer and Lady Health Visitor monitored the activities.  Government, UNICEF, and university officials provided supervision. | ASHA’s visited homes monthly to promote BCC and encourage VSHND attendance.  Monthly follow up visits at VSHND for VSHND session using BCC tools  12 voice and 12 text messages two times per week over six weeks.  Daily IFA for women with anaemia and weekly IFA for women with no anaemia. | BCC tools used:  - Health care manual  - Oral communication using flipbook  - Audio-video session including audio-visual documentary films. ANM showed short documentary films and group communication activities were carried out.  - Short message service (SMS) and voice recorded messages  - Counselling with specific diet chart was given to women with low BMI or high BMI.  - Home visits to promote BCC and encourage VSHND attendance. | **UNICEF 2021 framework:**  Investigation and treatment of RTI / STIs & chronic diseases affects underlying determinant **“services”** and immediate determinant **“care”**.  Provision of IFA, FA and take-home rations affects immediate determinant **“diet”.**  BCC in the form of flip charts, playing videos, sending SMS and audio mobile messages and tailored diet plans based on BMI affect underlying determinant **“practices”** and immediate determinant **“diet”.**  Counselling and group approaches to encourage quitting tobacco & alcohol, preventing pregnancy in adolescence and increasing inter-pregnancy interval following miscarriage or childbirth affect the enabling determinant **“norms**” and the underlying determinant “**practices**”.  **Partap 2021 framework:**  Counselling at home visits, audio messages, SMS messages and provision of IFA or FA supplements work mostly on the individual level on health and nutrition preconception pathways.  Group BCC approaches at VHSNDs affect household and community-level factors as well as individual factors in health, nutrition and social domains. | # follow-up visits (mean +/- SD):  Overall: 9.47 +/- 6.89  Women who became pregnant: 7.18 +/- 5.33  Non-pregnant women: 12.17 +/- 7.52.  Average duration:  IFA treatment (mean +/- SD): 5.4 months +/- 4.21  FA treatment (mean +/- SD): 6.77+/- 6.89. | Not discussed |

Abbreviations: ANMs, Auxiliary Nurse Midwives; ASHA, Accredited Social Health Activist; BCC, Behaviour Change Communication; BMI, Body Mass Index; FA, Folic acid; IFA, iron folic acid; LNS, lipid-based nutrient supplements, MMN, Multiple Micronutrient; MUAC, mid-upper arm circumference; PLA, Participatory Learning and Action; SHGs, Self-Help Groups; SMS, Short message service; VSHNDs, Village Health Sanitation and Nutrition Days; WASH, water, sanitation and hygiene. ^#^ Partap’s framework is depicted in Figure 2 and in Partap et al 2022(Partap et al., 2022); UNICEF 2021 framework reference(UNICEF, 2021)

# 10. **Supplementary file S8. Narrative summary of Results of individual studies**

**Impacts on blood micronutrient assays during pregnancy and in cord blood**

Vitamin A and beta-carotene supplementation during preconception and early pregnancy improved maternal serum retinol at <4 months gestation and reduced retinol concentrations <0.70 mmol/L from 19.3% in the control arm to 2.9% and 13.5% in the Vitamin A and beta-carotene arms respectively.(Katz et al., 2000) Preconception sprinkles with IFA compared with FA only did not improve anaemia, Hb or plasma ferritin in pregnancy, but earlier initiation and higher adherence to IFA led to greater Hb increments.(Khambalia et al., 2009) Preconception vitamin B12 and B12+MMN resulted in significantly higher B12 (at 28 weeks’ gestation) and holo-TC (at 28 weeks’ gestation and in cord blood at birth), and insignificantly higher BDNF (in cord blood at birth). Preconceptual B12 alone did not improve Hb, folate, Vitamins B2, B6 and B6 pyridoxal 5-phosphate, and homocysteine at 28 weeks’ gestation, but B12+MMN improved folate and B2 (D'Souza et al., 2021).

**Impacts on maternal anthropometry in pregnancy and fetal growth**

In the WINGS study, preconception intervention arms had 0.56 g/dL (98.3% CI: 0.48, 0.64) higher Hb, 1.42 kg (98.3% CI: 1.15, 1.70) higher gestational weight gain, and lower risk of BMI<18.5 kg/m2 (absolute risk reduction [ARR] −1.98%, 98.3% CI: −4.64, 0.68 (CI crossing null effect); Table 3) at 35–37 weeks’ gestation than the control group. Similarly, the preconception + pregnancy arm had 0.77g/dL (98.3% CI: 0.59, 0.95) higher Hb, lower moderate anaemia (ARR −14.9%, 98.3% CI: −19.37, −10.43) and 1.08 kg (98.3% CI: 0.80, 1.36; Table 3) higher GWG (Taneja et al., 2022). It was not reported in the HPHCI intervention write up whether BMI and Hb were during pregnancy (Gov. of Maharashtra et al., 2021). In the Swabhimaan trial, prevalence of thinness (MUAC <23 cm) during pregnancy decreased more in the control 45.1% to 34.5% than in the intervention 41% to 37.1% (difference in difference 6.59, not significant) (Kumar et al., 2023).

Provision of GLV, milk and dried fruit within a local snack in the MMNP did not impact measures of fetal size or growth during pregnancy, and no significant interactions between allocation group and maternal pre‐pregnancy BMI, height, age or fetal sex were found (in ITT or per-protocol analyses). Some interactions between allocation group and maternal parity were observed on fetal growth in ITT analyses in the 2^nd^ trimester. BPD (*p* = 0.02) and FL (*p* = 0.04) were smaller among foetuses of primiparous women and larger among multiparous women in treatment arms compared with control. In the subgroup analyses among women with >=3 months of preconception supplementation, the intervention did not have a significant effect on HC, AC or FL (Lawande et al., 2018).

**Impacts on gestational diabetes mellitus (GDM) in pregnancy**

In the MMNP trial, GLV, milk and dried fruit enrichment of local snacks reduced GDM prevalence using ‘old’ WHO 1999 definitions; 7.3% compared with 12.4% in control in intention-to- treat (OR: 0.56; 95% CI: 0.36, 0.86; *p* = 0.008) and 7.5% compared with 13.1% in control in per-protocol (OR: 0.54; 95% CI: 0.33, 0.86; *p* = 0.01). However, no difference between treatment and control was detected when using the WHO 2013 GDM criteria. Similarly mean fasting or 120-min glucose concentrations nor fasting insulin concentration did not differ by study arm, and no significant interactions were observed between allocation group and maternal age, BMI, height, or parity in relation to any outcome (Sahariah et al., 2016).

**Impacts on birth anthropometry**

*Micronutrient interventions effects on birth size*

Birth weight, length and head circumference did not differ by whether B12, B12+MMN, or multivitamins (added to iron and calcium supplements) were provided in preconception (D'Souza et al., 2021) (ICMR, 2000).

*Food intervention effects on birth size*

Four studies reported the impact of Women First on birth size. The combined analysis across the 4 countries and site-specific analyses (Hambidge, Westcott, et al., 2019) indicated that starting the supplement before conception did not significantly improve newborn LAZ (unadjusted or GAA-adjusted) or other binary GAA-adjusted outcomes compared to supplementation during pregnancy only. However, mean non-GAA adjusted LAZ, was higher for preconception + pregnancy than for control (<0.2 LAZ) for combined sites (*p* < 0.01) and for Pakistan (+0.34 (0.10, 0.58) p = 0.0057; non-significant for India (+0.17, *p* = 0.1244); Table 3). The effect size was positive for GAA-adjusted LAZ for combined sites (+0.20), for India (+0.23 (0.01, 0.45), p=0.0402), and for Pakistan (+0.35 (0.08, 0.62), p=0.0107; Table 3). LBW incidence for combined sites was lower in the preconception + pregnancy arm versus control (RR 0.86; 95% CI: 0.75, 0.98, *p* = 0.0263) but no differences between arms or sites were observed for either HCAZ or BMIAZ. Preconception + pregnancy vs control comparisons showed reductions in relative risks (RRs) for stunting (LAZ <−2), wasting (weight-to-length-ratio-for-age Z-score [WLRAZ] <−2), and SGA for combined sites, India and Pakistan (but not for wasting in Pakistan) (Hambidge, Westcott, et al., 2019)

When limiting analysis to South Asian sites of Women First (Dhaded et al., 2020), intervening in the preconception period combined with pregnancy had significant effects on effect sizes on LAZ (+0.29; 95% CI 0.11–0.46, *p* = 0.001), WAZ (+0.22; 0.07–0.37, *p* = 0.0043), WLRAZ (+0.27; 0.06–0.48, *p* = 0.013), LAZ<-2 (RR: 0.56; 0.38–0.82, *p* = 0.003), WAZ <-2 (RR: 0.68; 0.53–0.88, *p* = 0.0028); WLRAZ <-2 (RR: 0.76; 0.64–0.89, *p* = 0.001), SGA (RR: 0.74; 0.66–0.83, *p* < 0.0001), and LBW (RR: 0.81; 0.66–1.00, *p* = 0.0461; Table 3). In contrast, intervening during pregnancy without any preconception intervention showed relatively marginal effects for LAZ (0.21; 0.04–0.38) and WAZ <-2 (RR: 0.70; 0.53–0.92) and SGA (RR: 0.88; 0.79–0.97) (Dhaded et al., 2020).

Significant (*p* < 0.05) treatment arm x parity interactions for LAZ and WAZ were found, with larger effects for nulliparous women for all outcomes (LAZ (0.56; 0.28, 0.84, *p* < 0.001), WAZ (0.45; 0.20, 0.07, *p* < 0.001), and WLRAZ (0.52; 0.17, 0.88, *p* < 0.01)) when comparing preconception + pregnancy LNS versus control (only significant for birth length in pregnancy-only arm) (Hambidge, Bann, et al., 2019).

The other sub-study of Women First found an association between iodine status at 12-weeks’ gestation (resulting from the LNS supplement iodine content) and birth LAZ (mostly driven by the effect in Guatemala) and HCAZ (driven by India (0.18 per 100 I/Cr, *p* < 0.01; not observed in Pakistan), and mediation analysis suggested that iodine status is a mediator for these outcomes (Young et al., 2021).

ITT analyses of the effect of the enhanced snack with GLV, milk and dried fruit compared with low micronutrient density snack in the MMNP (R. D. Potdar et al., 2014) showed no significant differences between allocation groups in birth weight, LBW, SGA, large- for-gestational-age, chest (CC) or abdomen circumferences (AC), or subscapular or triceps skinfolds. Significant interactions between the allocation group and maternal prepregnant BMI (kg/m^2^) were found for birth weight, length, head circumference, chest circumference, abdomen circumference, MUAC), subscapular and triceps skinfolds whereby, unexpectedly, larger effects were found in women in the higher BMI categories. In per-protocol analyses, including only cases where preconception exposure exceeded 90 days, birth weight was 48 g (95% CI: 1, 96 g; *p* = 0.046) higher in the treatment group, and LBW and SGA were lower (LBW: treatment: 34%; control: 41% (OR: 0.76; 95% CI: 0.59-0.98 (*p* = 0.03)); SGA: treatment: 66%; control: 71% (OR: 0.80; 95% CI: 0.61-1.04 (*p* = 0.09)); not significant for LGA or other newborn measurements). Interestingly, the in per-protocol analyses, the birth weight effect increased with maternal BMI (28, +79, and +113 g in lowest (<18.6), middle (18.6–21.8), and highest (>21.8) thirds of BMI, respectively *p*-interaction = 0.001). In the highest (>21.8) third of maternal BMI, increases were found in birth length (0.3 cm, 95% CI: -0.2, 0.9), CC (0.6 cm, 95% CI: 0.2, 1.1 cm), MUAC (0.2 cm, 95% CI: 0.1, 0.4 cm), and triceps skinfold thickness (0.2 mm, 95% CI: -0.1, 0.5 mm)(R. D. Potdar et al., 2014)

In the WINGS study, intervening during preconception (with or without pregnancy intervention) reduced LBW (IRR 0.85, 98.3% CI: 0.75-0.97; ARR −3.80%, 98.3% CI: −6.99- −0.60), SGA (IRR 0.87, 98.3% CI: 0.78-0.98; ARR −4.04%, 98.3% CI: −7.47- −0.62), and stunting (IRR 0.81, 98.3% CI: 0.69, 0.96; ARR −3.18%, 98.3% CI: −5.91- −0.45) by 15-19% (Taneja et al., 2022). Birth weight was 40.84 g higher (98.3% CI: 7.84, 73.84 g) and birth length was 0.17 cm (98.3% CI: 0.01, 0.32 cm) higher (no difference in head circumference). When comparing preconception + pregnancy with control, intervention effects were similar but larger; LBW (IRR 0.76, 98.3% CI: 0.62, 0.91; ARR −5.59%, 98.3% CI: −10.32, −0.85), SGA (IRR 0.71, 98.3% CI: 0.61 to 0.83; ARR −11.84%, 98.3% CI: −16.94, −6.75) and stunting (IRR 0.68, 98.3% CI 0.53, 0.87; ARR −5.32%, 98.3% CI: −9.42, −1.22) were all reduced. The intervention was associated with increased birth weight by 77.67 g (98.3% CI: 26.38, 128.96), birth length by 0.21 cm (98.3% CI: −0.03, 0.45), and head circumference by 0.18 cm (98.3% CI: 0.02, 0.33) (Taneja et al., 2022).

In the HPHCI, LBW fell from baseline to study end in both arms but more in the intervention arm (Arm 1: 12.92 to 9.23; -28.56%, p = 0.0001 versus Arm 2: 13.44 to 11.25%; -16.29%, p = 0.02; Table 3) but no hypothesis test to compare arms was possible (Gov. of Maharashtra et al., 2021).

**Impacts on Preterm / gestational age at birth**

Preterm birth was not improved by Vitamin A and beta-carotene supplementation during preconception and early pregnancy compared with placebo (RR 1.06 (95% CI: 0.91, 1.25) for Vitamin A and 1.03 (95% CI: 0.87, 1.19) for beta-carotene) (Katz et al., 2000). In Women First, combined sites analysis suggested incidence of preterm was significantly lower in pregnancy-only (7.4%) intervention compared with intervention in pregnancy & preconception (12.5%) or control (11.5%; p = 0.0407) (Hambidge, Westcott, et al., 2019), but this was not significant in India or Pakistan alone (Dhaded et al., 2020). In the WINGS trial, intervening during the preconception period alone (Arm 2) was not associated with lower preterm IRR compared with the control, but intervening during pregnancy only (Arm 3) was (0.69 (0.54 to 0.89); P=0.005) (Taneja et al., 2022). In the MMNP study, preterm and gestational age at birth were similar between groups in ITT and per-protocol analyses (R. D. Potdar et al., 2014). In the HPHCI, although preterm was lower in the intervention (11.18%) compared to the control group (14.99%, p < 0.001), preterm fell more in the control than the intervention arm over time (Gov. of Maharashtra et al., 2021).

# 11. **Supplementary File S9. Mechanisms of action or mediating factors discussed by authors**

Supplementary File S7 above summarises information on mechanisms of action or mediating factors discussed by authors where available. The Women First trial paper explained how changes in fetal DNA methylation (epigenetic changes) brought about by early supplementation in the preconception period may result in positive changes in fetal and postnatal growth. Chronic inflammation and oxidative stress negatively affect fetal and postnatal growth potential, nutrient utilization and nutritional status, and immunologic development and function of mother and of the offspring. Anti-inflammatory and antioxidant features of the LNS initiated preconceptually may improve the maternal metabolic profile which has positive epigenetic effects (Hambidge et al., 2014; Hambidge, Westcott, et al., 2019). Similarly, MMNP authors hypothesised that initiating the improved snack more than 3 months before conception may affect processes of de-methylation and re-methylation of fetal DNA, affecting the development of the placenta and fetal organogenesis, which are important determinants of birth size and long-term health outcomes (Lawande et al., 2018). Young et al. hypothesised that significantly higher urinary I/Cr at 12- and 34-weeks’ gestation would be positively correlated with newborn anthropometry because iodine deficiency may otherwise be limiting fetal growth (Young et al., 2021).

The effect of the WINGS study may be informed by examining the WHO framework on childhood stunting (Stewart et al., 2013). The authors hypothesise that the larger impact of the WINGS intervention compared with studies examining simple interventions is because a complex, multifactorial problem like stunting can only be addressed by a complex intervention addressing key health, nutrition, psychosocial care, and environment issues at the same time (Taneja et al., 2022). Preconception interventions were at least as important as pregnancy interventions for birth outcomes in their study. A higher proportion of live births in the preconception intervention group (i.e. higher fertility) may be associated with improvements in anaemia and nutritional status, reduced the risk of reproductive tract infections. Also, counselling might have improved mental wellbeing, which is conducive to pregnancy planning (Taneja et al., 2022).

In the Swabhimaan trial, higher participation was associated with higher odds of consumption of micronutrients. Employing resource persons specifically trained for working with adolescent girls may have helped to improve their nutrition outcomes. Use of community cadres for MUAC screening, microplanning, and mobilising participation in group meetings resulted in increases demands for and coverage of health and nutrition services which suggests that community mobilisation approaches need more emphasis (Kumar et al., 2023).

*Biological mediating factors in the MMNP*

In the MMNP the food supplement appeared to have larger effects on the newborn size in mothers of higher BMI, which is opposite to the effect seen in trials of protein-energy supplementation in pregnancy, where birth weight increased more in undernourished women. However, meta-analysis of trials of MMN supplementation in pregnancy showed a greater birth-weight effect in mothers of higher BMI in 11 of 12 trials. The metabolism of nutrients, development of the fetal supply line, transportation of nutrients across the placenta, and fetal growth require energy and other substrates. So, underweight mothers may have had inadequate macronutrients or other substrates to use the nutrients supplied by supplements or to partition them to the foetus. Extra macronutrients, in addition to micronutrients, may be needed for underweight women in low- and middle-income countries to channel nutrients provided in preconception to the foetus (R. D. Potdar et al., 2014).

The effect of the preconception and pregnancy snacks might be attributable to more micro-nutrients, fatty acids, higher milk consumption or to higher energy (+0.32 MJ) and/or protein (+4 g) in treatment than control snacks. However, supplementation trials that improved birthweight used 2 to 10 times more energy and protein than that the additional provided by the MMNP snack (R. D. Potdar et al., 2014).

In the sub study of MMNP, limited information on the micronutrient status of women, limits the authors' ability to suggest mechanisms for the reduction in GDM. It appears that supplement had no effect on glucose concentrations in women with normal glucose tolerance or in those with established diabetes in pregnancy, but that there was an intermediate group of women who were vulnerable to diabetes and whose metabolic competence was improved by the supplement. There is a possibility that the control snacks increased the risk of GDM, but since control snacks contained less energy than the intervention snacks, and control group women did not gain more fat than intervention group women this seems unlikely. As baseline intakes were very low, the daily snack substantially increased the intake of GLV, fruit, and milk. Higher intakes of magnesium from GLV could be associated with a lower diabetes risk. Alternatively fatty acids (long-chain v-3 PUFAs) in GLV may improve insulin sensitivity by influencing the properties of cell membranes. Nitrates in GLV may increase thermogenesis, oxygen consumption, and b-oxidation or intakes of calcium, vitamin D, or whey protein in dairy might lower diabetes risk. (Sahariah et al., 2016).

Amongst newborns of mothers supplemented for ≥3 months before pregnancy, the MMNP snacks increased “soft tissue” (birth weight, skinfolds and abdominal, mid‐upper arm and chest circumference) but had no effect on “bony measurements” (length and head circumference). Also, greater effects on soft tissue measurements were found in women with normal or high pre‐pregnancy BMI (R. D. Potdar et al., 2014). However, the Lawande 2018 sub-study found no effect on the MMNP supplement on ultrasound measures of fetal size and growth, and no evidence of an interaction between allocation group and BMI. This might be because i) HC, BPD, and FL are measures of bone size; ii) AC (soft tissue measure) is highly variable and can be distorted by the transducer; iii) differences in fetal growth were unmeasured if they occurred during late gestation (>33 weeks). An interaction between intervention group and parity in relation to fetal BPD and FL at visit 2 (19–21 weeks) was found. This may be a chance finding, combined with the lack of interaction effects at 9-12 weeks or 28-32 weeks, could suggest that fetal growth was slower in early pregnancy in lower parity women in the intervention group, but “caught up” between visits 2 and 3. Or fetal growth could have been faster in early pregnancy among women of higher parity and became slower between visits 2 and 3 (Lawande et al., 2018) .

Potdar 2014 explained several factors which might explain why effects on the MMNP supplements were not greater. Pregnancy outcomes may have improved in both arms since counselling and IFA promotion was in control as well as intervention areas. Control women may have increased their GLV intake though dietary studies (not reported). The intention-to-treat analysis unexpectedly showed a smaller effect on birth weight in fully compliant women. This might reflect confounding whereby the most-deprived women were hungrier and more compliant, but also had smaller infants. However, this explanation seems unlikely since adjustment for confounders did not alter the effect. Contaminants like pesticides in GLV could have been toxic at higher intakes, but fresh leaves were thoroughly washed. The period used to define compliance (immediately before and during pregnancy) may not have been a good measure of long-term compliance with the snacks and those who were in the study longer may have benefitted more. (R. D. Potdar et al., 2014)

# 12. **Supplementary file S10. Visual presentation of risk of bias judgement of randomised controlled trials using the RoB2 tool**

| **Cochrane risk of bias tool for randomized control trials** | | | | | | |
| --- | --- | --- | --- | --- | --- | --- |
| **Study ID** | **Randomisation process** | **Deviations from the intended interventions** | **Missing outcome data** | **Measurement of the outcome** | **Selection of the reported result** | **Overall bias** |
| ICMR 2000 |  |  |  |  |  |  |
| Katz 2000 |  |  |  |  |  |  |
| Khambalia 2009 |  |  |  |  |  |  |
| D’Souza2021 |  |  |  |  |  |  |
| Hambidge 2019a |  |  |  |  |  |  |
| Hambidge 2019b |  |  |  |  |  |  |
| Dhaded 2020 |  |  |  |  |  |  |
| Young 2021 |  |  |  |  |  |  |
| Potdar 2014 |  |  |  |  |  |  |
| Sahariah 2016 |  |  |  |  |  |  |
| Lawande 2018 |  |  |  |  |  |  |
| Taneja 2022 |  |  |  |  |  |  |

Legend:

Low risk

 Some concerns

High Risk

# 13. **Supplementary file S11. Visual presentation of risk of bias judgement of non-randomised trials using the ROBINS-I tool**

| The Risk of Bias in Non-randomized Studies – of Interventions (ROBINS-I) assessment tool | | |
| --- | --- | --- |
| **Study ID** | Doke 2024 | Kumar 2023 |
| **Description** |  |  |
| Bias due to confounding |  |  |
| Bias in selection of participants into the study |  |  |
| Bias in classification of interventions |  |  |
| Bias due to deviations from intended interventions | No information |  |
| Bias due to missing data |  | No information |
| Bias in measurement of outcomes |  |  |
| Bias in selection of the reported result |  |  |
| **Overall bias** |  |  |

Legend:

Low risk

Moderate risk

Serious risk

# 14. **Supplementary file S12. The Risk Of Bias In Non-randomized Studies – of Interventions (ROBINS-I) assessment tool filled for Kumar A, Sethi V, Wagt A, et al.**

Kumar A, Sethi V, Wagt A, et al. Evaluation of impact of engaging federations of women groups to improve women's nutrition interventions- before, during and after pregnancy in social and economically backward geographies: Evidence from three eastern Indian States. *PLoS One* 2023; **18**(10): e0291866.

(version for cohort-type studies)

**Version 19 September 2016**

This work is licensed under a [Creative Commons Attribution-NonCommercial-NoDerivatives 4.0 International License](http://creativecommons.org/licenses/by-nc-nd/4.0/).

**ROBINS-I tool (Stage I): At protocol stage**

1. **Specify the review question**

| Participants |  |
| --- | --- |
| Experimental intervention |  |
| Comparator |  |
| Outcomes |  |

**List the confounding domains relevant to all or most studies**

**List co-interventions that could be different between intervention groups and that could impact on outcomes**

**ROBINS-I tool (Stage II): For each study**

**Specify a target randomized trial specific to the study**

| Design | non-randomised controlled study |
| --- | --- |
| Participants | adolescent girls, newlywed women and couples, pregnant women and mothers of children under 2years of age. |
| Experimental intervention | PLA groups implemented by Poshan Sakhis (nutrition friends) and (in Bihar only) Kishori Sakhis (adolescent friends) and Krishi Mitras (Farmer friends). The PLA groups implemented by Poshan Sakhis were part of the federation of Self Help Groups (SHGs). The federation coordinators worked with Poshan Sakhis to generate nutrition micro-plans which the Poshan Sakhis implemented with PLA groups and via home visits.  Complex intervention combining 14 nutrition-specific and nutrition- sensitive interventions in the domains of   - Access to government rations & IFA. - Uptake of health services - WASH - Dietary change - Nutrition sensitive Social and Behaviour Change.   **-** System strengthening |
| Comparator | Weekly IFA supplementation via National Iron Plus Initiative**.**  System strengthening: |

**Is your aim for this study…?**

| X | to assess the effect of *assignment to* intervention |
| --- | --- |
| □ | to assess the effect of *starting and adhering to* intervention |

Specify the outcome

Specify which outcome is being assessed for risk of bias (typically from among those earmarked for the Summary of Findings table). Specify whether this is a proposed benefit or harm of intervention.

BMI of adolescent girls

MUAC with cut-offs of <23 cm among pregnant women.

**Specify the numerical result being assessed**

In case of multiple alternative analyses being presented, specify the numeric result (e.g. RR = 1.52 (95% CI 0.83 to 2.77) and/or a reference (e.g. to a table, figure or paragraph) that uniquely defines the result being assessed.

% Mid-Upper Arm Circumference (MUAC) with cut-offs of <23 cm among pregnancy women: decreased from 41% to 37% (intervention arm) vs. 45% to 35% (control arm).

In adolescents (non-pregnant): % Thin (BMI <18.5) : decreased from 18% to 13% (intervention arm) vs/. 15% to 12% (control arm).

**Preliminary consideration of confounders**

Complete a row for each important confounding domain (i) listed in the review protocol; and (ii) relevant to the setting of this particular study, or which the study authors identified as potentially important.

#### “Important” confounding domains are those for which, in the context of this study, adjustment is expected to lead to a clinically important change in the estimated effect of the intervention. “Validity” refers to whether the confounding variable or variables fully measure the domain, while “reliability” refers to the precision of the measurement (more measurement error means less reliability).

| **(i) Confounding domains listed in the review protocol** | | | | |
| --- | --- | --- | --- | --- |
| Confounding domain | Measured variable(s) | Is there evidence that controlling for this variable was unnecessary?* | Is the confounding domain measured validly and reliably by this variable (or these variables)? | OPTIONAL: Is failure to adjust for this variable (alone) expected to favour the experimental intervention or the comparator? |
|  |  |  | Yes / No / No information | Favour experimental / Favour comparator / No information |

| **(ii) Additional confounding domains relevant to the setting of this particular study, or which the study authors identified as important** | | | | |
| --- | --- | --- | --- | --- |
| Confounding domain | Measured variable(s) | Is there evidence that controlling for this variable was unnecessary?* | Is the confounding domain measured validly and reliably by this variable (or these variables)? | OPTIONAL: Is failure to adjust for this variable (alone) expected to favour the experimental intervention or the comparator? |
|  |  |  | Yes / No / No information | Favour experimental / Favour comparator / No information |
| Socioeconomic | Education (none, primary, secondary, higher secondary +)  Adolescent in school or not | No significant difference in education using Chi squared for the 3 groups (adolescents, pregnant (PW) and mothers of children <2) | Yes (but self-reported by participant) |  |
| Socioeconomic | Wealth (top/bottom 50%) | No significant difference top and bottom 50% using Chi squared for the 3 groups (adolescents, pregnant (PW) and mothers of children <2) | Yes (from self-reported list of assets) |  |
| Socioeconomic | Religion (Hindu/other) | using Chi squared there were significantly more Hindus in intervention arm for adolescents, and mothers of children <2). It says Not significant for pregnant (PW) but difference is same as in the mothers. | Yes (but self-reported by participant) |  |
| Socioeconomic | Household Social class / Caste (SC/ST/OBC/other) | No significant difference in caste sing Chi squared for the 3 groups (adolescents, pregnant (PW) and mothers of children <2) but some differences | Yes (but self-reported by participant) |  |
| Age of participant | Adolescent (10-14y; 15-19y) | No significant difference in age group using Chi squared | Yes (but self-reported by participant and some people may not know their age) |  |
| Age of participant | Pregnant women & Mothers (<20, 20-29years; >=30 years) | No significant difference in age group using Chi squared for mothers but in PW there were more adolescents and more older women by 2+ percentage points | Yes (but self-reported by participant and some people may not know their age) |  |
| Exposure to intervention | PLA meetings attended |  |  |  |
| Exposure to intervention | VHSND meetings attended |  |  |  |
| Exposure to intervention | AHD meetings attended |  |  |  |
| Exposure to intervention | Frequency of PLA meeting attendance in last 3 years (none, 1-5 times, 6+ times) |  |  |  |

* In the context of a particular study, variables can be demonstrated not to be confounders and so not included in the analysis: (a) if they are not predictive of the outcome; (b) if they are not predictive of intervention; or (c) because adjustment makes no or minimal difference to the estimated effect of the primary parameter. Note that “no statistically significant association” is not the same as “not predictive”.

**Preliminary consideration of co-interventions**

Complete a row for each important co-intervention (i) listed in the review protocol; and (ii) relevant to the setting of this particular study, or which the study authors identified as important.

#### “Important” co-interventions are those for which, in the context of this study, adjustment is expected to lead to a clinically important change in the estimated effect of the intervention.

| **(i) Co-interventions listed in the review protocol** | | |
| --- | --- | --- |
| Co-intervention | Is there evidence that controlling for this co-intervention was unnecessary (e.g. because it was not administered)? | Is presence of this co-intervention likely to favour outcomes in the experimental intervention or the comparator |
|  |  | Favour experimental / Favour comparator / No information |

| **(ii) Additional co-interventions relevant to the setting of this particular study, or which the study authors identified as important** | | |
| --- | --- | --- |
| Co-intervention | Is there evidence that controlling for this co-intervention was unnecessary (e.g. because it was not administered)? | Is presence of this co-intervention likely to favour outcomes in the experimental intervention or the comparator |
|  |  | Favour experimental / Favour comparator / No information |

Risk of bias assessment

Responses underlined in green are potential markers for low risk of bias, and responses in red are potential markers for a risk of bias. Where questions relate only to sign posts to other questions, no formatting is used.

|  | **Signalling questions** | **Description** | **Response options** |
| --- | --- | --- | --- |
| **Bias due to confounding** | | | |
|  | 1.1 Is there potential for confounding of the effect of intervention in this study?  **If N/PN to 1.1:** the study can be considered to be at low risk of bias due to confounding and no further signalling questions need be considered | Yes, because there are differences between arms in terms of age, religion, (which were significant in some groups) and slightly by caste, education (though not significantly) | PY |
|  | **If Y/PY to 1.1**: determine whether there is a need to assess time-varying confounding: |  |  |
|  | 1.2. Was the analysis based on splitting participants’ follow up time according to intervention received?  **If N/PN**, answer questions relating to baseline confounding (1.4 to 1.6)  **If Y/PY**, go to question 1.3. |  | N |
|  | 1.3. Were intervention discontinuations or switches likely to be related to factors that are prognostic for the outcome?  **If N/PN**, answer questions relating to baseline confounding (1.4 to 1.6)  **If Y/PY**, answer questions relating to both baseline and time-varying confounding (1.7 and 1.8) |  |  |

|  | **Questions relating to baseline confounding only** | | |
| --- | --- | --- | --- |
|  | 1.4. Did the authors use an appropriate analysis method that controlled for all the important confounding domains? | Multivariable logistic regression was applied to endline data to examine the association between receipt of nutrition specific services and nutrition outcomes with status of participation, adjusted for socio-economic (education, quintile) characteristics.  At end of table 9 they say, “Adjusted for socio-economic characteristics of the respondent such as age, education, wealth, social group and religion.”  Quintile is mentioned but the indicator shown is top and bottom 50%, unclear which was used. The different reports of analyses in tables do not specify the confounders adjusted for so we don’t know if age and caste were adjusted for. Adjustment for age and caste was necessary as arms differed by these variables. | PY |
|  | 1.5. **If Y/PY to 1.4**: Were confounding domains that were controlled for measured validly and reliably by the variables available in this study? | Socio-economic characteristics are based on self-report (subjective) having lower validity and reliability. Age is often not accurately recalled or not known, especially in older women. | PY |
|  | 1.6. Did the authors control for any post-intervention variables that could have been affected by the intervention? | There were no variables of this kind | N |
|  | **Questions relating to baseline and time-varying confounding** | |  |
|  | 1.7. Did the authors use an appropriate analysis method that controlled for all the important confounding domains and for time-varying confounding? | The authors used a difference in differences analysis which adjusts for changes over time between baseline and endline, but they have used different blocks within the district at baseline and endline for Bihar and Odisha, which may not be comparable. | PN |
|  | 1.8. **If Y/PY to 1.7**: Were confounding domains that were controlled for measured validly and reliably by the variables available in this study? |  |  |
|  | **Risk of bias judgement** | The study appears to provide sound evidence for a non-randomized study but cannot be considered comparable to a well-performed randomized trial.  (i) Confounding expected, all known important confounding  domains appropriately measured and controlled for, but for some analysis authors have not reported which domains were adjusted.  and  (ii) Reliability and validity of measurement of important domains  were sufficient, such that we do not expect serious residual  confounding. | Moderate |
|  | Optional: What is the predicted direction of bias due to confounding? | There is no reason to believe that unmeasured confounders and time variation will act in favour of a specific group. | Unpredictable |

| **Bias in selection of participants into the study** | | | |
| --- | --- | --- | --- |
|  | 2.1. Was selection of participants into the study (or into the analysis) based on participant characteristics observed after the start of intervention?  **If N/PN to 2.1:** go to 2.4 | Participants were selected based on the inclusion criteria which was prespecified in the protocol based on the age, pregnancy and maternal status. | N |
|  | 2.2. **If Y/PY to 2.1**: Were the post-intervention variables that influenced selection likely to be associated with intervention?  2.3 **If Y/PY to 2.2**: Were the post-intervention variables that influenced selection likely to be influenced by the outcome or a cause of the outcome? |  |  |
|  | 2.4. Do start of follow-up and start of intervention coincide for most participants? | No information on the dates of the endline (2021-2022) survey or on the dates of initiating the intervention. | NI |
|  | 2.5. **If Y/PY to 2.2 and 2.3, or N/PN to 2.4**: Were adjustment techniques used that are likely to correct for the presence of selection biases? |  |  |
|  | **Risk of bias judgement** | (ii) Start of follow up and start of intervention do not coincide for all  participants;  and  (a) the proportion of participants for which this was the case  was too low to induce important bias;  There is insufficient information on the dates of the endline survey or on the dates of initiating the intervention to demonstrate that they coincide for all participants. | Moderate |
|  | Optional: What is the predicted direction of bias due to selection of participants into the study? |  | Favours experimental / Favours comparator / Towards null /Away from null / Unpredictable |

| **Bias in classification of interventions** | | | |
| --- | --- | --- | --- |
|  | 3.1 Were intervention groups clearly defined? | Interventions are well defined in the protocol: Nutrition sensitive and specific intervention. | Y |
|  | 3.2 Was the information used to define intervention groups recorded at the start of the intervention? |  | Y |
|  | 3.3 Could classification of intervention status have been affected by knowledge of the outcome or risk of the outcome? | These were prespecified in the protocol before the outcome was known. | N |
|  | **Risk of bias judgement** | the study is comparable to a well-performed randomized trial with regard to this  domain)  (i) Intervention status is well defined;  and  (ii) Intervention definition is based solely on information collected at the time of intervention. | Low |
|  | Optional: What is the predicted direction of bias due to classification of interventions? |  | Favours experimental / Favours comparator / Towards null /Away from null / Unpredictable |

| **Bias due to deviations from intended interventions** | | | |
| --- | --- | --- | --- |
|  | **If your aim for this study is to assess the effect of assignment to intervention, answer questions 4.1 and 4.2** | |  |
|  | 4.1. Were there deviations from the intended intervention beyond what would be expected in usual practice? | There was no deviation compared with the protocol. Nutritionally at-risk women were visited by Poshan Sakhis, but during COVID pandemic these home visits were also made by ICDS frontline and health workers. | PN |
|  | 4.2. **If Y/PY to 4.1**: Were these deviations from intended intervention unbalanced between groups *and* likely to have affected the outcome? |  |  |
|  | **If your aim for this study is to assess the effect of starting and adhering to intervention, answer questions 4.3 to 4.6** | |  |
|  | 4.3. Were important co-interventions balanced across intervention groups? |  | Y / PY / PN / N / NI |
|  | 4.4. Was the intervention implemented successfully for most participants? |  | Y / PY / PN / N / NI |
|  | 4.5. Did study participants adhere to the assigned intervention regimen? |  | Y / PY / PN / N / NI |
|  | 4.6. **If N/PN to 4.3, 4.4 or 4.5**: Was an appropriate analysis used to estimate the effect of starting and adhering to the intervention? |  | NA / Y / PY / PN / N / NI |
|  | **Risk of bias judgement** | The study is comparable to a well-performed randomized trial with regard to this  domain:  Effect of assignment to intervention:  (i) Any deviations from intended intervention reflected usual practice.  or  (ii) Any deviations from usual practice were unlikely to impact on the outcome. | Low |
|  | Optional: What is the predicted direction of bias due to deviations from the intended interventions? |  | Favours experimental / Favours comparator / Towards null /Away from null / Unpredictable |

| **Bias due to missing data** | | | |
| --- | --- | --- | --- |
|  | 5.1 Were outcome data available for all, or nearly all, participants? | They randomly selected participants from a household list in primary sampling unit. But author do not report about nonresponse or dropout rate. | NI |
|  | 5.2 Were participants excluded due to missing data on intervention status? |  | NI |
|  | 5.3 Were participants excluded due to missing data on other variables needed for the analysis? |  | NI |
|  | 5.4 **If PN/N to 5.1, or Y/PY to 5.2 or 5.3**: Are the proportion of participants and reasons for missing data similar across interventions? |  | NA / Y / PY / PN / N / NI |
|  | 5.5 **If PN/N to 5.1, or Y/PY to 5.2 or 5.3**: Is there evidence that results were robust to the presence of missing data? |  | NA / Y / PY / PN / N / NI |
|  | **Risk of bias judgement** | No information on which to base a judgement about risk of bias for this  domain  No information is reported about missing data or the potential for  data to be missing. | NI |
|  | Optional: What is the predicted direction of bias due to missing data? |  | Favours experimental / Favours comparator / Towards null /Away from null / Unpredictable |

| **Bias in measurement of outcomes** | | | |
| --- | --- | --- | --- |
|  | 6.1 Could the outcome measure have been influenced by knowledge of the intervention received? | Data collection teams and analysts are blind to allocation. | N |
|  | 6.2 Were outcome assessors aware of the intervention received by study participants? | Data collection teams and analysts are blind to allocation. | N |
|  | 6.3 Were the methods of outcome assessment comparable across intervention groups? | Standard anthropometric measurements and cut off were applied across intervention groups. | Y |
|  | 6.4 Were any systematic errors in measurement of the outcome related to intervention received? |  | PN |
|  | **Risk of bias judgement** | the study is comparable to a well-performed randomized trial with regard to this domain)  (i) The methods of outcome assessment were comparable across  intervention groups;  and  (ii) The outcome measure was unlikely to be influenced by  knowledge of the intervention received by study participants (i.e. is  objective) or the outcome assessors were unaware of the intervention  received by study participants;  and  (iii) Any error in measuring the outcome is unrelated to intervention  status. | Low |
|  | Optional: What is the predicted direction of bias due to measurement of outcomes? |  | Favours experimental / Favours comparator / Towards null /Away from null / Unpredictable |

| **Bias in selection of the reported result** | | | |
| --- | --- | --- | --- |
|  | Is the reported effect estimate likely to be selected, on the basis of the results, from... |  |  |
|  | 7.1. ... multiple outcome *measurements* within the outcome domain? |  | N |
|  | 7.2 ... multiple *analyses* of the intervention-outcome relationship? |  | N |
|  | 7.3 ... different *subgroups*? |  | N |
|  | **Risk of bias judgement** | The study is comparable to a well-performed randomized trial with regard to this domain  There is clear evidence (usually through examination of a pre-registered protocol or statistical analysis plan) that all reported results correspond to all intended outcomes, analyses and sub-cohorts. | Low |
|  | Optional: What is the predicted direction of bias due to selection of the reported result? |  | Favours experimental / Favours comparator / Towards null /Away from null / Unpredictable |

| **Overall bias** | | | |
| --- | --- | --- | --- |
|  | **Risk of bias judgement** | The study appears to provide sound evidence for a non-randomized study but cannot be considered comparable to a well-performed randomized trial.  The study is judged to be at low or moderate risk of bias for all domains.  Moderate for confounding and risk of selection of participants. | Moderate |
|  | Optional: What is the overall predicted direction of bias for this outcome? |  | Favours experimental / Favours comparator / Towards null /Away from null / Unpredictable |

This work is licensed under a [Creative Commons Attribution-NonCommercial-NoDerivatives 4.0 International License](http://creativecommons.org/licenses/by-nc-nd/4.0/).

# 15. **Supplementary file S13. The Risk Of Bias In Non-randomized Studies – of Interventions (ROBINS-I) assessment tool filled for Doke 2024.**

Doke PP, Chutke PA, Palkar HS, et al. Implementation of preconception care for preventing adverse pregnancy outcomes in rural and tribal areas of Nashik District, India. *Prev Med Rep* 2024; **43**: 102796.

**The Risk Of Bias In Non-randomized Studies – of Interventions (ROBINS-I) assessment tool**

(version for cohort-type studies)

**Version 19 September 2016**

This work is licensed under a [Creative Commons Attribution-NonCommercial-NoDerivatives 4.0 International License](http://creativecommons.org/licenses/by-nc-nd/4.0/).

ROBINS-I tool (Stage I): At protocol stage

Specify the review question

| Participants |  |
| --- | --- |
| Experimental intervention |  |
| Comparator |  |
| Outcomes |  |

**List the confounding domains relevant to all or most studies**

**List co-interventions that could be different between intervention groups and that could impact on outcomes**

**ROBINS-I tool (Stage II): For each study**

Specify a target randomized trial specific to the study

| Design | Community-based implementation study |
| --- | --- |
| Participants | Women aged 15–49 years aspiring to have a baby. (Actual age of enrolled women 14.42 to 45.5 years; mean 23.2 +/- 3.7). |
| Experimental intervention | Complex preconception care intervention involving:  -Health screening / treatment   - Anaemia prevention/ treatment with IFA - Family planning services - Multi-channel Behaviour Change Communication at VHSNDs and home - Tailored counselling for BMI optimisation   Nutrition-sensitive counselling |
| Comparator | Routine care. |

**Is your aim for this study…?**

| X |  | to assess the effect of *assignment to* intervention |
| --- | --- | --- |
| £ |  | to assess the effect of *starting and adhering to* intervention |

**Specify the outcome**

Specify which outcome is being assessed for risk of bias (typically from among those earmarked for the Summary of Findings table). Specify whether this is a proposed benefit or harm of intervention.

-LBW (birth weight < 2500 g)
-Preterm birth

**Specify the numerical result being assessed**

In case of multiple alternative analyses being presented, specify the numeric result (e.g. RR = 1.52 (95% CI 0.83 to 2.77) and/or a reference (e.g. to a table, figure or paragraph) that uniquely defines the result being assessed.

-LBW: 10.28 % in Arm 1 and 10.95% in Arm 2, *p* 0.66
-Preterm: 12.63% in Arm 1 and 16.72% in Arm 2, *p* = 0.02.

**Preliminary consideration of confounders**

Complete a row for each important confounding domain (i) listed in the review protocol; and (ii) relevant to the setting of this particular study, or which the study authors identified as potentially important.

#### “Important” confounding domains are those for which, in the context of this study, adjustment is expected to lead to a clinically important change in the estimated effect of the intervention. “Validity” refers to whether the confounding variable or variables fully measure the domain, while “reliability” refers to the precision of the measurement (more measurement error means less reliability).

| **(i) Confounding domains listed in the review protocol** | | | | |
| --- | --- | --- | --- | --- |
| Confounding domain | Measured variable(s) | Is there evidence that controlling for this variable was unnecessary?* | Is the confounding domain measured validly and reliably by this variable (or these variables)? | OPTIONAL: Is failure to adjust for this variable (alone) expected to favour the experimental intervention or the comparator? |
|  |  |  | Yes / No / No information | Favour experimental / Favour comparator / No information |
|  |  |  |  |  |

| **(ii) Additional confounding domains relevant to the setting of this particular study, or which the study authors identified as important** | | | | |
| --- | --- | --- | --- | --- |
| Confounding domain | Measured variable(s) | Is there evidence that controlling for this variable was unnecessary?* | Is the confounding domain measured validly and reliably by this variable (or these variables)? | OPTIONAL: Is failure to adjust for this variable (alone) expected to favour the experimental intervention or the comparator? |
|  |  |  | Yes / No / No information | Favour experimental / Favour comparator / No information |
| Socioeconomic | Caste (Scheduled tribe (ST) and non-ST) | **Significant difference in baseline** group using Chi squared | Yes (but self-reported by participant) |  |
| Socioeconomic | Family type  (nuclear, non-nuclear) | No significant difference in baseline using Chi squared | Yes (but self-reported by participant) |  |
| Socioeconomic | Education (<SSC, >= SSC) | **Significant difference in baseline** using Chi squared | Yes (but self-reported by participant) |  |
| Socioeconomic | Occupation (employed, unemployed) | **Significant difference in baseline** group using Chi squared | Yes (but self-reported by participant) |  |
| Age of participant | <20, 20–35, >35 | No significant difference in baseline using Chi squared | Yes (but self-reported by participant) |  |
| Biological risk factors | BMI | No significant difference in baseline using Chi squared | Yes (but self-reported by participant) |  |
| Biological risk factors | Consanguine marriage | **Significant difference in baseline** group using Chi squared | Yes (but self-reported by participant) |  |
| Biological risk factors | Multiparity | No significant difference in baseline using Chi squared | Yes (but self-reported by participant) |  |
| Biological risk factors | Tobacco | No significant difference in baseline using Chi squared | Yes (but self-reported by participant) |  |
| Biological risk factors | Calorie intake | No significant difference in baseline using Chi squared | Yes (but self-reported by participant) |  |
| Biological risk factors | Protein intake | **Significant difference in baseline** using Chi squared. | Yes (but self-reported by participant) |  |

* In the context of a particular study, variables can be demonstrated not to be confounders and so not included in the analysis: (a) if they are not predictive of the outcome; (b) if they are not predictive of intervention; or (c) because adjustment makes no or minimal difference to the estimated effect of the primary parameter. Note that “no statistically significant association” is not the same as “not predictive”.

**Preliminary consideration of co-interventions**

Complete a row for each important co-intervention (i) listed in the review protocol; and (ii) relevant to the setting of this particular study, or which the study authors identified as important.

#### “Important” co-interventions are those for which, in the context of this study, adjustment is expected to lead to a clinically important change in the estimated effect of the intervention.

| **(i) Co-interventions listed in the review protocol** | | |
| --- | --- | --- |
| Co-intervention | Is there evidence that controlling for this co-intervention was unnecessary (e.g. because it was not administered)? | Is presence of this co-intervention likely to favour outcomes in the experimental intervention or the comparator |
|  |  | Favour experimental / Favour comparator / No information |

| **(ii) Additional co-interventions relevant to the setting of this particular study, or which the study authors identified as important** | | |
| --- | --- | --- |
| Co-intervention | Is there evidence that controlling for this co-intervention was unnecessary (e.g. because it was not administered)? | Is presence of this co-intervention likely to favour outcomes in the experimental intervention or the comparator |
|  |  | Favour experimental / Favour comparator / No information |

**Risk of bias assessment**

Responses underlined in green are potential markers for low risk of bias, and responses in red are potential markers for a risk of bias. Where questions relate only to sign posts to other questions, no formatting is used.

|  | **Signalling questions** | **Description** | **Response options** |
| --- | --- | --- | --- |
| **Bias due to confounding** | | | |
|  | 1.1 Is there potential for confounding of the effect of intervention in this study?  **If N/PN to 1.1:** the study can be considered to be at low risk of bias due to confounding and no further signalling questions need be considered | Yes, because intervention and control differ in risk factors: caste, education, occupation, consanguine marriage and protein intake among women with pregnancy outcomes between intervention and control arm (p<0.05) – Table 1  Intervention and control differ in type of family though not significantly – Table 1  There are differences between arms in risk factors calorie intake and BMI but not significant – Table 1  Significant difference in pregnancy outcomes live births and abortions by study arms – Table 2 | Y |
|  | **If Y/PY to 1.1**: determine whether there is a need to assess time-varying confounding: |  |  |
|  | 1.2. Was the analysis based on splitting participants’ follow up time according to intervention received?  **If N/PN**, answer questions relating to baseline confounding (1.4 to 1.6)  **If Y/PY**, go to question 1.3. |  | N |
|  | 1.3. Were intervention discontinuations or switches likely to be related to factors that are prognostic for the outcome?  **If N/PN**, answer questions relating to baseline confounding (1.4 to 1.6)  **If Y/PY**, answer questions relating to both baseline and time-varying confounding (1.7 and 1.8) |  | N |

|  | **Questions relating to baseline confounding only** | | |
| --- | --- | --- | --- |
|  | 1.4. Did the authors use an appropriate analysis method that controlled for all the important confounding domains? | The authors state that “multivariate analysis was indispensable” because of the baseline differences between study arms in caste, education, occupation, consanguineous marriage, and protein intake. But then they say “In this, we included variables those were significantly associated with outcome variables in bivariate analysis”. This implies that the imbalanced sociodemographic factors were not all included in the model a priori, which should have been the case to control for baseline imbalance. Univariate analysis was undertaken for crude prevalence ratios between arms for neonatal death. Multivariate analyses were used to derive adjusted prevalence ratio for the following pregnancy outcomes: **Preterm and congenital anomaly (adjusted for caste)**, abortion (adjusted for family type and BMI) and stillbirth (adjusted for education and caste). Which variables are adjusted for is assumed from the table 3 but not mentioned specifically in the text.  Table 2 mentions that arms differed amongst all women delivering with lower preterm births, but higher congenital anomalies, abortions/miscarriages, stillbirths. Whereas amongst those who had 6 months of the intervention differences between arms were found with lower preterm and livebirths, and higher miscarriage/abortion in intervention areas. But none of these analyses were adjusted for confounders despite major differences between arms. | N |
|  | 1.5. **If Y/PY to 1.4**: Were confounding domains that were controlled for measured validly and reliably by the variables available in this study? | Socio-economic characteristics and biological risk factors are based on self-report (subjective) having lower validity and reliability. Age is often not accurately recalled or not known, especially in older women.  The method of capture of the pregnancy outcome differed by arm. It was done by intervention ANMs and ASHAs in the intervention arm and only ANMS ‘later’ in the control arm. ANMs collected data on tablets in the intervention arms but only on paper in the control arms and data appear to have been collected whilst undertaking the intervention but may have been missing or collected differently in the control arm. This is not clearly explained.  The information about the pregnancy outcomes were collected through home visits by ASHAs and were confirmed through ‘documents’ but it is not explained what documents were used. ASHAs are usually not highly educated so there could be some error in reporting. Moreover, it appears that ASHAs were conducting intensive visits in the intervention arm only, so it seems like data collection processes differed widely arm. | PN |
|  | 1.6. Did the authors control for any post-intervention variables that could have been affected by the intervention? | No | N |
|  | **Questions relating to baseline and time-varying confounding** | |  |
|  | 1.7. Did the authors use an appropriate analysis method that controlled for all the important confounding domains and for time-varying confounding? | They used z tests to compare crude outcomes in Table 2 (unadjusted). For baseline differences between arms they used to determine the p value Chi-Squared test to determine if the differences were significant. When the author chose confounders for adjusted prevalence ratios, they only adjusted for variables that were associated with the outcome, whereas adjustment for all the variables which were imbalanced at baseline would have been a better approach. The final LBW model for adjusted prevalence ratio removed the intervention arm from the model, so the adjusted effect of the intervention on LBW is not reported. | N |
|  | 1.8. **If Y/PY to 1.7**: Were confounding domains that were controlled for measured validly and reliably by the variables available in this study? | The confounding domains were based upon self-report, which may have limited reliability and validity. Data were collected by government health workers (ANMs) who may be less able to devote time to proper recording and reporting than dedicated data collectors. The domains seem to have been collected differently in intervention and control arms. |  |
|  | **Risk of bias judgement** | The non-randomized study provides some evidence but cannot be considered comparable to a well-performed randomized trial.  (i) Confounding expected, some important confounding  domains appropriately measured and controlled for, but not all imbalanced confounders were adjusted for. Moreover, the authors have not reported clearly which domains were adjusted for – the reader has to assume from Table 3.  and  (ii) Reliability and validity of measurement of important domains  were sufficient, was low that we suspect serious residual  confounding. The method of measuring birthweight was not clear. | Serious |
|  | Optional: What is the predicted direction of bias due to confounding? |  | Favours experimental / Favours comparator / Unpredictable |

| **Bias in selection of participants into the study** | | | |
| --- | --- | --- | --- |
|  | 2.1. Was selection of participants into the study (or into the analysis) based on participant characteristics observed after the start of intervention?  **If N/PN to 2.1:** go to 2.4 | The selection was not based on participant characteristics but based on the geographic location where the participant resides and seeks care. | N |
|  | 2.2. **If Y/PY to 2.1**: Were the post-intervention variables that influenced selection likely to be associated with intervention?  2.3 **If Y/PY to 2.2**: Were the post-intervention variables that influenced selection likely to be influenced by the outcome or a cause of the outcome? | There were no post-intervention variables that influenced selection. | N  N |
|  | 2.4. Do start of follow-up and start of intervention coincide for most participants? |  | NI |
|  | 2.5. **If Y/PY to 2.2 and 2.3, or N/PN to 2.4**: Were adjustment techniques used that are likely to correct for the presence of selection biases? |  |  |
|  | **Risk of bias judgement** | (i) Intervention status is well defined;  and  (ii) Some aspects of the assignments of intervention status were not reported. | Moderate |
|  | Optional: What is the predicted direction of bias due to selection of participants into the study? |  | Favours experimental / Favours comparator / Towards null /Away from null / Unpredictable |

| **Bias in classification of interventions** | | | |
| --- | --- | --- | --- |
|  | 3.1 Were intervention groups clearly defined? | The population groups were clearly defined by their cluster allocation (block).  The intervention involved health screening/treatment; anaemia prevention/treatment with IFA; family planning services; multi-channel behaviour change communication at VHSNDs and home; tailored counselling for BMI optimisation and nutrition sensitive counselling.  The intervention description did not report the ‘fidelity’ of the intervention to the protocol nor the ‘dose’ received by participants. | N |
|  | 3.2 Was the information used to define intervention groups recorded at the start of the intervention? | There were two randomly selected interventions blocks and two purposively selected adjacent blocks for comparison. Each study arm had one tribal block (block having a high proportion of the Scheduled Tribes population) and one non- tribal block to cover both tribal and non-tribal populations. | Y |
|  | 3.3 Could classification of intervention status have been affected by knowledge of the outcome or risk of the outcome? |  | NI |
|  | **Risk of bias judgement** | (i) Intervention status is not well defined; | Serious |
|  | Optional: What is the predicted direction of bias due to classification of interventions? |  | Favours experimental / Favours comparator / Towards null /Away from null / Unpredictable |

| **Bias due to deviations from intended interventions** | | | |
| --- | --- | --- | --- |
|  | **If your aim for this study is to assess the effect of assignment to intervention, answer questions 4.1 and 4.2** | |  |
|  | 4.1. Were there deviations from the intended intervention beyond what would be expected in usual practice? | There is no protocol. | NI |
|  | 4.2. **If Y/PY to 4.1**: Were these deviations from intended intervention unbalanced between groups *and* likely to have affected the outcome? |  |  |
|  | **If your aim for this study is to assess the effect of starting and adhering to intervention, answer questions 4.3 to 4.6** | |  |
|  | 4.3. Were important co-interventions balanced across intervention groups? |  |  |
|  | 4.4. Was the intervention implemented successfully for most participants? |  |  |
|  | 4.5. Did study participants adhere to the assigned intervention regimen? |  |  |
|  | 4.6. **If N/PN to 4.3, 4.4 or 4.5**: Was an appropriate analysis used to estimate the effect of starting and adhering to the intervention? |  |  |
|  | **Risk of bias judgement** | No information is reported on whether there is deviation from the  intended intervention. | NI |
|  | Optional: What is the predicted direction of bias due to deviations from the intended interventions? |  | Favours experimental / Favours comparator / Towards null /Away from null / Unpredictable |

| **Bias due to missing data** | | | |
| --- | --- | --- | --- |
|  | 5.1 Were outcome data available for all, or nearly all, participants? | 1726 women had pregnancy outcomes in intervention group and 1875 in control group. All were included for analysis. N is mentioned in Table 2 but not in table 3 so the numbers in the adjusted analyses are not provided. | PY |
|  | 5.2 Were participants excluded due to missing data on intervention status? | Have not reported about missing data. | NI |
|  | 5.3 Were participants excluded due to missing data on other variables needed for the analysis? |  | NI |
|  | 5.4 **If PN/N to 5.1, or Y/PY to 5.2 or 5.3**: Are the proportion of participants and reasons for missing data similar across interventions? |  | NI |
|  | 5.5 **If PN/N to 5.1, or Y/PY to 5.2 or 5.3**: Is there evidence that results were robust to the presence of missing data? |  | NI |
|  | **Risk of bias judgement** | Data seem to be reasonably complete as per the participant flow diagram, but the information on completeness of adjusted analyses is missing and there is no report on missing data. | Moderate |
|  | Optional: What is the predicted direction of bias due to missing data? |  | Favours experimental / Favours comparator / Towards null /Away from null / Unpredictable |

| **Bias in measurement of outcomes** | | | |
| --- | --- | --- | --- |
|  | 6.1 Could the outcome measure have been influenced by knowledge of the intervention received? | There is no information about birthweight measurement and how it was undertaken. It appears that some differences in measurement may have been there between arms. | NI |
|  | 6.2 Were outcome assessors aware of the intervention received by study participants? | No mention of blinding. ASHAs and ANMs who delivered the intervention were collecting the data in tablets (in the intervention arm only) and confirmed the information on pregnancy outcomes through health facility documents. | Y |
|  | 6.3 Were the methods of outcome assessment comparable across intervention groups? | Although broadly the same methods were used for all participants some data were collected during home visits in intervention arms but via health facility records for control arm. Tablets were provided by the government to ANMs in the intervention arms only which reflects systematic differences in how data were recorded. | PN |
|  | 6.4 Were any systematic errors in measurement of the outcome related to intervention received? |  | NI |
|  | **Risk of bias judgement** | i) The methods of outcome assessment were not comparable across  intervention groups (because tablets were used in intervention but not in control areas);  or  ii) The outcome measure was subjective (i.e. vulnerable to influence  by knowledge of the intervention received by study participants);  and  iii) The outcome was assessed by assessors aware of (and involved in) the intervention received by study participants. | Serious risk of bias |
|  | Optional: What is the predicted direction of bias due to measurement of outcomes? |  | Favours experimental / Favours comparator / Towards null /Away from null / Unpredictable |

| **Bias in selection of the reported result** | | | |
| --- | --- | --- | --- |
|  | Is the reported effect estimate likely to be selected, on the basis of the results, from... |  |  |
|  | 7.1. ... multiple outcome *measurements* within the outcome domain? |  | PN |
|  | 7.2 ... multiple *analyses* of the intervention-outcome relationship? |  | PN |
|  | 7.3 ... different *subgroups*? |  | PN |
|  | **Risk of bias judgement** | The study is comparable to a well-performed randomized trial with regard to this domain. | Low |
|  | Optional: What is the predicted direction of bias due to selection of the reported result? |  | Favours experimental / Favours comparator / Towards null /Away from null / Unpredictable |

| **Overall bias** | | | |
| --- | --- | --- | --- |
|  | **Risk of bias judgement** | The study is judged to be a serious risk of bias in at least one domain but not at critical risk of bias in any domain. | Serious risk of bias |
|  | Optional: What is the overall predicted direction of bias for this outcome? |  | Favours experimental / Favours comparator / Towards null /Away from null / Unpredictable |

This work is licensed under a [Creative Commons Attribution-NonCommercial-NoDerivatives 4.0 International License](http://creativecommons.org/licenses/by-nc-nd/4.0/).

# 16. **Supplementary File S14. References cited in supplementary material**

D'Souza, N., Behere, R. V., Patni, B., Deshpande, M., Bhat, D., Bhalerao, A., Sonawane, S., Shah, R., Ladkat, R., Yajnik, P., Bandyopadhyay, S. K., Kumaran, K., Fall, C., & Yajnik, C. S. (2021). Pre-conceptional Maternal Vitamin B12 Supplementation Improves Offspring Neurodevelopment at 2 Years of Age: PRIYA Trial. *Front Pediatr*, *9*, 755977. <https://doi.org/10.3389/fped.2021.755977>

Dhaded, S. M., Hambidge, K. M., Ali, S. A., Somannavar, M., Saleem, S., Pasha, O., Khan, U., Herekar, V., Vernekar, S., Kumar, S. Y., Westcott, J. E., Thorsten, V. R., Sridhar, A., Das, A., McClure, E., Derman, R. J., Goldenberg, R. L., Koso-Thomas, M., Goudar, S. S., & Krebs, N. F. (2020). Preconception nutrition intervention improved birth length and reduced stunting and wasting in newborns in South Asia: The Women First Randomized Controlled Trial. *PLoS One*, *15*(1), e0218960. <https://doi.org/10.1371/journal.pone.0218960>

Gov. of Maharashtra, UNICEF, & Bharati Vidyapeeth (Deemed to be University) Medical College Pune. (2021). *Healthy Parents, Healthy Child Initiative: Report of intervention that promoted the health of women desigin pregnancy and reduced low birth weight and newborn deaths in Nashik, Maharashtra, India.* (ISBN: 978-93-5578-838-2).

Hambidge, K. M., Bann, C. M., McClure, E. M., Westcott, J. E., Garces, A., Figueroa, L., Goudar, S. S., Dhaded, S. M., Pasha, O., Ali, S. A., Derman, R. J., Goldenberg, R. L., Koso-Thomas, M., Somannavar, M. S., Herekar, V., Khan, U., & Krebs, N. F. (2019). Maternal Characteristics Affect Fetal Growth Response in the Women First Preconception Nutrition Trial. *Nutrients*, *11*(10). <https://doi.org/10.3390/nu11102534>

Hambidge, K. M., Krebs, N. F., Westcott, J. E., Garces, A., Goudar, S. S., Kodkany, B. S., Pasha, O., Tshefu, A., Bose, C. L., Figueroa, L., Goldenberg, R. L., Derman, R. J., Friedman, J. E., Frank, D. N., McClure, E. M., Stolka, K., Das, A., Koso-Thomas, M., Sundberg, S., & Preconception Trial, G. (2014). Preconception maternal nutrition: a multi-site randomized controlled trial. *BMC Pregnancy Childbirth*, *14*(1), 111. <https://doi.org/10.1186/1471-2393-14-111>

Hambidge, K. M., Westcott, J. E., Garces, A., Figueroa, L., Goudar, S. S., Dhaded, S. M., Pasha, O., Ali, S. A., Tshefu, A., Lokangaka, A., Derman, R. J., Goldenberg, R. L., Bose, C. L., Bauserman, M., Koso-Thomas, M., Thorsten, V. R., Sridhar, A., Stolka, K., Das, A.,…Women First Preconception Trial Study, G. (2019). A multicountry randomized controlled trial of comprehensive maternal nutrition supplementation initiated before conception: the Women First trial. *Am J Clin Nutr*, *109*(2), 457-469. <https://doi.org/10.1093/ajcn/nqy228>

ICMR. (2000). Multicentric study of efficacy of periconceptional folic acid containing vitamin supplementation in prevention of open neural tube defects from India. . *Indian J Med Res*, *112*, 206–211.

Katz, J., West, K. P., Jr., Khatry, S. K., Pradhan, E. K., LeClerq, S. C., Christian, P., Wu, L. S., Adhikari, R. K., Shrestha, S. R., & Sommer, A. (2000). Maternal low-dose vitamin A or beta-carotene supplementation has no effect on fetal loss and early infant mortality: a randomized cluster trial in Nepal [Clinical Trial Randomized Controlled Trial Research Support, Non-U.S. Gov't Research Support, U.S. Gov't, Non-P.H.S.]. *Am J Clin Nutr*, *71*(6), 1570-1576. <https://doi.org/10.1093/ajcn/71.6.1570>

Khambalia, A. Z., O'Connor, D. L., Macarthur, C., Dupuis, A., & Zlotkin, S. H. (2009). Periconceptional iron supplementation does not reduce anemia or improve iron status among pregnant women in rural Bangladesh. *Am J Clin Nutr*, *90*(5), 1295-1302. <https://doi.org/10.3945/ajcn.2009.28350>

Kumar, A., Sethi, V., Wagt, A., Parhi, R. N., Bhattacharjee, S., Unisa, S., R, S. R., Saraswat, A., Kejrewal, N., Shrivastava, M., Tripathy, L., Murira, Z., Vir, S., & Swabhimaan Study, G. (2023). Evaluation of impact of engaging federations of women groups to improve women's nutrition interventions- before, during and after pregnancy in social and economically backward geographies: Evidence from three eastern Indian States. *PLoS One*, *18*(10), e0291866. <https://doi.org/10.1371/journal.pone.0291866>

Lawande, A., Di Gravio, C., Potdar, R. D., Sahariah, S. A., Gandhi, M., Chopra, H., Sane, H., Kehoe, S. H., Marley-Zagar, E., Margetts, B. M., Jackson, A. A., & Fall, C. H. D. (2018). Effect of a micronutrient-rich snack taken preconceptionally and throughout pregnancy on ultrasound measures of fetal growth: The Mumbai Maternal Nutrition Project (MMNP). *Matern Child Nutr*, *14*(1). <https://doi.org/10.1111/mcn.12441>

Partap, U., Chowdhury, R., Taneja, S., Bhandari, N., De Costa, A., Bahl, R., & Fawzi, W. (2022). Preconception and periconception interventions to prevent low birth weight, small for gestational age and preterm birth: a systematic review and meta-analysis. *BMJ Glob Health*, *7*(8). <https://doi.org/10.1136/bmjgh-2021-007537>

Potdar, R. D., Sahariah, S. A., Gandhi, M., Kehoe, S. H., Brown, N., Sane, H., Dayama, M., Jha, S., Lawande, A., & Coakley, P. J. (2014). Improving women's diet quality preconceptionally and during gestation: effects on birth weight and prevalence of low birth weight—a randomized controlled efficacy trial in India (Mumbai Maternal Nutrition Project). *American Journal of Clinical Nutrition*, *100*(5), 1257-1268. <http://ajcn.nutrition.org/content/100/5/1257.full.pdf>

Potdar, R. D., Sahariah, S. A., Gandhi, M., Kehoe, S. H., Brown, N., Sane, H., Dayama, M., Jha, S., Lawande, A., Coakley, P. J., Marley-Zagar, E., Chopra, H., Shivshankaran, D., Chheda-Gala, P., Muley-Lotankar, P., Subbulakshmi, G., Wills, A. K., Cox, V. A., Taskar, V.,…Fall, C. H. (2014). Improving women's diet quality preconceptionally and during gestation: effects on birth weight and prevalence of low birth weight--a randomized controlled efficacy trial in India (Mumbai Maternal Nutrition Project). *Am J Clin Nutr*, *100*(5), 1257-1268. <https://doi.org/10.3945/ajcn.114.084921>

Sahariah, S. A., Potdar, R. D., Gandhi, M., Kehoe, S. H., Brown, N., Sane, H., Coakley, P. J., Marley-Zagar, E., Chopra, H., Shivshankaran, D., Cox, V. A., Jackson, A. A., Margetts, B. M., & Fall, C. H. (2016). A Daily Snack Containing Leafy Green Vegetables, Fruit, and Milk before and during Pregnancy Prevents Gestational Diabetes in a Randomized, Controlled Trial in Mumbai, India. *J Nutr*, *146*(7), 1453S-1460S. <https://doi.org/10.3945/jn.115.223461>

Stewart, C. P., Iannotti, L., Dewey, K. G., Michaelsen, K. F., & Onyango, A. W. (2013). Contextualising complementary feeding in a broader framework for stunting prevention. *Matern Child Nutr*, *9 Suppl 2*(Suppl 2), 27-45. <https://doi.org/10.1111/mcn.12088>

Taneja, S., Chowdhury, R., Dhabhai, N., Upadhyay, R. P., Mazumder, S., Sharma, S., Bhatia, K., Chellani, H., Dewan, R., Mittal, P., Bhan, M. K., Bahl, R., Bhandari, N., & Group, W. S. (2022). Impact of a package of health, nutrition, psychosocial support, and WaSH interventions delivered during preconception, pregnancy, and early childhood periods on birth outcomes and on linear growth at 24 months of age: factorial, individually randomised controlled trial. *BMJ*, *379*, e072046. <https://doi.org/10.1136/bmj-2022-072046>

UNICEF. (2021). *UNICEF Conceptual Framework on Maternal and Child Nutrition*.

Young, A. E., Kemp, J. F., Uhlson, C., Westcott, J. L., Ali, S. A., Saleem, S., Garces, A., Figueroa, L., Somannavar, M. S., Goudar, S. S., Hambidge, K. M., Hendricks, A. E., Krebs, N. F., & Women First Preconception Maternal Nutrition Trial, G. (2021). Improved first trimester maternal iodine status with preconception supplementation: The Women First Trial. *Matern Child Nutr*, *17*(4), e13204. <https://doi.org/10.1111/mcn.13204>
