# Supplementary material for: Effects of preconception nutrition interventions on pregnancy and birth outcomes in South Asia: a systematic review
Source: Lancet Reg Health Southeast Asia. 2025 Apr 24;36:100580. doi: 10.1016/j.lansea.2025.100580 (PMC12105516; doi:10.1016/j.lansea.2025.100580)
Supplement: CRD42023398938_prospero_record_intervention_review [file mmc2.pdf]

# Regional review of interventions addressing preconception nutrition in South Asia

Review methods were amended after registration. Please see the revision notes and previous versions for detail.

## Citation

Faith Miller, Naomi Saville, Vani Sethi. Regional review of interventions addressing preconception nutrition in South Asia. PROSPERO 2023 CRD42023398938 Available from:
https://www.crd.york.ac.uk/prospero/display_record.php?ID=CRD42023398938

## Review question

Which interventions addressing preconception nutrition have been undertaken in South Asia, and which factors affect their success?

## Searches [1 change]

1. Electronic searches

i. Ovid (MEDLINE and Global Health)

ii. EMBASE

iii. Global Index Medicus

iv. Web of Science

v. Cochrane Library

2. Reports published by state, national and international organisations

Studies published since the year 2000 will be identified, which are published in the English language.

## Search strategy

https://www.crd.york.ac.uk/PROSPEROFILES/398938_STRATEGY_20231117.pdf

## Types of study to be included [1 change]

Inclusion criteria

- Randomised and non-randomised controlled trials of interventions to improve preconception nutrition

- Data collected since 2000

- Data collected in South Asia

Exclusion criteria

- Intervention trials with no control group

- Data collected before 2000

- Data collected outside of South Asia

## Condition or domain being studied

Maternal malnutrition remains a huge problem in South Asia, where an estimated one in five women are too thin (BMI>18.5 kg/m2), one in ten women are too short (<145 cm) and anaemia is prevalent(1). This has adverse consequences for the growth and development of children, as maternal malnutrition contributes to low birth weight, infant death, stunting during childhood, and poor health throughout the life course(2-5).

Numerous interventions to improve the nutritional status of women and their children have been carried out in South Asia during the first 1, 000 days (from conception to when the infant is aged 2 years;6). However, these interventions have had limited success at improving the nutritional status of children, supporting calls for interventions to target the preconception period, which represents a promising and under-explored window in which to improve maternal and infant nutrition in South Asia(7-11).

This review will undertake an evidence-based review of published evidence on interventions addressing preconception nutrition in South Asia. Using evidence from randomised controlled trials published since 2000, we aim to:

i. Characterise interventions which have been carried out to improve preconception nutrition in South Asia, and

ii. Identify factors affecting the success of interventions to improve preconception nutrition in South Asia

## Participants/population

Inclusion criteria

- Women and girls in South Asia

- Women and girls who have taken part in an intervention addressing preconception nutrition

- Women and girls of reproductive age (10-49 years) who are not currently pregnant

The WHO definition of women of reproductive age is 15-49 years, however we have extended this to 10-49 years as in some communities in South Asia early marriage and childbearing is common meaning girls become pregnant and may be pre-conceptual before this age

- Women and girls having outcome data from a subsequent pregnancy

Exclusion criteria

- Women and girls who have not taken part in an intervention addressing preconception nutrition, or taking part in an alternative intervention

- Women and girls of reproductive age (10-49 years) without an outcome from a subsequent pregnancy

- Women of reproductive age (10-49 years) who are currently pregnant

- Women who are not of reproductive age (<10 or >49 years)

## Intervention(s), exposure(s) [1 change]

Interventions addressing preconception nutrition in South Asia:

- Social and Behaviour Change Communication (SBCC) including:

a) Nutrition counselling of women/girls and/or their partners / family members who are planning pregnancy or who are new-wed or preparing for marriage

b) Group approaches with newly-weds or couples and/or their 'guardians' (parents/in-laws)

- Micronutrient supplementation (especially iron and folic acid) to adolescent girls and/or premarital/newly wed women/girls or to women between pregnancies

- Food fortification (with iron, folic acid, iodine or other micronutrients)

- Interventions to strengthen the food environment to improve access to nutritious diets (including nutrition-sensitive agriculture)

- Social protection interventions (cash transfers, vouchers, food supplements or others)

Components of the nutrition education / counselling (as per the UNICEF 2021 guidance) might include but will not be limited to:

- Healthy eating and physical exercise to avoid excessive weight gain or loss

- Increase in protein energy intake to increase BMI in undernourished women/girls

- Consumption of a diverse diet including locally available, affordable nutritious foods and fortified foods

- Avoiding tea/coffee near meals and limiting intake

- Intake of iron and folic acid (IFA) or other supplements where available

- Intake of balanced protein energy supplements where available

- Handwashing at critical times

- Food hygiene – safe handing, preparation and storage

- Measures of preconception nutritional status may include (but are not limited to):

- Height

- BMI

- MUAC

- Weight

- Haemoglobin status

- Iron levels (including ferritin)

- Vitamins A, B12, and D levels

- Folate levels

- Zinc levels

As indicators for nutritional status, including:

- Thin

- Severely thin

- Overweight

- Obese

- Short stature

- Insufficient dietary diversity (<5 WDDS)

- Anaemia

- Micronutrient deficiency

Exclusion criteria

- Interventions delivered during pregnancy without an aspect being delivered pre-conceptually

- Interventions not addressing nutritional status

## Comparator(s)/control

Inclusion criteria

- Women and girls receiving an intervention addressing preconception nutrition

Exclusion criteria

- Women and girls not receiving an intervention addressing preconception nutrition, or receiving an alternative intervention

- Women and girls receiving an intervention outside of the preconception period

- Women and girls receiving an intervention addressing factors other than nutrition

## Context

Inclusion criteria

- Studies in South Asia addressing preconception nutrition among reproductive aged women and girls which fulfill the criteria laid out above

Exclusion criteria

- Articles published in languages other than English

- Studies reporting duplicate data

## Main outcome(s) [1 change]

At least one outcome from a subsequent pregnancy, including health and nutrition from the prenatal or neonatal period.

The primary outcome will be nutritional status among neonates, including but not limited to:

- Low birth weight

- Birth weight

- Preterm delivery

- Small for gestational age

Outcomes may also include maternal nutritional status during pregnancy, including but not limited to:

- Gestational weight gain

- Gestational diabetes

- Maternal anthropometry (weight, MUAC, height)

- Anaemia

- Micronutrient deficiencies

### Measures of effect

A number of effect measures will be accepted, including but not limited to relative risks, odds ratios, regression coefficients, risk difference, and ‘number needed to treat’. For descriptive analysis we will also include numerical index (from $\chi^2$) and correlations.

## Additional outcome(s)

None

## Data extraction (selection and coding)

Two review authors will independently screen the titles and abstract and subsequently screen the full-texts to determine eligibility. Disagreements will be resolved by discussing and, if necessary, consulting with the third review author.

For each paper included, one author will extract data for review, including:

- Study details, including author, year, setting, and study design

- Details of intervention (including timing, delivery, engagement, and proposed mechanism of action)

- Exposures relating to preconception nutrition

- Outcomes relating to pre/post-natal health or nutritional status:

The primary outcome will be nutritional status among infants, including but not limited to:

- Low birth weight

- Birth weight

- Preterm delivery

- Small for gestational age

Other outcomes may include nutritional status among children (up to 19 years), including but not limited to:

- Weight-for-age z score

- Underweight

- Height-for-age z score

- Stunting

- Overweight

- Wasting

- Weight-for-height z score

- BMI-for-age z score

Outcomes may also include maternal nutritional status during pregnancy, including but not limited to:

- Gestational weight gain

- Gestational diabetes

- Maternal anthropometry (weight, MUAC, height)

- Anaemia during pregnancy

- Potentially mediating factors (as identified by authors of the primary studies)

- Author reflections on factors affecting the success/failure of preconception nutrition interventions, including factors relating to leadership, financing, governance, supplies, and capacity

- Author recommendations on strategies to improve preconception nutrition in South Asia

## Risk of bias (quality) assessment

Two authors will independently assess risk of bias using the Cochrane risk of bias tool for randomised trials (RoB 2)

## Strategy for data synthesis [3 changes]

Data synthesis

We will synthesise the following characteristics narratively by tabulating the following aspects and comparing narratively across studies and contexts, and over time (and diagrammatically representing as appropriate):

- Study characteristics including context

- Study sample characteristics

- Details of intervention (including randomisation and characteristics of intervention/control groups)

Which interventions have been undertaken to address preconception nutrition in South Asia, including whether the interventions are nutrition-sensitive or nutrition-specific, and which level of the UNICEF framework the interventions address (fig.1)

- How preconception nutrition is conceptualised within the literature.

- Type(s) of exposure explored

- Type(s) of outcomes reported

To summarise how pre/post-natal nutritional status is targeted within the literature.

- Type(s) of mediators explored

To summarise how biological, behavioural, and social pathways are conceptualised within the literature

- Effect of intervention

Summarise descriptively the effect of interventions addressing preconception nutrition and pre/post-natal nutritional status. This will involve diagrammatically representing associations between interventions and preconception nutrition and/or birth outcomes, grouping interventions by type and where they lie in the UNICEF framework.

We will draw out and summarise potential mechanisms of action as these become evident and produce a conceptual diagram of preconception nutrition interventions, their mode of action and impacts, highlighting gaps in knowledge.

- Strengths/limitation of exposure/intervention

Summarise challenges faced in addressing preconception nutrition descriptively

## Analysis of subgroups or subsets

If possible, we will undertake subgroup analysis exploring the effect of interventions at the women, household, and community level. If there is sufficient homogeneity between interventions, we may undertake a meta-analysis of the effectiveness of preconception nutritional interventions, however we do not expect this to be possible due to the limited evidence base.

## Contact details for further information

Faith Miller

rmjwffm@ucl.ac.uk

## Organisational affiliation of the review

University College London, Institute for Global Health

https://www.ucl.ac.uk/global-health/institute-global-health

## Review team members and their organisational affiliations [1 change]

Miss Faith Miller. University College London

Dr Naomi Saville. University College London

Dr Vani Sethi. UNICEF

## Type and method of review

Intervention, Narrative synthesis, Systematic review

## Anticipated or actual start date [1 change]

21 August 2023

## Anticipated completion date [1 change]

31 December 2023

## Funding sources/sponsors

UNICEF Regional Office for South Asia (ROSA)

## Conflicts of interest

## Language

English

## Country

England, Nepal

## Stage of review

Review Ongoing

## Subject index terms status

Subject indexing assigned by CRD

## Subject index terms

Asia, Southern; Female; Humans; Maternal Nutritional Physiological Phenomena; Nutritional Status

## Date of registration in PROSPERO

23 August 2023

## Date of first submission

12 February 2023

## Stage of review at time of this submission

The review has not started

| Stage | Started | Completed |
| --- | --- | --- |
| Preliminary searches | No | No |
| Piloting of the study selection process | No | No |
| Formal screening of search results against eligibility criteria | No | No |
| Data extraction | No | No |
| Risk of bias (quality) assessment | No | No |
| Data analysis | No | No |

## Revision note

We have edited again our protocol to focus our review on pregnancy/neonatal/birth outcomes (excluding child outcomes up to 19 years) as the review was too broad, and provide more specific terms in our searches

*The record owner confirms that the information they have supplied for this submission is accurate and complete and they understand that deliberate provision of inaccurate information or omission of data may be construed as scientific misconduct.*

*The record owner confirms that they will update the status of the review when it is completed and will add publication details in due course.*

## Versions

23 August 2023

29 August 2023

20 November 2023
